# New antifungal and less toxic zwitterionic derivatives of 26-membered polyene antibiotic – pimaricin containing *N*-benzyl and *N*-alkyl moieties and their molecular mechanism

**DOI:** 10.1080/14756366.2026.2724228

**Published:** 2026-09-10

**Authors:** Ewelina Smolarz, Adam Buczkowski, Wojciech Schilf, Anita Ciesielska, Paweł Stączek, Piotr Ruszkowski, Maria Gdaniec, Franz Bartl, Piotr Przybylski

**Affiliations:** a Faculty of Chemistry, Adam Mickiewicz University, Poznan, Poland; b Department of Physical Chemistry, Faculty of Chemistry, University of Lodz, Lodz, Poland; c Institute of Organic Chemistry, Polish Academy of Sciences, Warsaw, Poland; d Department of Molecular Microbiology, Faculty of Biology and Environmental Protection, University of Lodz, Lodz, Poland; e Department of Pharmacology, Poznan University of Medical Sciences, Poznań, Poland; f Institut für Biologie, Humboldt Universität zu Berlin, Berlin, Germany

**Keywords:** Natamycin, macrolides, antifungal, cytotoxicity, membrane model

## Abstract

*N*-derivatives of pimaricin (**PIM**) were synthesised as zwitterions or in a non-ionic form, influencing water solubility and toxicity in normal cells (HDF). The introduction of benzyl-*O*-aliphatic (**15**) and methylene-nitrophenol (**7**) moieties into the polyene resulted in high and broad-spectrum antifungal activities, even better than known antifungal agents. The promising antifungal potency of **7** is attributed to formation of a 6:1 complexes with ergosterol (**ERG**) as indicated by ITC, FT-IR and NMR studies. The self-assembly of 6:1 complexes into greater structures of polar channels explains antifungal activity of **7**, as suggested by MOG-PM6 calculations. In contrast, fluorobenzyl derivative **3** despite weak antifungal potency, revealed unexpected high anticancer activity in PC-3 and SKOV-3 cells, at improved selectivity (IC_50_ = 0.6 µM; SI ∼ 3–4) and lower toxicity, compared to **PIM**. The activity of *N*-derivatives of **PIM** towards fungi strains or cancer cells, depending on polar head structure, is discussed.

## GRAPHICAL ABSTRACT

**Figure:**
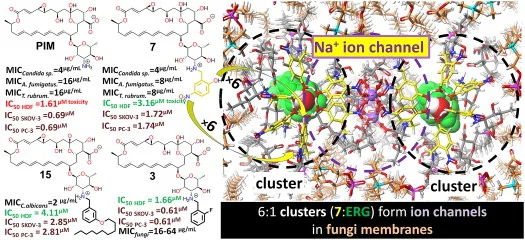

## Introduction

**Figure 1.**
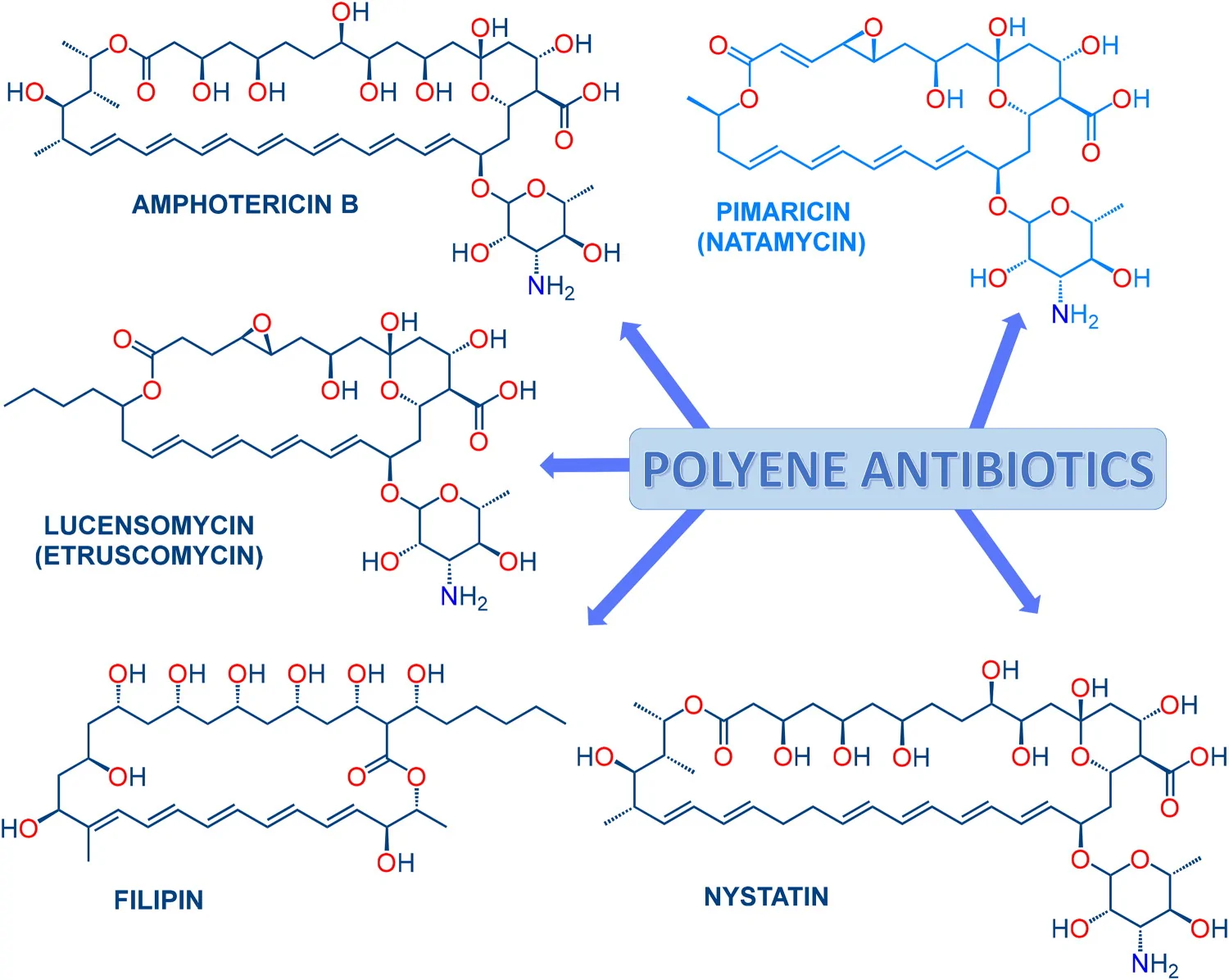
The most common examples of antifungal polyene macrocyclic antibiotics.

Polyene antibiotics constitute a large group of natural compounds, produced mainly by *Streptomyces* strains and are highly effective against fungal pathogens such as *Candida, Aspergillus,* and *Fusarium*^1–5^. Representative polyene members include amphotericin B, nystatin, pimaricin (**PIM**, natamycin), filipin, and lucensomycin (etruscomycin)^5–8^ (Figure 1). The structure of polyene antibiotics contains a macrolactone ring comprising polyene chain (chromophore) with three to eight double bonds, leading to classification as trienes, tetraenes, pentaenes, etc^9^. Pimaricin (**PIM**) belongs to tetraene subgroup of antibiotics and possesses a relatively small 28-membered macrolactone ring (Figure 1). Like many polyenes **PIM** contains common amino-saccharide called mycosamine, which plays an important role in biological activity^10^. It should be also noted that antifungal activity is not restricted to polyenes as several lactone-containing macrolides of smaller rings exhibit similar properties^11^. Fungal infections represent an increasing global health problem, particularly among immunocompromised patients, including transplant recipients and patients with cancer or AIDS^12^^,^^13^. In addition fungal sepsis cases, have risen significantly in the 21^st^ century^14–16^. Current antifungal therapies based on polyenes remain rather limited to the amphotericin B or nystatin of poor physico-chemical properties, showing deleterious nephrotoxicity and inducing fungi resistance^17^. In contrast resistance to polyene antibiotics of smaller aglycone ring such as **PIM** remains relatively rare, highlighting their therapeutic potential^18–20^. **PIM** is widely used as food preservative, but also is applied as a drug for different fungal infections in human as e.g. aspergillosis or candidiasis, which are especially a problem for those patients with weakened immune systems^21–24^. Furthermore, **PIM** is commonly used for the treatment of fungal corneal infections (known as keratitis) and conjunctivitis^21^^,^^22^^,^^24^^,^^25^.

However, the clinical utility of **PIM** is limited by rather poor solubility in water and organic solvents, as well as toxic effects, which pose a challenge in the development of new potent antifungals based on **PIM** scaffold^6^^,^^24^. Previous structural modifications of **PIM** were mainly focused on the carboxyl group through amidation reactions based on introducing alkyl or aromatic substituents^22^. Among the amide derivatives of **PIM**, compounds containing short alkyl chains and exhibiting average water solubility were the most biologically attractive^22^. Modifications of **PIM** mycosamine saccharide were achieved using the Kabachnik-Fields reactions yielding α-aminophosphonates and hydrophosphoryl derivatives with enhanced activity against *Candida krusei* strain^24^^,^^26^. Earlier obtained aniline-type **PIM** derivatives by S_N_Ar reaction with fluorobenzenes, also resulted in antifungal activity against *Candida* species^23^. Phosphonylbenzyl derivatives and nitrophenyl substituted analogs exhibited lower MFC values against *Candida* species than native **PIM**^24^^,^^26^. The antifungal activity of polyenes as amphotericin B and nystatin is generally attributed to their interactions and association with ergosterol (**ERG**) in fungal cell membranes, resulting in enhanced membrane permeability and leakage of different metal cations^27–30^. Breukink et al in 2008 suggested **PIM** acts primarily through a slightly different mechanism, which, although based on specific interactions with **ERG**, results in blocking the fusion of isolated vacuoles without affecting the membrane permeability^31^. Recently, also the third-type mechanism of antifungal activity, independent of **ERG** binding, has been proposed for the polyene mandimycin, which acts by extracting phospholipids from the fungi cell membrane^32^. To date, the molecular basis of **PIM**-**ERG** interactions remains insufficiently understood. Additionally to the antifungal activity, **PIM** has shown antiparasite properties against *Acanthamoeba keratitis*^33^ and antiviral potency^34^. The debateable presentation of the earlier-obtained **PIM** derivatives in a non-ionic form^23^^,^^24^^,^^26^^,^^35^, prompted us to investigate a real structure of **PIM** derivatives. Since acid-base equilibria strongly influence biological activity of many macrolide antibiotics^36–38^, we aimed to determine how structural modifications at amino group of mycosamine affect structure, molecular polarity (water solubility/lipophilicity), interactions with **ERG**, self-assembly behaviour, antifungal potency and toxicity. The modification of “polar head” of **PIM** was a plan to achieve derivatives of comparable or greater length to those of nystatin or amphotericin, in order to realisation of membrane-type mechanism of antifungal potency.

## Experimental section

### General experimental

Pimaricin (**PIM**) was purchased from Pol-Aura catalogue number PA-03-5100-A#1G CAS: 7681-93-8. The solvents DMSO-*d_6_* for NMR spectroscopic measurements, as well as 4-ethynylbenzaldehyde, 3-ethynylbenzaldehyde, 2-fluorobenzaldehyde, 3-fluorobenzaldehyde, 4-(trifluoromethyl)benzaldehyde, 4-nitrobenzaldehyde, 2-hydroxy-5-nitrobenzaldehyde, 5-bromosalicylaldehyde, 4-formylbenzoic acid, 3-formylbenzoic acid, 4-(4-hydroxybuy-1-yn-1-yl)benzaldehyde, 4-(4-methoxyphenyl)benzaldehyde, 4-propylbenzaldehyde, 2-(2-hydroxyetoxy)benzaldehyde, 4-(decyloxy)benzaldehyde, benzyloxyacetaldehyde, 2-naphthaldehyde, 9-anthracenecarboxyaldehyde, cyclopropanecarboxyaldehyde, 2-methylbutyraldehyde, decanal, dodecyl aldehyde, octanal, acetaldehyde, sodium cyanoborohydride, hydroxybenzotriazole, *N,N’*-dicyclohexylcarbodiimide, methanol, TEA and DMF used for the synthesis of new PIM derivatives were purchased from Merck. Dichloromethane was purchased from Chemland.

### General procedure for synthesis of PIM derivatives 1–24

**PIM** (100 mg, 0.15 mmol) was dissolved in 6 ml of a MeOH/DCM mixture (2:1). Then, the aldehyde was added (0,18 mmol): 4-ethynylbenzaldehyde (**1**), 3-ethynylbenzaldehyde (**2**), 2-fluorobenzaldehyde (**3**), 3-fluorobenzaldehyde (**4**), 4-(trifluoromethyl)benzaldehyde (**5**), 4-nitrobenzaldehyde (**6**), 2-hydroxy-5-nitrobenzaldehyde (**7**), 5-bromosalicylaldehyde (**8**), 4-formylbenzoic acid (**9**), 3-formylbenzoic acid (**10**), 4-(4-hydroxybut-1-yn-1-yl)benzaldehyde (**11**), 4-(4-methoxyphenyl)benzaldehyde (**12**), 4-propylbenzaldehyde (**13**), 2-(2-hydroxyetoxy)benzaldehyde (**14**), 4-(decyloxy)benzaldehyde (**15**), benzyloxyacetaldehyde (**16**), 2-naphthaldehyde (**17**), 9-anthracenecarboxyaldehyde (**18**), cyclopropanecarboxyaldehyde (**19**), 2-methylbutyraldehyde (**20**), decanal (**21**), dodecyl aldehyde (**22**), octanal (**23**), acetaldehyde (**24**). Then TEA (0.15 mmol) was added to the mixture. The mixture was stirred at room temperature for 1.5 h, after which NaBH_3_CN was added and stirring was continued at room temperature for 18–24 h. The progress of the reaction was monitored by TLC chromatography using a dichloromethane/methanol mixture as the eluent. Before evaporation, the product was extracted with ethyl acetate and brine (with the exception of compound 10, which was directly evaporated on a vacuum evaporator). The organic layer was then washed with NaHCO_3_ and subsequently evaporated. The products were purified by column chromatography using a mixture of dichloromethane/methanol as the eluent. After evaporation of the solvent, the products were obtained as beige powders (with the exception of compounds **7**, **8**, and **18**, which were yellow powders).

### Procedure for synthesis of derivative 25

Derivative **15** (20 mg, 0.022 mmol) was dissolbed in 2 ml of dimethylformamide (DMF). The mixture was cooled to 0 °C. Hydroxybenzotriazole (HOBt) (0.022 mmol) was added, and the mixture was stirred for 20 min at 0 °C. Next, *N,N’*-dicyclohexylcarbodiimide (0.022 mmol) was added to the mixture, and stirring was continued for 1 h at 0 °C. Then the three-fold excess 4-dimethylaminobenzylamine dihydrochloride (0.066 mmol) was added to the mixture with TEA (0.139 mmol) and stirred at room temperature for 72 h. The progress of the reaction was monitored by TLC chromatography using a dichloromethane/methanol mixture as the eluent. Before evaporation, the product was extracted with ethyl acetate and brine. The organic layer was then washed with NaHCO_3_ and subsequently evaporated. The products were purified by column chromatography using a mixture of dichloromethane/methanol as the eluent. After evaporation of the solvent, the product was obtained as beige powder.

### FT-IR measurements

The FT-IR spectra of **PIM** analogs **1–25** were recorded in the KBr pellet and in dmso_aq_ solution (*c* = 0.05 M). FT-IR measurements were performed at a spectrometer equipped with a DTGS detector and two columnar purge gas generator at resolution 2 cm^−1^, NSS = 150, range 4000–400 cm^−1^. The Happ-Genzel apodization function was used.

### NMR measurements

The solution state ^1^H and ^13^C NMR measurements of **PIM** and compounds **1–25** were performed in deuterated dimethyl sulfoxide DMSO-*d_6_* (with traces of water) usually at temperature of 40 °C on Varian-Agilent 600 VNMRS and Varian-Agilent 500 VNMRS spectrometers (Agilent Technologies, Inc., Santa Clara, CA, United States) using 5 mm probeheads equipped with Z-gradient coils. Additionally, some spectra were measured at: T = 243.15, 253.15, 273.15, 298.15 K; For the 1D carbon spectrum, a 500 MHz instrument with Auto XDB (direct configuration) probehead was used. For ^1^H and homo- and heteronuclear correlation spectra run on Varian-Agilent 600 spectrometer the indirect Auto XID probehaed was applied.

For Varian-Agilent 600 spectrometer: the operating frequency for ^1^H measurement was 599.84 MHz; typical spectral width sw = 8500 Hz, acquisition time at = 4.2 s; relaxation delay d_1_ = 1.0 s. TMS was used as the internal standard. No window function or zero filling were used for ^1^H 1D spectra. Digital resolution was 0.2 Hz/point.

The operating frequency for ^13^C{1H} measurement run on Varian Agilent 600 instrument was 150.84 MHz; spectral width sw = 43 743.6 Hz, acquisition time at = 1.2 s; relaxation delay d_1_ = 0.5 s; TMS was used as the internal standard. Line broadening parameters of 0.5 or 1 Hz were applied.

For Varian-Agilent 500 spectrometer: the operating frequency for ^1^H measurement was 499.82 MHz; spectral width sw = 8012.8 Hz, acquisition time at = 4.0 s; relaxation delay d_1_ = 1.0 s;; TMS was used as the internal standard. No window or zero filling were used. Digital resolution was 0.2 Hz/point.

The ^1^H and ^13^C{1H} NMR resonances in solution were unambiguously assigned on the basis of the ^1^H-^13^C gHSQC,^1^H-^13^C gHMBC,^1^H-^1^H COSY, ^1^H-^1^H ROESY, ^1^H-^1^H NOESY correlation spectra performed on Varian Agilent 600 MHz spectrometer.

^15^N NMR correlation spectra were run on 600 MHz instrument applying gHMBC and gHSQC sequences using inverse probehead. The resonance frequency for ^15^N measurements was 60.782 MHz. The nitromethane was used as the chemical shift standard compound.

For acquisition and processing of all NMR spectra the OpenVnmrJ software package was used.

## ESI MS analyses

The Electrospray Ionisation (ESI) mass spectra were recorded on ZQ Waters spectrometer using in the *m/z* range from 100 to 2000.

### Isothermal titration calorimetry (ITC)

Using isothermal titration calorimetry at 25 °C (VP-ITC calorimeter equipped with Hastelloy C-276 cells, MicroCal) the heat effects of titrations of 20 μM ergosterol (**Erg**, 396.65 g mol^−1^) solutions with 1000 μM solutions of polyenes as ligands, respectively pimaricin (**PIM**, MW 665.74 g mol^−1^) and its 8 derivatives (**7**, MW 816.85 g mol^−1^, **12**, MW 861.98 g mol^−1^, **15**, MW 912.13 g mol^−1^, **17**, MW 805.92 g mol^−1^, **19**, MW 719.83 g mol^−1^, **21**, MW 806.00 g mol^−1^, **23**, MW 890.17 g mol^−1^ and **25**, MW 1044.34 g mol^−1^) in DMSO solvent were measured. Ergosterol solution with volume 1427.5 μl (in working cell) was titrated with adding 5 μl aliquots (from the syringe) in total 280 μl volume of pimaricin or its derivatives. The duration time of each injection was 10 s and the time between successive injections was 1200s. Independently the dilution heat effects of pimaricin and its derivatives (from the syringe) into the solvent (in the cell) were measured, maintaining the same parameters as during the proper titration.

Heat effects of direct interactions q of studied ligands with ergosterol were calculated by subtracting the heat effects of ligand dilution from the heat effects of proper titration. Such obtained isotherms were next fitted with non-linear regression method (Origin 7.0 MicroCal) using One Set of Sites model ([ITC Date Analysis in Origin – Tutorial Guide. Northampton: MicroCal, 2004]) to obtain the binding parameters: stoichiometry n of the formed complexes and standard enthalpy ΔH. Dissociation $K_{d}$ constants of ligands from their complexes with ergosterol were recalculated from binding K constants, using equation: $K_{d}=1/K$. Standard entropy ΔS and Gibbs free energy ΔG of binding were calculated from the basic thermodynamic relationships: ΔG=−RT ln K=ΔH−TΔS, where: T – absolute temperature and R – gas constant.

### Evaluation of antifungal activity

#### Inoculum preparation

*Inocula* were prepared according to CLSI recommendations (M27-A3 for yeast and M-38_A2 for filamentous fungi) for broth microdilution antifungal susceptibility testing, with organism-specific modifications for yeasts and filamentous fungi.^39^^,^^40^ All suspensions were prepared in sterile 0.85% saline under aseptic conditions and used immediately after standardisation.

*Candida* isolates were subcultured on Sabouraud dextrose agar and incubated at 35 °C for 24 h to obtain fresh colonies. Several well-isolated colonies were transferred into sterile 0.85% saline and thoroughly mixed to obtain a homogeneous yeast cell suspension. The turbidity of the suspension was adjusted spectrophotometrically at 530 nm to an optical density of 0.12–0.15, corresponding approximately to 2.5–5 × 10⁶ CFU/mL. The standardised suspension was then diluted in RPMI 1640 medium to obtain the final inoculum concentration required for the broth microdilution assay.

*Aspergillus* isolates were cultured on potato dextrose agar and incubated at 35 °C for up to 7 days, or until adequate sporulation was obtained. Sporulating colonies were covered with approximately 1 ml of sterile 0.85% saline. When necessary, one drop of Tween 20 was added to facilitate preparation of a homogeneous conidial suspension. Conidia were gently dislodged from the colony surface using a sterile transfer pipette tip or sterile swab. The resulting suspension was transferred to a sterile tube and allowed to stand for 3–5 min to permit larger hyphal fragments and heavy particles to settle. The upper homogeneous conidial suspension was collected and vortexed briefly. The optical density was adjusted spectrophotometrically at 530 nm to 0.09–0.13, corresponding approximately to 1–5 × 10⁶ CFU/mL. The standardised suspension was subsequently diluted in RPMI 1640 medium to obtain the working inoculum.

*Fusarium* isolates were cultured on potato dextrose agar to induce conidial formation. Cultures were incubated at 35 °C for 48–72 h and then, if required, maintained at 25–28 °C until day 7 to obtain sufficient sporulation. Conidia were harvested by covering the colony surface with approximately 1 ml of sterile 0.85% saline and gently scraping the sporulating area with a sterile transfer pipette tip or sterile swab. The suspension was transferred to a sterile tube and left to stand for 3–5 min to allow larger hyphal fragments to sediment. The upper homogeneous conidial suspension was collected and mixed briefly. The optical density was adjusted at 530 nm to 0.15–0.17, corresponding approximately to 1–5 × 10⁶ CFU/mL. The standardised suspension was diluted in RPMI 1640 medium before inoculation of the microdilution plates.

Dermatophyte isolates, including *Trichophyton* spp., were grown on potato dextrose agar. For poorly sporulating isolates, particularly *Trichophyton rubrum*, oatmeal agar was used to promote conidial formation. Cultures were incubated at 30 °C for 4–5 days or until sufficient conidial growth was observed. After incubation, colonies were covered with approximately 1 ml of sterile 0.85% saline, and conidia were gently released from the colony surface using a sterile transfer pipette tip or sterile swab. The suspension was allowed to stand for 5–10 min to enable larger hyphal fragments to settle. The upper homogeneous suspension was collected and standardised spectrophotometrically. For *Trichophyton* spp., the optical density was adjusted to 0.08–0.10, corresponding approximately to 2–5 × 10⁶ CFU/mL. The standardised suspension was then diluted in RPMI 1640 medium before use in the broth microdilution assay.

#### Final inoculation, incubation and endpoint reading

Standardised microbial suspensions were diluted in RPMI 1640 medium to obtain the appropriate final inoculum concentration in the wells. Each well of the microdilution plate was inoculated with 0.1 ml of the corresponding diluted suspension. After inoculation, microdilution plates were incubated without agitation under conditions appropriate for the tested fungal group. Plates containing Candida isolates were incubated at 35 °C and examined after 24 h of incubation. For filamentous fungi, including Aspergillus spp. and Fusarium spp., microdilution plates were incubated at 35 °C without agitation. MIC endpoints for Aspergillus spp. and Fusarium spp. were read after 46–50 h of incubation, provided that adequate growth was observed in the drug-free growth control wells. For dermatophyte isolates, including Trichophyton spp., plates were incubated at 35 °C without agitation and evaluated after four days of incubation. Growth control wells were inspected before endpoint determination to confirm sufficient fungal growth.

### Evaluation of toxicity and anticancer activity

Human cancer cells SKOV-3 (ovarian cancer cell line) were cultured in McCoy’s Modified Medium. Human cancer cells PC-3 (human prostate cancer cell line) were cultured in an F–12 K medium. Human Dermal Fibroblasts cell line (HDF) and non-proliferated Human Umbilical Vein Endothelial Cells (HUVEC) were cultured in Fibroblast Basal Medium. Each medium was supplemented with 10% foetal bovine serum, 1% *L*-glutamine, and 1% penicillin/streptomycin solution. The cell lines were kept in the incubator at 37 °C. The optimal plating density of cell lines was determined to be 5 × 10^4^. All the cell lines and mediums were obtained from the American Type Culture Collection (ATCC) supplied by LGC-Standards. The protein-staining SRB (Sigma-Aldrich) microculture colorimetric assay, developed by the National Cancer Institute (USA) for *in vitro* antitumor screening was used in this study, to estimate the cell number by providing a sensitive index of total cellular protein content, being linear to cell density. The monolayer cell culture was trypsinized and the cell count was adjusted to 5 × 10^4^ cells. To each well of the 96 well microtiter plate, 0.1 ml of the diluted cell suspension (approximately 10 000 cells) was added. After 24 h, when a partial monolayer was formed, the supernatant was washed out and 100 μL of six different compound concentrations (0.1, 0.2, 1, 2, 10, and 20 μM) were added to the cells in microtiter plates. The tested compounds were dissolved in DMSO (containing 10% of water) (100 μL) and the content of DMSO did not exceed 0.1%; this concentration was found to be non-toxic to the cell lines. The cells were exposed to compounds for 72 h at 37 °C in a humidified atmosphere (90% RH) containing 5% CO_2_. After that, 25 μL of 50% trichloroacetic acid was added to the wells and the plates were incubated for 1 h at 4 °C. The plates were then washed out with distilled water to remove traces of medium and next dried by the air. The airdried plates were stained with 100 μL of 0.4% sulforhodamine B (prepared in 1% acetic acid) and kept for 30 min at room temperature. The unbound dye was removed by rapidly washing with 1% acetic acid and then air dried overnight. The protein-bound dye was dissolved in 100 μL of 10 mM unbuffered Tris base (pH 10.5) for optical density determination at 490 nm. All cytotoxicity experiments were performed three times. Cell survival was measured as the percentage absorbance compared to the control (nontreated cells). Actinomycin D (**ActD**), mitomycin (**mitC**), **PIM** and **NYS** were used as the internal standards. Additionally, biological assays were performed in the Human Dermal Fibroblasts cell line (HDF) with the aim to evaluate the cytotoxicity of PIM and its analogues in healthy cells. Results of anticancer studies of novel analogues of PIM are shown in Table 3. SI indexes were calculated from equation SI = IC_50_ normal cell line HDF/IC_50_ and HUVEC/IC_50_ respective cancerous cell line (Table 3). A beneficial SI > 1.0 indicates a compound with efficacy against cancer cells greater than the toxicity against normal cells.

### MOG-PM6 calculations

Single molecule models of pimaricin (**PIM**) and its derivatives were built on X-ray determined structure of **PIM** reported in the current work and adding the *N*-substituents (Figure 4(a–c)). The mutual arrangement of aglycone, mycosamine, and substituent parts were assumed on the basis of ^1^H-^1^H ROESY correlations and initially optimised by molecular mechanics (MM3 method). The final single molecule models of **PIM** derivatives were optimised stepwise with MOG-PM6 algorithm dedicated to huge molecules with energy gradient not exceeding 2 kcal/mol in one calculation cycle (*Scigress F.J. 2.6, EU 3.1.9* package)^41^. In this way obtained models of **PIM** [**PIM** (Figure 4(d); E = −282.73 kcal/mol) and its derivatives: **3** (Figure S33; E = −304.83 kcal/mol), **4** (Figure S42; E = −302.11 kcal/mol), **7** (Figure 6; E = −332.31 kcal/mol), **10** (Figure S223; E = −356.45 kcal/mol), **12** (Figure S112; E = −361.64 kcal/mol), **15** (Figure 6; E= −374.70 kcal/mol), **18** (Figure S161; E = −382.05 kcal/mol), **21** (Figure 7; E = −353.39 kcal/mol), **23** (Figure 7; E = −379.01 kcal/mol)] represent local-minima energy conformations, being in agreement with the recorded NMR data in solution. Calculations of two-types 6:1 complexes between **7** and **ERG** (Figure 8(a,b)), were performed with the use of earlier-optimised **7** and available X-ray structure of ergosterol **ERG** (CCDC 146357)^42^ at 18 347 calculation cycles, with energy gradient not exceeding 5 kcal/mol at one step by MOG-PM6 method. Membrane model was built on the basis of optimised structures of 6:1 clusters (**7**:**ERG**) and introduction of POPC (1-palmitoyl-2-oleoylphosphatidylcholine) molecules, arranged according to polarity of their portions. Membrane molecular model of cluster, with incorporated 6:1 complex (**7**:**ERG**) was obtained in 39 732 calculation cycles with energy gradient not exceeding 7 kcal/mol at one step (Figure 9(a)). A more complexed model of self-assembled clusters (Figure 9(b,c)) was obtained by optimised association of the two 6:1 clusters (**7**:**ERG**), forming Na^+^_(H2O)_ channel *via* interactions between polyol faces of the four molecules of **7** per one side of the membrane (Figure 10(a,b)), and next adding of the lipid matrix (POPC) surrounding the channel according to the earlier reported 7:3 ratio between phospholipids and **ERG** in fungi membranes. The initial optimisation of the model (∼ 8000 atoms) was performed by molecular mechanics (MM3) and next the model was subjected to gradual mutual fitting between the channel and the phospholipid matrix (112 295 calculation cycles) with energy gradient not exceeding ∼ 11 kcal/mol at the one step) – calculation performed by MOG-PM5 method (*Scigress F.J. 2.6, EU 3.1.9* package)^41^.

### X-ray studies of PIM crystals

Two solvated crystal forms were obtained by recrystallization of **PIM** from methanolic solutions. The initially obtained solvate, **PIM1,** formed needle-shaped crystals that were very unstable in the air and cracked when cooled. The crystal was mounted on a plastic loop with small amount of perfluoropolyether. The diffraction data were collected at 130 K up to 1 Å resolution with an Agillent SuperNova diffractometer using hi-flux micro-focus Nova Cu-Kα radiation (λ = 1.54184 Å) and Apex CCD detector. Data collection and reduction were performed with the CrysAlis Pro software^43^. The structure was solved by intrinsic phasing method using the SHELXT program^44^ and refined by full-matrix least-squares method on F^2^ with SHELXL^45^. All calculations were carried out within OLEX-2^46^. The assumed absolute structure of **PIM** conforms with its known absolute configuration. A fragment of the aliphatic chain within the macrocycle was disordered over two sites. In the refinement process restraints were imposed on the geometry and occupancy factors of the disordered fragment. Within the disordered fragment, the atoms with the lower occupancy factor were refined isotropically. Solvent molecules could not be identified reliably therefore a disordered solvent contribution was extracted from structure factors using the “Solvent mask” routine of Olex-2 with the probe radius of 1.2 Å and a resolution of 0.2 Å. A solvent accessible area in this structure consists 21% of the unit-cell volume (Figure S229). The hydrogen atom positions from C-H and N-H groups were determined geometrically and were refined in the riding-model approximation, with U_iso_(H) = 1.2 U_eq_(N,C) and U_iso_(H) = 1.5 Ueq(C_methyl_). The hydrogen atom positions from O-H groups were determined considering formation of possible hydrogen bonds. A rather high discrepancy factors for the crystal structure of **PIM1** are related to a poor quality of the diffraction data. Our attempts to repeat crystallisation of **PIM1** and collect better diffraction data unfortunately failed as a new form, **PIM2**, was obtained in subsequent crystallizations. These crystals were also needle-shaped however more stable when removed from the mother liquor. The diffraction data for **PIM2** were collected at 100 K with a Bruker Quest D8 diffractometer using IµS DIAMOND II microfocus Cu-Kα source and PHOTON I detector. Data collection and reduction were performed with APEX5^47^. The structure was solved by intrinsic phasing method using the SHELXT program^44^ and refined by full-matrix least-squares method on F^2^ with SHELXL^45^. In case of **PIM2**, the **PIM** molecule was ordered and solvent molecules were easily located, despite their disorder, indicating PIM·(CH_3_OH)_2.81_·(H_2_O)_0.58_ stoichiometry.

### Determination of compounds chemical stability at different pH

Each of compounds (0.5 mg) with 2-thiophenecarboxylic hydrazide (6 mg) as internal standard were dissolved in 100 µM of DMSO followed by addition of 900 µM of different pH buffer solutions (pH 2.00, 6.86, 9.18). Chromatograms were recorded right after preparation of the mixture and at time intervals. For each injection 100 µM of prepared mixture was added to vial followed by addition of 900 µM of acetonitrile. HPLC separations were performed with a Dionex Ultimate 3000 equipped with LPG-3400 SD gradient pump using Waters C-8 X-Bridge CB 150 × 3 mm (3.5 μm) (column, TCC-3000SD thermostat to columns (column temp. equal 25 °C) and Dionex VWD-3400 RS variable wavelength UV-Vis detector. The analytical wavelength was λ*max* = 303 nm. The flow rates were 0.5 ml/min with injection volumes 10 μL in acetonitrile and the mobile phase 55:40:5 H*2*O/CH*_3_*CN/NH_4_OH.

## Results and discussion

### Synthesis and structure/conformation of PIM and its new derivatives

**Figure 2.**
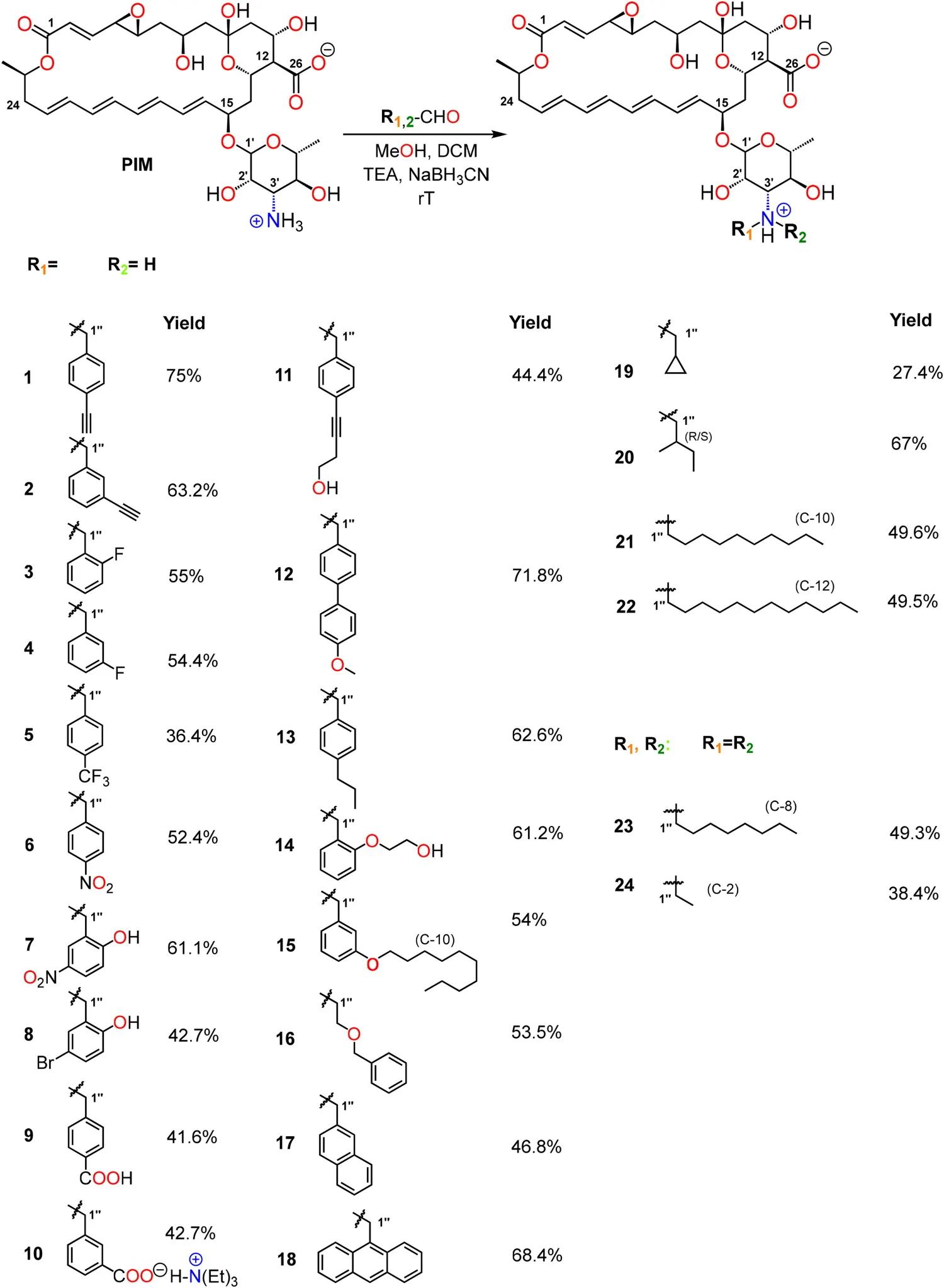
Synthesis of *N*-alkylamine derivatives at the mycosamine portion of **PIM** and yields after column chromatographic separations.

**Figure 3.**
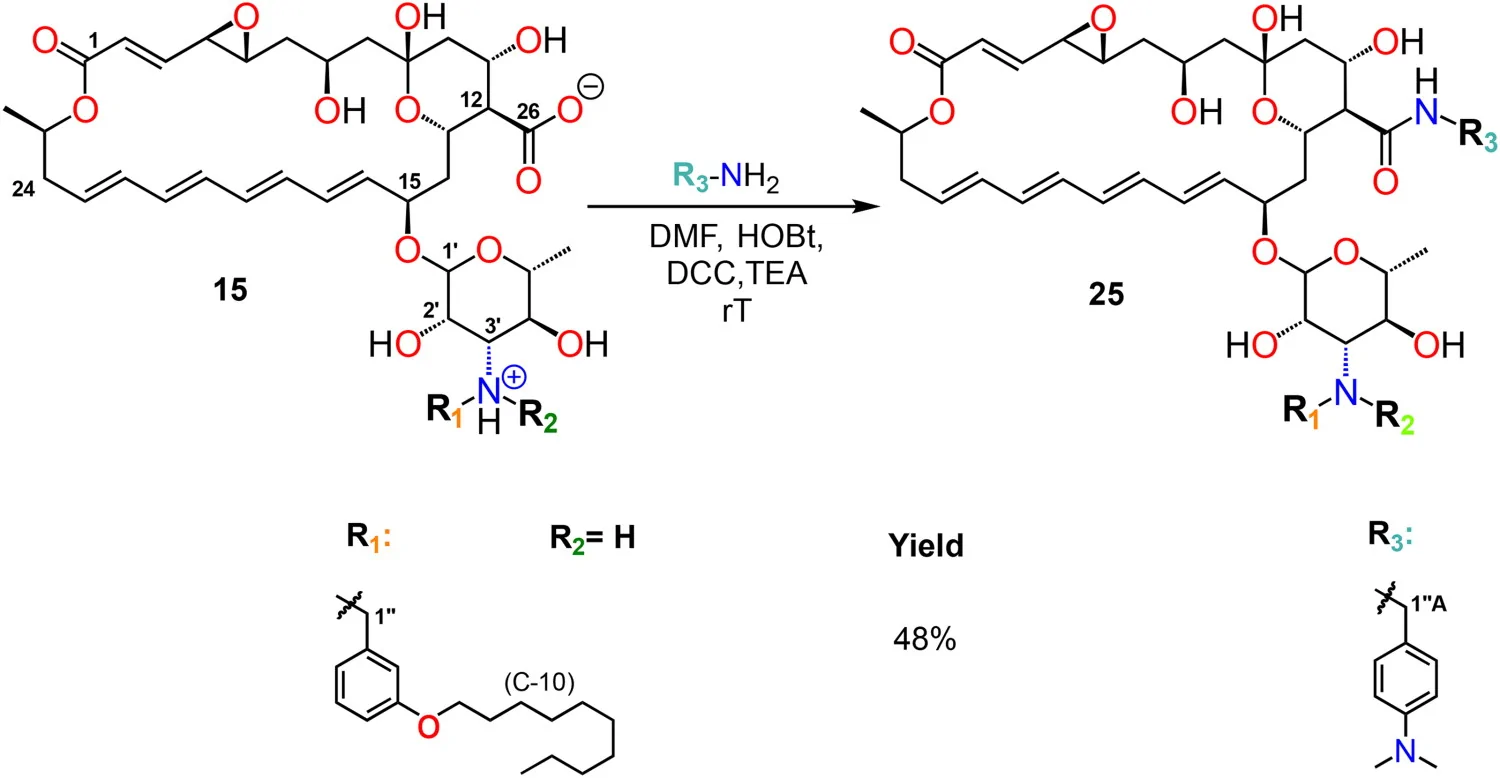
Synthesis of doubly modified amino-amide derivative of **PIM** (compound **25**) from derivative **15**.

A series of novel *N*-alkyl-amine derivatives of **PIM** (**1–24**) were synthesised *via* reductive amination with NaBH_3_CN as a mild reducing agent in the presence of TEA in MeOH/DCM solvent mixture (Figure 2). To evaluate the role of the C12 carboxylate group in biological activity an additional amidation reaction was performed affording the hybrid *N*-alkylamine/amide derivative **25** (Figure 3). The yield of these transformations was moderate. Benzyl derivatives were obtained in yields ranging from approximately ∼50 to 70% (with the exception of **5**), whereas aliphatic derivatives were obtained in lower yields from approximately 30 to 50%. Successful modification at **PIM** amino group was confirmed by analysing 1D and 2D NMR, FT-IR and ESI MS spectra, recorded for all 25 new derivatives (Supplementary data).

**Figure 4.**
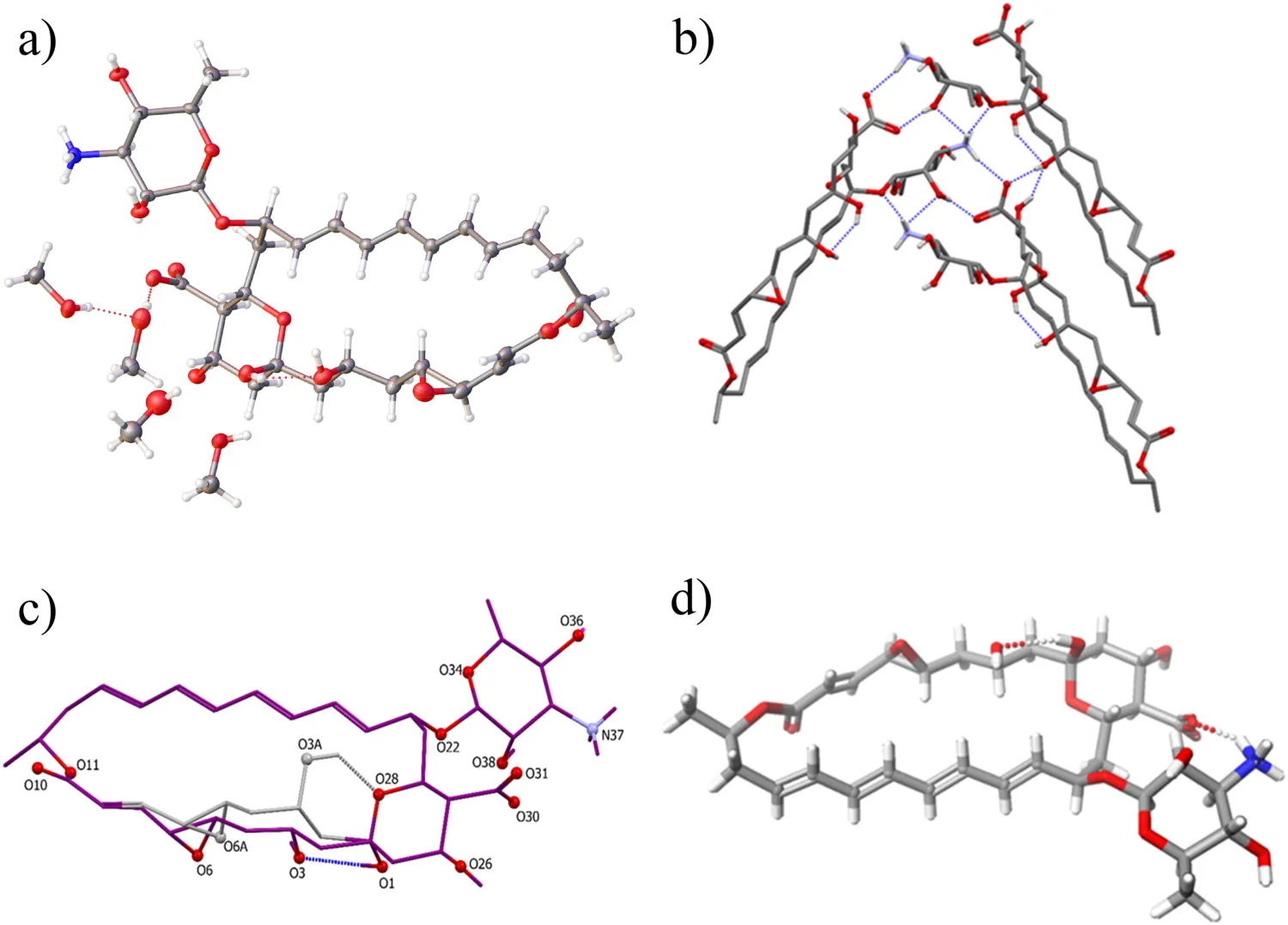
Structure of **PIM** (pimaricin/natamycin) determined by X-ray method in crystal: a) the zwitterionic form of the **PIM** molecule in the solvated crystal form PIM2 (Supplementary data); b) hydrogen bonds involving the polar head of **PIM** in the PIM2 solvated crystal; c) disorder in the crystal structure of PIM1 resulting from two distinct conformations. Atoms belonging to the fragment in the minor conformation are shown in gray, intramolecular H-bonds as dashed lines; and d) intramolecular H-bond interaction between protonated amino group and carboxylate noted in solution (on the basis of FT-IR, ^1^H, ^13^C and ^15^N NMR data).

Importantly, prior to modification, **PIM** existed exclusively as zwitterion, with the proton transferred from the carboxylic group to the amine nitrogen of mycosamine. This is evidenced by the structures of two **PIM** crystalline forms, PIM1 and PIM2 (Supplementary data- Figure S225, Figure S226), obtained from a protic MeOH-H_2_O solvent system (Figure 4(a–c)). Furthermore, in the PIM2 form, a portion of the macrocycle adopts two distinct conformations differing in the arrangement of the polyol fragment relative to the epoxide; specifically, the C(7) hydroxyl group is situated either on the same side of the macrocycle mean plane as the epoxide moiety, or on the opposite side (Figure 4(c)). Consequently, these two conformers of **PIM** form distinct intramolecular O–H···O hydrogen bonds (Supplementary data-Figure S227, Figure S228). There are no short intramolecular contacts between the carboxylate and protonated amine groups in the two crystalline forms of **PIM**, thereby ruling out the formation of an intramolecular N–H^+^···O^-^ hydrogen bond in solid. Instead, these two groups are involved in numerous strong intermolecular interactions, as shown in Figure 4(b).

However, in solution direct intramolecular interactions between the protonated amino group and the carboxylate within the zwitterionic form (Figure 4(d)), take place. This statement is supported by the presence of a broad and red shifted ν(N^+^-H) absorption (shoulder at ∼2800–3000 cm^−1^) in FT-IR spectrum of **PIM**, corresponding to N^+^-H^…^‾OOC- hydrogen bond realised for the zwitterion in solution (Supplementary data - Figure S224). Notably the ability of **PIM** and its derivatives to exist as zwitterions, similarly to amphotericin B and nystatin, has not been adequately considered in previous studies^23^^,^^24^^,^^26^, despite the importance of this structural feature for chemical reactivity, polarity, lipophilicity of the antibiotic and ultimately of the biological mechanism of these antibiotics.

**Figure 5.**
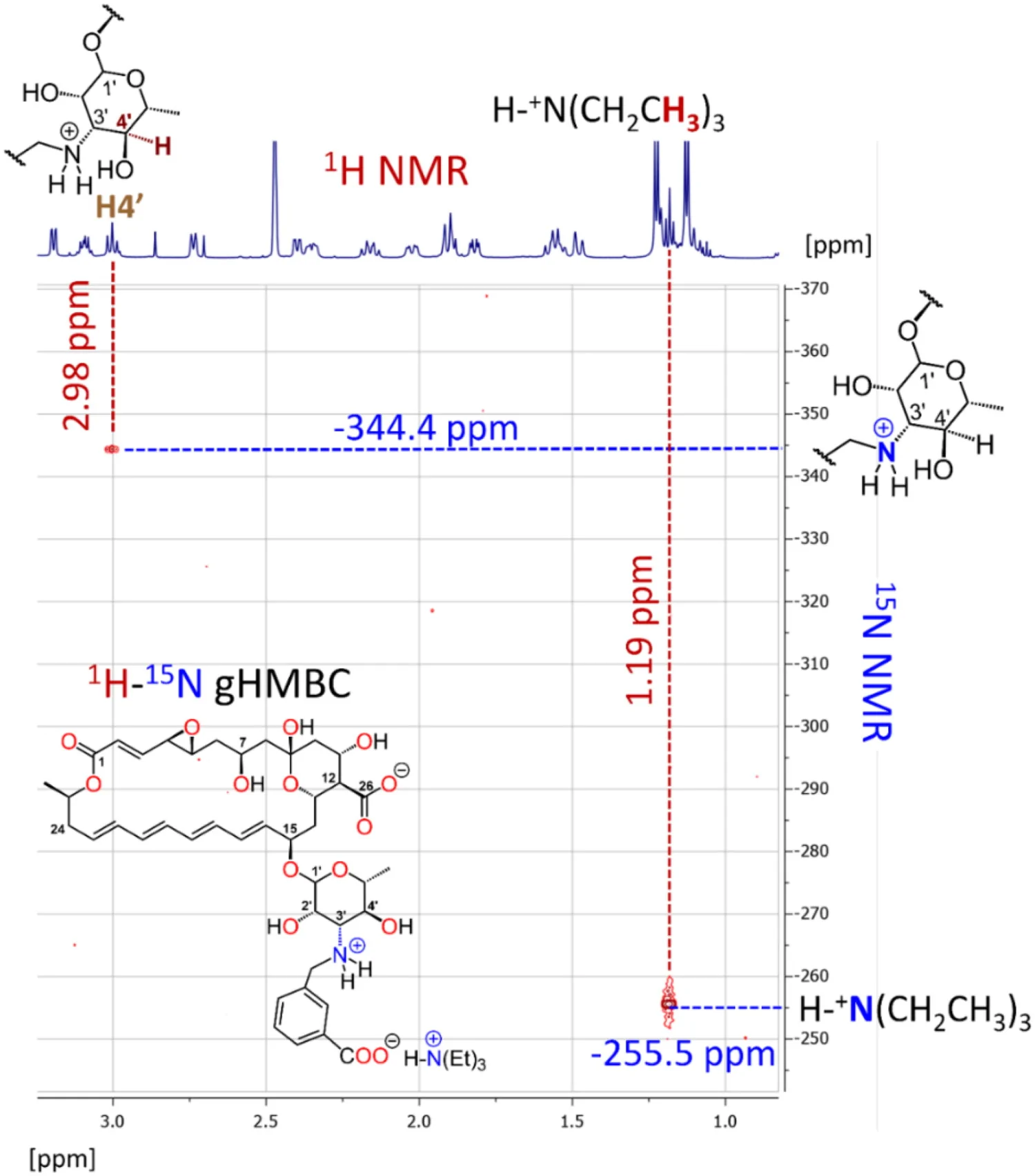
The ^1^H-^15^N gHMBC spectrum of **10** revealing its internal/external double salt structure.

**Figure 6.**
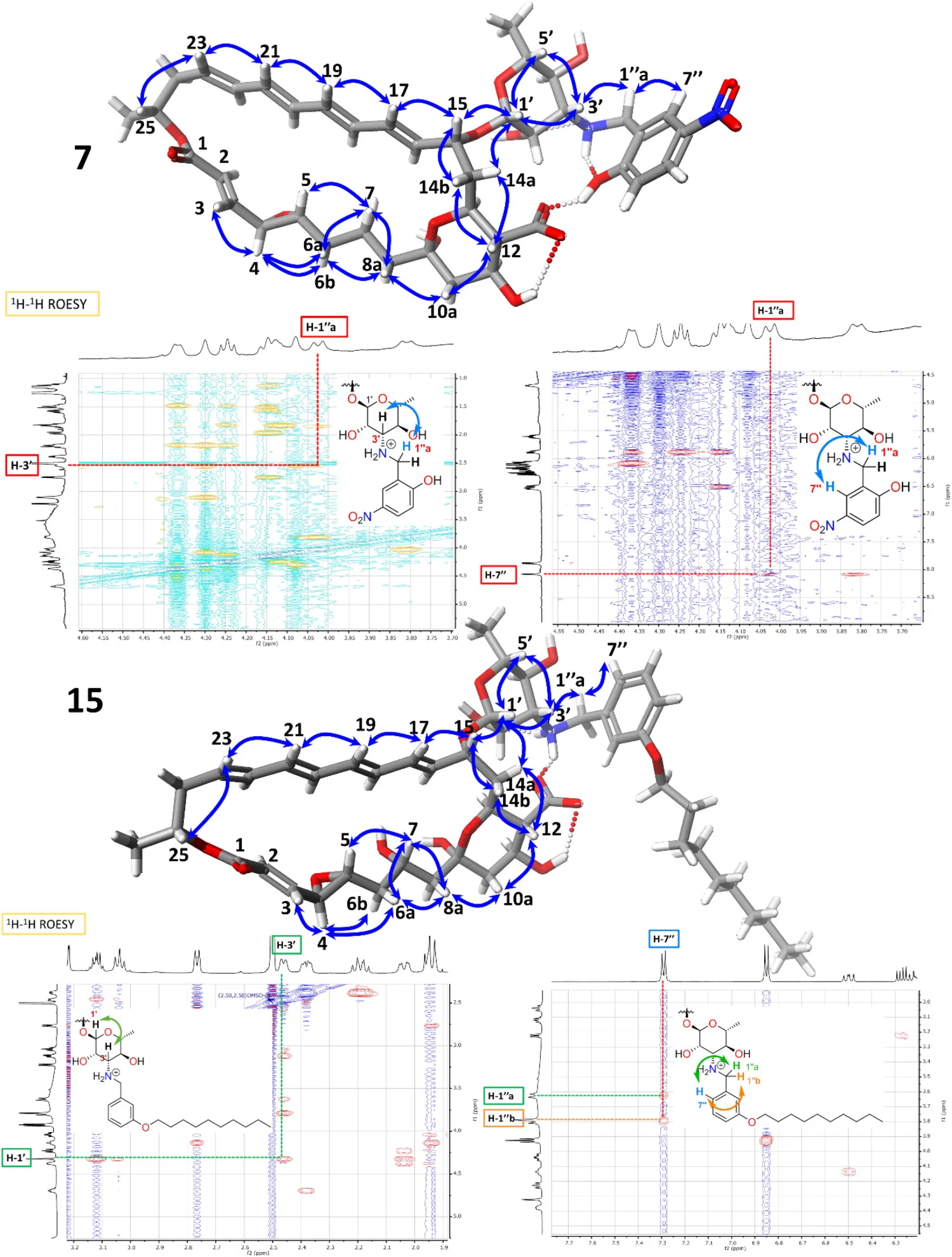
Computationally optimised structures of **PIM** derivatives containing functionalised benzyl substituent part (**7** and **15**), being in agreement with ^1^H-^1^H ROESY contacts, noted in the spectra recorded in dmso-d_6_ solutions [*Scigress* FJ. 2.6 EU 3.1.9, Fujitsu 2019–2026; method B88LYP (GGA)]^41^.

**Figure 7.**
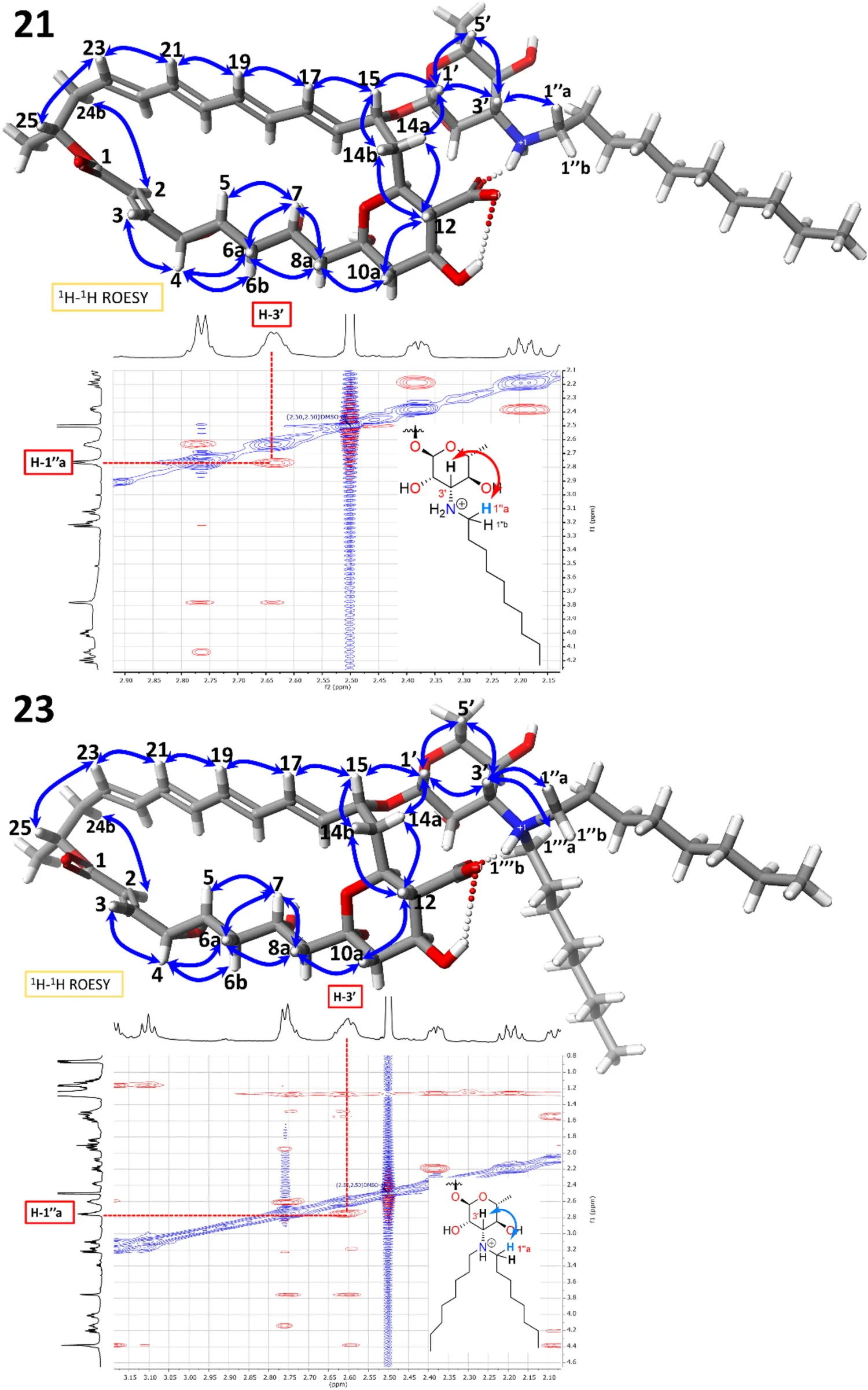
Computationally optimised structures of **PIM** derivatives containing mono- and di-alkyl moieties at the nitrogen as substituent parts (**21** and **23**), being in agreement with ^1^H-^1^H ROESY contacts, noted in the spectra recorded in dmso-d_6_ solutions [*Scigress* FJ. 2.6 EU 3.1.9, Fujitsu 2019–2026; method B88LYP (GGA)]^41^.

To elucidate the structures of the **PIM** derivatives extensive 1D and 2D NMR studies have been performed in dmso-d_6_, in analogous solvent used for antifungal and cytotoxicity assays. Assignments of ^1^H, ^13^C and ^15^N NMR were verified by correlation spectra: ^1^H-^1^H COSY, ^1^H-^1^H ROESY, ^1^H-^13^C gHSQC, ^1^H-^13^C gHMBC, ^1^H-^15^N gHSQC and ^1^H-^15^N gHMBC (Figures 5–7; Tables S1–S6; Supporting Data). The zwitterion structure of the **PIM** and its derivatives is evidenced by a comparison of *δ*_C26_ between **PIM** and derivatives **1–8** and **11–24** revealing C(26) carbon atom signals in a comparable narrow spectral range ∼173–175 ppm in solution (Supplementary data - Table S1, Table S2). This interpretation is further supported by stronger shielding of C(26) atom signal observed for double functionalised amide/*N*-alkyl derivative **25** (*δ*_C26_ = 171.4 ppm) in which the proton transfer is not possible, in contrast to the other derivatives. The conclusion concerning existence of **1–24** derivatives as zwitterions is further supported by their ^13^C NMR spectra in other region, where a consistently deshielding of carbon atom signals assigned to C(3′) (*δ*_C3′_=61.9–63.7 ppm) compared to non-zwitterionic amide derivative **25** (*δ*_C3′_ = 61.1 ppm), were noted. The deshielding of C(3′) carbon atom signal in the spectra of derivatives **1–24** is explainable by the vicinity of the positively charged nitrogen atom at the mycosamine in a result of the internal proton transfer and zwitterionization (Figure 4). It is well-established that aliphatic carboxylic acids are in general less acidic than aromatic carboxylic acids (*pK*_a_ of acetic acid ∼ 4.8 vs. *pK*_a_ of benzoic acid ∼ 4.2)^48^^,^^49^. Particularly noteworthy from the perspective of improving water solubility and antifungal activity was isolation of compounds **9** (after extraction) and **10** (without extraction) as internal (zwitterion) and internal/external (TEA containing) salts, respectively (Figure 2). These distinct salt forms were identified inter-alia by ^1^H-^15^N gHMBC NMR experiments. Compound **9** exhibited a single nitrogen atom resonance at *δ*_N_ = −344 ppm, correlated with H4’, whereas compound **10** displayed an additional signal at *δ*_N_ = −255 ppm, correlated with the methyl protons of protonated TEA (Figure 5; Figure S94, Figure S95). The signal assigned to the three exchangeable protons from amino group in the zwitterion of **PIM**, appeared as a single averaged resonance above 7 ppm, as confirmed by ^1^H-^15^N gHSQC NMR analysis (Figure S8). In contrast, most of zwitterionic **PIM** derivatives **1–22** exhibited two broadened signals at approximately ∼ 10 and 9.5 ppm assigned to two diastereotopic protons –NH_2_^+^- at the nitrogen of mycosamine, which could not be fully integrated due to the rapid chemical exchange (Figure S66A). A greater deshielding one of these -NH_2_^+^- proton signals in the spectra suggests a stronger H-bonding in zwitterion being in line with structural conclusions derived from NMR and theoretical studies (Figures 6 and 7). Collectively, X-ray, FT-IR and 1D and 2D NMR studies indicated that **PIM** and its *N*-derivatives are present solely as zwitterions with the proton of carboxyl group transferred to the nitrogen of mycosamine, both in solid or in solution. For derivative **9**, one of carboxylic acid protons is transferred to the nitrogen of the mycosamine and stabilised *via* H-bond (26) COO‾^…^H_2_N^+^(mycosamine) whereas the second acidic proton is transferred intermolecularly to TEA, forming an internal/external salt system (Figure S223). The structural distinction between compounds **9** and **10** is also reflected in approximately 10 ppm chemical shift difference in ^13^C NMR between signals of analogous C6’’ (compound **9**, *δ*_C6’’_ = 167.2 ppm) and C8’’ (compound **10**, *δ*_C8’’_ = 177.6 ppm), assigned to the carboxylic acid groups at the aromatic substituents. Elemental analysis further confirmed the presence of compound **10** as internal/external salt with TEA (Supplementary data - Experimental section). Thus, all studied derivatives of **PIM**, with exception of amide **25** and internal/external salt **10**, exist in solution as zwitterions, regardless of the nature of the introduced substituent at the nitrogen. From a mechanistic perspective the arrangement of a newly introduced substituent at the nitrogen regarding the saccharide and aglycone ring was important for **PIM** derivatives. The *δ*_H3′_ signal of the mycosamine portion for alkyl or aryl amine derivatives of **PIM** can be a spectral probe for spatial arrangement of the *N*-substituent (Figure 5). In ^1^H-NMR spectra of **1–24** characteristic proton signal H3′ appears in a relatively wide range 2.37–2.83 ppm, depending on substituent type at the nitrogen (Supplementary data-Tables S3–S6). It is worth to note that for typical *N*-aliphatic mono- and dialkyl-derivatives **20–23** the H(3′) proton signal appears in the range *δ*_H3′_=2.60–2.64 ppm whereas for *N*-benzyl-type derivatives (**1–17**) is a markedly shielded, i.e. *δ*_H3′_ = 2.37–2.54 ppm (Figure 5).

**Table 1.** Antifungal activity of **PIM** and its derivatives in comparison with **NYS** (nystatin A_1_) and **FLU** (fluconazole).

	MIC [µg/mL]				
	*Candida glabrata*	*Candida albicans*	*Aspergillus fumigatus*	*Trichophyton rubrum*	*Fusarium oxysporum*
**PIM**	4	4	16	16	16
**NYS**	2	4	16	16	16
**FLU**	64	64	64	16	64
**1**	32	64	64	64	64
**2**	64	32	–	–	–
**3**	64	64	16	64	32
**4**	32	32	32	32	32
**5**	16	32	32	32	32
**6**	64^a^	32^a^	– ^a^	– ^a^	– ^a^
**7**	4	4	8	8	16
**8**	64^a^	32^a^	– ^a^	– ^a^	– ^a^
**9**	64	64	16	64	32
**10**	32	64	32	16	32
**11**	16	32	32	32	16
**12**	64	64	64	64	64
**13**	32	32	32	64	64
**14**	16	32	32	64	32
**15**	4	2	16	64	64
**16**	64	64	64	64	64
**17**	16	16	32	64	64
**18**	64	32	64	64	64
**19**	8	8	8	32	16
**20**	16	8	16	32	32
**21**	16	16	8	16	16
**22**	32	32	32	64	64
**23**	16	32	64	64	64
**24**	32	32	32	32	64
**25**	> 64	> 64	> 64	> 64	> 64

^a^Compounds precipitated at MIC assignments and of limited solubility in medium during experiments.

Lower *δ*_H(3′)_ for benzyl-type **PIM** derivatives is an evidence of specific conformation where H3′ is placed in the ring current of the aryl moiety (shielding zone), as illustrated for derivatives **7** and **15** (Figure 6) and for **3**, **4** and **12** (Supplementary data-Figure S33, Figure S42, Figure S112). An exception is derivative **18**, bearing large anthracene substituent. In this case the aromatic substituent adopts different orientation relative to the mycosamine moiety (Figure S161) resulting in a pronounced deshielding of H3′ signal (*δ*_H3′_ = 2.83 ppm) as well as those assigned to H1’ and H2’ protons of the mycosamine (Supplementary data- Tables S3–S6). The mutual orientation of the functionalised *N*-benzyl substituent and mycosamine as well as general conformation of derivatives were evidenced using ^1^H-^1^H ROESY spectroscopy (Figure 6). The restricted conformation liability of benzyl moiety is dictated by polarised hydrogen bonding network -N^+^-H…(3′’)O-H…‾OOC(12), as observed for **^7^**. These interactions generate 14- or 12-membered pseudocyclic systems, respectively. This intramolecular H-bonded structure (Figures 6 and 7) is manifested by the presence of a broad absorption at ∼2800 − 3000 cm^−1^ in their FT-IR spectra in solution (Supplementary data-Figure S224). Interestingly, the lack of ^1^H-^1^H ROESY contacts between *N*-aliphatic substituents and mycosamine moiety, regardless of whether one or two alkyl chains are attached to the nitrogen, suggests that these aliphatic chains do not participate in any intramolecular hydrophobic interactions with either the mycosamine or aglycone portions of the molecule (Figure 7).

### Antifungal potency and physico-chemical parameters of PIM derivatives

All **PIM** derivatives **1–25** were evaluated for antifungal activity against: *C. glabrata*, *C. albicans*, *A. fumigatus*, *T. rubrum* and *F. oxysporum,* where pimaricin (**PIM**), nystatin (**NYS**) and fluconazole (**FLU**) were used as reference compounds (Table 1). All **PIM** derivatives exhibited antifungal properties ranging from very good to weak, with MIC values falling within the range of 2–64 µg/mL and above. It should be highlighted that the reference derivative **25**, containing carboxyl transformed into amide and *N*-benzyl-OC_10_ substituent installed at the nitrogen of mycosamine, revealed the lowest potency compared to those of derivatives **1–24**, in which only the mycosamine nitrogen atom was substituted. This finding is somewhat surprising in light of earlier studies by Tevyashowa et al who reported a high antifungal potency of amide **PIM** derivatives against *C. glabrata* and *C. albicans* (MICs = 1–4 µg/mL)^22^. The highest antifungal potency revealed derivative **15** against *Candida albicans* with MIC = 2 µg/mL. This result was the most favorable taking into regard even all used reference compounds (**NYS**, **FLU**) and **PIM**. However, the broadest range with together high antifungal activity level (MICs 4–16 µg/mL) exhibited other derivative – compound **7,** which contains a methylene-nitrophenol moiety. It is noteworthy that neither the presence of nitrobenzyl moiety (compound **6**) nor the methylene-bromobenzylphenol substituent (compound **8**) was beneficial to antifungal potency, in contrast to the methylene-nitrophenol moiety (compound **7**). Furthermore, the presence of *N*-benzyl-alkyl hybrid combination of substituents (compounds **13** and **14**), containing either a shorter alkyl chain (up to 3 C atoms) or the altered aryl substitution pattern (*o*- or *p*-), did not result in enhanced antifungal potency (MICs ≥16 µg/mL) when referred to **PIM**. Compound **7** showed comparable MIC values against two *Candida* and one *Fusarium* strains with **PIM**. Interestingly antifungal activity of **7** against *Aspergillus fumigatus* and *Trichophyton rubrum* strains was better not only than **PIM** but also than **NYS** and **FLU**. Similarly to **7**, compounds **19** and **21** exhibited higher antifungal potency than **PIM** and other reference compounds, although against one strain *A. fumigatus*. However, the highly active zwitterionic compounds **7**, **19** and **21** against *A. fumigatus* contain structural diverse *N*-substituents (**7** has benzyl with nitro and phenol; **19** possesses methylene cyclopropyl and **21** has one C_10_ aliphatic chain), so therefore the SAR here is not observed, similarly to activity results concerning tests against *Candida* sp. It can be also supported by result regarding compound **3,** containing *N*-benzylfluorine moiety, and showing activity also comparable to the reference polyenes against *A. fumigatus* at MICs =16 µg/mL. Furthermore, no clear relationship was observed between antifungal activity of either the length of the *N*-alkyl chain or steric bulk of the *N*-substituent. The introduction of a second alkyl chain at the nitrogen (compounds **23** and **24**) or double ionisation of the antibiotic scaffold (compound **10**) proved to be ineffective strategies for improving efficacy against *A. fumigatus*. In turn, compounds **7**, **19**, **11** and **21** showed the highest activity against *F. oxysporum* with MIC values of 16 µg/mL. These values were comparable for **PIM** and **NYS** and better than those of **FLU**.

According to the above discussion, tests against *Candida* sp. revealed that compounds **15** and **7** exhibited the highest activity or the broadest spectrum of activity among all investigated derivatives (Table 1). Interestingly, compounds **15** and **7** have markedly different physico-chemical profiles, the former shows almost perfect logP = 2.86 at limited water solubility (S_H2O_ = 40 mg/L, Table 2) whereas the latter is characterised by poor logP and better water solubility (S_H2O_ = 110 mg/L, Table 2). Overall, the introduction of *N*-benzyl-type substituents into **PIM** scaffold was more beneficent towards increased water solubility than the attachment of long *N*-alkyl substituents (Table 2). The poor biological result noted for double functionalised **25** (at carboxylate and nitrogen of mycosamine) can be explained by an unfavourable physico-chemical profile (logP = 0.31, S_H2O_ = 40 mg/L). Derivatives **19**, containing methylene-cyclopropyl, and **20**, containing pentyl substituent at the nitrogen of mycosamine showed antifungal activities approaching that of **PIM** (2-fold lower, MICs =16 µg/mL) against *Candida sp*. (Table 1). This result could be understandable since smaller *N*-substituents of **19** and **20** contributed to a slightly improved water solubility (S_H2O_ ≥100 mg/L, Table 2) regarding **PIM**. Likewise the presence of **PIM** derivatives in the form internal/external salt (compound **10**) or internal zwitterionic form (compound **9**), despite enhanced water solubility, did not improve antifungal activity against *Candida sp.* resulting in MIC values of 32–64 µg/mL. Moreover, high water solubility alone was not sufficient to enhance antifungal activity against *Candida* sp. as demonstrated by compounds **8** and **20** (Tables 1 and 2). Similarly, higher lipophilicity alone did not appear to be a crucial factor influencing potency against *A. fumigatus* since a relatively lipophilic compound **21** (logP = 1.95) revealed high and equal antifungal activity as **7** and **19** showing substantially lower logP values (∼ −0.8). Overall, compound **7** emerged as the most and broad-spectrum antifungal derivative exhibiting although moderate water solubility but ultimately higher than **PIM** (Table 2). It should be noted that higher potency of **7** against *A. fumigatus* and *T. rubrum* than **PIM** can be only partially explained by a slightly better water solubility but not solely by this factor, as demonstrated by our further discussed model of the complex between self-assembled **PIM** derivative and ergosterol (**ERG**) - one of the principal components of the fungal cell membranes.

**Table 2.** Physico-chemical parameters (lipophilicity logP, water solubility S_H2O_^exp^ [mg/L]) determined for pimarycin (**PIM)**, its derivatives **1–25** as well as reference compounds nystatin (**NYS**) and fluconazole (**FLU**).

	logP	S_H2O_^exp^ [mg/L]
**PIM**	−1.67	60
**NYS**	−0.20	340
**FLU**	0.88	110
**1**	0.14	140
**2**	0.02	70
**3**	0.28	100
**4**	0.09	110
**5**	0.87	<30
**6**	−0.59	120
**7**	−0.69	110
**8**	0.13	160
**9**	−0.41	130
**10**	−0.67	100
**11**	0.05	150
**12**	1.06	40
**13**	0.78	60
**14**	−1.00	70
**15**	2.86	40
**16**	−0.16	40
**17**	0.91	<30
**18**	1.53	130
**19**	−0.78	100
**20**	−0.14	160
**21**	1.95	<30
**22**	2.00	<30
**23**	2.51	<30
**24**	−0.56	75
**25**	0.31	40

#### Toxicity in healthy cells and unexpected high anticancer activity of PIM derivatives

All synthesised **PIM** derivatives were evaluated towards their toxicity in normal human dermal fibroblast cells (HDF, Table 3) and human umbilical vein endothelial cells (HUVEC, Table 3). Given the IC_50 (HDF)_ values collected in Table 3, is clear that nearly all selected and studied derivatives showed lower toxicity in normal fibroblast cell line than **PIM**, with exception of derivative **23** (IC_50_ **23**_(HDF)_ < 1.61 µM; Table 3). Results obtained in HDF are in line with test results in other normal cell line HUVEC, where studied derivatives (**3** and **7**) were also less toxic than **PIM** (Table 3). Particularly noteworthy are compounds **7** and **15** which combine broad-spectrum antifungal activity with markedly reduced toxicity towards HDF cells, compared with **PIM** (Table 3). Furthermore, especially limited toxicity of a broad–spectrum active antifungal derivative **7** is even lower than those of typical anticancer agents as e.g. **mitC** or **actD** in HDF cells (Table 3). In turn, transformation of **PIM** into zwitterionic **15** afforded the least toxic compound among all derivatives (IC_50 (HDF)_ = 4.11 µM; Table 3), even lower toxic than sister polyene **NYS** (IC_50 (HDF)_ = 3.96 µM; Table 3). In contrast, fluorobenzyl *N*-derivatives **3** and **4,** were the most toxic representatives among *N*-benzyl analogs in HDF cells, although their toxicity remained slightly lower if compared to **PIM**. The overall trend in toxicity is also in agreement when compared with test results of **PIM**, **3, 4, 7** and **23** in other normal cell line HUVEC (Table 3). It is worth to note that compounds **3** and **4** were relatively toxic in normal cell lines even despite their relatively good water solubility (S_H2O_ ≥100 mg/L, Table 2). The most toxic of all studied derivatives in HDF and HUVEC normal cells was zwitterionic **23**, containing two C_8_ alkyl chains at the nitrogen (Table 3). This derivative showed unfavourable higher toxicity not only regarding **PIM** but also **NYS** and typical anticancers as **mitC** and **actD**, in HDF cell line.

**Table 3.** Toxicity of **PIM** and its selected derivatives, **NYS**, **mitC** and **actD** evaluated in normal human dermal fibroblasts/HDF [µM]/, and human umbilical vein endothelial cells/HUVEC [µM]/and anticancer activity evaluated in prostate cancer/PC-3/and epithelial ovarian cancer/SKOV-3/cell lines [µM]; [SI]^HDF^; [SI]^HUVEC^ - selectivity indices are given in square brackets.

	IC_50_ [µM] /[SI _HDF_],[SI _HUVEC_]			
	PC-3	SKOV-3	HDF	HUVEC
**PIM**	0.69 ± 0.06 [2.33]^HDF^; [3.16]^HUVEC^	0.69 ± 0.03 [2.33]^HDF^, [3.16]^HUVEC^	1.61 ± 0.04	2.18 ± 0.02
**NYS**	2.11 ± 0.01 [1.87]^HDF^, [2.74]^HUVEC^	2.06 ± 0.01 [1.92]^HDF^, [2.80]^HUVEC^	3.96 ± 0.03	5.78 ± 0.14
**mitC**	0.58 ± 0.11 [2.38]^HDF^	0.61 ± 0.02 [2.26]^HDF^	1.38 ± 0.07	–
**actD**	1.17 ± 0.03 [2.40]^HDF^	1.14 ± 0.11 [2.47]^HDF^	2.81 ± 1.15	–
**3**	0.61 ± 0.03 [2.72]^HDF^, [4.23]^HUVEC^	0.61 ± 0.02 [2.72]^HDF^, [4.23]^HUVEC^	1.66 ± 0.59	2.58 ± 0.08
**4**	0.84 ± 0.01 [2.01]^HDF^, [2.63]^HUVEC^	0.83 ± 0.01 [2.04]^HDF^, [2.66]^HUVEC^	1.69 ± 0.06	2.21 ± 0.06
**7**	1.74 ± 0.02 [1.82]^HDF^, [1.71]^HUVEC^	1.72 ± 0.02 [1.84]^HDF^, [1.73]^HUVEC^	3.16 ± 0.07	2.98 ± 0.06
**15**	2.81 ± 0.01 [1.46] ^HDF^	2.85 ± 0.01 [1.44]^HDF^	4.11 ± 0.09	–
**18**	1.84 ± 0.03 [1.50]^HDF^	1.86 ± 0.02 [1.49]^HDF^	2.77 ± 0.16	–
**19**	1.03 ± 0.02 [1.79]^HDF^	0.99 ± 0.03 [1.87]^HDF^	1.85 ± 0.04	–
**21**	1.21 ± 0.03 [1.59]^HDF^	1.08 ± 0.01 [1.78]^HDF^	1.93 ± 0.04	–
**22**	1.22 ± 0.01 [1.90]^HDF^	1.08 ± 0.04 [2.16]^HDF^	2.33 ± 0.03	–
**23**	0.68 ± 0.02 [1.53]^HDF^, [3.16]^HUVEC^	0.66 ± 0.02 [1.57]^HDF^, [3.26]^HUVEC^	1.04 ± 0.12	2.15 ± 0.04

*Notes:* **NYS**: nystatin**; mitC:** mitomycin; **actD:** actinomycin D; selectivity indices [**SI _HDF_**] was calculated as ratio of IC_50_ in the normal cell HDF to IC_50_ in the cancer cell line. Selectivity indices [**SI _HUAVEC_**] was calculated as ratio of IC_50_ in the normal cell HUVEC to IC_50_ in the cancer cell line.

To date investigations of the anticancer properties of **PIM** have been limited to prostate, gastric and hepatocellular carcinoma models^50–52^. Taking the above into consideration, the pronounced and interesting antiproliferative activities have been noted for our **PIM** derivatives in tested cancer cell lines PC-3 and SKOV-3 (Table 3). **PIM** itself revealed potent anticancer activity in both studied cancer cell lines, comparable to that of **mitC** or even higher regarding **actD**. However, this attractive anticancer activity was accompanied by relatively high toxicity in normal cell lines HDF and HUVEC, compared to **mitC** and **actD**. In contrast, sister polyene **NYS** showed limited anticancer potency in both PC-3 and SKOV-3 cells at reduced toxicity in HDF and HUVEC cells, when referred to **PIM (**Table 3). Compound **23**, bearing *N*-alkyl chains C_8_, was as anticancer active as **PIM** (IC_50_ ∼ 0.67 µM), although highly toxic in a healthy HDF and HUVEC cell lines (IC_50_/_HDF/_∼ 1 µM; IC_50_/_HUVEC/_∼ 2 µM; Table 3). More promising results were obtained for fluorobenzyl derivative **3**, which displayed slightly improved anticancer effects in SKOV-3 and PC-3 cells (IC_50_ ∼ 0.6 µM, Table 3) at lower toxicity in HDF and HUVEC, in comparison with **PIM** and **23**. Importantly, compound **3** showed higher anticancer potency not only than **PIM** but also **NYS** and **actD** at lower anticancer effects than **mitC** (Table 3), while remaining lower toxic in HDF cells than **mitC**. This favourable balance between toxicity and anticancer effects is reflected by the highest selectivity indices (SI_HDF_ = 2.72; SI_HUVEC_ = 4.23) among all studied **PIM** derivatives and reference compounds **PIM**, **NYS**, **mitC** and **actD** (Table 3). Shifting the fluorine in the aryl ring from *ortho*- to *meta*-position (compound **4**) resulted in only modest reduction of anticancer effects (IC_50_ ∼ 0.8 µM, Table 3), at comparable toxicity to that of compound **3** in HDF. Interestingly, both fluorobenzyl derivatives **3** and **4**, displayed only weak antifungal effects (Table 1), suggesting that structural features responsible for anticancer potency differ from those governing antifungal effects for this-type polyene derivatives. This observation is well reflected in antifungal potent compounds **7** and **15**, exhibiting only moderate anticancer effects (averaged IC_50_ ∼1–2 µM, Table 3) at the lowest toxicity in HDF normal cells of all studied compounds (IC_50 (_**_7_**_)_ HDF= 3.16 µM and IC_50 (_**_7_**_)_ HUVEC = 2.98; IC_50 (_**_15_**_)_ HDF= 4.11 µM, Table 3). Compound **19**, containing methylene-cyclopropyl substituent, seems to be compromise between antifungal (Table 1) and anticancer (Table 3) properties but at toxicity in normal cells higher than **7** and **15**. Other, not described above **PIM** derivatives showed moderate anticancer effects with IC_50_ values >1 µM.

Overall, compound **3** represents the most promising anticancer candidate, combining enhanced antiproliferative activity in SKOV-3 and PC-3 cancer cell lines with a slightly reduced toxicity in HDF and HUVEC at the highest selectivity indices SI ∼ 3–4. The molecular mechanism underlying the anticancer activity of **PIM** derivatives remains to be elucidated. Previous studies suggested that **PIM** induces anticancer effects by involving oxidative stress and blocking of molecular chaperone function^50–52^. These mechanisms may likewise contribute to the anticancer activity of the most promising derivatives **3** and **4**.

### Explanation of antifungal effects of PIM derivatives based on association processes with ERG in fungi membranes and physico-chemical parameters (logP and S_H2O_)

Although the precise molecular mechanism underlying the antifungal activity of **PIM** has not been fully disclosed up to now, ergosterol (**ERG**) is generally considered its primary membrane-associated target. To gain insight into the superior and broad-spectrum antifungal activities of compounds **7** and **15**, their interactions with **ERG** were investigated by ITC (Isothermal titration calorimetry). ITC measurements demonstrated spontaneous association between all investigated derivatives and **ERG**, as indicated by negative Gibbs free energy values (Δ*G* < 0; Table 4). The driving force of association between **PIM**, **15**, **19** or **23** and **ERG** is both exothermic enthalpy Δ*H* < 0 and positive entropy of binding Δ*S* > 0. In contrast, association of **ERG** with compounds **7** or **17** was characterised by unfavourable endothermic enthalpy changes (Δ*H* > 0) compensated by substantial positive entropic contributions (Δ*S* > 0), indicating an entropy-driven binding process. The averaged binding constants between **ERG** and **PIM** derivatives are comparable and high (logK > 3, Table 4) indicating relatively effective formation of complexes in solution. Thus, despite variable K_D_ values (K_D_ = 0.7–92 µM), expressed per one molecule of **PIM** or its derivative, a much more important information was the number of derivative molecules bound to **ERG** (n-parameter**;** Table 4) from the molecular mechanism perspective. Both ITC curves for **ERG** binding with **PIM** and **7** have distinctive sigmoidal-shape with corresponding inflection points additionally confirming the calculated stoichiometric parameters for these systems (Supplementary data). As outlined above in the hypothesis regarding the biological mechanism by which **PIM** binds to **ERG**, this polyene specifically blocks vacuolar fusion in fungi, without disturbing integrity of the membrane^31^. Taking into account the number of **PIM** molecules per **ERG** in the complex formed (2:1) this mechanism seems to be plausible and experimentally confirmed. Such a low aggregation number is little to support formation of membrane-anchored self-associated complexes between **ERG** and **PIM**, as was in contrast evidenced for **NYS** or other polyenes by ITC thermograms^9^^,^^30^ or AFM images^27^. Previous biophysical studies indicate that at least six to eight molecules of **NYS** or other similar-size polyene are required to form stable pores/channels disturbing integrity of membranes or DOPC-**ERG** model systems. Derivatives **12**, **15**, **17**, **21** and **23**, similarly as **PIM**, form complexes with **ERG** at most of stoichiometry 3:1 (n-parameter up to 3, Table 4). Hence, these **PIM** derivatives act via molecular mechanism depending on the impairment of **ERG** function by blocking the vacuolar fusion or inhibition of other ergosterol-dependent cellular processes but not through pore formation in fungi membranes. Taking into consideration lower K_D_ values of derivatives **12**, **15**, **17**, **21** and **23** than that of **7** the former ones can act also alternatively as agents efficiently extracting **ERG** from the fungi membrane, yielding sponges at polar heads of phospholipids^29^. The less favourable K_D_ and *n* < 3 of *N*-benzyl/amide derivative **25** (Table 4) as well as its unfavourable physico-chemical properties (Table 2) seem to explain a lack of interesting antifungal activity. The broad-spectrum antifungal derivative **7** displayed a markedly different behaviour forming complexes with **ERG** of higher stoichiometry, equal 6:1 (*n* = 6; Table 4).

**Table 4.** Averaged dissociation constants $\;K_{d}$ per molecule, logK, standard enthalpy changes ΔH , entropy changes ΔS and Gibbs free energy ΔG for **ERG** associated with **PIM** and its derivatives (**7**, **12**, **15**, **17**, **19**, **21**, **23, 25**) at 25 °C in dmso_aq_; where n = number of **PIM** or **PIM** derivative molecules binding per one molecule of **ERG**.

Cmpd	*n*	K_D_ /per molecule/[μM]	logK	ΔH [kcal mol^−1^]	ΔG [kcal mol^−1^]	ΔS [kcal mol^−1^ K^−1^]
**PIM**	1.8 ± 0.1	0.7 ± 0.4	6.13 ± 0.24	–2.4 ± 0.3	–8.4 ± 0.3	20 ± 2
**7**	6.2 ± 0.2	32.1 ± 7.6	4.49 ± 0.11	5.3 ± 0.4	–6.1 ± 0.2	38 ± 2
**12**	1.5 ± 1.0	–	–	–0.2 ± 0.1	–	–
**15**	2.7 ± 1.7	28 ± 16	4.56 ± 0.29	–0.5 ± 0.1	–6.2 ± 0.4	19 ± 2
**17**	2.4 ± 0.8	28 ± 18	4.55 ± 0.33	0.6 ± 0.3	–6.2 ± 0.4	23 ± 2
**19**	4.6 ± 0.4	2.0 ± 1.5	5.70 ± 0.41	–0.2 ± 0.1	–7.8 ± 0.4	25 ± 2
**21**	2.0 ± 0.5	–	–	0.3 ± 0.1	–	–
**23**	0.7 ± 0.2	0.8 ± 0.3	6.12 ± 0.21	–2.3 ± 0.6	–8.4 ± 0.3	20 ± 4
**25**	2.6 ± 1.3	92 ± 37	4.04 ± 0.18	–4.6 ± 2.7	–5.5 ± 0.2	3 ± 10

A similarly elevated aggregation number was also observed for derivative **19** (*n* ∼ 5, Table 4), which also displayed good antifungal properties against a different fungi strains (Table 1). Such an increased stoichiometry, as observed for **7** and **19** at binding with **ERG**, is consistent with formation of supramolecular complexes resembling those proposed for membrane-active polyene antibiotics of greater aglycone rings (e.g. **NYS** or amphotericin B). Interestingly, compounds **7** and **19**, apart from sharing of a broader range of antifungal potency, also show similar physico-chemical properties (logP ∼ −0.7; S_H2O_ ∼ 100 mg/L; Table 2). In turn, the most cytotoxic and one of the less antifungal active derivative **23** revealed association with **ERG** close to 1:1 stoichiometry and K_D_ comparable with that of **PIM** (Table 4). This observation suggests that binding affinity alone (K_D_/one molecule, Table 4) is insufficient to explain antifungal activity and that the supramolecular self-organisation between the polyene derivative and **ERG** may play a critical role in the molecular mechanism of action.

To further investigate the molecular basis of **ERG** recognition by **PIM** derivatives ^1^H and ^13^C NMR spectra of a 6:1 mixture (**7**:**ERG**) were recorded. Comparison of ^1^H NMR data of **7** and **ERG** taken separately to the NMR data of 6:1 complex (**7**:**ERG**) revealed deshielding of diastereotopic proton signals, assigned to -NH_2_^+^- in the zwitterionic form, from 9.2 and 9.9 ppm to 10.03 and 10.73 ppm (Supplementary data-Figure S221). Furthermore, in the ^1^H NMR spectrum of the complex, between signals assigned to diastereotopic -NH_2_^+^- protons, a new broadened resonance appeared at ∼10.5 ppm. This signal was assigned to proton from the H-bonded phenol group belonging to the “polar head” area of molecule **7**. Additional deshielding of signal assigned to carbonyl carbon C(26) in the 13C NMR spectrum of the mixture, from 172.6 ppm for **7** to 175.5 ppm for 6:1 (**7**:**ERG**), further supports involvement of the “polar head area” of compound **7** in H-bond stabilisation. At the same time proton H(3) and C(1) carbon atom signals of the lactone region, remain at the same chemical shifts before and after 6:1 (**7**:**ERG**) complex formation (*δ*_H3_ = 6.26 ppm; *δ*_C1_ = 164.3 ppm, Supplementary data – Figure S222). This result indicates that the lactone moiety is not directly involved in specific interactions with **ERG** at association in solution.

**Figure 8.**
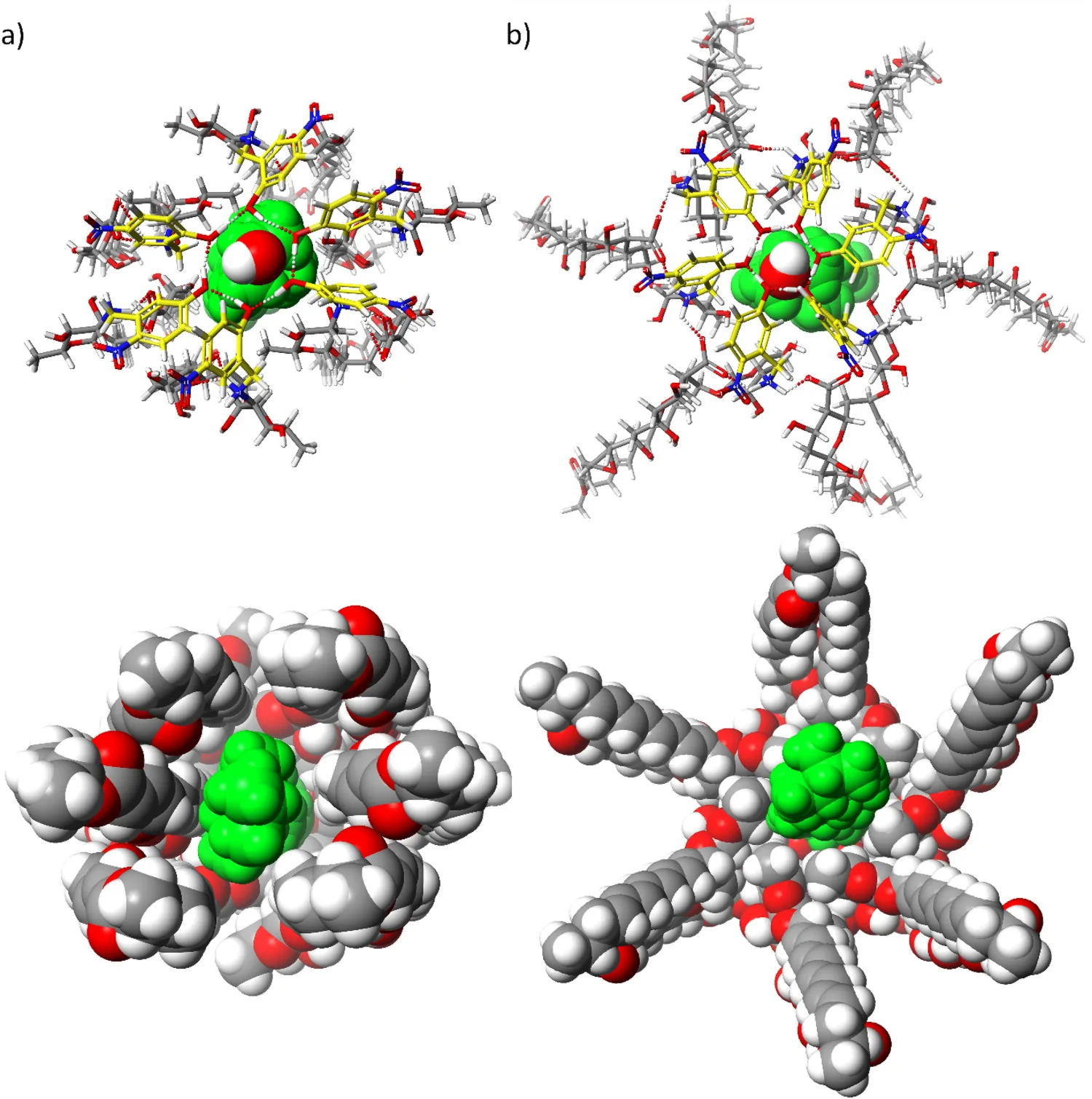
Models of two possible alternative structures of 6:1 complexes between hexamer of derivative **7** (grey; nitrophenol moieties are highlighted in yellow) and **ERG** (green) formed in dmso solution, where driving force of self-assembly process is strong collective intermolecular H-bonding of polar head phenol groups of molecules of **7**, whereby association of hydroxyl group of **ERG** and phenol groups is also visible: a) strong hydrophobic interactions realised along nearly whole backbones of **ERG** and **7** (long-distance close hydrophobic fit, ΔE= −127 kcal/mol); b) hydrophobic stabilisation between **ERG** and **7** is realised predominantly by unpolar face of mycosamine containing methyl group (fitting by a short hydrophobic contact, ΔE = −92 kcal/mol); optimised by MO-G PM6 method^41^.

To obtain further insight into the contribution of **ERG**-mediated self-assembly of **7** to antifungal activity, molecular calculation studies were performed, on the basis of: experimentally determined 6:1 stoichiometry (ITC), chemical shift changes noted in NMR, and earlier suggestions concerning formation of **ERG**-clusters in membranes with participation of polyenes of a greater aglycone ring^30^^,^^53^^,^^54^ (Figure 8(a,b)). The molecular calculations of the 6:1 complex (**7**: **ERG**) revealed two-type possible structures in solution, differing primarily by tighter (Figure 8(a)) or looser (Figure 8(b)) contact of **ERG** with the polyene moiety of derivative molecules. In both models the “driving force” of association is cooperative H-bonding between the phenolic groups of six zwitterionic molecules of **7,** analogously to calixarene-like structures. The existence of such cooperative H-bond network is supported by a broadened and downfielded proton signals of phenol groups (∼10.5 ppm) in the ^1^H NMR of the complex and by extended continuous absorption observed in FT-IR spectrum of the complex (Figure S224), both recorded in dmso-d_6_ and dmso_aq_, respectively. Among the two proposed models of 6:1 complexes (**7**:**ERG**), the structure shown in Figure 8(b) appear to be a more consistent with the spectroscopic data, despite less favourable energetic profit. In the structure in Figure 8(b), methyl groups of mycosamine of **7**, provide hydrophobic stabilisation with **ERG** backbone whereby the lactone groups of six molecules of **7** are exposed to solvation. This model is in line with the lack of any spectroscopic changes (^1^H and ^3^C NMR) assigned to the lactone region upon complexation, compared to **7**.

**Figure 9.**
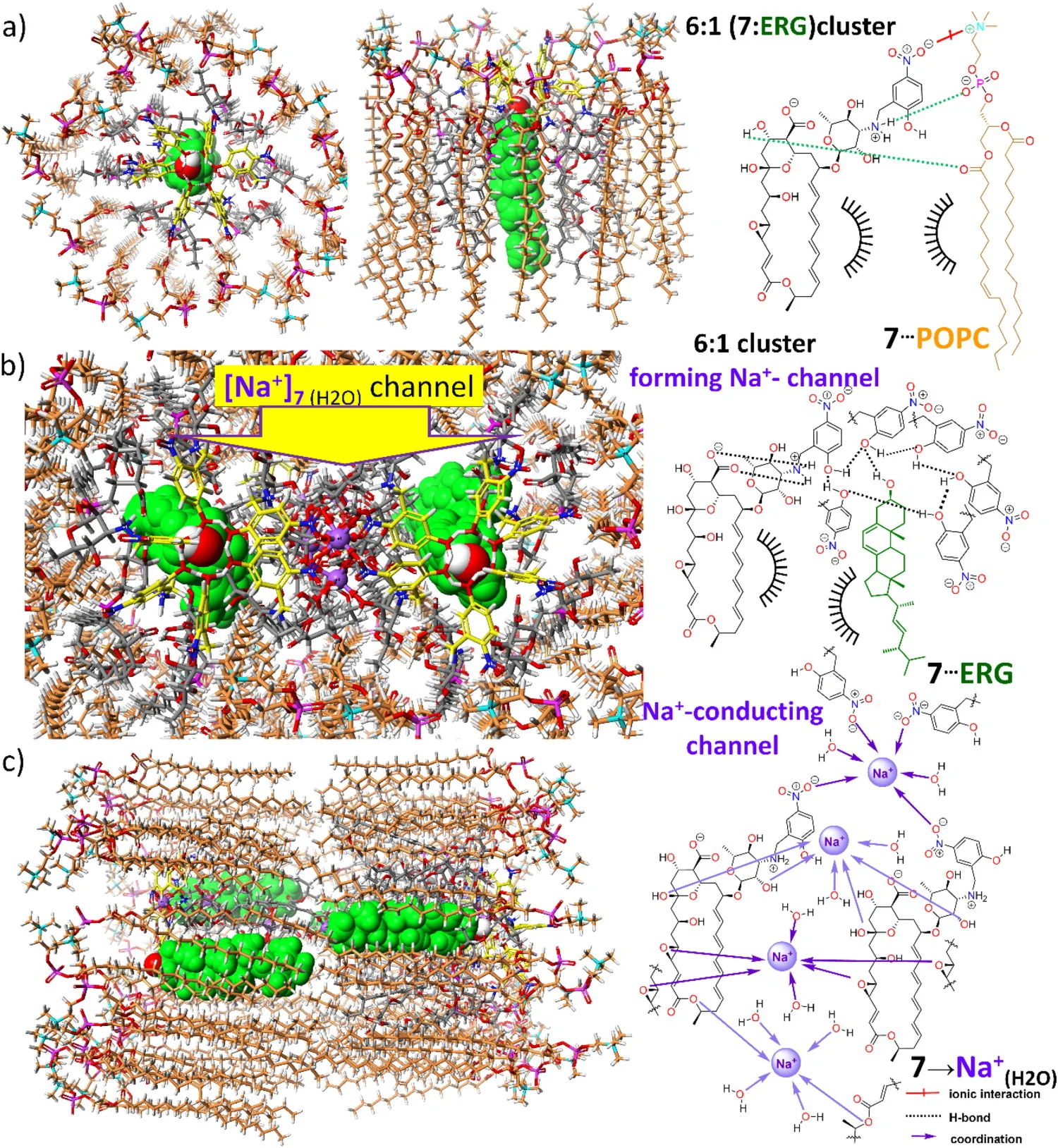
Molecular interactions between of the broadest-spectrum antifungal derivative **7** and **ERG** in phospholipid (POPC) matrix simulated fungi membrane: (a) model of interactions in 6:1 cluster (**7**:**ERG**) surrounded by POPC matrix (ΔE per one ERG molecule = −297.21 kcal/mol); (b) the view from the entrance of the Na^+^ channel (violet balls) formed between neighbouring two 6:1 complexes (**7**:**ERG**), the channel is filled with water molecules and seven Na^+^ metal cations are coordinated in octahedral spheres with participation of polyol face and nitrophenol moieties of **7** as well as water molecules (ΔE per one ERG molecule = −342.94 kcal/mol, left); the interactions between **ERG** and six molecules of **7** realised in the cluster self-assembled into channel structure (right); (c) side view on the Na^+^_(H2O)_-conducting channel revealing the mutual location of **ERG** molecules in the phospholipid membrane; calculated by MO-G PM6 method - *Scigress F.J. 2.6, EU 3.1.9* package^41^.

**Figure 10.**
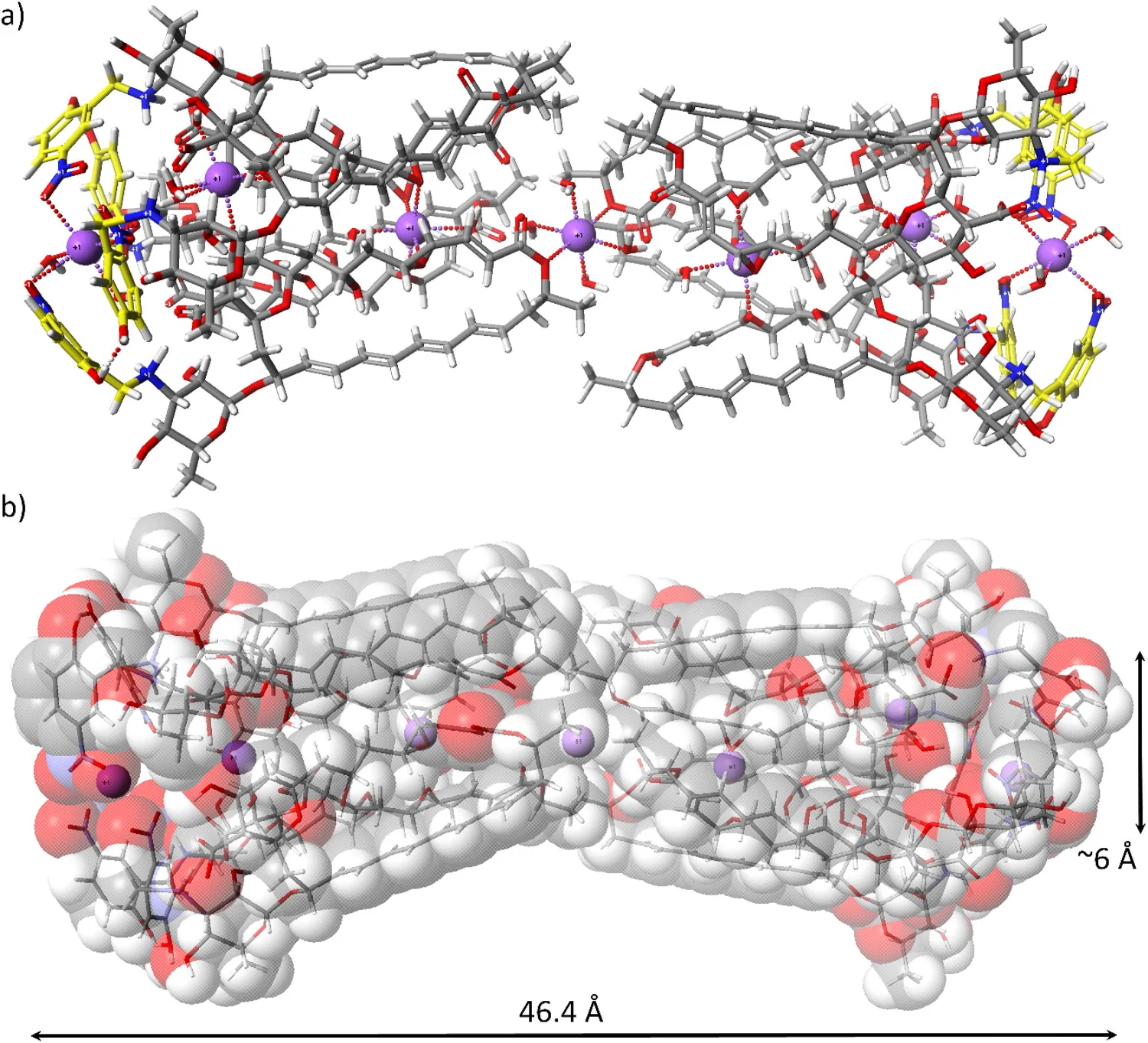
Optimised model of Na^+^_(H2O)_-conducting channel, formed between **7** molecules belonging to different 6:1 (7:ERG) clustersand nearby anchoring site of **ERG** (extracted structure from that shown in Figure 9(b)). The Na^+^-channel is filled with water molecules and seven Na^+^ cations (violet balls); 5-nitrobenzyl-2-phenol moieties of derivative **7**, taking part in active attraction of Na^+^ cations at the entrance of the tunnel, are marked by yellow; calculated by MOG-PM6 - *Scigress F.J. 2.6, EU 3.1.9* package^41^.

Molecular modelling of the 6:1 complex (**7**:**ERG**) incorporated into POPC (1-palmitoyl-2-oleoylphosphatidylcholine) model membrane revealed random hydrogen-bonding interactions between the ester carbonyl groups of phospholipid molecules surrounding the complex and the hydroxyls belonging to the six molecules of **7** (Figure 9(a)). In Figure 9(a) is also well-visible that the polyene portion of **7** has the hydrophobic contacts with the aliphatic chains of POPC and that the negatively charged oxygens of -NO_2_ groups within the **7** can be involved in electrostatic stabilisation with the positively charged nitrogen of choline portion within POPC. Thus, several interaction zones between polar head groups and membrane phospholipids can be distinguished. Molecular calculations indicate that despite derivative **7** possesses a shorter macrolide aglycone than **NYS** or amphotericin B, is capable of forming highly polar channels between self-assembled 6:1 complexes with **ERG** in fungal membranes (Figure 9(b,c)). These channels are predicted to permit the passage of small polar molecules and hydrated ions, thereby contributing to membrane dysfunction. The conformation flexibility of the polyol portion C_4_-C_8_, evidenced in this work by two alternative conformations of **PIM** aglycone in crystal (Figure 4(c)), is one of structural features enabling the assembly of the channel-forming 6:1 supramolecular clusters (Figure 9(b)). Furthermore, due to the above-mentioned conformational freedom, the polar polyol faces of **7** can be directed towards each other in phospholipid matrix, thus creating a polar areas outside the clusters. The resulting channel-like structure is lined by hydroxyl groups, amino groups and the nitro-substituents of **7** and is predicted to contain hydrated Na^+^ cations and water molecules (Figure 10). The estimated diameter of such channel equal ∼6 Å and the length ∼46 Å are consistent with dimensions required for transport of small ions and polar molecules. Notable the simulated distance between two **ERG** molecules trapped in clusters within the POPC membrane equal 15.9 Å agrees well with values ∼8–16 Å reported from isotope-probe and multinuclear solid-state NMR experiments, AFM imaging or neutron and X-ray scattering^55–57^ .The postulated structure of ion-conducting channel by us seems to be in agreement with the averaged natural ratio of **ERG** relative to phospholipids in fungi cell membranes (7:3 phospholipids: **ERG**^58^^,^^59^)

**Figure 11.**
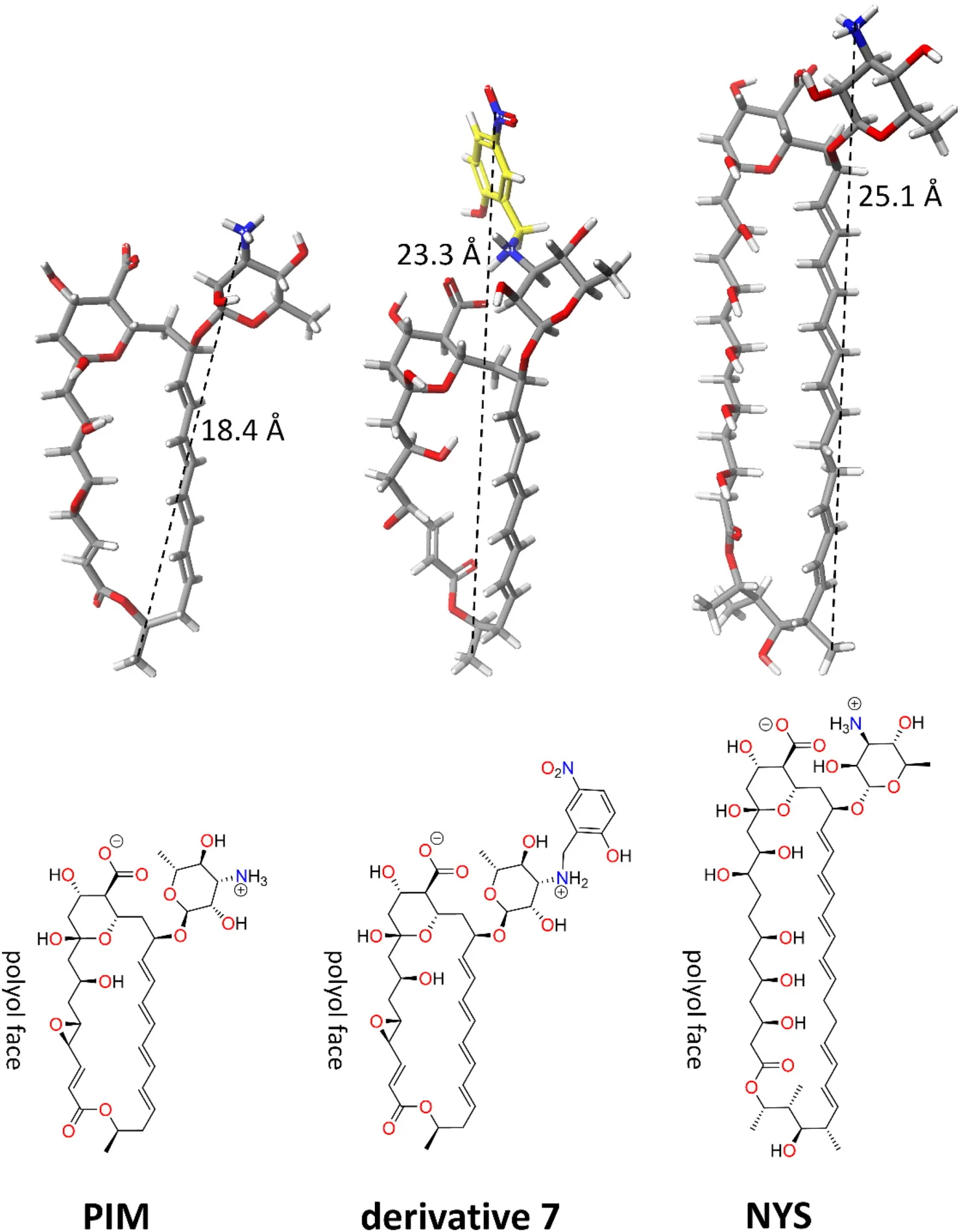
Comparison of molecule sizes between **PIM**, antifungal active **7** (extracted conformation from the calculated Na^+^-channel) and **NYS,** illustrated on interatomic distances between methyl near lactone and the most distant positively charged nitrogen atom, revealing similar molecule size (length) of derivative **7** and **NYS**.

Our calculations revealed also that some of nitro- groups within **7**, forming clusters with **ERG**, are important in attraction of metal cations as e.g. Na^+^ since these groups are oriented towards inside of the channel by collective H-bonding of phenols in the “crown” of the 6:1 clusters (Figures 8, 9(b) and 10). This observation explains why compound **6**, despite containing the nitro- group at introduced *N*-benzyl moiety, lacking the phenol, fail to reproduce the broad-spectrum antifungal activity of compound **7**. The H-bond association of phenols in hexamer of **7** orients the nitro groups towards the outside of the cluster but inside of the channel, so both of these groups are necessary at the *para* substitution pattern, to form such stable pores/channels in the fungi membrane. Furthermore, the formation of such channel-type structures between 6:1 clusters (**7**:**ERG**) reduces unfavourable contacts between hydroxyls of polyol portion and non-polar portions of POPC (long alkyl chains) since forces hiding of polar polyol moieties towards the inside of the ion-channel in order to coordinate Na^+^ ions. This assumption is in line with differences between calculated energetic profits per one **ERG** molecule in a single 6:1 cluster incorporated into POPC lipid bilayer (Figure 9(a); ΔE per one **ERG** molecule = −297.21 kcal/mol)

and in the cluster forming ion-conducting channel in POPC bilayer matrix (Figure 9(b); ΔE per one **ERG** molecule = −342.94 kcal/mol). At the analogous *para*-substitution pattern of phenol moiety, bromine atom (compound **8**) instead NO_2_- group (compound **7**), antifungal potency is weaker (Table 1) since probably bromine atom is weaker ligand at coordination of the metal cation inside the channel. In turn, compound **19**, despite ability to **ERG**-driven association into ∼ 5:1 complex (**19**:**ERG**) (Table 4) and slightly decreased water solubility, does not offer possibility of as good stabilisation of the polar heads in the analogous cluster/channel structures as those formed by **7**, so lower antifungal effects of **19** than **7** are understandable. Comparison of molecular sizes made on Figure 11, reveals that derivative **7** is only a slightly shorter than **NYS** by ∼ 2 Å at analogous tendency to association with **ERG** (6:1) and a more chemically compatible structure for interactions with fungi membrane phospholipids than **PIM** (see Figure 9(a)) - greater polarity of the polar head (protonated amine/carboxylate/nitro group vs. protonated amine/carboxylate). This key structural aspect indicates that postulated analogous mechanisms for **NYS** and **PIM** are likely.

Taken together, the combined ITC, NMR, FT-IR and molecular modelling results suggest that exceptional broad antifungal activity of compound **7** originates not simply from high affinity towards **ERG** but from its unique ability to form high-ordered **ERG**-associated supramolecular clusters, next self-organized into the structure of ion-channels in the phospholipid matrix. The favourable tendency to self-assembly with **ERG**, improved water solubility explain superior antifungal profile of **7** over **PIM**.

### HPLC chemical stability tests of zwitterionic PIM derivatives at different pH

Exemplary compound **3** was subjected to HPLC stability tests at different pH (pH = 2, pH = 6.86 and pH =9.18) in the presence of internal standard (Figure S230). Analysis of chromatograms revealed that this compound is relatively stable at neutral or alkaline environment, in contrast to acidic conditions where the decomposition is observed probably due to the presence of epoxide moiety. Analogous experiments performed for other **PIM** derivatives as e.g. **7**, showed similar results regarding stability (data not shown). Taking into regard this result concerning **PIM** derivatives the intravenous rather than oral administration would be better solution.

## Conclusions

A series of 24 novel amino derivatives of **PIM** were synthesised by reductive amination in the form of zwitterions or zwitterionic/external salt. In turn, one *N*-benzyl/amide derivative was synthesised for structural and biological comparisons in a non-ionic form. Modifications of **PIM** mycosamine amino group with functionalised benzyl substituents generally increased water solubility and reduced toxicity in normal human dermal fibroblasts/HDF/and human umbilical vein endothelial cells (HUVEC). Among the compounds evaluated those bearing benzyl-*O*-aliphatic and methylene-nitrophenol substituents exhibited the best (MIC**_15_** *_C. albicans_* = 2 µg/mL) or the broadest-spectrum of antifungal activity (MIC**_7_** = 4–16 µg/mL against *C. albicans, C. glabrata, A. fumigatus*, *T. rubrum* and *F. oxysporum*). It should be noted that derivative **15**, the most active against *C. glabrata*, revealed MIC lower than the parent **PIM**, and reference compounds **NYS** and **FLU**. The ITC, NMR, FT-IR, and molecular modelling studies suggest that enhanced and broadened antifungal potency of **7** is likely associated with its ability to form of 6:1 supramolecular complexes with **ERG**. Subsequent incorporation of these complexes in fungal membranes, according to the known natural abundance of **ERG** regarding phospholipids, generated highly polar transmembrane channels, capable of transporting hydrated ions such as Na^+^ and small polar molecules, thereby disrupting fungal cell homeostasis as suggested by molecular modelling. This postulated mechanism differs substantially from that proposed for **PIM**, which predominantly forms 2:1 complexes with **ERG** and has been suggested to inhibit vacuolar fusion by effective binding with **ERG** (here experimentally determined K_D_ = 0.7 µM). In contrast, incorporation of fluorobenzyl substituents into polyene scaffold switched the biological profile from predominantly antifungal to anticancer one. Derivative **3**, bearing a 2-fluorobenzyl substituent, displayed remarkable antiproliferative activity and selectivity in PC-3 and SKOV-3 cell lines (IC_50_ = 0.6 µM at SI_HDF_ = 2.72; SI_HUVEC_ = 4.23), while exhibiting slightly lower toxicity compared to that of **PIM**. The anticancer potency of **3** was not only higher than **PIM** but also a more beneficent or comparable with those of **NYS**, **actD** or **mitC**. The HPLC chemical stability studies revealed that this-type derivatives are stable at pH = 6.86 and 9.18 but are unstable at pH = 2. Overall, the present study demonstrates that rational modification of the mycosamine amino group provides an effective strategy for tuning both the physico-chemical and biological profiles. The identified antifungal compound **7** and anticancer active compound **3** are promising candidates for further preclinical studies and highlight the potential of **PIM** derivatives as versatile platform for the development of novel antifungal or anticancer agents. Taking into regard good chemical stability of studied **PIM** derivatives in solutions at pH = 6 and 9 and low chemical stability at pH = 2 the administration is an important defiance for this type polyenes. Thus, this result creates interesting perspective for leader optimisation among new **PIM** derivatives, active against fungi (nitrophenol-containing congeners) or against cancer cell lines (fluorobenzyl-containing congeners) at reduced toxicity in normal cells.

## Supplementary Material

2026_JEIMC_PIM_PS_Supp_R1_f_clean_copy_anonymous__.docx

## Data Availability

All data associated with the article are placed in Supplementary Material and are available on request.
