# Supplementary material for: New antifungal and less toxic zwitterionic derivatives of 26-membered polyene antibiotic – pimaricin containing N-benzyl and N-alkyl moieties and their molecular mechanism
Source: J Enzyme Inhib Med Chem. 2026 Sep 10;41(1):2724228. doi: 10.1080/14756366.2026.2724228 (PMC13564172; doi:10.1080/14756366.2026.2724228)
Supplement: Supplementary file 1 [file IENZ_A_2724228_SM2072.docx]

Supplementary data

New antifungal and less toxic zwitterionic derivatives of 26-membered polyene antibiotic – pimaricin containing *N*-benzyl and *N*-alkyl moieties and their molecular mechanism

Contents:

[Spectral characteristic of PIM derivatives 1-25	11](#_Toc237346032)

[Fig. S1 FT-IR spectrum of PIM (in KBr).	38](#_Toc237346033)

[Fig. S2 1H NMR spectra of PIM in DMSO-d6.	38](#_Toc237346034)

[Fig. S3 ^13^C NMR spectra of PIM in DMSO-d_6_.	39](#_Toc237346035)

[Fig. S4 ^1^H-^13^C HSQC spectra of PIM in DMSO-d_6_.	39](#_Toc237346036)

[Fig. S5 ^1^H-^13^C HMBC spectra of PIM in DMSO-d_6_.	40](#_Toc237346037)

[Fig. S6 ^1^H-^1^H COSY spectra of PIM in DMSO-d_6_.	40](#_Toc237346038)

[Fig. S7 ^1^H-^1^H ROESY spectra of PIM in DMSO-d_6_.	41](#_Toc237346039)

[Fig. S8 ^1^H-^15^N gHSQC NMR spectrum of **PIM** revealing chemical shift of a broadened signal assigned to a highly exchangeable protons form group -NH_3_^+^.	41](#_Toc237346040)

[Fig. S9 FT-IR spectrum of compound **1** (in KBr).	42](#_Toc237346041)

[Fig. S10 ESI-MS spectrum of compound 1.	42](#_Toc237346042)

[Fig. S11 ^1^H NMR spectra of compound **1** in DMSO-d_6_.	43](#_Toc237346043)

[Fig. S12 ^13^C NMR spectra of compound **1** in DMSO-d_6_.	43](#_Toc237346044)

[Fig. S13 ^1^H-^13^C HSQC spectra of compound **1** in DMSO-d_6_.	44](#_Toc237346045)

[Fig. S15 ^1^H-^1^H COSY spectra of compound **1** in DMSO-d_6_.	45](#_Toc237346046)

[Fig. S16 ^1^H-^1^H ROESY spectra of compound **1** in DMSO-d_6_.	45](#_Toc237346047)

[Fig. S17 FT-IR spectrum of compound **2** (in KBr).	45](#_Toc237346048)

[Fig. S19 ^1^H NMR spectra of compound **2** in DMSO-d_6_.	47](#_Toc237346049)

[Fig. S21 ^1^H-^13^C HSQC spectra of compound **2** in DMSO-d_6_.	48](#_Toc237346050)

[Fig. S22 ^1^H-^13^C HMBC spectra of compound **2** in DMSO-d_6_.	48](#_Toc237346051)

[Fig. S23 ^1^H-^1^H COSY spectra of compound **2** in DMSO-d_6_.	49](#_Toc237346052)

[Fig. S24B Fragment of ^1^H-^15^N gHMBC NMR spectrum of **2** showing correlation between hydrogen atom (H-4’) of mycosamine and nitrogen atom from -NH_2_^+^-.	50](#_Toc237346053)

[Fig. S25 FT-IR spectrum of compound **3** (in KBr).	50](#_Toc237346054)

[Fig. S26 ESI-MS spectrum of compound **3**.	51](#_Toc237346055)

[Fig. S27 1H NMR spectra of compound 3 in DMSO-d_6_.	51](#_Toc237346056)

[Fig. S28 ^13^C NMR spectra of compound **3** in DMSO-d_6_.	52](#_Toc237346057)

[Fig. S29 ^1^H-^13^C HSQC spectra of compound **3** in DMSO-d_6_.	52](#_Toc237346058)

[Fig. S30 ^1^H-^13^C HMBC spectra of compound **3** in DMSO-d_6_.	53](#_Toc237346059)

[Fig. S31 ^1^H-^1^H COSY spectra of compound **3** in DMSO-d_6_.	53](#_Toc237346060)

[Fig. S32 ^1^H-^1^H ROESY spectra of compound **3** in DMSO-d_6_.	54](#_Toc237346061)

[Fig. S33 Structure of compound **3**	54](#_Toc237346062)

[Fig. S34 FT-IR spectrum of compound **4** (in KBr).	55](#_Toc237346063)

[Fig. S35 ESI-MS spectrum of compound **4**.	55](#_Toc237346064)

[Fig. S37 ^13^C NMR spectra of compound **4** in DMSO-d_6_.	56](#_Toc237346065)

[Fig. S38 ^1^H-^13^C HSQC spectra of compound **4** in DMSO-d_6_.	57](#_Toc237346066)

[Fig. S39 ^1^H-^13^C HMBC spectra of compound **4** in DMSO-d_6_.	57](#_Toc237346067)

[Fig. S40 ^1^H-^1^H COSY spectra of compound **4** in DMSO-d_6_.	58](#_Toc237346068)

[Fig. S41 ^1^H-^1^H ROESY spectra of compound **4** in DMSO-d_6_.	58](#_Toc237346069)

[Fig. S42 Structure of compound **4**	59](#_Toc237346070)

[Fig. S42A Fragment of 1H-1H ROESY spectra of compound **4**	59](#_Toc237346071)

[Fig. S43 FT-IR spectrum of compound **5** (in KBr).	60](#_Toc237346072)

[Fig. S45 ^1^H NMR spectra of compound **5** in DMSO-d_6_.	61](#_Toc237346073)

[Fig. S46 ^13^C NMR spectra of compound **5** in DMSO-d_6_.	61](#_Toc237346074)

[Fig. S47 ^1^H-^13^C HSQC spectra of compound **5** in DMSO-d_6_.	62](#_Toc237346075)

[Fig. S48 ^1^H-^13^C HMBC spectra of compound **5** in DMSO-d_6_.	62](#_Toc237346076)

[Fig. S49 ^1^H-^1^H COSY spectra of compound **5** in DMSO-d_6_.	63](#_Toc237346077)

[Fig. S50 ^1^H-^1^H ROESY spectra of compound **5** in DMSO-d_6_.	63](#_Toc237346078)

[Fig. S51 FT-IR spectrum of compound **6** (in KBr).	64](#_Toc237346079)

[Fig. S52 ESI-MS spectrum of compound **6**.	64](#_Toc237346080)

[Fig. S53 ^1^H NMR spectra of compound **6** in DMSO-d_6_.	65](#_Toc237346081)

[Fig. S54 ^13^C NMR spectra of compound **6** in DMSO-d_6_.	65](#_Toc237346082)

[Fig. S55 ^1^H-^13^C HSQC spectra of compound **6** in DMSO-d_6_.	66](#_Toc237346083)

[Fig. S56 ^1^H-^13^C HMBC spectra of compound **6** in DMSO-d_6_.	66](#_Toc237346084)

[Fig. S57 ^1^H-^1^H COSY spectra of compound **6** in DMSO-d_6_.	67](#_Toc237346085)

[Fig. S58 ^1^H-^1^H ROESY spectra of compound **6** in DMSO-d_6_.	67](#_Toc237346086)

[Fig. S59 FT-IR spectrum of compound **7** (in KBr).	68](#_Toc237346087)

[Fig. S60 ESI-MS spectrum of compound **7**.	68](#_Toc237346088)

[Fig. S61 ^1^H NMR spectra of compound **7** in DMSO-d_6_.	69](#_Toc237346089)

[Fig. S62 ^13^C NMR spectra of compound **7** in DMSO-d_6_.	69](#_Toc237346090)

[Fig. S63 ^1^H-^13^C HSQC spectra of compound **7** in DMSO-d_6_.	70](#_Toc237346091)

[Fig. S64 ^1^H-^1^H COSY spectra of compound **7** in DMSO-d_6_.	70](#_Toc237346092)

[Fig. S65 ^1^H-^1^H ROESY spectra of compound **7** in DMSO-d_6_.	71](#_Toc237346093)

[Fig. S66A Fragment of 1H NMR spectrum of compound 7, showing signals of exchangeable protons at the nitrogen of mycosamine	71](#_Toc237346094)

[Fig. S66B Fragment of ^1^H-^15^N gHMBC NMR spectrum of **7** showing correlation between hydrogen atom (H-4’) of mycosamine and nitrogen atom from -NH_2_^+^-.	72](#_Toc237346095)

[Fig. S67 Fragment of ^13^C NMR spectrum of compound **7**, showing lack of signals from TEA.	72](#_Toc237346096)

[Fig. S68 Fragment of ^1^H-^13^C HSQC NMR spectrum of compound **7**, showing lack of correlations from TEA.	73](#_Toc237346097)

[Fig. S69 FT-IR spectrum of compound **8** (in KBr).	73](#_Toc237346098)

[Fig. S70 ESI-MS spectrum of compound **8**.	74](#_Toc237346099)

[Fig. S71 ^1^H NMR spectra of compound **8** in DMSO-d_6_.	74](#_Toc237346100)

[Fig. S72 ^13^C NMR spectra of compound **8** in DMSO-d_6_.	75](#_Toc237346101)

[Fig. S73 ^1^H-^13^C HSQC spectra of compound **8** in DMSO-d_6_.	75](#_Toc237346102)

[Fig. S74 ^1^H-^13^C HMBC spectra of compound **8** in DMSO-d_6_.	76](#_Toc237346103)

[Fig. S75 ^1^H-^1^H COSY spectra of compound **8** in DMSO-d_6_.	76](#_Toc237346104)

[Fig. S76 FT-IR spectrum of compound **9** (in KBr).	77](#_Toc237346105)

[Fig. S77 ESI-MS spectrum of compound **9**.	77](#_Toc237346106)

[Fig. S78 ^1^H NMR spectra of compound **9** in DMSO-d_6_.	78](#_Toc237346107)

[Fig. S79 ^13^C NMR spectra of compound **9** in DMSO-d_6_.	78](#_Toc237346108)

[Fig. S80 ^1^H-^13^C HSQC spectra of compound **9** in DMSO-d_6_.	79](#_Toc237346109)

[Fig. S81 ^1^H-^13^C HMBC spectra of compound **9** in DMSO-d_6_.	79](#_Toc237346110)

[Fig. S82 ^1^H-^1^H COSY spectra of compound **9** in DMSO-d_6_.	80](#_Toc237346111)

[Fig. S83 ^1^H-^1^H ROESY spectra of compound **9** in DMSO-d_6_.	80](#_Toc237346112)

[Fig. S84 Fragment of ^1^H-^13^C HMBC spectra of **9** showing correlation between atom C-6” and protons H-4”a and H-4”b.	81](#_Toc237346113)

[Fig. S85 Fragment of ^1^H-^13^C HMBC spectra of **9** showing correlation between atom C-26 and protons H-11 and H-12.	81](#_Toc237346114)

[Fig. S86 FT-IR spectrum of compound **10** (in KBr).	82](#_Toc237346115)

[Fig. S87 ESI-MS spectrum of compound **10**.	82](#_Toc237346116)

[Fig. S88 ^1^H NMR spectra of compound **10** in DMSO-d_6_.	83](#_Toc237346117)

[Fig. S89 ^13^C NMR spectra of compound **10** in DMSO-d_6_.	83](#_Toc237346118)

[Fig. S90 ^1^H-^13^C HSQC spectra of compound **10** in DMSO-d_6_.	84](#_Toc237346119)

[Fig. S91 ^1^H-^13^C HMBC spectra of compound **10** in DMSO-d_6_.	84](#_Toc237346120)

[Fig. S92 ^1^H-^1^H COSY spectra of compound **10** in DMSO-d_6_.	85](#_Toc237346121)

[Fig. S93 ^1^H-^1^H ROESY spectra of compound **10** in DMSO-d_6_.	85](#_Toc237346122)

[Fig. S94 Fragment of ^13^C NMR spectrum of **10** showing singals from internal/external salt with TEA.	86](#_Toc237346123)

[Fig. S95 Fragment of ^1^H-^13^C HSQC NMR spectrum of compound **10** showing presence of internal/external salt with TEA.	86](#_Toc237346124)

[Fig. S96 FT-IR spectrum of compound **11** (in KBr).	87](#_Toc237346125)

[Fig. S97 ESI-MS spectrum of compound **11**.	87](#_Toc237346126)

[Fig. S98 ^1^H NMR spectra of compound **11** in DMSO-d_6_.	88](#_Toc237346127)

[Fig. S99 ^13^C NMR spectra of compound **11** in DMSO-d_6_.	88](#_Toc237346128)

[Fig. S100 ^1^H-^13^C HSQC spectra of compound **11** in DMSO-d_6_.	89](#_Toc237346129)

[Fig. S101 ^1^H-^13^C HMBC spectra of compound **11** in DMSO-d_6_.	89](#_Toc237346130)

[Fig. S102 ^1^H-^1^H COSY spectra of compound **11** in DMSO-d_6_.	90](#_Toc237346131)

[Fig. S103 ^1^H-^1^H ROESY spectra of compound **11** in DMSO-d_6_.	90](#_Toc237346132)

[Fig. S104 FT-IR spectrum of compound **12** (in KBr).	91](#_Toc237346133)

[Fig. S105 ESI-MS spectrum of compound **12**.	91](#_Toc237346134)

[Fig. S106 ^1^H NMR spectra of compound **12** in DMSO-d_6_.	92](#_Toc237346135)

[Fig. S107 ^13^C NMR spectra of compound **12** in DMSO-d_6_.	92](#_Toc237346136)

[Fig. S108 ^1^H-^13^C HSQC spectra of compound **12** in DMSO-d_6_.	93](#_Toc237346137)

[Fig. S109 ^1^H-^13^C HMBC spectra of compound **12** in DMSO-d_6_.	93](#_Toc237346138)

[Fig. S110 ^1^H-^1^H COSY spectra of compound **12** in DMSO-d_6_.	94](#_Toc237346139)

[Fig. S111 ^1^H-^1^H ROESY spectra of compound **12** in DMSO-d_6_.	94](#_Toc237346140)

[Fig. S112 Structure of **12**	95](#_Toc237346141)

[Fig. S113 FT-IR spectrum of compound **13** (in KBr).	96](#_Toc237346142)

[Fig. S114 ESI-MS spectrum of compound **13**.	96](#_Toc237346143)

[Fig. S115 ^1^H NMR spectra of compound **13** in DMSO-d_6_.	97](#_Toc237346144)

[Fig. S116 ^13^C NMR spectra of compound **13** in DMSO-d_6_.	97](#_Toc237346145)

[Fig. S117 ^1^H-^13^C HSQC spectra of compound **13** in DMSO-d_6_.	98](#_Toc237346146)

[Fig. S118 ^1^H-^13^C HMBC spectra of compound **13** in DMSO-d_6_.	98](#_Toc237346147)

[Fig. S119 ^1^H-^1^H COSY spectra of compound **13** in DMSO-d_6_.	99](#_Toc237346148)

[Fig. S120 ^1^H-^1^H ROESY spectra of compound **13** in DMSO-d_6_.	99](#_Toc237346149)

[Fig. S121 FT-IR spectrum of compound **14** (in KBr).	100](#_Toc237346150)

[Fig. S122 ESI-MS spectrum of compound **14**.	100](#_Toc237346151)

[Fig. S123 ^1^H NMR spectra of compound **14** in DMSO-d_6_.	101](#_Toc237346152)

[Fig. S124 ^13^C NMR spectra of compound **14** in DMSO-d_6_.	101](#_Toc237346153)

[Fig. S125 ^1^H-^13^C HSQC spectra of compound **14** in DMSO-d_6_.	102](#_Toc237346154)

[Fig. S126 ^1^H-^13^C HMBC spectra of compound **14** in DMSO-d_6_.	102](#_Toc237346155)

[Fig. S127 ^1^H-^1^H COSY spectra of compound **14** in DMSO-d_6_.	103](#_Toc237346156)

[Fig. S128 ^1^H-^1^H ROESY spectra of compound **14** in DMSO-d_6_.	103](#_Toc237346157)

[Fig. S129 FT-IR spectrum of compound **15** (in KBr).	104](#_Toc237346158)

[Fig. S130 ESI-MS spectrum of compound **15**.	104](#_Toc237346159)

[Fig. S131 ^1^H NMR spectra of compound **15** in DMSO-d_6_.	105](#_Toc237346160)

[Fig. S132 ^13^C NMR spectra of compound **15** in DMSO-d_6_.	105](#_Toc237346161)

[Fig. S133 ^1^H-^13^C HSQC spectra of compound **15** in DMSO-d_6_.	106](#_Toc237346162)

[Fig. S134 ^1^H-^13^C HMBC spectra of compound **15** in DMSO-d_6_.	106](#_Toc237346163)

[Fig. S135 ^1^H-^1^H COSY spectra of compound **15** in DMSO-d_6_.	107](#_Toc237346164)

[Fig. S136 ^1^H-^1^H ROESY spectra of compound **15** in DMSO-d_6_.	107](#_Toc237346165)

[Fig. S137 FT-IR spectrum of compound **16** (in KBr).	108](#_Toc237346166)

[Fig. S138 ESI-MS spectrum of compound **16**.	108](#_Toc237346167)

[Fig. S139 ^1^H NMR spectra of compound **16** in DMSO-d_6_.	109](#_Toc237346168)

[Fig. S140 ^13^C NMR spectra of compound **16** in DMSO-d_6_.	109](#_Toc237346169)

[Fig. S141 ^1^H-^13^C HSQC spectra of compound **16** in DMSO-d_6_.	110](#_Toc237346170)

[Fig. S142 ^1^H-^13^C HMBC spectra of compound **16** in DMSO-d_6_.	110](#_Toc237346171)

[Fig. S143 ^1^H-^1^H COSY spectra of compound **16** in DMSO-d_6_.	111](#_Toc237346172)

[Fig. S144 ^1^H-^1^H ROESY spectra of compound **16** in DMSO-d_6_.	111](#_Toc237346173)

[Fig. S145 FT-IR spectrum of compound **17** (in KBr).	112](#_Toc237346174)

[Fig. S146 ESI-MS spectrum of compound **17**.	112](#_Toc237346175)

[Fig. S147 ^1^H NMR spectra of compound **17** in DMSO-d_6_.	113](#_Toc237346176)

[Fig. S148 ^13^C NMR spectra of compound **17** in DMSO-d_6_.	113](#_Toc237346177)

[Fig. S149 ^1^H-^13^C HSQC spectra of compound **17** in DMSO-d_6_.	114](#_Toc237346178)

[Fig. S150 ^1^H-^13^C HMBC spectra of compound **17** in DMSO-d_6_.	114](#_Toc237346179)

[Fig. S151 ^1^H-^1^H COSY spectra of compound **17** in DMSO-d_6_.	115](#_Toc237346180)

[Fig. S152 ^1^H-^1^H ROESY spectra of compound **17** in DMSO-d_6_.	115](#_Toc237346181)

[Fig. S153 FT-IR spectrum of compound **18** (in KBr).	116](#_Toc237346182)

[Fig. S154 ESI-MS spectrum of compound **18**.	116](#_Toc237346183)

[Fig. S155 ^1^H NMR spectra of compound **18** in DMSO-d_6_.	117](#_Toc237346184)

[Fig. S156 ^13^C NMR spectra of compound **18** in DMSO-d_6_.	117](#_Toc237346185)

[Fig. S157 ^1^H-^13^C HSQC spectra of compound **18** in DMSO-d_6_.	118](#_Toc237346186)

[Fig. S158 ^1^H-^13^C HMBC spectra of compound **18** in DMSO-d_6_.	118](#_Toc237346187)

[Fig. S159 ^1^H-^1^H COSY spectra of compound **18** in DMSO-d_6_.	119](#_Toc237346188)

[Fig. S160 ^1^H-^1^H ROESY spectra of compound **18** in DMSO-d_6_.	119](#_Toc237346189)

[Fig. S161 Structure of 18 derivative	120](#_Toc237346190)

[Fig. S162 FT-IR spectrum of compound **19** (in KBr).	121](#_Toc237346191)

[Fig. S163 ESI-MS spectrum of compound **19**.	121](#_Toc237346192)

[Fig. S164 ^1^H NMR spectra of compound **19** in DMSO-d_6_.	122](#_Toc237346193)

[Fig. S165 ^13^C NMR spectra of compound **19** in DMSO-d_6_.	122](#_Toc237346194)

[Fig. S166 ^1^H-^13^C HSQC spectra of compound **19** in DMSO-d_6_.	123](#_Toc237346195)

[Fig. S167 ^1^H-^13^C HMBC spectra of compound **19** in DMSO-d_6_.	123](#_Toc237346196)

[Fig. S168 ^1^H-^1^H COSY spectra of compound **19** in DMSO-d_6_.	124](#_Toc237346197)

[Fig. S169 ^1^H-^1^H ROESY spectra of compound **19** in DMSO-d_6_.	124](#_Toc237346198)

[Fig. S170 FT-IR spectrum of compound **20** (in KBr).	125](#_Toc237346199)

[Fig. S171 ESI-MS spectrum of compound **20**.	125](#_Toc237346200)

[Fig. S172 ^1^H NMR spectra of compound **20** in DMSO-d_6_.	126](#_Toc237346201)

[Fig. S173 ^13^C NMR spectra of compound **20** in DMSO-d_6_.	126](#_Toc237346202)

[Fig. S174 ^1^H-^13^C HSQC spectra of compound **20** in DMSO-d_6_.	127](#_Toc237346203)

[Fig. S175 ^1^H-^13^C HMBC spectra of compound **20** in DMSO-d_6_.	127](#_Toc237346204)

[Fig. S176 ^1^H-^1^H COSY spectra of compound **20** in DMSO-d_6_.	128](#_Toc237346205)

[Fig. S177 ^1^H-^1^H ROESY spectra of compound **20** in DMSO-d_6_.	128](#_Toc237346206)

[Fig. S178 FT-IR spectrum of compound **21** (in KBr).	129](#_Toc237346207)

[Fig. S179 ESI-MS spectrum of compound **21**.	129](#_Toc237346208)

[Fig. S180 ^1^H NMR spectra of compound **21** in DMSO-d_6_.	130](#_Toc237346209)

[Fig. S181 ^13^C NMR spectra of compound **21** in DMSO-d_6_.	130](#_Toc237346210)

[Fig. S182 ^1^H-^13^C HSQC spectra of compound **21** in DMSO-d_6_.	131](#_Toc237346211)

[Fig. S183 ^1^H-^13^C HMBC spectra of compound **21** in DMSO-d_6_.	131](#_Toc237346212)

[Fig. S184 ^1^H-^1^H COSY spectra of compound **21** in DMSO-d_6_.	132](#_Toc237346213)

[Fig. S185 ^1^H-^1^H ROESY spectra of compound **21** in DMSO-d_6_.	132](#_Toc237346214)

[Fig. S186 FT-IR spectrum of compound **22** (in KBr).	133](#_Toc237346215)

[Fig. S187 ESI-MS spectrum of compound **22**.	133](#_Toc237346216)

[Fig. S188 ^1^H NMR spectra of compound **22** in DMSO-d_6_.	134](#_Toc237346217)

[Fig. S189 ^13^C NMR spectra of compound **22** in DMSO-d_6_.	134](#_Toc237346218)

[Fig. S190 ^1^H-^13^C HSQC spectra of compound **22** in DMSO-d_6_.	135](#_Toc237346219)

[Fig. S191 ^1^H-^13^C HMBC spectra of compound **22** in DMSO-d_6_.	135](#_Toc237346220)

[Fig. S192 ^1^H-^1^H COSY spectra of compound **22** in DMSO-d_6_.	136](#_Toc237346221)

[Fig. S193 ^1^H-^1^H ROESY spectra of compound **22** in DMSO-d_6_.	136](#_Toc237346222)

[Fig. S194 FT-IR spectrum of compound **23** (in KBr).	137](#_Toc237346223)

[Fig. S195 ESI-MS spectrum of compound **23**.	137](#_Toc237346224)

[Fig. S196 ^1^H NMR spectra of compound **23** in DMSO-d_6_.	138](#_Toc237346225)

[Fig. S197 ^13^C NMR spectra of compound **23** in DMSO-d_6_.	138](#_Toc237346226)

[Fig. S198 ^1^H-^13^C HSQC spectra of compound **23** in DMSO-d_6_.	139](#_Toc237346227)

[Fig. S199 ^1^H-^13^C HMBC spectra of compound **23** in DMSO-d_6_.	139](#_Toc237346228)

[Fig. S200 ^1^H-^1^H COSY spectra of compound **23** in DMSO-d_6_.	140](#_Toc237346229)

[Fig. S201 ^1^H-^1^H ROESY spectra of compound **23** in DMSO-d_6_.	140](#_Toc237346230)

[Fig. S202 FT-IR spectrum of compound **24** (in KBr).	141](#_Toc237346231)

[Fig. S203 ESI-MS spectrum of compound **24**.	141](#_Toc237346232)

[Fig. S204 ^1^H NMR spectra of compound **24** in DMSO-d_6_.	142](#_Toc237346233)

[Fig. S205 ^13^C NMR spectra of compound **24** in DMSO-d_6_.	142](#_Toc237346234)

[Fig. S206 ^1^H-^13^C HSQC spectra of compound **24** in DMSO-d_6_.	143](#_Toc237346235)

[Fig. S207 ^1^H-^13^C HMBC spectra of compound **24** in DMSO-d_6_.	143](#_Toc237346236)

[Fig. S208 ^1^H-^1^H COSY spectra of compound **24** in DMSO-d_6_.	144](#_Toc237346237)

[Fig. S209 ^1^H-^1^H ROESY spectra of compound **24** in DMSO-d_6_.	144](#_Toc237346238)

[Fig. S210 FT-IR spectrum of compound **25** (in KBr).	145](#_Toc237346239)

[Fig. S211 ESI-MS spectrum of compound **25**.	145](#_Toc237346240)

[Fig. S212 ^1^H NMR spectra of compound **25** in DMSO-d_6_.	146](#_Toc237346241)

[Fig. S213 ^13^C NMR spectra of compound **25** in DMSO-d_6_.	146](#_Toc237346242)

[Fig. S214 ^1^H-^13^C HSQC spectra of compound **25** in DMSO-d_6_.	147](#_Toc237346243)

[Fig. S215 ^1^H-^13^C HMBC spectra of compound **25** in DMSO-d_6_.	147](#_Toc237346244)

[Fig. S216 ^1^H-^1^H COSY spectra of compound **25** in DMSO-d_6_.	148](#_Toc237346245)

[Fig. S217 ^1^H-^1^H ROESY spectra of compound **25** in DMSO-d_6_.	148](#_Toc237346246)

[Fig. S218 Shielding of H3’ proton signal in 1H NMR spectra	149](#_Toc237346247)

[ITC titration of ergosterol solutions with PIM and its derivatives solutions in DMSO	149](#_Toc237346248)

[Fig. S219 Thermal effects	156](#_Toc237346249)

[Fig. S220 Thermal effects of the direct interaction between **ERG** and ligands in DMSO at 25℃	158](#_Toc237346250)

[Fig. S221 Fragment of 1H NMR spectrum of mixture of compound **7** and **ERG (6:1**)	160](#_Toc237346251)

[Fig. S223 Model of Internal/ external salt of compound **10**	161](#_Toc237346252)

[Fig. S224 FT-IR spectrum of **PIM**, **7**, **2** showing absorption continuum measured in DMSO (with traces of water).	161](#_Toc237346253)

[Table S1 Summary of chemical C atoms shifts [ppm] for **PIM** and derivatives **1**-**14** from ^13^C NMR spectra.	162](#_Toc237346254)

[Table S2 Summary of chemical C atoms shifts [ppm] for derivatives **15-25** from ^13^C NMR spectra.	166](#_Toc237346255)

[Table S3 Summary of chemical H atoms shifts [ppm] for PIM and derivatives **1-5** from ^1^H NMR spectra.	171](#_Toc237346256)

[Table S4 Summary of chemical H atoms shifts [ppm] for PIM derivatives **7-13** from ^1^H NMR spectra.	179](#_Toc237346257)

[Table S5 Summary of chemical H atoms shifts [ppm] for PIM derivatives **14-20** from ^1^H NMR spectra.	188](#_Toc237346258)

[Table S6 Summary of chemical H atoms shifts [ppm] for PIM derivatives **21-25** from ^1^H NMR spectra.	199](#_Toc237346259)

[Fig. S225 The **PIM** molecule in the **PIM2** crystal structure with the labelling scheme for non-hydrogen atoms. Displacement ellipsoids are shown at the 50% probability level.	206](#_Toc237346260)

[Fig. S226 The **PIM** molecule in the **PIM2** crystal structure with the labels shown for O and N atoms. atoms. Displacement ellipsoids are shown at the 20% probability level.	206](#_Toc237346261)

[Fig. S227 O-H···O and N-H···O hydrogen bonding interactions connecting PIM molecules into a 1D network extending along the a axis in the crystal form **PIM2**.	207](#_Toc237346262)

[Fig. S228 O-H···O and N-H···O hydrogen bonding interactions connecting PIM molecules into a 1D network extending along the a axis in the crystal form **PIM1**	207](#_Toc237346263)

[Fig. S229 Solvate accessible area in the crystal structure of **PIM1.**	208](#_Toc237346264)

[Table S7. Crystal data and details of structure refinement	209](#_Toc237346265)

[210](#_Toc237346266)

[Fig. S230 Chromatograms of chemical stability of compound **3** in solutions of different pH with 2-thiophenecarboxylic hydrazide as internal standard.	211](#_Toc237346267)

# Spectral characteristic of PIM derivatives 1-25

**PIM** (1R,3S,5R,7R,8E,12R,14E,16E,18E,20E,22R,24S,25R,26S)-22-(((2S,3S,4S,5S,6R)-4-ammonio-3,5-dihydroxy-6-methyltetrahydro-2H-pyran-2-yl)oxy)-1,3,26-trihydroxy-12-methyl-10-oxo-6,11,28-trioxatricyclo[22.3.1.05,7]octacosa-8,14,16,18,20-pentaene-25-carboxylate, ESI-MS *m/z*: [M+H]^+^ 665.73; melting point : 242-244°C with decomposition; FT-IR (KBr): ν(O-H) 3489 cm^-1^, ν(=C-H) 3276 cm^-1^, ν(C-H) 3017 cm^-1^, ν(N-H) 2945 cm^-1^, ν(C=O) 1715 cm^-1^, ν(C=C) 1637 cm^-1^, δ(C-OH) 1291 cm^-1^, ν(C-O-C) 1059 cm^-1^

^1^H NMR (600 MHz, dmso-d_6_) δ 7.24^×^ (H- NH_3_^+^), 6.49 (dd, *^3^J*_H18-H19_=14.6 Hz, *^3^J*_H18-H17_=10.8 Hz, 1H, H-18), 6.27 (dd, *^3^J*_H2-H3_=15.8 Hz, *^3^J*_H3-H4_=7.7 Hz, 1H, H-3), 6.24-6.16. (m, 3H, H-19,H-20, H-21), 6.13-6.11 (m, 1H, H-2), 6.10-6.01 (m, 2H, H-17,H-22), 5.91 (dd, *^3^J*_H16-H17_=15.3 Hz, *^3^J*_H15-H16_=8.8 Hz, 1H, H-16), 5.60 (ddd, *^3^J*_H22-H23_=15.4Hz, *^3^J*_H23-H24a_=9.7 Hz, *^3^J*_H23-H24b_=5.9 Hz, 1H, H-23), 5.22^#^ (C_9_-OH), 4.68-4.62 (m, 1H, H-25), 4.48 (bs, 1H, H-1’), 4.41-4.36 (m, 1H, H-15), 4.21-4.18 (m, 1H, H-13), 4.16-4.12 (m. 1H. H-7), 4.01 (td, *^3^J*_H8a-H7_ =10.7 Hz, *^3^J*_H11-10a_= 4.7 Hz, 1H, H-11), 3.75 (d, *^3^J*_H2’-H3’_ =3.1 Hz, 1H, H-2’), 3.23-3.19 (m, 2H, H-4, H-5’), 3.13-3.09 (m, 1H, H-4’) 2.79-2.74 (m, 2H, H-5, H-3’), 2.38-2.32 (m, 1H, H-24b) 2.24-2.15 (m, 2H, H-14a, H-24a), 1.98-1.93 (m, 1H, H-6b), 1.85-1.78(m, 3H, H-10a, H-12), 1.57 (dd, *^2^J*_H8a-H8b_=14.4 Hz, *^3^J*_H7-H8a_=10.4 Hz, 1H, H-8a), 1.49-1.45 (m, 2H, H-8b, H-14b), 1.26 (d, *^3^J*_H25-H27_ =6.2 Hz, 3H, H-27), 1.17 (d, *^3^J*_H5’-H6’_ =6 Hz, 3H, H-6’), 1.16-1.12 (m, 1H, H-6a), 1.10-1.07 (m, 1H, H-10b)

^#^- determined from ^1^H-^1^H ROESY NMR

^13^C NMR (126 MHz, dmso-d_6_) δ 175.1^ (C-26), 164.3 (C-1), 144.4 (C-3), 136.3 (C-16), 135.4 (C-22), 133.4 (C-18), 131.9 (C-20), 131.3 (C-21), 131.1 (C-19), 128.5 (C-23), 128.3 (C-17), 124.5 (C-2), 96.9 (C-9), 95.7 (C-1’), 74.2 (C-15), 72.7 (C-5’), 70.4 (C-4’), 69.5 (C-25), 68.1 (C-2’), 66.1 (C-7), 65.3 (C-11), 65.00 (C-13), 58.1 (C-12), 57.9 (C-5), 56.2 (C-3’), 53.6 (C-4), 46.4 (C-8), 44.2 (C-10), 40.8 (C-6), 38.8*(C-24), 36.4 (C-14), 20.1 (C-27), 18.8 (C-6’)

*-overlapped, ^- determined from ^1^H-^13^C HMBC NMR, ×- determined from ^1^H-^15^N HSQC NMR,

*Compound* ***1*** (1R,3S,5R,7R,8E,12R,14E,16E,18E,20E,22R,24S,25R,26S)-22-(((2S,3S,4S,5S,6R)-4-((4-ethynylbenzyl)ammonio)-3,5-dihydroxy-6-methyltetrahydro-2H-pyran-2-yl)oxy)-1,3,26-trihydroxy-12-methyl-10-oxo-6,11,28-trioxatricyclo[22.3.1.05,7]octacosa-8,14,16,18,20-pentaene-25-carboxylate (90 mg, yield 75%), Elemental analysis C_42_H_53_NO_13_: C, 64.61; H, 6.82; N, 1.74; ESI-MS *m/z*: [M+ Na]^+^ 824.0, [M-H]^-^  778.0; FT-IR (KBr): ν(O-H) 3384 cm^-1^, ν(C-H)_ar_ 3297 cm^-1^, ν(C-H) 2977 cm^-1^, ν(N-H) 3017 cm^-1^ , ν(C ≡C) 2105cm^-1^, ν(C=O) 1712 cm^-1^, ν(C=C) 1650 cm^-1^, δ(C-OH) 1303 cm^-1^, ν(C-O-C) 1059 cm^-1^, γ(=C-H)_ar_ 841 cm^-1^

^1^H NMR (600 MHz, dmso-d_6_) δ 10.50 (H_a_-NH_2_^+^), 9.55 (H_b_-NH_2_^+^), 7.41 (s, 1H, H-4”a; H-4”b) , 7.40 (s, 1H, H-3”a; H-3”b), 6.49 (dd,^3^*J*_H18-H19_=14.6 Hz, ^3^*J*_H17-H18_=10.8 Hz 1H, H-18), 6.26 (dd, *^3^J*_H3-H2_= 15.8 Hz, *^3^J*_H3-H4_=7.7 Hz, 1H, H-3), 6.21-6.16 (m, 3H, H-19, H-20, H-21), 6.15-6.10 (m, 1H H-2), 6.09-6.05 (m, 2H, H-17, H-22), 5.92-5.86 (m, 1H, H-16), 5.60 (ddd, *^3^J*_H23-H22_= 15.3Hz; *^3^J*_H23-H24a_= 9.6 Hz, *^3^J*_H23-H24b_= 5.9Hz, 1H, H-23), 5.24^#^ (C_9_-OH), 4.70-4.64 (m, 1H, H-25), 4.40-4.36 (m, 2H, H-15), 4.33 (bs, 1H, H-1’), 4.22-4.20 ( m, 1H, H-13), 4.19-4.10 (m, 4H, H-7), 4.06 (s, 1H, H-7”), 4.06-4.01(m, 1H, H-11), 3.87 (d, *^2^J*_H1”a-H1”b_= 13.9Hz, 1H, H-1”a), 3.76 (d, *^3^J*_H2’-H3’_= 3.1 Hz, 1H, H-2’), 3.69 (d, *^2^J*_H1”a-H1”b_= 13.8 Hz, 1H, H-1”b), 3.22 (dd, *^3^J*_H3-H4_= 7.7 Hz, *^4^J*_H2-H4_ = 2.0 Hz, 1H, H-4), 3.12-3.09 (m, 1H, H-5’), 3.02-2.98 m, 1H, H-4’), 2.76-2.73 (m, 1H, H-5), 2.42-2.35 (m, 2H, H-3’, H-24b), 2.19-2.14 ( m, 1H, H-24a), 2.05-2.00 (m, 1H, H-14a), 1.98-1.90 (m, 3H, H-6b; H-12), 1.85 ( dd, *^2^J*_H10a-H10b_= 12.4 Hz, *^3^J*_H10a-H11_= 4.7 Hz, 1H, H-10a), 1.63-1.53 (m, 2H, H-8a, H-14b), 1.51-1.47 (m, 1H, H-8b), 1.50-1.48 (m, 1H, H-8a) 1.26 (d, *^3^J*_H25-H27_= 6.1 Hz, 3H, H-27), 1.15 (d, *^3^J*_H5’-H6’_= 6.1 Hz, 3H, H-6’), 1.14-1.07 (m, 2H, H-10b, H-6a),

^#^- determined from ^1^H-^1^H ROESY NMR

^13^C NMR (126 MHz, dmso-d_6_) δ 174.0 (C-26), 164.3 (C-1), 144.4 (C-3), 142.1 (C-2’’), 136.3 (C-16), 135.4 (C-22), 133.4 (C-18), 131.9 (C-4’’a; C-4”b, C-20), 131.3 (C-21), 131.1 (C-19), 128.5 (C-17), 128.4 (C-3”a; C-3”b), 124.5 (C-2), 119.8 (C-5’’), 97.1 (C-9), 97.0 (C-1’), 83.5 (C-6”), 80.1 (C-7”) , 74.3 (C-15), 73.00 (C-5’), 70.9 (C-4’), 69.5 (C-25), 66.1 (C-2’), 66.0 (C-7), 65.2 (C-11), 65.1 (C-13), 62.42 (C-3’), 57.9 (C-5), 57.1 (C-12), 53.7 (C-4), 49.4 (C-1’’), 46.3 (C-8), 44.1 (C-10), 40.75 (C-6) 38.9* (C-24), 36.9 (C-14), 20.06 (C-27), 18.0 (C-6’).

*-overlapped

*Compound* ***2*** (1R,3S,5R,7R,8E,12R,14E,16E,18E,20E,22R,24S,25R,26S)-22-(((2S,3S,4S,5S,6R)-4-((3-ethynylbenzyl)ammonio)-3,5-dihydroxy-6-methyltetrahydro-2H-pyran-2-yl)oxy)-1,3,26-trihydroxy-12-methyl-10-oxo-6,11,28-trioxatricyclo[22.3.1.05,7]octacosa-8,14,16,18,20-pentaene-25-carboxylate (74 mg, yield 63.2%), Elemental analysis C_42_H_53_NO_13_: C, 64.68; H, 6.85; N, 1.80; ESI-MS *m/z*: [M + H]^+^ 781.0, [M -H]^-^ 778.0; melting point : 150-152°C; FT-IR (KBr): ν(O-H) 3389 cm^-1^, ν(C-H)_ar_ 3295 cm^-1^, ν(C-H) 2977 cm^-1^, ν(N-H) 3017 cm^-1^, ν(C≡C) 2100 cm^-1^, ν(C=O) 1714 cm^-1^, ν(C=C) 1653 cm^-1^, δ(C-OH) 1300 cm^-1^, ν(C-O-C) 1059 cm^-1^, γ(=C-H)_ar_ 841 cm^-1^

^1^H NMR (600 MHz, dmso-d_6_) δ 10.01 (H_a_-NH_2_^+^), 9.54 (H_b_-NH_2_^+^), 7.51 (s, 1H, H-3”), 7.43 (d, *^3^J*_H5”-H6”_=2.4 Hz, 1H, H-5”) , 7.35-7.29 (m, 6H, H-6’’; H-7’’), 6.49 (dd,^3^*J*_H18-H19_=14.7 Hz, ^3^*J*_H18-H17_=10.7 Hz 1H, H-18), 6.27 (dd,*^3^J*_H2-H3_=15.8Hz, *^3^J*_H3-H4_=7.7Hz, 1H, H-3), 6.24-6.16 (m, 3H, H-19, H-20, H-21), 6.14-6.10 (m, 1H H-2), 6.09-6.05 (m, 2H, H-17, H-22), 5.90 (dd, *^3^J*_H16-H17_=15.3 Hz, *^3^J*_H16-H15_=8.8 Hz,1H, H-16), 5.60 (ddd, *^3^J*_H23-H22_=15.4, *^3^J*_H23-H24a_= 9.6 Hz, *^3^J*_H23-H24b_= 5.8Hz, 1H, H-23), 5.20^#^ (C_9_-OH), 4.70-4.65 (m, 1H, H-25), 4.38 (bs, 1H, H-1’), 4.38-4.30 (m, 3H, H-15), 4.25-4.18 ( m, 3H, H-13), 4.17-4.13 (m, 1H, H-7), 3.99-3.95 (m, 1H, H-11), 3.85 (d, *^2^J*_H1”a-H1”b_= 13.6 Hz, 1H, H-1”a), 3.75 (d, *^3^J*_H2’-H3’_= 3.1 Hz, 1H, H-2’), 3.65 (d, *^2^J*_H1”a-H1”b_= 13.6 Hz, 1H, H-1”b), 3.22 (dd, *^3^J*_H3-H4_= 7.7 Hz, *^4^J*_H2-H4_ = 2.0 Hz, 1H, H-4), 3.15-3.08 (m, 1H, H-5’), 3.01-2.97 (m, 1H, H-4’), 2.77-2.74 (m , 1H, H-5), 2.43-2.35 (m, 2H, H-3’, H-24b), 2.19-2.15 ( m, 1H, H-24a), 2.11-2.07 (m, 1H, H-14a), 1.96-1.92 (m, 1H, H-6b), 1.88-1.86 (m, 1H, H-12), 1.85 ( dd, *^2^J*_H10a-H10b_= 12.4 Hz, *^3^J*_H10a-H11_= 4.9 Hz, 1H, H-10a), 1.63-1.54 (m, 6H, H-8a, H-14b), 1.51-1.47 (m, 1H, H-8b), 1.26 (d, *^3^J*_H25-H27_= 6.2 Hz, 3H, H-27), 1.15 (d, *^3^J*_H5’-H6’_= 6.1 Hz 3H, H-6’), 1.13-1.06 (m, 2H, H-10b, H-6a),

^#^- determined from ^1^H-^1^H ROESY NMR

^13^C NMR (126 MHz, dmso-d_6_) δ 174.26 (C-26), 164.3 (C-1), 144.4 (C-3), 141.6 (C-2’’), 136.5 (C-16), 135.4 (C-22), 133.5 (C-18), 131.9 (C-20), 131.2 (C-21), 131.00 (C-19), 129.7 (C-7’’), 129.6 (C-5”), 128.5 (C-6’’) 128.4 (C-23), 128.3 (C-17), 127.0 (C-3’’), 124.4 (C-2), 121.4 (C-4’’), 97.3 (C-1’), 97.00 (C-9), 83.6 (C-8”), 80.1 (C-9”), 74.5 (C-15), 73.00 (C-5’), 70.9 (C-4’), 69.5 (C-25), 66.1 (C-2’), 65.9 (C-7), 65.2*(C-11; C-13), 62.5 (C-3’), 57.8 (C-5), 57.7 (C-12), 53.7 (C-4), 49.2 (C-1’’), 46.4 (C-8), 44.1 (C-10), 40.8 (C-6), 38.8* (C-24), 37.2 (C-14), 20.0 (C-27), 18.0 (C-6’)

*-overlapped

^15^N NMR (28 kHz, dmso-d_6_ with traces of water) δ 342.2* (N-mycosamine)

*-determined from ^1^H-^15^N gHMBC NMR

*Compound* ***3*** (1R,3S,5R,7R,8E,12R,14E,16E,18E,20E,22R,24S,25R,26S)-22-(((2S,3S,4S,5S,6R)-4-((2-fluorobenzyl)ammonio)-3,5-dihydroxy-6-methyltetrahydro-2H-pyran-2-yl)oxy)-1,3,26-trihydroxy-12-methyl-10-oxo-6,11,28-trioxatricyclo[22.3.1.05,7]octacosa-8,14,16,18,20-pentaene-25-carboxylate (64 mg, yield 55%), Elemental analysis C_40_H_52_FNO_13_: C, 62.00; H, 6.70; N, 1.80; ESI-MS *m/z*: [M + Na]^+^  796.0, [M-H]^-^ for 772.0; melting point : 154-156°C ; FT-IR (KBr): ν(O-H) 3401 cm^-1^, ν(C-H)_ar_ 3297 cm^-1^, ν(C-H) 2928 cm^-1^, ν(N-H) 3017 cm^-1^, ν(C=O) 1713 cm^-1^, ν(C=C) 1576 cm^-1^, δ(C-OH) 1424 cm^-1^, ν(C-O-C) 1289 cm^-1^, γ(=C-H)_ar_ 942 cm^-1^, ν(C-F) 540 cm^-1^

^1^H NMR (500 MHz, dmso-d_6_) δ 10.24 (H_a_-NH_2_^+^), 9.50 (H_b_-NH_2_^+^), 7.56-7.50 (m, 1H, H-4’’), 7.28 (tdd, *^3^J*_H4”-H5”_ = 7.5 Hz, *^3^J*_H5’’-H6’’_ = 5.3 Hz, 1H, H-6’’), 7.19-7.14 (m, 2H, H-5’’; H7’’), 6.49 (dd, *^3^J*_H18-H19_ = 14.7 Hz, *^3^J*_H17-H18_ = 10.7 Hz, 1H, H-18); 6.28 (dd, *^3^J*_H3-H2_= 15.8 Hz, *^3^J*_H3-H4_=7.7 Hz, 1H, H-3), 6.26-6.19-6.14 (m, 3H, H-19, H-20; H-21), 6.10-6.08 (m, 1H, H-2), 6.08-6.05 (m, , H-17; H-22), 5.89 (dd, *^3^J*_H16-H17_ = 15.3 Hz, *^3^J*_H15-H16_ = 8.7 Hz, 1H, H-16), 5.60 (ddd, *^3^J*_H22-H23_= 15.3 Hz, *^3^J*_H23-H24a_ =9.6 Hz, *^3^J*_H23-H24b_ = 5.9 Hz, 1H, H-23), 5.21^#^ (C_9_-OH), 4.74-4.66 (m, 1H, H-25), 4.38-4.35 (m, 1H, H-15), 4.34 (bs,1H, H-1’), 4.22-4.19 (m, 1H, H-13), 4.14-4.10 (m, 1H, H-7), 4.01-3.97 (m, 1H, H-11), 3.89 (d, *^2^J*_H1’’a-H1’’b_ = 13.9 Hz, 1H, H-1’’a), 3.78 (d, *^3^J*_H2’-H3’_ = 3 Hz, 1H, H-2’), 3.74 ( d, *^2^J*_H1’’a-H1’’b_ = 13.9 Hz, 1H, H-1’’b), 3.22 (dd, *^3^J*_H3-H4_ = 7.6 Hz, *^4^J*_H2-H4_ = 2.0 Hz, 1H, H-4), 3.12-3.07 (m, 1H, H-5’), 3.02-2.97 (m, 1H, H-4’), 2.77-2.74 (m, 1H, H-5), 2.43 (dd, *^3^J*_H3’-H4’_ = 9.6 Hz, *^3^J*_H2’-H3’_ = 3.0 Hz, 1H, H-3’), 2.41-2.35 (m, 1H, H-24b), 2.19-2.14 (m, 1H, H-24a), 2.09-2.02 (m, 1H, H-14a), 1.96-1.92 (m, , H-12; H-6b), 1.85 (dd, *^2^J*_H10a-H10b_= 10.8 Hz, *^3^J*_H10a-H11_= 4.9 Hz, 1H, H-10a), 1.59-1.53 (m, 2H, H-8a; H-14b), 1.51 (dd, *^2^J*_H8a-H8b_= 14.2 Hz, *^3^J*_H8b-H7_= 2.0 Hz ,1H, H-8b), 1.27 (d, *^3^J*_H25-H27_ = 6.0 Hz, 3H, H-27), 1.15-1.06 (m, 3H, H-6a, H-10b; H-6’)

^#^- determined from ^1^H-^1^H ROESY NMR

^13^C NMR (126 MHz, dmso-d_6_) δ 174.0 (C-26), 164.3 (C-1), 160.3(C-3’’), 144.4 (C-3), 136.3 (C-16), 135.4 (C-22), 133.4 (C-18), 131.9 (C-20), 131.3 (C-21), 131.0 (C-19), 130.19 (C-4’’), 128.3 (C-23), 128.2 (C-17), 127.6 (C-6’’), 124.5 (C-2),124.4 (C-2’’), 124.0 (C-5’’), 114.7 (C-7’’), 97.1 (C-9), 97.0 (C-1’), 74.3 (C-15), 73.00 (C-5’), 70.9 (C-4’), 69.6 (C-25), 66.2 (C-2’), 65.9 (C-7), 65.1 (C-11), 64.7 (C-13), 62.5 (C-3’), 57.8 (C-5), 57.0 (C-12), 53.7 (C-4), 46.3 (C-8), 44.1 (C-10), 43.0 (C-1’’), 40.8 (C-6), 39.0* (C-24), 37.0 (C-14), 20.0 (C-27), 18.0 (C-6’)

*-overlapped,

*Compound* ***4*** (1R,3S,5R,7R,8E,12R,14E,16E,18E,20E,22R,24S,25R,26S)-22-(((2S,3S,4S,5S,6R)-4-((3-fluorobenzyl)ammonio)-3,5-dihydroxy-6-methyltetrahydro-2H-pyran-2-yl)oxy)-1,3,26-trihydroxy-12-methyl-10-oxo-6,11,28-trioxatricyclo[22.3.1.05,7]octacosa-8,14,16,18,20-pentaene-25-carboxylate (40 mg, yield 54.4%), Elemental analysis C_40_H_52_FNO_13_: C, 62.08; H, 6.77; N, 1.81; ESI-MS *m/z*: [M+Na]^+^ 796.0, [M-H]^-^ 792.0, melting point : 154-156°C ;FT-IR (KBr): ν(O-H), 3401 cm^-1^, ν(C-H)_ar_ 3017 cm^-1^, ν(C-H) 2977 cm^-1^, ν(N-H) 3017 cm^-1^ ν(C=O) 1713 cm^-1^, ν(C=C) 1575 cm^-1^, δ(C-OH) 1424 cm^-1^, ν(C-O-C) 1289 cm^-1^, γ(=C-H)_ar_ 942 cm^-1^, ν(C-F) 541 cm^-1^

^1^H NMR (600 MHz, dmso-d_6_) δ 10.02 (H_a_-NH_2_^+^), 9.53 (H_b_-NH_2_^+^), 7.33-7.30 (m, 1H, H-6’’), 7.30-7.25 m, 1H, H-3’’), 7.21 (d, *^3^J*_H6’’-H7’’_= 7.6 Hz, 1H, H-7’’), 7.02 (td, *^3^J*_H5’’-H6’’_= 8.6 Hz, 1H, H-5’’), 6.49 (dd, *^3^J*_H18-H19_ = 14.6 Hz, *^3^J*_HH17-H18_ = 10.8 Hz, 1H, H-18), 6.27 (dd, *^3^J*_H3-H2_= 15.8 Hz, *^3^J*_H3-H4_=7.7 Hz, 1H, H-3), 6.21-6.14 (m, 3H, H-19, H-21, H-20), 6.09-6.05 (m, 3H, H-22; H-17; H-2), 5.87-5.82 (m, 1H, H-16), 5.62-5.58 (m, 1H, H-23), 5.24^#^ (C_9_-OH), 4.74-4.64 (m, 1H, H-25), 4.38 (ddd, *^3^J*_H16-H15_ =8.3 Hz, *^3^J*_H14a-H15_ = 4.6 Hz, *^4^J*_H15-H17_ =2.7 Hz ,1H, H-15), 4.33 (bs, 1H, H-1’), 4.25-4.19 (m, 1H, H-13), 4.13-4.10 (m, 1H, H-7), 4.02 (td, *^3^J*_H10a-H11_=4.7 Hz, 1H, H-11), 3.87 (d, *^2^J*_H1’’a-H1’’b_= 13.9 Hz, 1H, H-1’’a), 3.76 (d, *^3^J*_H2’-H3’_ = 3.1 Hz, 1H, H-2’), 3.70 (*^2^J*_H1’’a-H1’’b_= 14 Hz, 1H, H-1’’b), 3.22 (dd, *^3^J*_H3-H4_= 7.6 Hz, *^4^J*_H2-H4_ = 2.0 Hz, 1H, H-4), 3.13-3.10 (m, 1H, H-5’), 3.02 (m, 1H, H-4’), 2.77-2.73 (m , 1H, H-5), 2.41-2.38 (m, 2H, H-24b; H-3’), 2.21-2.19 (m, 1H, H-24a), 2.05-2.00 (m, 1H, H-14a), 1.95-1.92 (m, 1H, H-12), 1.92-1.89 ( m, 1H, H-6b), 1.85 (dd, *^2^J*_H10a-H10b_=12.3 Hz, *^3^J*_H10a-H11_= 4.9 Hz, 1H, H-10a), 1.60-1.55 (m, 2H, H-8a; H-14b), 1.52 (dd, *^2^J*_H8a-H8b_= 14.2 Hz, *^3^J*_H8b-H7_= 2.0 Hz 1H, H-8b), 1.26-1.24 (m, 3H, H-27), 1.19-1.12 (m,5H, H-6a; H-10b; H-6’),

^#^- determined from ^1^H-^1^H ROESY NMR

^13^C NMR (151 MHz, dmso-d_6_) δ 174.0 (C-26), 164.3 (C-1), 162.20 (C-4’’), 144.4 (C-3), 144.1 (C-2’’), 136.29 (C-16), 135.4 (C-22), 133.42 (C-18), 131.9 (C-20), 131.3 (C-21), 131.1 (C-19), 129.7 (C-6’’), 128.46 (C-23), 128.40 (C-17), 124.4 (C-2), 123.7 (C-7’’), 114.4 (C-3’’), 113.1 (C-5’’), 97.1 (C-9), 97.0 (C-1’), 74.3 (C-15), 73.0 (C-5’), 70.9 (C-4’), 69.5 (C-25), 66.1 (C-2’), 65.9 (C-7), 65.0 (C-11), 64.7 (C-13), 62.4 (C-3’), 57.8 (C-5), 57.0 (C-12), 53.7 (C-4), 49.0 (C-1’’), 46.3 (C-8), 44.1 (C-10), 40.7 (C-6), 39.0* (C-24), 36.9 (C-14), 20.0 (C-27), 18.0 (C-6’)

*-overlapped

*Compound* ***5*** (1R,3S,5R,7R,8E,12R,14E,16E,18E,20E,22R,24S,25R,26S)-22-(((2S,3S,4S,5S,6R)-3,5-dihydroxy-6-methyl-4-((4-(trifluoromethyl)benzyl)ammonio)tetrahydro-2H-pyran-2-yl)oxy)-1,3,26-trihydroxy-12-methyl-10-oxo-6,11,28-trioxatricyclo[22.3.1.05,7]octacosa-8,14,16,18,20-pentaene-25-carboxylate (45 mg, yield 36.4%), Elemental analysis C_41_H_52_F_3_NO_13_: C, 59.79; H, 6.31; N, 1.68; ESI-MS *m/z*: [M+Na]^+^ 846.0, [M-H]^-^ 822.0; melting point : 140-141°C ; FT-IR (KBr): ): ν(O-H) 3372 cm^-1^, ν(N-H) 3372 cm^-1^, ν(C-H)_ar_ 3018 cm^-1^, ν(C-H) 2978 cm^-1^, ν(C=O) 1715 cm^-1^, ν(C=C) 1574 cm^-1^, ν(C-O-C) 1265 cm^-1^, ν(C-O)1123 cm^-1^, γ(=C-H)_ar_ 946 cm^-1^, ν(C-F) 594 cm^-1^

^1^H NMR (600 MHz, dmso-d_6_) δ 10.20 (H_a_-NH_2_^+^), 8.80 (H_b_-NH_2_^+^), 8.06 (s, 2H, H-4”a, H-4”b), 7.64-7.61 (m, 2H, H-3”a, H-3”b), 6.49 (dd, *^3^J*_H18-H19_ = 14.5Hz, *^3^J*_H18-H17_ = 10.8 Hz, 1H, H-18), 6.27 (dd, *^3^J*_H2-H3_ = 15.7 Hz, *^3^J*_H3-H4_ = 7.8 Hz, 1H, H-3), 6.21-6.16 (m, 3H, H-19, H-21, H-20), 6.15-6.06 (m, 3H, H-22; H-17; H-2), 5.89 (dd, *^3^J*_H16-H17_ = 15.3 Hz, *^3^J*_H16-H15_ = 8.8 Hz, 1H, H-16), 5.60 (ddd, *^3^J*_H22-H23_ = 15.3 Hz, *^3^J*_H23-H24a_ = 9.6 Hz, *^3^J*_H23-H24b_ = 5.9 Hz, 2H, H-23), 5.24^#^ (C_9_-OH), 4.69-4.64 (m, 1H, H-25), 4.37-4.34 (m, 4H, H-15), 4.35 (bs, 1H, H-1’), 4.25-4.20 (m, 1H, H-13), 4.16-4.10 (m, 1H, H-7), 4.02 (td, *^3^J*_H12-H11_=10.7Hz, *^3^J*_H11-10a_= 4.6 Hz, 1H, H-11), 3.94 (d, *^2^J*_H1’’a-H1’’b_= 14.2 Hz, 1H, H-1”a), 3.81-3.79 (m, 2H, H-2’, H-1”b), 3.22 (dd, *^3^J*_H3-H4_= 7.7 Hz, *^4^J*_H2-H4_ = 2.0 Hz, 1H, H-4), 3.14-3.09 (m, 1H, H-5’), 3.04-2.98 (m, 1H, H-4’), 2.77-2.73 (m, 1H, H-5), 2.41 (dd, *^3^J*_H3’-H4’_ = 9.4 Hz, *^3^J*_H2’-H3’_ = 2.6 Hz, 1H,H-3’), 2.40-2.35 (m, 1H, H-24b), 2.22-2.15 (m, 1H, H-24a), 2.08-2.00 (m, 1H, H-14a), 1.94-1.92 (m, 1H, H-12), 1.91-1.88 (m, 1H, H-6b), 1.85 (dd, *^2^J*_H10a-H10b_=12.4 Hz, *^3^J*_H11-10a_= 4.7 Hz, 1H, H-10a), 1.63-1.53 (m, H, H-8a; H-14b), 1.50-1.47 (m, 1H, H-8b), 1.25(d, *^3^J*_H25-H27_ = 6.1 Hz, 3H, H-27), 1.15 (d, *^3^J*_H5’-H6’_ = 6.1 Hz, 3H, H-6’), 1.14-1.06 (m, 2H, H-6a; H-10b)

^#^- determined from ^1^H-^1^H ROESY NMR

^13^C NMR (126 MHz, dmso-d_6_) δ 174.1^^^ (C-26),164.3 (C-1), 146.0 (C-2’’), 144.4 (C-3), 136.4 (C-16), 135.4 (C-22), 133.5 (C-18), 131.9 (C-20), 131.3 (C-21), 131.1 (C-19), 129.4 (C-4”a; C-4”b), 128.5 (C-23), 128.4 (C-17), 127.2 (C-3”a; C-3”b), 124.4 (C-2), 123.3 (C-5”), 97.2 (C-1’), 97.00 (C-9), 74.5 (C-15), 73.00 (C-5’), 70.9 (C-4’), 69.5 (C-25), 66.2 (C-2’), 66.0 (C-7), 65.21(C-13),65.15 (C-11), 62.4 (C-3’), 57.9 (C-5), 56.9 (C-12), 53.7 (C-4), 49.1 (C-1’’), 46.4 (C-8), 44.1 (C-10), 40.8 (C-6), 38.9* (C-24), 37.1 (C-14), 20.05 (C-27), 18.03 (C-6’).

*-overlapped, ^- determined from ^1^H-^13^C HMBC NMR

*Compound* ***6*** (1R,3S,5R,7R,8E,12R,14E,16E,18E,20E,22R,24S,25R,26S)-22-(((2S,3S,4S,5S,6R)-3,5-dihydroxy-6-methyl-4-((4-nitrobenzyl)ammonio)tetrahydro-2H-pyran-2-yl)oxy)-1,3,26-trihydroxy-12-methyl-10-oxo-6,11,28-trioxatricyclo[22.3.1.05,7]octacosa-8,14,16,18,20-pentaene-25-carboxylate, (63 mg, yield 52.4%), Elemental analysis C_42_H_52_N_2_O_15_: C, 60.00; H, 6.52; N, 3.41; ESI-MS *m/z*: [M + H]^+^ for C_42_H_2_N_2_O_15_: 800.0, [M+Na]^+^ 823.0, [M-H]^-^ 799.0; melting point : 161-163°C; FT-IR (KBr): ν(O-H) 3399 cm^-1^, ν(C-H)_ar_ 3200 cm^-1^, ν(C-H) 2978 cm^-1^, ν(N-H) 3019 cm^-1^, ν(C=O) 1714 cm^-1^, ν(C=C) 1605 cm^-1^, ν(NO_2_) 1573 cm^-1^, δ(C-OH) 1346 cm^-^1, ν(C-O-C) 1058 cm^-1^, γ(=C-H)_ar_ 840 cm^-1^

^1^H NMR (600 MHz, dmso-d_6_) δ 10.50 (H_a_-NH_2_^+^), 9.50 (H_b_-NH_2_^+^), 8.18-8.14 (m, 2H, H-3”a, H-3”b), 7.71 (d, *^3^J*_H4”a, H4”b-H3”a-H3”b_ = 8.7 Hz, 3H, H-4”a, H-4”b), 6.49 (dd, *^3^J*_H18-H19_ = 14.6 Hz, *^3^J*_H18-H17_ = 10.8 Hz, 1H, H-18), 6.26 (d, *^3^J*_H3-H4_ = 7.7 Hz, 1H, H-3), 6.21-6.16 (m, 3H, H-19, H-20, H-21), 6.15-6.03 (m, 3H , H-2, H-17, H-22), 5.90 (dd, *^3^J*_H16-H17_ = 15.3 Hz, *^3^J*_H16-H15_ = 8.8 Hz, 2H, H-16), 5.60 (ddd, *^3^J*_H22-H23_ = 15.3 Hz, *^3^J*_H23-H24a_ =9.7 Hz, *^3^J*_H23-H24b_ = 5.9 Hz, 1H, H-23), 5.22^#^ (C_9_-OH), 4.69-4.64 (m, 1H, H-25), 4.42-4.31 (m, 1H, H-1’), 4.21-4.17 (m, 1H, H-13), 4.16-4.10 (m, 1H, H-7), 4.02 (td, *^3^J*_H12-H11_=10.7 Hz, *^3^J*_H11-H10a_ = 4.6 Hz, 1H, H-11), 4.02-3.95 (m, 1H, H-1”a), 3.85 (d, *^2^J*_H1”a-H1”b_ =14.9 Hz, 1H, H-1”b), 3.79 (d, *^3^J*_H2’-H3’_ = 2.9 Hz, 1H. H-2’), 3.22 (dd, *^3^J*_H3-H4_ =7.7 Hz, *^4^J*_H2-H4_ = 2.0 Hz, 1H, H-4), 3.15-3.08 (m, 3H, H-5’), 3.08-3.02 (m, 1H, H-4’), 2.77-2.73 (m, 1H, H-5), 2.42-2.35 (m, 2H, H-24b, H-3’), 2.19-2.15 (m, 1H, H-24a), 2.15-2.08 (m, 1H, H-14a), 1.96-1.85 (m, 2H, H-6b, H-12), 1.88-1.81(m, 1H, H-10b) 1.62-1.52 (m, 3H, H-8a, H-8b, H-14b), 1.24 (d, *^3^J*_H_ = 8.1 Hz, 3H, H-27), 1.16 (d, *^3^J*_H5-‘H6’_ = 6 Hz, 3H, H-6’),1.14-1.06 (m, 2H, H-6a, H-10a)

^#^- determined from ^1^H-^1^H ROESY NMR

^13^C NMR (126 MHz, dmso-d_6_) δ ^174.6 (C-26), 164.3 (C-1), 149.6 (C-2”), 146.21 (C-5”), 144.4 (C-3), 138.63 (C-6’’), 136.4 (C-16), 135.4 (C-22), 133.5 (C-18), 131.9 (C-20), 131.2 (C-21), 131.0 (C-19), 128.9 (C-4”a, C-4”b)128.4 (C-23), 128.3(C-17), 124.4 (C-2), 123.0 (C-3”a, C-3”b), 97.26 (C-9), 96.9 (C-1’), 74.6 (C-15), 73.0 (C-5’), 71.0 (C-4’), 69.5 (C-25), 66.3(C-2’), 65.9 (C-7), 65.2 (C-11), 64.7 (C-13), 62.4 (C-3’), 57.9 (C-5), 56.9 (C-12), 53.7 (C-4), 49.1 (C-1”), 46.4 (C-8), 44.1 (C-10), 40.7 (C-6), 38.5* (C-24), 37.2 (C-14), 20.1 (C-27), 18.0 (C-6’).

*-overlapped, ^- determined from ^1^H-^13^C HMBC NMR

*Compound* ***7*** (1R,3S,5R,7R,8E,12R,14E,16E,18E,20E,22R,24S,25R,26S)-22-(((2S,3S,4S,5S,6R)-3,5-dihydroxy-4-((2-hydroxy-5-nitrobenzyl)ammonio)-6-methyltetrahydro-2H-pyran-2-yl)oxy)-1,3,26-trihydroxy-12-methyl-10-oxo-6,11,28-trioxatricyclo[22.3.1.05,7]octacosa-8,14,16,18,20-pentaene-25-carboxylate, (120 mg, yield 61.1%), Elemental analysis C_40_H_52_N_2_O_16_: C, 58.80; H, 6.38; N, 3.41; ESI-MS *m/z*: [M +Na]^+^ 837.0, [M-H]^-^ 813.0; melting point : 260-262°C with decomposition; FT-IR (KBr): ν(O-H) 3403 cm^-1^, ν(C-H)_ar_ 3200 cm^-1^, ν(C-H) 2978 cm^-1^, ν(N-H) 3021 cm^-1^, ν(C=O) 1706 cm^-1^, ν(C=C) 1604 cm^-1^, ν(NO_2_) 1523 cm^-1^, δ(C-OH) 1384 cm^-1^, ν(C-O-C) 1055 cm^-1^, γ(=C-H)_ar_ 840 cm^-1^

^1^H NMR (600 MHz, dmso-d_6_) δ 10.70 (H_a_-NH_2_^+^), 10.03 (H_b_-NH_2_^+^), 8.10-8.6 (m, 1H, H-7’’), 7.88 (dd, *^3^J*_H4’’-H5’’_ = 9.2 Hz, 1H, H-5’’), 6.54-6.50 (m, 2H, H-18; H-4’’), 6.27 (dd, *^3^J*_H2-H3_ = 15.9 Hz; *^3^J*_H3-H4_ = 7.7 Hz, 1H, H-3), 6.21-6.13 (m, 3H, H-19; H-20; H-21), 6.10-6.08 (m, 1H, H-2), 6.08- 6.06 (m, 2H, H-17; H-22), 5.88 (dd, *^3^J*_H16-H17_ = 15.3 Hz, *^3^J*_H15-H16_ = 9.0 Hz, 1H, H-16), 5.60 (ddd, *^3^J*_H23-H22_ = 15.4 Hz; *^3^J*_H23-H24a_ = 9.9 Hz; *^3^J*_H23-H24b_ = 5.8Hz, 1H, H-23), 5.32^#^ (C_9_-OH), 4.68-4.63 (m, 1H, H-25), 4.40-4.33 (m, H, H-15), 4.30 (bs, 1H, H-1’), 4.25-4.20 (m, 1H, H-13), 4.19-4.11 (m, 2H, H-7), 4.09-4.07 (m, 1H, H-11), 4.02 (d, *^2^J*_H1’’a-H1’’b_=13.5 Hz, 1H, H-1’’a), 3.81 (d, *^2^J*_H1’’a-H1’’b_=13.5 Hz, 1H, H-1’’b), 3.22 (dd, *^3^J*_H3-H4_ = 7.7Hz, 1H, H-4), 3.14-3.01 (m, 2H, H-4’; H-5’), 2.75-2.72 (m, 1H, H-5), 2.54* (H-3’), 2.42-2.35 (m, 1H, H-24b), 2.20-2.28 (m, 2H, H-24a, H-14a), 1.96-1.90 (m, 1H, H-6b), 1.88-1.79 (m, 3H, H-10a; H-12), 1.58 (dd, *^2^J*_H8a-8b_= 14.3 Hz, 1H, H-8a), 1.53-1.44 (m, 2H , H-8b; H-14b), 1.25- 1.20 (m, 3H, H-27), 1.17-1.14- (m, 5H , H-6’;H-10b; H-6a)

^#^- determined from ^1^H-^1^H ROESY NMR

^13^C NMR (126 MHz, dmso-d_6_) δ 172.63 (C-26), 164.3 (C-1), 155.0 (C-3’’), 144.4 (C-3), 138.63 (C-6’’), 135.6 (C-16), 135.3 (C-2’’), 133.4 (C-21), 133.2 (C-18), 131.8 (C-19), 131.4 (C-20), 128.6 (C-17), 128.4 (C-23), 126.6 (C-7’’), 125.7 (C-5’’), 124.4 (C-2), 117.7 (C-4’’), 97.00 (C-9), 96.3 (C-1’), 74.4 (C-15), 72.8 (C-5’), 69.5 (C-4’), 69.48 (C-25), 66.1 (C-7), 65.5 (C-11), 65.2 (C-2’), 64.9 (C-13), 60.7 (C-3’), 58.0 (C-12), 57.8 (C-5), 53.5 (C-4), 46.2 (C-8), 44.2 (C-10), 44.1 (C-1’’), 40.7 (C-6), 38.92* (C-24), 36.4 (C-14), 20.1 (C-27), 17.8 (C-6’).

*-overlapped

^15^N NMR (28 kHz, dmso-d_6_ with traces of water) δ 344.3* (N-mycosamine)

*-determined from ^1^H-^15^N gHMBC NMR

*Compound* ***8*** (1R,3S,5R,7R,8E,12R,14E,16E,18E,20E,22R,24S,25R,26S)-22-(((2S,3S,4S,5S,6R)-4-((5-bromo-2-hydroxybenzyl)ammonio)-3,5-dihydroxy-6-methyltetrahydro-2H-pyran-2-yl)oxy)-1,3,26-trihydroxy-12-methyl-10-oxo-6,11,28-trioxatricyclo[22.3.1.05,7]octacosa-8,14,16,18,20-pentaene-25-carboxylate,( 54.6 mg, yield 42.7%), Elemental analysis C_40_H_52_BrNO_14_: C, 56.41; H, 6.12; N, 1.60; ESI-MS *m/z*: [M+H]^+^ 848.0, [M+H]^+^ 850.0; melting point : 220-221°C with decomposition; FT-IR (KBr): ν(O-H) 3401 cm^-1^, ν(C-H)_ar_ 3200 cm^-1^, ν(C-H) 2980 cm^-1^, ν(N-H) 3019 cm^-1^, ν(C=O) 1714 cm^-1^, ν(C=C) 1657 cm^-1^, δ(C-OH) 1400 cm^-1^, ν(C-O-C) 1059 cm^-1^, γ(=C-H)_ar_ 841 cm^-1^, ν(C-Br) 626 cm^-1^

^1^H NMR (500 MHz, dmso-d_6_) δ 10.0 (H_a_-NH_2_^+^), 8.50(H_b_-NH_2_^+^), 7.31 (d, *^4^J*_H7’’-H5’’_= 2.6 Hz, 1H, H-7’’), 7.19 (dd, *^3^J*_H5’’-H4’’_= 8.56 Hz, *^4^J*_H5’’-H7’’_ = 2.6 Hz, 1H, H-5’’), 6.72 (d, *^3^J*_H4’’-H5’’)_= 8.6 Hz, 1H, H-4’’), 6.50-6.45 (m, 1H, H-18), 6.26 (dd, *^3^J*_H3-H2_= 15.8 Hz, *^3^J*_H3-H4_=7.7 Hz, 1H, H-3), 6.19-6.14 (m, 3H, H-19, H-20; H-21), 6.13-6.10 (m, 1H, H-2), 6.09-6.05 (m 2H, H-17, H-22), 5.89-5.84 (m, 1H, H-16), 5.63 (ddd, *^3^J*_H23-H22_=15.2 Hz, *^3^J*_H23-H24a_=9.6 Hz, *^3^J*_H23-H24b_= 5.9 Hz, 1H, H-23), 5.24^#^ (C_9_-OH), 4.69-4.63 (m, 1H, H-25), 4.38 (dd, *^3^J*_H15-H16_=8.8 Hz, *^3^J*_H15-H14a_= 4.2 Hz, 1H, H-15), 4.34 (bs, 1H, H-1’), 4.22-4.20 (m, 1H, H-13), 4.14-4.10 (m, 1H, H-7), 4.04-3.99 (m,1H, H-11), 3.89 (d, *^2^J*_H1’’a-H1’’b_=14.1 Hz, 1H, H-1’’a), 3.84 (d, *^3^J*_H2’-H3’_ =3.0 Hz, 1H, H-2’), 3.76 (d, *^2^J*_H1’’a-H1’’b_=14.1 Hz, 1H, H-1’’b), 3.22 (dd, *^3^J*_H3-H4_ =7.6 Hz, *^4^J*_H2-H4_ = 2.0 Hz, 1H, H-4), 3.12-3.08 (m, 1H, H-5’), 3.04-2.99 (m, 1H, H-4’), 2.76-2.73 (m, 1H, H-5), 2.46 (dd, *^3^J*_H3’-H4’_ =9.7 Hz, *^3^J*_H2’-H3’_ = 2.8 Hz, 1H, H-3’), 2.43-2.34 (m, 1H, H-24b), 2.18-2.08 (m, 2H, H-24a, H-14a), 1.98-1.89 (m, 3H, H-6b, H-12), 1.84 (dd, *^2^J*_H10a-H10b_= 12.3 Hz, *^3^J*_H10a-H11_=4.6 Hz 1H, H-10a), 1.59-1.50 (m, 3H, H-8a; H-8b; H-14b), 1.26 (d, *^3^J*_H25-H27_ =6.2 Hz, 3H, H-27), 1.16 (d, *^3^J*_H5’-H6’_ =5.9 Hz, 3H, H-6’), 1.15-1.06 (m, 2H, H-6a; H-10b)

^#^- determined from ^1^H-^1^H ROESY NMR

^13^C NMR (126 MHz, dmso-d_6_) δ 174.3 (C-26), 164.3 (C-1), 156.6 (C-3’’), 144.4 (C-3), 136.2 (C-16), 135.4 (C-22), 133.4 (C-18), 131.9 (C-20), 131.3 (C-21), 131.1 (C-7’’), 130.9 (C-19), 130.1 (C-5’’), 128.5 (C-17), 128.4 (C-23), 127.7 (C-2’’), 124.4 (C-2), 117.7 (C-4’’), 109.2 (C-6’’), 97.0 (C-9), 96.9 (C-1’), 74.4 (C-15), 73.1 (C-5’), 70.5 (C-4’), 69.5 (C-25), 66.0 (C-7), 66.0 (C-11), 65.2 (C-2’),64.9 (C-13), 61.9 (C-3’), 57.8 (C-5), 57.3 (C-12), 53.6 (C-4), 46.3 (C-1’’), 46.2 (C-8), 44.1 (C-10), 40.8 (C-6), 38.7* (C-24), 36.9 (C-14), 20.0 (C-27), 17.9 (C-6’)

*-overlapped

*Compound* ***9*** (1R,3S,5R,7R,8E,12R,14E,16E,18E,20E,22R,24S,25R,26S)-22-(((2S,3S,4S,5S,6R)-4-((4-carboxybenzyl)ammonio)-3,5-dihydroxy-6-methyltetrahydro-2H-pyran-2-yl)oxy)-1,3,26-trihydroxy-12-methyl-10-oxo-6,11,28-trioxatricyclo[22.3.1.05,7]octacosa-8,14,16,18,20-pentaene-25-carboxylate,( 50 mg, yield 41.6%), Elemental analysis C_41_H_53_NO_15_: C, 61.52; H, 6.63; N, 1.71; ESI-MS *m/z*: [M + Na]^+^ 822.0, [M -H]^-^ 799.0; melting point : 168-170°C; FT-IR (KBr): ν(O-H) 3413 cm^-1^, ν(C-H)_ar_ 3200 cm^-1^, ν(C-H) 2980 cm^-1^, ν(N-H) 3017 cm^-1^, ν(C=O) 1711 cm^-1^, ν(C=C) 1615 cm^-1^, δ(C-OH) 1423 cm^-1^, ν(C-O) 1275 cm^-1^, ν(C-O-C) 1058 cm^-1^, γ(=C-H)_ar_ 842 cm^-1^

^1^H NMR (600 MHz, dmso-d_6_) δ 10.09 (H_a_-NH_2_^+^), 9.20 (H_b_-NH_2_^+^), 7.89 (dd, *^3^J*_H4”b-H3”b, H3”a-H4”a_ = 8.0 Hz, 5H, H-4”a; H-4”b), 7.53 (d, *^3^J* _H4”b-H3”b, H3”a-H4”a_ = 8.0 Hz, 1H, H-3”a; H-3”b), 6.49 (dd, *^3^J*_H18-H19_=14.6 Hz, *^3^J*_H18-H17_=10.8 Hz, 1H, H-18), 6.27 (dd, *^3^J*_H2-H3_=15.8 Hz, *^3^J*_H3-H4_=7.8 Hz, 1H, H-3), 6.22-6.16 (m, 3H, H-19, H-21, H-20), 6.12-6.06 (m, 3H, H-2; H-17, H-22), 5.88 (dd, *^3^J*_H16-H17_ = 15.3 Hz, *^3^J*_H15-H16_= 8.7 Hz, 1H, H-16), 5.61 (ddd, *^3^J*_H23-H22_ = 15.5 Hz, *^3^J*_H23-H24a_ = 10.7 Hz, ,*^3^J*_H23-H24b_= 5.9 Hz, 1H, H-23), 5.26^#^ (C_9_-OH), 4.69-4.62 (m, 1H, H-25), 4.38 (dd, *^3^J*_H15-H16_= 8.7 Hz, *^3^J*_H15-H14a_ = 4.7 Hz, 1H, H-15), 4.31 (bs, 1H, H-1’), 4.26-4.21 (m, 1H, H-13), 4.14-4.10 (m, 1H, H-7), 4.05 (td, *^3^J*_H11-H12_ = 10.8 Hz, *^3^J*_H10a-H11_=4.6 Hz, 1H, H-11), 3.95 (d, *^2^J*_H1’’a-H1’’b_= 14.0 Hz, 1H, H-1’’a), 3.79-3.78 (m, 2H, H-2’; H-1’’b), 3.22 (dd, *^3^J*_H3-H4_ = 7.7 Hz, *^4^J*_H2-H4_ = 2.0 Hz 2H, H-4), 3.12-3.08 (m, 1H, H-5’), 3.07-3.00 (m, 1H, H-4’), 2.76-2.73 (m, 1H, H-5), 2.46-2.42 (m, 1H, H-3’), 2.41-2.35 (m, 1H, H-24b), 2.19-2.11 (m, 1H, H-24a), 2.04-1.99 (m, 1H, H-14a), 1.95-1.92 (m, 2H, H-6b; H-12), 1.86 (dd, *^2^J*_H10a-H10b_= 12.4 Hz, *^3^J*_H10a-H11_=4.6 Hz, 1H, H-10a), 1.64-1.56 (m, 4H, H-14b, H-8a), 1.52 (dd, *^2^J*_H8a-H8b_= 14.3 Hz, *^3^J*_H8b-H7_ =2.0 Hz, 1H, H-8b), 1.26 (d, *^3^J*_H25-H27_ = 6.1 Hz, 3H, H-27), 1.16 (d, *^3^J*_H5’-H6’_ = 5.8 Hz, 3H, H-6’), 1.15-1.11 (m, 2H, H-10b)

^#^- determined from ^1^H-^1^H ROESY NMR

^13^C NMR (126 MHz, dmso-d_6_) δ 173.8 (C-26), 167.2 (C-6”), 164.3 (C-2), 147.5 (C-2”), 144.4 (C-3), 136.1 (C-16), 135.3 (C-22), 133.3 (C-18), 131.8 (C-20), 131.3 (C-21), 131.2 (C-19), 129.1 (C-4”a; C-4”b), 129.0 (C-5’’), 128.4 (C-23), 128.0 (C-17; C-3”a; C-3”b), 124.4 (C-2), 97.1 (C-1’), 96.9 (C-9), 74.2 (C-15), 72.9 (C-5’), 70.7 (C-4’), 69.5 (C-25), 66.1 (C-2’), 66.0 (C-7), 65.2 (C-13), 65.0 (C-11), 62.4 (C-3’), 57.9 (C-5), 57.2 (C-12), 53.7 (C-4), 49.4 (C-1’’), 46.2 (C-8), 44.1 (C-10), 40.7 (C-6), 38.8* (C-24), 36.8 (C-14), 20.0 (C-27), 17.9 (C-6’).

*-overlapped

*Compound* ***10*** (1R,3S,5R,7R,8E,12R,14E,16E,18E,20E,22R,24S,25R,26S)-22-(((2S,3S,4S,5S,6R)-4-((3-carboxybenzyl)ammonio)-3,5-dihydroxy-6-methyltetrahydro-2H-pyran-2-yl)oxy)-1,3,26-trihydroxy-12-methyl-10-oxo-6,11,28-trioxatricyclo[22.3.1.05,7]octacosa-8,14,16,18,20-pentaene-25-carboxylate (51.3 mg, yield 42.7%), Elemental analysis C_41_H_53_NO_15_+C_6_H_15_N (C_47_H_68_N_2_O_15_): C, 62.60; H, 7.55; N, 3.07; ESI-MS *m/z* [M + Na]^+^ 822.0, [M-H]^-^ 799.1; FT-IR (KBr): ): ν(O-H) 3393 cm^-1^, ν(C-H)_ar_ 3200 cm^-1^, ν(C-H) 2978 cm^-1^, ν(N-H) 3017 cm^-1^, ν(C=O) 1710 cm^-1^, ν(COO^-^) 1595 cm^-1^, ν(C=C) 1564 cm^-1^, δ(C-OH) 1392 cm^-1^, ν(C-O) 1299 cm^-1^, ν(C-O-C) 1060 cm^-1^, γ(=C-H)_ar_ 842 cm^-1^

^1^H NMR (600 MHz, dmso-d_6_) δ 10.03 (H_a_-NH_2_^+^), 9.52 (H_b_-NH_2_^+^), 8.06-8.02 (m, 1H, H-3’’), 7.88-7.78 (m, 2H , H-5’’), 7.42-7.37 (m, 1H, H-7’’), 7.29-7.23 (m, 1H, H-6’’), 6.45 (dd, *^3^J*_H18-H19_=14.6; *^3^J*_H18-H17_=10.9 Hz, 1H, H-18), 6.27 (dd, *^3^J*_H2-H3_= 15.8 Hz, *^3^J*_H3-H4_= 7.7 Hz, 1H, H-3), 6.17-6.12 (m, 4H, H-19; H-20; H-21; H-2), 6.05-6.00 (m, 2H , H-17; H-22), 5.91-5.88 (m, 1H,H-16), 5.59 (ddd, *^3^J*_H22-H23_=15.3 Hz, *^3^J*_H23-H24a_= 9.2 Hz, *^3^J*_H23-H24b_= 6.2 Hz, 1H, H-23), 5.07^#^ (C_9_-OH), 4.71-4.66 (m, 1H, H-25), 4.37 (bs, 1H, H-1’), 4.30- 4.27 (m,1H, H-15), 4.24-4.20 (m, 1H, H-13), 4.11-4.07 (m, 1H, H-7), 4.04-3.99 (m, 1H, H-11), 3.88-3.72 (m, 3H, H-2’; H1’’a; H1’’b), 3.22 (dd, *^3^J*_H3-H4_= 7.7 Hz, *^4^J*_H2-H4_ = 2.0 Hz, 1H, H-4); 3.06-3.04 (m, 1H,H-5’), 2.98-2.95 (m, 1H, H-4’), 2.93 (q, *^3^J*_H1TEA-H2TEA_ =7.2Hz, 1H, H-1_TEA_), 2.78-2.74 (m, 1H, H-5), 2.41-2.33 (m, 2H, H-24b, H-3’), 2.23-2.14 (m, 2H, H-24a; H-14a), 1.92-1.90 (m, 1H, H-6b), 1.90-1.85 (m,1H, H-12), 1.84-1.80 (m, 1H, H-10a), 1.66-1.55 (m, 2H, H-14b, H-8a) 1.51-1.48 (m, 1H, H-8b), 1.25-1.19 (m, 3H, H-27), 1.19-1.07 (m, 5H, H-6a; H-10b, H-6’, H-2_TEA_)

^#^- determined from ^1^H-^1^H ROESY NMR

^13^C NMR (126 MHz, dmso-d_6_) δ 177.6 (C-8”), 172.2 (C-26), 164.3 (C-1), 144.5 (C-3), 139.9 (C-2”), 139.0 (C-4”), 137.3 (C-16), 135.4 (C-22), 133.7 (C-18), 132.0 (C-19), 131.0 (C-20), 130.6 (C-21), 130.1 (C-3’’), 129.9 (C-7’’), 129.8 (C-4”), 128.2 (C-23), 127.8 (C-17), 127.6 (C-5’’), 127.33 (C-6’’), 124.4 (C-2), 98.3 (C-1’), 96.8 (C-9), 75.6 (C-15), 72.7 (C-5’), 70.8 (C-4’), 69.4 (C-25), 66.3 (C-2’), 65.8 (C-7), 65.5 (C-13), 64.7 (C-11), 62.4 (C-3’), 57.8 (C-5), 57.7 (C-12), 53.8 (C-4), 49.5 (C-1’’), 46.8 (C-8), 45.5 (C-1_TEA_), 43.6 (C-10), 40.7 (C-6), 38.6* (C-24), 37.9 (C-14), 19.9 (C-27), 18.0 (C-6’), 9.04 (C-2_TEA_)

*-overlapped

*Compound* ***11*** (1R,3S,5R,7R,8E,12R,14E,16E,18E,20E,22R,24S,25R,26S)-22-(((2S,3S,4S,5S,6R)-3,5-dihydroxy-4-((4-(4-hydroxybut-1-yn-1-yl)benzyl)ammonio)-6-methyltetrahydro-2H-pyran-2-yl)oxy)-1,3,26-trihydroxy-12-methyl-10-oxo-6,11,28-trioxatricyclo[22.3.1.05,7]octacosa-8,14,16,18,20-pentaene-25-carboxylate, (55 mg, yield 44.4%), Elemental analysis C_44_H_57_NO_14_: C, 64.10; H, 6.91; N, 1.58; ESI-MS *m/z*: [M + Na]^+^ 846.0, [M-H]^-^ 822.0; melting point : 150-152°C; FT-IR (KBr): ν(O-H) 3393 cm^-1^, ν(C-H)_ar_ 3200 cm^-1^, ν(N-H) 3017 cm^-1^, ν(C-H) 2976 cm^-1^, ν(C ≡C) 2270 cm^-1^, ν(C=O) 1711 cm^-1^, ν(C=C) 1651 cm^-1^, δ(C-OH) 1400 cm^-1^, ν(C-O) 1276 cm^-1^, ν(C-O-C) 1058 cm^-1^, γ(=C-H)_ar_ 841 cm^-1^

^1^H NMR (500 MHz, dmso-d_6_) δ 10.00 (H_a_-NH_2_^+^), 9.42 (H_b_-NH_2_^+^), 7.39-7.33 (m, 2H, H-4”a, H-4”b), 7.34-7.31 (m, 3H, H-3”a, H-3”b), 6.49 (dd, *^3^J*_H18-H19_ =14.7 Hz, *^3^J*_H18-H17_ = 10.7 Hz, 1H, H-18), 6.27 (dd, *^3^J*_H2-H3_ = 15.8 Hz, *^3^J*_H3-H4_ = 7.7 Hz, 1H, H-3), 6.21-6.16 (m, 3H, H-19, H-21, H-20), 6.13-6.10 (m, 1H, H-2), 6.09-6.03 (m, 2H, H-17, H-22) 5.89 (dd, *^3^J*_H16-H17_ = 15.3 Hz, *^3^J*_H16-H15_ = 8.7 Hz, 1H, H-16), 5.60 ( ddd, *^3^J*_H23-H22_ = 15.2 Hz, *^3^J*_H23-H24a_ =9.7 Hz, *^3^J*_H23-H24b_ =5.9 Hz, 1H, H-23), 5.23^#^ (C_9_-OH), 4.70-4.65 (m, 1H, H-25), 4.40-4.34 (m, 1H , H-15), 4.33 (bs, 1H, H-1’), 4.26-4.19 (m, 1H, H-13), 4.17-4.10 (m,1H , H-7), 4.02 (td, *^3^J*_H11-H12_ = 10.8 Hz, *^3^J*_H11-H10a_ = 4.6 Hz, 1H, H-11), 3.85 (d, *^2^J*_H1’’a-H1’’b_ = 13.7 Hz, 1H, H-1’’a), 3.77 (d, *^3^J*_H2’-H3’_ = 3.1 Hz, 1H, H-2’), 3.66 (d, *^2^J*_H1’’a-H1’’b_ = 13.7 Hz, 1H, H-1’’b), 3.58 (t, *^3^J*_H8”-H9”_ = 6.9 Hz, 2H, H-9”), 3.22 (dd, *^3^J*_H3-H4_ = 7.7 Hz, *^4^J*_H2-H4_ = 2.0 Hz, 2H, H-4), 3.14-3.09 (m, 1H , H-5’), 3.02-2.97 (m, 1H, H-4’), 2.76-2.73 (m, 1H, H-5), 2.54 (t, *^3^J*_H8”-H9”_ = 6.9 Hz 2H, H-8”), 2.42- 2.38 (m, 2H , H-3’, H-24b), 2.25-2.14 (m, 1H , H-24a), 2.05-2.00 (m, 1H, H-14a), 1.98-1.88 (m, 2H , H-6b, H-12), 1.85 (dd, *^2^J*_H10a-H10b_= 12.4 Hz, *^3^J*_H10a-H11_=4.6 Hz ,1H, H-10a), 1.60-1.58 (m, 1H, H-8a), 1.58-1.48 (m, 2H, H-14b, H-8b), 1.26 (d, *^3^J*_H25-H27_ = 6.2 Hz, 3H, H-27) 1.20-1.07 (m, 5H, H-6a, H-10b, H-6’),

^#^- determined from ^1^H-^1^H ROESY NMR

^13^C NMR (126 MHz, dmso-d_6_) δ 174.0 (C-26), 164.3 (C-1), 144.4 (C-3), 140.9 (C-2”), 136.3 (C-16), 135.4 (C-22), 133.4 (C-18), 131.9 (C-21), 131.3 (C-20), 131.0 (C-3”a, C-3”b), 130.9 (C-19), 128.4 (C-4”a, C-4”b), 128.3 (C-23), 127.9 (C-17), 124.4 (C-2), 121.3 (C-5”), 97.2 (C-1’), 97.0 (C-9), 87.8 (C-7”), 81.0 (C-6”), 74.3 (C-15), 73.00 (C-5’), 70.9 (C-4’), 69.5 (C-25), 66.1 (C-2’), 66.00 (C-7), 65.1 (C-11), 64.7 (C-13), 62.4 (C-3’), 59.7 (C-9”), 57.9 (C-5), 57.1 (C-12), 53.7 (C-4), 49.4 (C-1”), 46.3 (C-8), 44.1 (C-10), 40.8 (C-6), 38.6* (C-24), 36.9 (C-14), 23.2 (C-8”), 20.0 (C-27), 18.0 (C-6’).

*- overlapped

*Compound* ***12*** (1R,3S,5R,7R,8E,12R,14E,16E,18E,20E,22R,24S,25R,26S)-22-(((2S,3S,4S,5S,6R)-3,5-dihydroxy-4-(((4'-methoxy-[1,1'-biphenyl]-4-yl)methyl)ammonio)-6-methyltetrahydro-2H-pyran-2-yl)oxy)-1,3,26-trihydroxy-12-methyl-10-oxo-6,11,28-trioxatricyclo[22.3.1.05,7]octacosa-8,14,16,18,20-pentaene-25-carboxylate, (93 mg, yield 71.8%), Elemental analysis C_47_H_59_NO_14_: C, 65.45; H, 6.80; N, 1.59; ESI-MS *m/z*: [M + H]^+^ 862.0, [M+Na]^+^ 885.0, [M - H]^-^ 860.0; FT-IR (KBr): ν(O-H) 3416 cm^-1^, ν(C-H)_ar_ 3200 cm^-1^, ν(N-H) 3019 cm^-1^, ν(C-H) 2976 cm^-1^, ν(C=O) 1714 cm^-1^, ν(C=C) 1660 cm^-1^, δ(C-OH) 1376 cm^-1^, ν(C-O) 1275 cm^-1^, ν(C-O-C) 1060 cm^-1^, γ(=C-H)_ar_ 844 cm^-1^

^1^H NMR (600 MHz, dmso-d_6_) δ 10.30 (H_a_-NH_2_^+^), 9.55 (H_b_-NH_2_^+^), 7.61-7.57 (m, 3H, H-7” a, H-7”b), 7.57-7.53 (m, 3H,H-4”a, H-4”b) 7.44 (d, *^3^J*_H3”a,H3”b-H4”a,4”b_ = 8.2 Hz, 3H, H-3”a, H-3”b), 7.01-6.09 (m, 3H, H-8”a, H-8”b), 6.49 (dd, *^3^J*_H18-H19_ =14.5 Hz, *^3^J*_H18-H17_ = 10.8 Hz, 1H, H-18), 6.27 (dd, *^3^J*_H2-H3_ =,15.8 Hz, *^3^J*_H3-H4_ = 7.7 Hz 1H, H-3), 6.24-6.16 (m, 3H, H-19, H-21, H-20), 6.13-6.10 (m, 2H, H-2,H-17), 6.07-6.03 (m, 2H, H-22), 5.89 (dd, *^3^J*_H16-H17_ = 15.3 Hz, *^3^J*_H16-H15_ = 8.8 Hz, 1H, H-16), 5.61 ( ddd, *^3^J*_H23-H22_ = 15.3 Hz, *^3^J*_H23-H24a_=9.6 Hz, *^3^J*_H23-H24b_ =5.9 Hz, 1H, H-23), 5.23^#^ (C_9_-OH), 4.70-4.65 (m, 1H, H-25), 4.41-4.37 (m, 1H, H-15), 4.36 (bs, 1H, H-1’), 4.23-4.20 (m, 1H, H-13), 4.14-4.10 (m, 1H, H-7), 4.03 (td, *^3^J*_H11-H12_ = 10.8 Hz, *^3^J*_H11-10a_ = 4.7 Hz, 1H, H-11), 3.88 (d, *^2^J*_H1’’a-H1’’b_ = 13.4 Hz, 1H, H-1’’a), 3.81 (d, *^3^J*_H2’-H3’_ = 3.2 Hz, 1H, H-2’), 3.80 (s, 3H, H-10”), 3.69 (d, *^2^J*_H1’’a-H1’’b_ = 13.4 Hz, 1H, H-1’’b), 3.22 (dd, *^3^J*_H3-H4_ = 7.7 Hz, *^4^J*_H2-H4_ = 2.0 Hz, 1H, H-4), 3.13 (dq, *^3^J*_H4’-H5’_ = 9.2 Hz,*^3^J*_H5’-H6’_ = 6 Hz, 1H, H-5’), 3.05-3.00 (m,1H, H-4’), 2.77-2.73 (m, 1H, H-5), 2.44 (dd, *^3^J*_H3’-H4’_ = 9.7, *^3^J*_H2’-H3’_ = 3.0 Hz, 1H, H-3’), 2.41-2.35 (m, 1H, H-24b), 2.20-2.15 (m, 1H, H-24a), 2.07-2.00 (m, 1H. H-14a), 1.97-1.89 (m, 3H, H-6b ,H-12), 1.85 (dd, *^2^J*_H10a-H10b_= 12.3 Hz, *^3^J*_H10a-H11_=4.7 Hz 1H, H-10a), 1.65-1.53 (m, 2H, H-14b, H-8a), 1.51 (dd, *^2^J*_H8a-H-8b_= 14.2 Hz, *^3^J*_H8b-H7_ =2.0 Hz, 1H, H-8b), 1.26 (d, *^3^J*_H25-H27_ = 6.3 Hz, 3H, H-27), 1.22-1.16 (m, 2H, H-6a), 1.16 (d, *^3^J*_H5’-H6’_=6.0Hz, 3H, H-6’), 1.14- 1.10 (m, 1H, H-10b),

^#^- determined from ^1^H-^1^H ROESY NMR

^13^C NMR (126 MHz, dmso-d_6_) δ 174.1 (C-26), 164.3 (C-1), 158.7 (C-9”), 144.4 (C-3), 139.3 (C-2”), 138.1 (C-6”), 136.4 (C-16), 135.4 (C-22), 133.4 (C-18), 132.4 (C-5”), 131.9 (C-21), 131.3 (C-19), 131.0 (C-20), 128.4 (C-23, C-3”a, C-3”b), 128.3 (C-17), 127.5 (C-7”a, C-7”b), 125.8 (C-4”a, C-4”b), 124.4 (C-2), 114.3 (C-8”a, C-8”b), 97.2 (C-9), 97.0 (C-1’), 74.3 (C-15), 73.0 (C-5’), 70.9 (C-4’), 69.5 (C-25), 66.00 (C-7), 65.9 (C-2’), 65.2 (C-11), 65.1 (C-13), 62.4 (C-3’), 57.8 (C-5), 57.1 (C-12), 55.1 (C-10”), 53.7 (C-4), 49.3 (C-1”), 46.3 (C-8), 44.1 (C-10), 40.7 (C-6), 38.7* (C-24), 37.1 (C-14), 20.1 (C-27), 18.0 (C-6’).

*-overlapped

*Compound* ***13*** (1R,3S,5R,7R,8E,12R,14E,16E,18E,20E,22R,24S,25R,26S)-22-(((2S,3S,4S,5S,6R)-3,5-dihydroxy-6-methyl-4-((4-propylbenzyl)ammonio)tetrahydro-2H-pyran-2-yl)oxy)-1,3,26-trihydroxy-12-methyl-10-oxo-6,11,28-trioxatricyclo[22.3.1.05,7]octacosa-8,14,16,18,20-pentaene-25-carboxylate, (75 mg, yield 62.6%), Elemental analysis C_43_H_59_NO_13_: C, 64.71; H, 7.41; N, 1.70; ESI-MS *m/z*: [M + H]^+^ 798.0, [M + Na]^+^ 821.0, [M - H]^-^ for C_43_H_59_NO_13_: 796.0; melting point : 155-157°C; FT-IR (KBr): ): ν(O-H) 3367 cm^-1^, ν(C-H)_ar_ 3200 cm^-1^, ν(C-H) 2986 cm^-1^, ν(N-H) 2942 cm^-1^, ν(C=O) 1719 cm^-1^, ν(C=O)_lactone_ 1706 cm^-1^, ν(C=C) 1660 cm^-1^, δ(C-OH) 1400 cm^-1^, ν(C-O-C) 1233 cm^-1^, ν(C-O) 1188 cm^-1^, γ(=C-H)_ar_ 844 cm^-1^

^1^H NMR (500 MHz, dmso-d_6_) δ 9.96 (H_a_-NH_2_^+^), 9.54 (H_b_-NH_2_^+^), 7.28 (d, *^3^J*_H3”a-4”a, H4”b-H-3”b_ = 7.7 Hz, 2H, H-3”a, H-3”b), 7.12 (d, *^3^J*_H3”a-4”a, H4”b-H-3”b_ = 7.7 Hz, 2H, H-4”a, H-4”b), δ 6.49 (dd, *^3^J*_H19-H19_ =14.6 Hz, *^3^J*_H18-H17_ = 10.0 Hz, 1H, H-18), 6.26 (dd, *^3^J*_H3-H2_= 15.8 Hz, *^3^J*_H3-H4_=7.7 Hz, 1H, H-3), 6.24- 6.16(m, 3H, H-19, H-21, H-20), 6.13-6.10 (m,2H, H-2, H-17), 6.08-6.02 (m,2H, H-22), 5.89 (dd, *^3^J*_H16-H17_ = 15.3 Hz, *^3^J*_H16-H15_ = 8.7 Hz, 1H, H-16), 5.61 ( ddd, *^3^J*_H23-H22_ = 15.2 Hz, *^3^J*_H23-H24a_ =9.6 Hz, *^3^J*_H23-H24b_ =5.8 Hz, 1H, H-23), 5.24^#^ (C_9_-OH), 4.73-4.65 (m,1H, H-25), 4.38-4.35 (m, 1H, H-15), 4.34 (bs, 1H, H-1’), 4.23-4.20 (m, 1H. H-13), 4.14-4.10 (m, 1H, H-7), 4.03 (td, *^3^J*_H11-H12_= 10.8 Hz, *^3^J*_H11-H10a_= 4.7 Hz, 1H, H-11), 3.81 (d, *^2^J*_H1’’a-H1’’b_ = 13.3 Hz, 1H, H-1’’a), 3.78 (d, *^3^J*_H2’-H3’_ = 3.2 Hz, 1H, H-2’), 3.69 (d, *^2^J*_H1’’a-H1’’b_ = 13.3 Hz, 1H, H-1’’b), 3.22 (dd, *^3^J*_H3-H4_ = 7.7 Hz, *^4^J*_H2-H4_ = 2.0 Hz, 1H, H-4), 3.15-3.09 (m, 1H, H-5’), 3.05-3.00 (m, 1H, H-4’), 2.76-2.73 (m, 1H, H-5), 2.54 (d, *^3^J*_H-6”-H7”_= 7.6 Hz, 2H, H-6”), 2.42-2.34 (m, 2H, H-24b, H-3’), 2.20-2.15 (m, 1H, H-24a), 2.05-2.00 (m, 1H. H-14a), 1.98-1.88 (m, 2H, H-6b ,H-12), 1.85 (dd, *^2^J*_H10a-H10b_= 12.3 Hz, *^3^J*_H10a-H11_=4.7 Hz 1H, H-10a), 1.65-1.58 (m, 2H, H-7”, H-8a), 1.57 -1.48 (m, 2H, H-8b, H-14b), 1.26 (d, *^3^J*_H25-H27_ = 6.2 Hz, 3H, H-27), 1.15-1.13 (m, , H-10b, H-6’, H-6a ), 1.15 (d, *^3^J=* 6.0 Hz, 3H, H-6’), 0.89 (t, *^3^J*_H7”-H8”_= 7.3 Hz, 3H, H-8”)

^#^- determined from ^1^H-^1^H ROESY NMR

^13^C NMR (126 MHz, dmso-d_6_) δ 174.0 (C-26), 164.3 (C-1), 144.4 (C-3), 140.2 (C-5”), 137.9 (C-2”), 136.3 (C-16), 135.4 (C-22), 133.4 (C-18), 131.9 (C-20), 131.3 (C-19), 131.1 (C-21), 128.5 (C-23), 128.4 (C-17), 127.9 (C-4”a, C-4”b), 127.8 (C-3”a, C-3”b), 124.5 (C-2), 97.2 (C-9), 97.1 (C-1’), 74.2 (C-15), 72.9 (C-5’), 70.8 (C-4’), 69.5 (C-25), 66.0 (C-2’), 65.9 (C-7), 65.2 (C-13), 65.1 (C-11), 62.5 (C-3’), 57.8 (C-5), 57.1 (C-12), 53.7 (C-4), 49.5 (C-1”), 46.3 (C-8), 44.1 (C-10), 40.8 (C-6), 38.7* (C-24), 36.9 (C-14), 36.8 (C-6”), 23.9 (C-7”), 20.1 (C-27), 18.0 (C-6’), 13.5 (C-8’’)

*-overlapped

*Compound* ***14*** (1R,3S,5R,7R,8E,12R,14E,16E,18E,20E,22R,24S,25R,26S)-22-(((2S,3S,4S,5S,6R)-3,5-dihydroxy-4-((2-(2-hydroxyethoxy)benzyl)ammonio)-6-methyltetrahydro-2H-pyran-2-yl)oxy)-1,3,26-trihydroxy-12-methyl-10-oxo-6,11,28-trioxatricyclo[22.3.1.05,7]octacosa-8,14,16,18,20-pentaene-25-carboxylate, (75 mg, yield 61.2%), Elemental analysis C_42_H_57_NO_15_: C, 61.81; H, 6.99; N, 1.64; ESI-MS *m/z*: [M + H]^+^ 816.0, [M + Na]^+^ 838.0, [M - H]^-^ 814.0; melting point : 147-152°C; FT-IR (KBr): ν(O-H) 3394 cm^-1^, ν(C-H)_ar_ 3019 cm^-1^, ν(C-H) 2977 cm^-1^, ν(N-H) 2942 cm^-1^, ν(C=O) 1714 cm^-1^, ν(C=C) 1651 cm^-1^, δ(C-OH) 1400 cm^-1^, ν(C-O) 1295 cm^-1^, ν(C-O-C) 1254 cm^-1^, γ(=C-H)ar 841 cm^-1^

^1^H NMR (600 MHz, dmso-d_6_) δ 10.46 (H_a_-NH_2_^+^), 9.54 (H_b_-NH_2_^+^), 7.36 (dd, *^3^J*_H7’’-H6’’_=7.5 Hz, *^4^J*_H5’’-H7’’_= 1.8 Hz, 1H, H-7’’), 7.21 (td, *^3^J*_H5’’-H4’’_=7.8Hz, *^4^J*_H5’’-H7’’_= 1.1 Hz, 1H, H=5”), 6.97 (dd, *^3^J*_H4’’-H5’’_=7.8Hz, *^4^J*_H4’’-H6’’_=1.1 Hz, 1H, H-4”), 6.90 (td, *^3^J*_H6’’-H7’’_=7.5Hz, *^4^J*_H6’’-H4’’_= 1.1 Hz, 1H, H-6”), 6.49 (dd, *^3^J*_H18-H19_ =14.6 Hz, *^3^J*_H18-H17_ = 10.8 Hz, 1H, H-18), 6.27 (dd, *^3^J*_H3-H2_= 15.8 Hz, *^3^J*_H3-H4_=7.7 Hz, 1H, H-3), 6.22-6.14 (m, 3H, H-19,H-20, H-21), 6.14-6.10 (m, 2H, H-17, H-2), 6.08–6.03 (m, 1H, H-22), 5.89 (dd, *^3^J*_H16-H17_ = 15.3 Hz, *^3^J*_H16-H15_ = 8.8 Hz, 1H, H-16), 5.61 ( ddd, *^3^J*_H23-H22_ = 15.4 Hz, *^3^J*_H23-H24a_ =9.7 Hz, *^3^J* _H23-H24b, H23-H21_ =5.9 Hz, 1H, H-23), 5.24^#^ (C_9_-OH), 4.70-4.64 (m, 1H, H-25), 4.34 (bs, 1H, H-1’), 4.38 (ddd, *^3^J*_H15-H16_ =8.3 Hz, *^3^J*_H15-H14a_ = 4.6 Hz, *^4^J*_H15-H17_ =2.7 Hz, 1H, H-15), 4.22-4.19 (m, 1H, H-13), 4.17-4.10 (m,1H , H-7), 4.05-4.03 (m, 3H, H-11, H-8”), 3.85 (d, *^2^J*_H1’’a-H1’’b_ = 13.3 Hz, 1H, H-1”a), 3.80 (d, *^3^J*_H2’-H3’_ = 3.1 Hz, 1H, H-2’), 3.75-3.70 (m, 1H, H-9”), 3.69 (d, *^2^J*_H1’’a-H1’’b_ = 13.3 Hz, 1H, H-1’’b), 3.22 (dd, *^3^J*_H3-H4_ = 7.7 Hz, *^4^J*_H2-H4_ = 2.0 Hz, 2H, H-4), 3.13-3.04 (m, 1H, H-5’), 3.02-2.98 (m, 1H, H-4’), 2.76-2.73 (m, 1H, H-5), 2.46 (dd, *^3^J*_H3’-H4’_= 9.6 Hz, *^4^J*_H3’-H2’_= 3.0 Hz, 1H, H-3’), 2.42-3.35 (m,1H, H-24b), 2.24-2.18 (m, 1H, H-24a), 2.03-1.99 (m, 1H, H-14a), 1.97-1.91 (m, 3H, H-6b, H-12), 1.85 (dd, *^2^J*_H10a-H10b_= 12.3 Hz, *^3^J*_H10a-H11_=4.7 Hz, 1H, H-10a), 1.60-1.55 (m, 2H, H-8a, H-14b), 1.60 (d, *^3^J*_H_ = 2.0 Hz, 2H, H-6a), 1.55-1.50 (m, 1H, H-8b), 1.26 (d, *^3^J*_H25-H27_ = 6.1 Hz, 3H, H-27), 1.15 (d, *^3^J*_H5’-H6’_ =6.1 Hz, 3H, H-6’), 1.14-1.10 (m, 3H, H-6b, H-10b,)

^#^- determined from ^1^H-^1^H ROESY NMR

^13^C NMR (126 MHz, dmso-d_6_) δ 174.1 (C-26), 164.3 (C-1), 156.64 (C-3”), 144.4 (C-3), 136.3 (C-16), 135.4 (C-22), 133.4 (C-18), 131.9 (C-20), 131.3 (C-21), 131.1 (C-19), 129.4 (C-7”), 128.5 (C-23), 128.4 (C-17, C-2”), 127.9 (C-5”), 124.5 (C-2), 120.2 (C-6”), 111.9 (C-4”), 97.2 (C-9), 97.1 (C-1’), 74.4 (C-15), 72.9 (C-5’), 70.7 (C-4’), 69.8 (C-8”), 69.5 (C-25), 66.00 (C-7), 65.7^×^ (C-2’), 65.2 (C-11), 64.7 (C-13), 62.4 (C-3’), 59.6 (C-9”), 57.1 (C-12), 53.7 (C-4), 46.3 (C-8), 45.00 (C-1”), 44.1 (C-10), 40.8 (C-6), 38.7* (C-24), 37.0 (C-14), 20.1 (C-27), 18.00 (C-6’)

*-overlapped, ×- determined from ^1^H-^13^C HSQC NMR

*Compound* ***15*** (1R,3S,5R,7R,8E,12R,14E,16E,18E,20E,22R,24S,25R,26S)-22-(((2S,3S,4S,5S,6R)-4-((3-(decyloxy)benzyl)ammonio)-3,5-dihydroxy-6-methyltetrahydro-2H-pyran-2-yl)oxy)-1,3,26-trihydroxy-12-methyl-10-oxo-6,11,28-trioxatricyclo[22.3.1.05,7]octacosa-8,14,16,18,20-pentaene-25-carboxylate, (74 mg, yield 54%), Elemental analysis C_50_H_73_NO_14_: C, 65.81; H, 8.01; N, 1.52; ESI-MS *m/z*: [M + H]^+^ 913.0, [M - H]^-^ 911.0; melting point : 147-148°C; FT-IR (KBr): ν(O-H) 3404 cm^-1^, ν(C-H)_ar_ 3017 cm^-1^, ν(C-H) 2926 cm^-1^, ν(N-H) 2853 cm^-1^, ν(C=O) 1719 cm^-1^, ν(C=C) 1650 cm^-1^, δ(C-OH) 1404 cm^-1^, ν(C-O) 1303 cm^-1^, ν(C-O-C) 1258 cm^-1^, γ(=C-H)_ar_ 842 cm^-1^

^1^H NMR (600 MHz, dmso-d_6_) δ 9.86 (H_a_-NH_2_^+^), 9.25 (H_b_-NH_2_^+^), 7.32-7.27 (m, 3H, H-5”, H7”), 6.87-6.83 (m, 3H, H-3”, H-6”), 6.50 (dd, *^3^J*_H18-H19_ =14.5 Hz, *^3^J*_H18-H17_ = 10.8 Hz, 1H, H-18), 6.27 (dd, *^3^J*_H2-H3_ = 15.8Hz, *^3^J*_H3-H4_ = 7.8 Hz, 1H, H-3), 6.24-6.14 (m, 3H, H-19, H-21, H-20), 6.12-6.07 (m, 2H, H-2, H-17)), 6.06 (d, *^3^J*_H_ = 5.1 Hz H-22), 5.93(dd, *^3^J*_H16-H17_ = 15.3 Hz, *^3^J*_H16-H15_ = 8.7 Hz, 1H, H-16), 5.60 (ddd, *^3^J*_H23-H22_ = 15.4 Hz, *^3^J*_H23-H24a_ =9.7 Hz, *^3^J*_H23-H24b_ =5.9 Hz, 1H, H-23), 5.26^#^ (C_9_-OH), 4.69-4.62 (m, 1H, H-25), 4.40-4.38 (m, 2H, H-15), 4.32 (bs, 1H, H-1’), 4.23-4.20 (m,1H, H-13), 4.14-4.09 (m, 1H, H-7), 4.04 (td, *^3^J*_H11-H12_ = 10.8Hz , *^3^J*_H11-H10a_ = 4.7 Hz 1H, H-11), 3.93 (t, *^3^J*_H8”-H_ = 6.5 Hz, 3H. H-8”), 3.83-3.77 (m, 2H, H-1”a, H-2’), 3.63 (d, *^2^J*_H1”a-H1”b_ = 12.9 Hz, 1H, H-1”b), 3.22 (dd, *^3^J*_H3-H4_ = 7.7 Hz, *^4^J*_H2-H4_ = 2.0 Hz, 1H, H-4), 3.12 (dq, *^3^J*_H4’-H5’_ = 8.9 Hz, *^3^J*_H5’-H6’_ = 6.1 Hz, 1H, H-5’), 3.04-2.99 (m, 1H, H-4’), 2.77-2.73 (m, 1H, H-5), 2.46 (dd, *^3^J*_H3’-H4’_ = 9.8 Hz, *^3^J*_H2’-H3’_ = 3.0 Hz, 1H, H-3’), 2.41-2.35 (m, 1H, H-24b), 2.19-2.14 (m, 1H, H-24a), 2.04-2.00 (m, 1H. H-14a), 1.98-1.89 (m, 2H, H-6b, H-12), 1.85 (dd, *^2^J*_H10a-H10b_= 12.4 Hz, *^3^J*_H10a-H11_=4.7 Hz, 1H, H-10a), 1.72-1.65 (m, 4H, H-8a, H-8b, H-14b, H-9”), 1.45-1.34 (m. 4H, H-10”), 1.30-1.23* (m, 24H , H-27, H-11”, H-12”, H-13”, H-14”, H-15”, H-16”), 1.22-1.16 (m, 1H, H-6a), 1.15 (d, *^3^J*_H5’-H6’_ = 6.0 Hz, 3H, H-6’),1.14-1.09 (m, 2H, H-10b), 0.86 (t, *^3^J*_H17”-H16”_= 6.9 Hz, 4H, H-17”)

^#^- determined from ^1^H-^1^H ROESY NMR

^13^C NMR (126 MHz, dmso-d_6_) δ 174.0 (C-26), 164.3 (C-1), 157.5 (C-4”), 144.4 (C-3), 136.2 (C-16), 135.4 (C-22), 133.4 (C-18), 131.9 (C-21), 131.7 (C-2”), 131.3 (C-19), 131.1 (C-20), 130.2 (C-6”), 129.3 (C-7”), 128.5 (C-23, C-17), 124.4 (C-2), 114.1 (C-3”, C-5”), 97.1 (C-9), 96.80 (C-1’) 74.3 (C-15), 72.9 (C-5’), 70.6 (C-4’), 69.5 (C-25), 67.3 (C-8”), 65.9 (C-7), 65.7 (C-2’), 65.2 (C-11), 65.1 (C-13), 62.2 (C-3’), 57.8 (C-5), 57.1 (C-12), 53.7 (C-4), 48.9 (C-1”), 46.3 (C-8), 44.1 (C-10), 40.8 (C-6), 38.7* (C-24), 37.0 (C-14), 31.1 (C-15”), 28.8 (C-12”), 28.7 (C-13”), 28.6 (C-11”, C-14”), 28.5 (C-9”), 25.4 (C-10”), 21.9 (C-9”), 20.1 (C-27), 17.9 (C-6’), 13.7 (C-17”).

*-overlapped

*Compound* ***16*** (1R,3S,5R,7R,8E,12R,14E,16E,18E,20E,22R,24S,25R,26S)-22-(((2S,3S,4S,5S,6R)-4-((2-(benzyloxy)ethyl)ammonio)-3,5-dihydroxy-6-methyltetrahydro-2H-pyran-2-yl)oxy)-1,3,26-trihydroxy-12-methyl-10-oxo-6,11,28-trioxatricyclo[22.3.1.05,7]octacosa-8,14,16,18,20-pentaene-25-carboxylate, (64.3 mg, yield 53.5%), Elemental analysis C_42_H_57_NO_14_: C, 63.00; H, 7.11; N, 1.71; ESI-MS *m/z*: [M + H]^+^ for C_42_H_57_NO_14_: 800.0, [M + Na]^+^ for C_42_H_57_NO_14_: 822.0, [M - H]^-^ for C_42_H_57_NO_14_: 798.0; melting point : 172-174°C with decomposition; FT-IR (KBr): ν(O-H) 3420 cm^-1^, ν(C-H)_ar_ 3017 cm^-1^, ν(C-H) 2928 cm^-1^, ν(N-H) 2853 cm^-1^, ν(C=O) 1720 cm^-1^, ν(C=C) 1639 cm^-1^, δ(C-OH) 1378 cm^-1^, ν(C-O) 1299 cm^-1^, ν(C-O-C) 1263 cm^-1^, γ(=C-H)_ar_ 845 cm^-1^

^1^H NMR (600 MHz, dmso-d_6_) δ 10.02 (H_a_-NH_2_^+^), 9.50 (H_b_-NH_2_^+^), 7.93-7.85 (m, 2H, H-6”a, H-6”b), 7.64-7.51 (m, 1H, H-7”), 7.38-7.24 (m, 2H, H-5”a, H-5”b), 6.49 (dd, *^3^J*_H18-H19_ = 14.7 Hz, *^3^J*_H17-H18_ = 10.7 Hz, 1H, H-18), 6.27 (dd, *^3^J*_H2-H3_= 15.8 Hz, *^3^J*_H3-H4_ = 7.7 Hz, 1H, H-3), 6.23-6.12 (m, 3H, H-19, H-20, H-21), 6.12-6.11 (m, 1H, H-2), 6.11-6.02 (m, 2H, H-17, H-22), 5.88-5.80 (m, 1H, H-16), 5.64–5.57 (m, 1H, H-23), 5.27^#^ (C_9_-OH), 4.70-4.65 (m, 1H, H-25), 4.38-4.33 (m, 1H, H-15), 4.32-4.30 (m, 1H, H-1’), 4.52 – 4.48 (m, 1H, H-3”a, H-3”b), 4.43–4.34 (m, 1H, H-2”a, H-2”b), 4.24-4.20 (m, 1H, H-13), 4.17-4.10 (m, 1H, H-7), 4.10–3.99 (m, 2H, H-11, H-1”a), 3.93–3.85 (m, 1H, H-1”b), 3.77-3.69 (m, 1H, H-2’), 3.23 (dd, *^3^J*_H3-H4_ = 7.6 Hz, *^4^J*_H2-H4_ = 2.0 Hz, 1H, H-4), 3.18-3.11 (m, 1H, H-5’), 3.12-3.05 (m, 1H, H-4’), 2.78-2.73 (m, 1H, H-5), 2.64-2.68 (m, 1H, H-3’), 2.42-2.36 (m, 1H, H-24b), 2.24-2.15 (m, 1H, H-24a), 2.05-1.99 (m, 1H, H-14a), 2.00-1.92 ( m, 2H, H-6b, H-12), 1.89-1.83 (m, 1H, H-10a), 1.64-1.53 (m, 3H, H-8a, H-14b,H-8b), 1.26 (d, *^3^J*_H25-H27_ = 6.2 Hz, 3H, H-27), 1.21-1.10 (m, 5H,. H-6a, H-6’, H-10b),

^#^- determined from ^1^H-^1^H ROESY NMR

^13^C NMR (126 MHz, dmso-d_6_) δ 164.3 (C-1), 144.3 (C-3), 138.3 (C-4”), 135. 7 (C-16), 135.3 (C-22), 133.3 (C-18), 131.8 (C-21), 131.3 (C-20), 128.5 (C-23), 128.1 (C-17), 127.5 (C-5”a, C-5”b), 127.4 (C-6”a, C-6”b), 126.8 (C-7”), 124.5 (C-2), 97.1 (C-9), 96.8 (C-1’), 74.2 (C-15), 73.00 (C-5’), 71.9 (C-3”), 70.7 (C-2”), 69.7 (C-4’), 69.5 (C-25), 66.0 (C-7),65.7 (C-2’), 65.1 (C-11), 65.00 (C-13), 62.3 (C-3’), 57.9 (C-5), 57.1 (C-12), 53.6 (C-4), 49.7 (C-1”), 46.2 (C-8), 44.1 (C-10), 40.8 (C-6), 38.79* (C-24) 36.5 (C-14), 20.1 (C-27), 17.8 (C-6’)

*-overlapped

*Compound* ***17*** (1R,3S,5R,7R,8E,12R,14E,16E,18E,20E,22R,24S,25R,26S)-22-(((2S,3S,4S,5S,6R)-3,5-dihydroxy-6-methyl-4-((naphthalen-2-ylmethyl)ammonio)tetrahydro-2H-pyran-2-yl)oxy)-1,3,26-trihydroxy-12-methyl-10-oxo-6,11,28-trioxatricyclo[22.3.1.05,7]octacosa-8,14,16,18,20-pentaene-25-carboxylate,( 57.6 mg, yield 46.8%), Elemental analysis C_44_H_55_NO_13_: C, 65.52; H, 6.84; N, 1.70; ESI-MS *m/z*: [M + H]^+^ 806.0, [M - H]^-^ 804.0; melting point : 170-171°C with decomposition FT-IR (KBr): ν(O-H) 3380 cm^-1^, ν(C-H)_ar_ 2980 cm^-1^, ν(C-H) 2946 cm^-1^, ν(N-H) 2835 cm^-1^, ν(C=O) 1710 cm^-1^, ν(C=C) 1587 cm^-1^, δ(C-OH) 1408 cm^-1^, ν(C-O-C) 1265 cm^-1^, ν(C-O) 1028 cm^-1^,γ(=C-H)_ar_ 844 cm^-1^

^1^H NMR (600 MHz, dmso-d_6_) δ 10.16 (H_a_-NH_2_^+^), 9.51 (H_b_-NH_2_^+^), 7.92-7.83 (m, 6H, H-3”, H5”, H8”, H-10”), 7.57 (dd, *^3^J*_H_ = 8.4 Hz, *^3^J*_H_ = 1.8 Hz, 2H, H-11”), 7.50-7.44 (m, 3H, H-6”, H-7”), 6.50 (dd, *^3^J*_H18-H19_ = 14.7 Hz, *^3^J*_H17-H18_ = 10.8 Hz, 1H, H-18), 6.27 (dd, *^3^J*_H2-H3_ = 15.8 Hz, *^3^J*_H3-H4_ = 7.7 Hz, 1H, H-3), 6.21-6.16 (m, 3H, H-19, H-20, H-21), 6.12-6.10 (d, *^3^J*_H_ = Hz, 1H, H-2), 6.10–6.04 (m, 2H, H-17, H-22), 5.89 (dd, *^3^J*_H16-H17_ = 15.3 Hz, *^3^J*_H_ = Hz, 1H, H-16), 5.60 (ddd, *^3^J*_H23-H22_ = 15.3 Hz, *^3^J*_H23-H24a_ = 9.7 Hz, 1H, H-23), 5.26^#^ (C_9_-OH), 4.69-4.62 (m, 1H, H-25) 4.41 – 4.36 (m, 2H, H-15, H-1’), 4.24-4.20 (m, 1H, H-13), 4.14-4.09 (m, 1H, H-7), 4.07-4.03 (m, 2H, H-11, H-1”a), 3.88-3.81 (m, 2H, H-2’, H-1”b), 3.22 (dd, *^3^J*_H3-H4_ = 7.7 Hz, *^4^J*_H2-H4_ = 2.0 Hz, 1H, H-4), 3.13 (dq, *^3^J*_H4’-H5’)_ = 9.0 Hz, *^3^J*_H5’-H6’_ = 6.0 Hz, 1H, H-5’), 3.07-3.01 (m, 1H, H-4’), 2.77-2.73 (m, 1H, H-5), 2.50^ (H-3’), 2.41-2.35 (m, 1H, H-24b) 2.19-2.13 (m, 1H, H-24a), 2.05-2.00 (m, 1H, H-14a), 1.98-1.89 (m, 2H, H-12, H-6b), 1.86 (dd, *^2^J*_H10a-H10b_ = 12.6 Hz, *^3^J*_H10a-H11_ =4.7 Hz, 1H, H-10a), 1.64-1.54 (m, 2H, H-8a, H-14b), 1.51 (dd, *^2^J*_H8a-H8b_ = 14.2 Hz, *^3^J*_H_ =2.0 Hz , 1H, H-8b), 1.25 (d, *^3^J*_H25-H27_ = 6.9 Hz, 3H, H-27), 1.21-1.18 (m, 1H, H-6a), 1.16 (d, *^3^J*_H5’-H6’_ = 6.0 Hz, 3H, H-6’) 1.15-1.09 (m, 1H, H-10b),

^-overlapped, ^#^- determined from ^1^H-^1^H ROESY NMR

^13^C NMR (126 MHz, dmso-d_6_) δ 174.1 (C-26), 164.3 (C-1), 144.4 (C-3), 138.3 (C-2”), 136.3 (C-16), 135.4 (C-22), 133.4 (C-18), 132.9 (C-20, C-4”, C-9”), 132.0 (C-21), 131.9 (C-19), 131.295, 131.110, 128.5 (C-23), 128.2 (C-17) 127.4 (C-3”), 127.3 (C-10”), 126.7 (C-11”), 125.9 (C-5”), 125.8 (C-8”), 125.3 (C-6”, C-7”), 124.4 (C-2), 97.2 (C-9), 97.1 (C-1’), 74.3 (C-15), 73.0 (C-5’), 70.8 (C-4’), 69.5 (C-25), 66.1 (C-2’), 65.9 (C-7), 65.2 (C-11), 65.1 (C-13), 62.5 (C-3’), 57.9 (C-5), 57.1 (C-12), 53.7 (C-4), 49.8 (C-1”), 46.3 (C-8), 44.1 (C-10), 40.8 (C-6), 38.7* (C-24), 37.0 (C-14), 20.1 (C-27), 18.0 (C-6’).

*-overlapped

*Compound* ***18*** (1R,3S,5R,7R,8E,12R,14E,16E,18E,20E,22R,24S,25R,26S)-22-(((2S,3S,4S,5S,6R)-4-((anthracen-9-ylmethyl)ammonio)-3,5-dihydroxy-6-methyltetrahydro-2H-pyran-2-yl)oxy)-1,3,26-trihydroxy-12-methyl-10-oxo-6,11,28-trioxatricyclo[22.3.1.05,7]octacosa-8,14,16,18,20-pentaene-25-carboxylate, (88 mg, yield 68.4%), Elemental analysis C_48_H_57_NO_13_: C, 67.31; H, 6.69; N, 1.60; ESI-MS *m/z*: [M + H]^+^ 856.0, [M + Na]^+^ 878.0, [M - H]^-^ 854.0; melting point : 165-166°C; FT-IR (KBr): ν(O-H) 3459 cm^-1^, ν(C-H)_ar_ 3049 cm^-1^, ν(C-H) 3015 cm^-1^, ν(N-H) 2978 cm^-1^, ν(C=O) 1716 cm^-1^, ν(C=C) 1660 cm^-1^, δ(C-OH) 1400 cm^-1^, ν(C-O-C) 1265 cm^-1^, ν(C-O) 1060 cm^-1^, γ(=C-H)_ar_ 845 cm^-1^

^1^H NMR (600 MHz, dmso-d_6_) δ 10.13 (H_a_-NH_2_^+^), 9.50 (H_b_-NH_2_^+^), 8.54 (s, 1H, H-9”), 8.47 (d, *^3^J*_H4”a,H4”b-H5”a,H5”b_ = 8.9 Hz, 3H, H-4”a, H-4”b) 8.08 (dt, *^3^J*_H7”a,H7”b-H6”a,H6”b_ =8.9 Hz, 4H, H-7”a, H-7”b), 7.54-7.49 (m, 3H, H-5”a, H-5”b), 7.54-7.49 (m, 3H, H-6”a, H-6”b), 6.53 (dd, *^3^J*_H18-H19_ =14.6 Hz, *^3^J*_H18-H17_ = 10.8 Hz, 1H, H-18), 6.28 (dd, *^3^J*_H2-H3_ = 15.8Hz, *^3^J*_H3-H4_ = 7.8 Hz, 1H, H-3), 6.26-6.20 (m, 2H, H-19, H-21, 6.18-6.12 (m, 2H, H-2, H-17, H-20) 6.08 (d, *^3^J*_H22-H23_ = 15.0 Hz, 2H, H-22), 5.93(dd, *^3^J*_H16-H17_ = 15.3 Hz, *^3^J*_H16-H15_ = 8.7 Hz, 1H, H-16), 5.61 (ddd, *^3^J*_H23-H22_ = 15.4 Hz, *^3^J*_H23-H24a_ =9.7 Hz, *^3^J*_H23-H24b_ =5.9 Hz, 1H, H-23), 5.31^#^ (C_9_-OH), 4.81 (d, *^2^J*_H1”a-H1”b_ = 11.9 Hz, 1H, H-1”a), 4.70-4.64 (m, 1H, H-25), 4.58 (d, *^2^J*_H1”a-H1”b_ = 11.9 Hz, 1H, H-1”b), 4.46-4.45 (m, 2H, H-1’,H-15), 4.32-4.28 (m, 1H, H-13), 4.20-4.14 (m, 1H, H-2’), 4.12- 4.06 (m, 3H, H-7, H-11), 3.23 (m, 2H, H-4, H-5’), 3.07-3.01 (m, 1H, H-4’), 2.82 (dd, *^3^J*_H3’-H4’_ = 9.8 Hz, *^3^J*_H2’-H3’_ = 3.0 Hz, 1H, H-3’), 2.78-2.73 (m, 1H, H-5), 2.41-2.36 (m, 1H, H-24b), 2.20-2.15 (m, 1H, H-24a ), 2.12-2.08 (m, 1H, H-14a), 2.01-1.94 (m, 2H, H-6b, H-12), 1.88 (dd, *^2^J*_H10a-H10b_= 12.3 Hz, *^3^J*_H10a-H11_=4.6 Hz 1H, H-10a), 1.67-1.59 (m, 3H, H-8a, H-8b, H-14b), 1.26 (d, *^3^J*_H25-H27_ =6.2 Hz, 3H, H-27), 1.18 (d, *^3^J*_H5’-H6’_ = 6.1 Hz, 3H, H-6’), 1.16-1.05 (m, 2H, H-6a, H-10b)

^#^- determined from ^1^H-^1^H ROESY NMR

^13^C NMR (126 MHz, dmso-d_6_) δ 174.0 (C-26), 164.3 (C-1), 144.4 (C-3), 136.2 (C-16), 135.4 (C-2), 133.4 (C-18), 132.1 (C-3”a, C-3”b), 131.3 (C-19), 131.2 (C-21, C-8”a, C-8”b), 131.0 (C-20), 129.8 (C-2”), 128.6 (C-23), 128.5 (C-17, C-7”a, C-7”b), 125.9 (C-9”), 125.6 (C-4”a, C-4”b), 124.9 (C-6”a, C-6”b), 124.6 (C-5”a, C-5”b), 124.5 (C-2), 97.1 (C-9), 97.0 (C-1’), 74.1 (C-15), 73.2 (C-5’), 70.8 (C-4’), 69.5 (C-25), 66.8 (C-7), 66.1 (C-2’), 65.2 (C-11), 65.0 (C-13), 63.7 (C-3’), 57.8 (C-5), 57.3 (C-12), 53.7 (C-4), 46.2 (C-8), 44.1 (C-10), 42.6 (C-1”), 40.8 (C-6), 38.7* (C-24), 36.7 (C-14), 20.0 (C-27), 18.0 (C-6’),

*-overlapped

*Compound* ***19*** (1R,3S,5R,7R,8E,12R,14E,16E,18E,20E,22R,24S,25R,26S)-22-(((2S,3S,4S,5S,6R)-4-((cyclopropylmethyl)ammonio)-3,5-dihydroxy-6-methyltetrahydro-2H-pyran-2-yl)oxy)-1,3,26-trihydroxy-12-methyl-10-oxo-6,11,28-trioxatricyclo[22.3.1.05,7]octacosa-8,14,16,18,20-pentaene-25-carboxylate, (30 mg ,yield 27.4%), Elemental analysis C_37_H_53_NO_13_: C, 61.73; H, 7.45; N, 1.96; ESI-MS *m/z*: [M + H]^+^ 720.0, [M + Na]^+^ 742.0, [M - H]^-^ 718.0; melting point : 174-176°C with decomposition; FT-IR (KBr): ν(O-H) 3378 cm^-1^, ν(=C-H) 3017 cm^-1^, ν(C-H) 2976 cm^-1^, ν(N-H) 2835 cm^-1^, ν(C=O) 1713 cm^-1^, ν(C=C) 1577 cm^-1^, δ(C-OH) 1401 cm^-1^, ν(C-O-C) 1279 cm^-1^, ν(C-O) 1030 cm^-1^

^1^H NMR (600 MHz, dmso-d_6_) δ 9.60 (H_a_-NH_2_^+^), 8.80 (H_b_-NH_2_^+^), 6.49 (dd, *^3^J*_H18-H19_ =14.6 Hz, *^3^J*_H18-H17_ = 10.8 Hz, 1H, H-18), 6.26 (dd, *^3^J*= 15.8 Hz, *^3^J*_H3-H4_ = 7.7 Hz, 1H, H-3), 6.21-6.16 (m, 3H, H-19, H-21, H-20), 6.12-6.10 (m, 1H, H-2), , 6.09-6.04 (m, 2H, H-17, H-22), 5.88 (dd, *^3^J*_H16-H17_ = 15.3 Hz, *^3^J*_H16-H15_ = 8.8 Hz, 1H, H-16), 5.60 (ddd, *^3^J*_H23-H22_ = 15.2 Hz, *^3^J*_H23-H24a_ =9.6 Hz, *^3^J*_H23-H24b_ =5.8 Hz, 1H, H-23), 5.24^#^ (C_9_-OH), 4.69-4.64 (m, 1H, H-25), 4.41-4.35 (m, 2H, H-1’, H-15), 4.20-4.18 (m, 1H, H-13), 4.17-4.10 (m, 1H, H-7), 4.00 (td, *^3^J*_H11-H12_ = 10.8 Hz, ,*^3^J*_H11-H10a_ = 4.7 Hz, 1H, H-11), 3.72 (d, *^3^J*_H2’-H3’_ = 3.2 Hz, 1H, H-2’), 3.22 (dd, *^3^J*_H3-H4_ = 7.7 Hz, *^4^J*_H2-H4_ = 2.0 Hz, 1H, H-4), 3.16 (dq, *^3^J*_H4’-H5’_ =9.1 Hz, *^3^J*_H5’-H6’_ = 6.1 Hz, 1H, H-5’), 3.05-2.99 (m, 1H, H-4’), 2.76-2.73 (m, 1H, H-5), 2.56-2.52 (m, 1H, H-3’), 2.50^ (H1”a, H-1”b), 2.41-2.35 (m, 1H , H-24b), 2.19-2.13 (m, 1H, H-24a), 2.09-2.04 (m, 1H, H-14a), 1.98-1.92 (m, 2H, H-6b ,H-12), 1.83 (dd, *^2^J*_H10a-H10b_= 12.3 Hz, *^3^J*_H10a-H11_=4.7 Hz, 1H, H-10a), 1.59 (dd, *^2^J*_H8a-H8b_= 14.1 Hz, *^3^J*_H8a-H7_=10.6 Hz, 1H. H-8a), 1.56-1.47 (m, 2H, H-8b, H-14b), 1.26 (d, *^3^J*_H25-H27_ = 6.1 Hz, 3H, H-27), 1.16 (d, *^3^J*_H5’-H6’_ = 6.0 Hz, 3H, H-6’), 1.13-1.07 (m, 2H, H-10b, H-6a ), 1.03-0.92 (m, 1H, H-2”) 0.48-0.40 (m, 2H, H-3”), 0.20-0.13 (m, 2H, H-4”)

^-overlapped in dmso-d_6_*_,_* ^#^- determined from ^1^H-^1^H ROESY NMR

^13^C NMR (126 MHz, dmso-d_6_) δ 174.45 (C-26), 164.3 (C-1), 144.4 (C-3), 136.3 (C-16), 135.4 (C-22), 133.4 (C-18), 131.9 (C-20), 131.3 (C-19), 131.1 (C-21), 128.5 (C-23), 128.4 (C-17), 124.5 (C-2), 97.0 (C-9), 96.6 (C-1’), 74.2 (C-15), 72.8 (C-5’), 70.2 (C-4’), 69.5 (C-25), 66.0 (C-7), 65.7 (C-2’), 65.0 (C-13), 64.7 (C-11), 62.1 (C-3’), 57.8 (C-5), 57.3 (C-12), 53.6 (C-4), 50.26 (C-1”), 46.3 (C-8), 44.2 (C-10), 40.8 (C-6), 38.9* (C-24), 36.7 (C-14), 20.1 (C-27), 17.9 (C-6’), 10.2 (C-2”), 3.4 (C-3”), 3.2 (C-4”)

*-overlapped

*Compound* ***20*** (1R,3S,5R,7R,8E,12R,14E,16E,18E,20E,22R,24S,25R,26S)-22-(((2S,3S,4S,5S,6R)-3,5-dihydroxy-6-methyl-4-((2-methylbutyl)ammonio)tetrahydro-2H-pyran-2-yl)oxy)-1,3,26-trihydroxy-12-methyl-10-oxo-6,11,28-trioxatricyclo[22.3.1.05,7]octacosa-8,14,16,18,20-pentaene-25-carboxylate; (74 mg, yield 67%), Elemental analysis C_38_H_57_NO_13_: C, 62.00; H, 7.77; N, 1.90; ESI-MS *m/z*: [M + H]^+^ 736.0, [M + Na]^+^ 758.0, [M - H]^-^ 734.0; melting point : 149-152°C; FT-IR (KBr): ν(O-H) 3431 cm^-1^, ν(=C-H) 3021 cm^-1^, ν(C-H) 2968 cm^-1^, ν(N-H) 2879 cm^-1^, ν(C=O) 1717 cm^-1^, ν(C=C) 1575 cm^-1^, δ(C-OH) 1400 cm^-1^, ν(C-O-C) 1263 cm^-1^, ν(C-O) 1058 cm^-1^

^1^H NMR (500 MHz, dmso-d_6_) δ 9.60 (H_a_-NH_2_^+^), 8.30 (H_b_-NH_2_^+^), 6.49 (dd, *^3^J*_H18-H19_ =14.6 Hz, *^3^J*_H18-H17_ = 10.7 Hz, 1H, H-18), 6.27 (dd, *^3^J*_H3-H2_= 15.9 Hz, *^3^J*_H3-H4_=7.7 Hz, 1H, H-3, 6.21-6.16 (m, 3H, H-19, H-21, H-20), 6.12-6.10 (m, 1H, H-2), 6.10-6.02 (m, 2H, H-17, H-22), 5.88 (dd, *^3^J*_H16-H17_ = 15.3 Hz, *^3^J*_H16-H15_ = 8.7 Hz, 1H, H-16), 5.60 ( ddd, *^3^J*_H23-H22_ = 15.3 Hz, *^3^J*_H23-H24a_ =9.6 Hz, *^3^J*_H23-H24b_ =5.9 Hz, 1H, H-23), 5.26^#^ (C_9_-OH), 4.73-4.65 (m, 1H, H-25), 4.38-4.34 (m, 1H, H-15), 4.33 (bs, 1H, H-1’), 4.21-4.18 (m, 1H, H-13), 4.14-4.09 (m, 1H, H-7), 4.02 (td, *^3^J*_H11-H12_ = 10.8 Hz, *^3^J*_H11-H10a_= 4.7 Hz, 1H, H-11), 3.68 (d, *^3^J*_H2’-H3’_ = 3.1 Hz, 1H, H-2’), 3.22 (dd, *^3^J*_H3-H4_ = 7.7 Hz, *^4^J*_H2-H4_ = 2.0 Hz, 1H, H-4), 3.17-3.10 (m, 1H, H-5’), 2.98-2.92 (m, 1H, H-4’), 2.76-2.73 (m, 1H, H-5), 2.59 (dd, *^2^J*_H1”a=1”b_ = 11.5 Hz, *^3^J*_H1”b-2”_ =6.4 Hz, 1H, H-1”b), 2.42-2.34 (m, 2H, H-24b, H-3’), 2.31(dd, *^2^J*_H1”a=1”b_ = 11.5 Hz, *^3^J*_H1”a-2”_ =6.4 Hz, 1H, H-1”a), 2.19-2.13 (m, 1H, H-24a), 2.04-2.00 (m, 1H, H-14a), 2.00-1.91 (m, 2H, H-6b, H-12), 1.85 (dd, *^2^J*_H10a-H10b_= 12.3 Hz, *^3^J*_H10a-H11_=4.6 Hz, 1H, H-10a), 1.64-1.50 (m, 3H, H-8a, H-8b, H-14b), 1.49 (d, *^3^J*_H1”a-2”_= 6.0 Hz, 1H, H-2”), 1.26 (d, *^3^J*_H25-H27_ = 6.1 Hz, 3H, H-27), 1.22-1.17 (m, 1H-6a), 1.15-1.04 (m, 5H, H-10b, H-6’, H-3” ) 0.88-0.86 (m, 6H, H-4”, H-5”)

^#^- determined from ^1^H-^1^H ROESY NMR

^13^C NMR (126 MHz, dmso-d_6_) δ 174.1 (C-26), 164.3 (C-1), 144.4 (C-3), 136.3 (C-16), 135.4 (C-22), 133.4 (C-18), 131.9 (C-20), 131.3 (C-19), 131.1 (C-21), 128.5 (C-23), 128.3 (C-17), 124.5 (C-2), 97.0 (C-9), 96.8 (C-1’), 74.1 (C-15), 72.9 (C-5’), 70.5 (C-4’), 69.5 (C-25), 66.00 (C-2’), 65.8 (C-7), 65.2 (C-11), 65.0 (C-13), 62.9 (C-3’), 57.8 (C-5), 57.2 (C-12), 53.6 (C-4), 51.9/51.7 (C-1”), 46.2 (C-8), 44.1 (C-10), 40.8 (C-6), 38.7* (C-24), 36.8 (C-14), 34.3/34.2 (C-2”), 26.9/26.6 (C-3”), 20.0 (C-27), 17.9 (C-6’), 17.5/17.4 (C-5”), 11.1/ 10.9 (C-4”)

*Compound* ***21*** (1R,3S,5R,7R,8E,12R,14E,16E,18E,20E,22R,24S,25R,26S)-22-(((2S,3S,4S,5S,6R)-4-(decylammonio)-3,5-dihydroxy-6-methyltetrahydro-2H-pyran-2-yl)oxy)-1,3,26-trihydroxy-12-methyl-10-oxo-6,11,28-trioxatricyclo[22.3.1.05,7]octacosa-8,14,16,18,20-pentaene-25-carboxylate; (60 mg ,yield 49.6%), Elemental analysis C_43_H_67_NO_13_: C, 64.01; H, 8.36; N, 1.70; ESI-MS *m/z*: [M + H]^+^ 806.0, [M + H]^+^ 828.0, [M - H]^-^ 804.0; melting point : 159-161°C; FT-IR (KBr): ν(O-H) 3414 cm^-1^, ν(=C-H) 3019 cm^-1^, ν(C-H) 2954 cm^-1^, ν(N-H) 2855 cm^-1^, ν(C=O) 1717 cm^-1^, ν(C=C) 1575 cm^-1^, δ(C-OH) 1376 cm^-1^, ν(C-O-C) 1265 cm^-1^, ν(C-O) 1060 cm^-1^

^1^H NMR (600 MHz, dmso-d_6_) δ 8.50 (H_a_-NH_2_^+^), 8.20 (H_b_-NH_2_^+^), 6.50 (dd, *^3^J*_H18-H19_ =14.5 Hz, *^3^J*_H18-H17_ = 10.8 Hz, 1H, H-18), 6.27 (dd, *^3^J*_H2-H3_ = 15.8Hz, *^3^J*_H3-H4_ = 7.7 Hz, 1H, H-3), 6.21-6.16 (m, 3H, H-19, H-21, H-20), 6.13-6.10 (m, 1H, H-2), 6.10-6.07 (m, 2H, H-17, H-22), 5.89 (dd, *^3^J*_H16-H17_ = 15.3 Hz, *^3^J*_H16-H15_ = 8.7 Hz, 1H, H-16), 5.60 (ddd, *^3^J*_H23-H22_ = 15.3 Hz, *^3^J*_H23-H24a_ =9.7 Hz, *^3^J*_H23-H24b_ =5.9 Hz, 1H, H-23), 5.26^#^ (C_9_-OH), 4.69-4.62 (m, 1H, H-25), 4.41 (bs, 1H, H-1’), 4.40-4.36 (m, 1H, H-15), 4.20-4.18 (m, 1H, H-13) 4.14-4.09 (m, 1H, H-7), 4.00 (td, *^3^J*_H11-H12_ = 10.7 Hz, 4.7 Hz, 1H, H-11), 3.78 (d, *^3^J*_H2’-H3’_ = 3.1 Hz, 1H, H-2’), 3.22 (dd, *^3^J*_H3-H4_ = 7.7 Hz, *^4^J*_H2-H4_ = 2.0 Hz, 2H, H-4), 3.21-3.17 (m, 1H, H-5’), 3.12-3.07 (m, 1H, H-4’), 2.80-2.74 (m, 2H, H-5, H-1”a, ), 2.67-2.59 (m, 2H, H-3’, H-1”b), 2.38-2.33 (m, 1H, H-24b), 2.19-2.12 (m, 1H, H-24a), 2.11-2.05 (m, 1H. H-14a), 1.94-1.90 (m, 1H, H-6b), 1.89-1.85 (m, 1H, H-12), 1.84-1.80 (m, 1H, H-10a), 1.62-1.54 (m, 1H, H-8a), 1.56-1.49 (m, 2H, H-8b, H-14b), 1.26- 1.20* (m, 19H, H-27, H-2”, H-3”, H-4”, H-5”, H-6”, H-7”, H-8”, H-9”), 1.16 (d, *^3^J­*_H5’-H6’_*=*6.1 Hz, 3H, H-6’), 1.15-1.08 (m, 2H, H-6a, H-10b), 0.88-0.84 (m, 4H , H-10”)

*-overlapped, ^#^- determined from ^1^H-^1^H ROESY NMR

^13^C NMR (126 MHz, dmso-d_6_) δ 174.9, (C-26) 164.3 (C-1), 144.4 (C-3), 136.2 (C-16), 135.4 (C-22), 133.4 (C-18), 131.9 (C-21), 131.3 (C-19), 131.1 (C-20), 128.5 (C-23), 128.4 (C-17), 124.4 (C-2), 97.00 (C-9), 96.3 (C-1’), 74.3 (C-15), 72.6 (C-5’), 69.8 (C-4’), 69.6 (C-25), 66.0 (C-7), 65.5 (C-2’), 65.23 (C-11), 65.1 (C-13), 62.3 (C-3’), 57.9 (C-5), 57.4 (C-12), 53.6 (C-4), 46.4 (C-8), 45.3 (C-1”), 44.1 (C-10), 40.8 (C-6), 38.7* (C-24), 36.7 (C-14), 31.2 (C-8”), 28.9 (C-3”), 28.8 (C-2”), 28.7 (C-6”), 28.5 (C-7”), 26.5 (C-4”, C-5”), 21.9 (C-9”), 20.0 (C-27), 17.9 (C-6’), 13.8 (C-10”).

*Compound* ***22*** (1R,3S,5R,7R,8E,12R,14E,16E,18E,20E,22R,24S,25R,26S)-22-(((2S,3S,4S,5S,6R)-4-(dodecylammonio)-3,5-dihydroxy-6-methyltetrahydro-2H-pyran-2-yl)oxy)-1,3,26-trihydroxy-12-methyl-10-oxo-6,11,28-trioxatricyclo[22.3.1.05,7]octacosa-8,14,16,18,20-pentaene-25-carboxylate; (57 mg, yield 49.5%), Elemental analysis C_45_H_71_NO_13_: C, 64.73; H, 8.51; N, 1.64; ESI-MS *m/z*: [M + H]^+^ 835.0, [M + Na]^+^ 857.0, [M - H]^-^ 832.0 ; melting point : 168-170°C with decomposition; FT-IR (KBr): ν(O-H) 3398 cm^-1^, ν(=C-H) 3019 cm^-1^, ν(C-H) 2926 cm^-1^, ν(N-H) 2853 cm^-1^, ν(C=O) 1720 cm^-1^, ν(C=C) 1573 cm^-1^, δ(C-OH) 1376 cm^-1^, ν(C-O-C) 1232 cm^-1^, ν(C-O) 1056 cm^-1^

^1^H NMR (600 MHz, dmso-d_6_) δ 10.30 (H_a_-NH_2_^+^), 9.50 (H_b_-NH_2_^+^), 6.50 (dd, *^3^J*_H18-H19_ =14.5 Hz, *^3^J*_H18-H17_ = 10.8 Hz, 1H, H-18), 6.27 (dd, *^3^J*_H2-H3_ = 15.8Hz, *^3^J*_H3-H4_ = 7.7 Hz, 1H, H-3), 6.24-6.16 (m, 3H, H-19, H-20, H-21), 6.13-6.10 (m, 1H, H-2), 6.10-6.07 (m, 2H, H-17, H-22), 5.89 (dd, *^3^J*_H16-H17_ = 15.3 Hz, *^3^J*_H16-H15_ = 8.8 Hz, 1H, H-16), 5.60 (ddd, *^3^J*_H23-H22_ = 15.3 Hz, *^3^J*_H23-H24a_ =9.7 Hz, *^3^J*_H23-H24b_ =6.0 Hz, 1H, H-23), 5.27^#^ (C_9_-OH), 4.69-4.62 (m, 1H, H-25), 4.44-4.35 (m, 2H, H-15, H-1’), 4.20-4.17 (m, 1H, H-13) 4.14-4.09 (m, 1H, H-7), 4.00 (td, *^3^J*_H11-H12_ = 10.7 Hz, 4.7 Hz, 1H, H-11), 3.78 (d, *^3^J*_H1’-H2’_ = 3.1 Hz, 1H, H-2’), 3.22 (dd, *^3^J*_H3-H4_ = 7.6 Hz, *^4^J*_H2-H4_ = 1.9 Hz, 1H, H-4), 3.22-3.16 (m, 1H, H-5’), 3.12-3.07 (m, 1H, H-4’), 2.80-2.73 (m, 2H, H-5, H-1”a), 2.66-2.60 (m, 2H, H-3’, H-1”b), 2.38-2.34 (m, 1H, H-24b), 2.19-2.13 (m, 1H, H-24a), 2.11-2.04 (m, 1H. H-14a), 1.94-1.90 (m, 1H, H-6b), 1.88-1.85 (m, 1H, H-12), 1.84 (dd, *^2^J*_H10a-H10b_= 12.3 Hz, *^3^J*_H10a-H11_=4.6 Hz 1H, H-10a), 1.62–1.57 (m, 1H, H-8a), 1.58–1.51 (m, 2H, H-8b, H-14b), 1.30.-1.20 (m, 23H, H-27, H-2”, H-3”, H-4”, H-5”, H-6”, H-7”, H-8”, H-9”, H-10”, H-11”), 1.20–1.09 (m, 5H, H-6a, H-10b, H-6’), 0.86 (t, *^3^J*_H11”-H12”_ =6.9 Hz, 3H, H-12”)

*-overlapped, ^#^- determined from ^1^H-^1^H ROESY NMR

^13^C NMR (126 MHz, dmso-d_6_) δ 174.8 (C-26), 164.3 (C-1), 144.4 (C-3), 136.2 (C-16), 135.4 (C-22), 133.4 (C-18), 131.9 (C-21), 131.3 (C-19), 131.1 (C-20), 128.5 (C-23), 128.4 (C-17), 124.4 (C-2), 97.00 (C-9), 96.3 (C-1’), 74.3 (C-15), 72.7 (C-5’), 69.8 (C-4’), 69.5 (C-25), 66.0 (C-7), 65.5 (C-2’), 65.3 (C-11), 65.1 (C-13), 62.3 (C-3’), 57.9 (C-5), 57.4 (C-12), 53.6 (C-4), 46.4 (C-8), 45.3 (C-1”), 44.1 (C-10), 40.8 (C-6), 38.8* (C-24), 36.7 (C-14), 31.1 (C-10”), 29.0 (C-3”), 28.9 (C-4”), 28.8 (C-5”), 28.7 (C-6”, C-7”, C-8”), 28.5 (C-9”), 26.5 (C-2”), 21.9 (C-11”), 20.0 (C-27), 17.9 (C-6’), 13.7 (C-12”).

*-overlapped

*Compound* ***23*** (1R,3S,5R,7R,8E,12R,14E,16E,18E,20E,22R,24S,25R,26S)-22-(((2S,3S,4S,5S,6R)-4-(dioctylammonio)-3,5-dihydroxy-6-methyltetrahydro-2H-pyran-2-yl)oxy)-1,3,26-trihydroxy-12-methyl-10-oxo-6,11,28-trioxatricyclo[22.3.1.05,7]octacosa-8,14,16,18,20-pentaene-25-carboxylate;( 66 mg, yield 49.3%), Elemental analysis C_49_H_79_NO_13_: C, 66.10; H, 8.91; N, 1.53; ESI-MS *m/z*: [M + H]^+^ 890.0, [M + Na]^+^ 912.0, [M - H]^-^ 888.0; melting point : 145-146°C; FT-IR (KBr): ν(O-H) 3394 cm^-1^, ν(=C-H) 3019 cm^-1^, ν(C-H) 2929 cm^-1^, ν(N-H) 2856 cm^-1^, ν(C=O) 1716 cm^-1^, ν(C=C) 1570 cm^-1^, δ(C-OH)= 1398 cm^-1^, ν(C-O-C) 1275 cm^-1^, ν(C-O) 1058 cm^-1^

^1^H NMR (600 MHz, dmso-d_6_) δ 8.28 (H-NH^+^), 6.49 (dd, *^3^J*_H18-H19_ =14.6 Hz, *^3^J*_H18-H17_ = 10.8 Hz, 1H, H-18), 6.27 (dd, *^3^J*_H2-H3_ = 15.8Hz, *^3^J*_H3-H4_ = 7.7 Hz, 1H, H-3), 6.21-6.16 (m, 3H, H-19, H-20, H-21), 6.13-6.10 (m, 1H, H-2), 6.10 – 6.03 (m, 2H, H-17, H-22), 5.88 (dd, *^3^J*_H16-H17_ = 15.3 Hz, *^3^J*_H16-H15_ = 8.8 Hz, 1H, H-16), 5.61 ( ddd, *^3^J*_H22-H23_ = 15.3 Hz, *^3^J*_H23-H24a_ =9.7 Hz, *^3^J*_H23-H24b_ =5.9 Hz, 1H, H-23), 5.25^#^ (C_9_-OH), 4.68-4.61 (m, 1H, H-25), 4.42-4.39 (m,1H , H-15), 4.38 (bs, 1H, H-1’), 4.20-4.17 (m, 1H, H-13) 4.14-4.09 (m, 1H, H-7), 4.01 (td, *^3^J*_H11-H12_ = 10.8 Hz, *^3^J*_H11-H10a_= 4.7 Hz, 1H, H-11), 3.75 (d, *^3^J*_H2’-H3’_ = 3.1 Hz, 1H, H-2’), 3.22 (dd, *^3^J*_H3-H4_ = 7.7 Hz, *^4^J*_H2-H4_ = 2.0 Hz, 1H, H-4), 3.21-3.16 (m, 1H, H-5’), 3.10-3.05 (m, 1H, H-4’), 2.78-2.73 (m, 3H, H-5, H-1”a, H-1’”a), 2.65-2.56 (m, 3H, H-3’, H-1”b, H-1”’b), 2.41-2.35 (m, 1H, H-24b), 2.19-2.13 (m, 1H, H-24a), 2.08-2.00 (m, 1H, H-14a), 1.98-1.91 (m, 2H , H-6b, H-12), 1.84 (dd, *^2^J*_H10a-H10b_= 12.3 Hz, *^3^J*_H10a-H11_=4.6 Hz 1H, H-10a) 1.63-1.48 (m, 5H, H-8a, H-8b H-2”, H-2’”, H-14b), 1.29-1.24* (m, 26H , H-27, H-3”,H-4”,H-5”, H-6”, H-7”,H-3”’, H-4”’, H-5”’, H-6”’, H-7”’), 1.16-1.10 (m, 3H, H-6’), 1.16-1.07 (m, 9H, H-6a, H-10b,), 0.89-0.87 (m, 8H, H-8”, H-8”’)

^#^- determined from ^1^H-^1^H ROESY NMR

^13^C NMR (126 MHz, dmso-d_6_) δ 174.7 (C-26), 164.3 (C-1), 144.4 (C-3), 136.2 (C-16), 135.4 (C-22), 133.4 (C-18), 131.9 (C-20), 131.3 (C-21), 131.1 (C-19), 128.5 (C-23), 128.4 (C-17), 124.5 (C-2), 97.0 (C-9), 96.3 (C-1’), 74.1 (C-15), 72.7 (C-5’), 69.9 (C-4’), 69.5 (C-25), 66.1 (C-7), 65.6 (C-2’), 65.3 (C-11), 65.1 (C-13), 62.2 (C-3’), 57.9 (C-12), 57.5 (C-5), 53.6 (C-4), 46.3 (C-8), 45.2 (C-1”, C-1’”), 44.2 (C-10), 40.7 (C-6), 38.7* (C-24), 36.6 (C-14), 31.1 (C-6”,C-6’”), 28.6 (C-5”,C-5’”), 28.5 (C-4”,C-4’”), 28.0 (C-2”, C-2’”), 26.5 (C-3”, C-3’”), 21.9 (C-7”, C-7’”), 20.1 (C-27), 17.9 (C-6’), 13.7 (C-8”, C-8’”)

*-overlapped

*Compound* ***24*** (1R,3S,5R,7R,8E,12R,14E,16E,18E,20E,22R,24S,25R,26S)-22-(((2S,3S,4S,5S,6R)-4-(diethylammonio)-3,5-dihydroxy-6-methyltetrahydro-2H-pyran-2-yl)oxy)-1,3,26-trihydroxy-12-methyl-10-oxo-6,11,28-trioxatricyclo[22.3.1.05,7]octacosa-8,14,16,18,20-pentaene-25-carboxylate; (40 mg, yield 38.4%), Elemental analysis C_37_H_55_NO_13_: C, 61.50; H, 7.61; N, 1.98; ESI-MS *m/z*: [M + H]^+^ 722.0, [M - H]^-^ 720.0; melting point: 137-140°C FT-IR (KBr): ν(O-H), 3394 cm^-1^, ν(=C-H) 3015 cm^-1^, ν(C-H) 2930 cm^-1^, ν(N-H) 2855 cm^-1^, ν(C=O) 1715 cm^-1^, ν(C=C) 1574 cm^-1^, δ(C-OH) 1399 cm^-1^, ν(C-O-C) 1264 cm^-1^, ν(C-O) 1057 cm^-1^

^1^H NMR (600 MHz, dmso-d_6_) δ 9.50 (H-NH^+^), 6.49 (dd, ^3^*J* _H19-H18_= 14.8, ^3^*J*_H18-H17_ =10.0 Hz, 1H, H-18), 6.27 (dd, *^3^J*_H3-H2_= 15.8 Hz, *^3^J*_H3-H4_=7.7 Hz, 1H, H-3, 6.25-6.16 (m, 3H, H-19, H-20, H-21), 6.10-6.08 (m, 2H, H-2, H-17), 6.08-6.02 (m, 1H, H-22), 5.92-5.85 (m, 1H, H-16), 5.60 (ddd, *^3^J*_H23-H22_= 15.3Hz; *^3^J*_H23-H24a_= 9.7Hz, *^3^J*_H23-H24b_= 5.9Hz, 1H, H-23), 5.21^#^ (C_9_-OH), 4.72-4.67 (m, 1H, H-25), 4.40-4.34 (m, 2H, H-15),4.32-4.29 (m, 1H, H-1’), 4.20-4.17 ( m, 1H, H-13), 4.12-4.09 (m, 1H, H-7), 4.00-3.97 (m, 1H, H-11), 3.81 (d, *^3^J*_H2’-H3’_= 2.5 Hz, 1H, H-2’), 3.40-3.38 ( m, 1H, H-4’), 3.22-3.21 (m, 1H, H-4), 3.16-3.09 (m, 1H, H-5’), 2.85-2.80 (m, 2H, H-1’’a; H-1”’a), 2.76-2.72 (m, 1H, H-5), 2.71-2.65 (m, 1H, H-1’’b; H-1”’b), 2.51-2.45 (m, 1H, H-3’), 2.41-2.34 (m, 1H, H-24b), 2.19-2.13 ( m, 1H, H-24a), 2.06-2.02 (m, 1H, H-14a), 1.95-1.90 (m, 2H, H-6b; H-12), 1.84 ( dd, *^2^J*_H10a-H10b_= 12.3 Hz, *^3^J*_H10a-H11_= 4.6 Hz, 1H, H-10a), 1.63-1.52 (m, 2H, H-8a, H-14b), 1.50 (dd, *^2^J*_H8a-H8b_= 14.2 Hz, 1H, *^3^J*_H8b-H7_= 2.0 Hz, 1H, H-8b) 1.28-1.22 (m, 3H, H-27), 1.19-1.16 (m, 1H, H-6a), 1.15-1.14 (m, 3H, H-6’), 1.14-1.07 (m, 3H, H-10b), 0.99 (q, *^3^J* _H1”-H2”, H1”’-H2”_= 6.7 Hz, 7H, H-2’’;H-2”’)

^#^- determined from ^1^H-^1^H ROESY NMR

^13^C NMR (126 MHz, dmso-d_6_) δ 174.1 (C-26), 164.3 (C-1), 144.43 (C-3), 136.33 (C-16), 135.36 (C-22), 133.42 (C-18), 131.89 (C-20), 131.28 (C-21), 131.07 (C-19), 128.45 (C-23), 128.37 (C-17), 124.45( C-2), 97.15 (C-9), 97.03 (C-1’), 74.07 (C-15), 73.67 (C-5’), 69.47 (C-25), 68.62 (C-2’), 67.26 (C-4’), 66.00 (C-7), 65.07 (C-11), 64.73 (C-13), 64.22 (C-3’), 57.88 (C-5), 56.98 (C-12), 53.67 (C-4), 46.31 (C-8), 44.25 (C-10), 44.10 (C-1’’, C-1”’), 40.77 (C-6), 38.78* (C-24), 36.94 (C-14), 20.05 (C-27), 17.93 (C-6’), 14.47 (C-2”, C-2”’)

*-overlapped

*Compound* ***25*** (1R,3S,5R,7R,8E,12R,14E,16E,18E,20E,22R,24S,25R,26S)-22-(((2S,3S,4S,5S,6R)-4-((3-(decyloxy)benzyl)amino)-3,5-dihydroxy-6-methyltetrahydro-2H-pyran-2-yl)oxy)-N-(4-(dimethylamino)benzyl)-1,3,26-trihydroxy-12-methyl-10-oxo-6,11,28-trioxatricyclo[22.3.1.05,7]octacosa-8,14,16,18,20-pentaene-25-carboxamide; (11.1 mg, yield 48%), Elemental analysis C_59_H_85_N_3_O_13_: C, 67.82; H, 8.25; N, 4.12; ESI-MS *m/z*: [M + H]^+^ 1045.0, [M - H]^-^ 1043.0; FT-IR (KBr): ν(O-H) 3398 cm^-1^, ν(O-H)_amide_ 3280 cm^-1^ , ν(C-H)_ar_ 3076 cm^-1^, ν(C-H) 2924 cm^-1^, ν(N-H) 2853 cm^-1^, ν(C=O) 1723 cm^-1^, ν(C=O)_amide_ 1616 cm^-1^, ν(C=C) 1570 cm^-1^, δ(C-OH) 1295 cm^-1^, ν(N-C)_amide_ 1285 cm^-1^, ν(C-O-C) 1251 cm^-1^, ν(C-O) 1058 cm^-1^, γ(=C-H)_ar_ 839 cm^-1^

^1^H NMR (600 MHz, dmso-d_6_) δ 10.13 (H_a_-NH_2_^+^), 9.67 (H_b_-NH_2_^+^), 7.38-7.30 (m, 3H, H-5”, H7”), 7.06-7.02 (m, 2H, H-4A, H-4AB), 6.82 (d, *J* = 8.5 Hz, 2H, H-3”, H-6”), 6.64-6.61 (m,2H, H-3A, H-3AB), 6.50 (dd, *^3^J*_H18-H19_= 14.7 Hz, *^3^J*_H18-H17_= 10.9 Hz, 1H H-18), 6.28 (dd, *^3^J*_H2-H3_=15.8 Hz, *^3^J*_H3-H4_= 7.6 Hz, 1H, H-3), 6.24–6.17 (m, 3H, H-19,H-20, H-21), 6.12-6.10 (m, 1H, H-2), 6.09–6.03 (m, 2H, H-17, H-22), 5.89 (dd, *^3^J*_H16-H17_= 15.3 Hz, *^3^J*_H16-H15_= 8.9 Hz, 1H, H-16), 5.60 (ddd, *^3^J*_H22-H23_= 15.4 Hz, *^3^J*_H23-H24a_= 9.8 Hz, *^3^J*_H23-H24b_= 5.9 Hz, 1H, H-23), 5.30^#^ (C_9_-OH), 4.69-4.62 (m, 1H, H-25), 4.36-4.31 (m, 1H, H-15), 4.32-4.28 (m, 1H, H-13), 4.24-4.03 (m, 4H, H-7,H-11, H-1’, H-1A), 3.95 – 3.91 (m, 2H, H-1”a, H-2’), 3.85 – 3.80 (m, 2H, H-1”’), 3.69 (d, *^2^J*_H1”a-H1”b_=11.3 Hz, 1H, H-1”b), 3.24-3.21 (m, 1H, H-4), 3.16 – 2.97 (m, 1H, H-4’), 2.96 – 2.91 (m, 1H, H-5’), 2.90 (s, 4H, H-6A), 2.84 (s, 10H, H-7A), 2.76-2.73 (m, 1H, H-5), 2.42 – 2.35 (m, 2H, H-24b, H-3’), 2.19-2.14 (m, 1H, H-24a), 2.00-1.93 (m, 3H, H-6b, H-12, H-14a), 1.87 (dd, *^2^J*_H10a-H10b_= 12.4 Hz, *^3^J*_H10a-H11_= 4.7 Hz, 1H, H-10a), 1.69-1.58 (m, 2H, H-2”’, H-8a), 1.51-1.47 (m, 1H, H-8b), 1.44-1.41 (m, 1H, H-14b), 1.40-1.32 (m, 2H, H-3”’), 1.17-1.13 (m, 10H, H-6a, H-10b), 1.31–1.23* (m, 28H, H-27, H-4”’, H-5”’, H-6”’, H-7”’, H-8”’, H-9”’), 1.12 (d, *^3^J*_H5’-H6’_= 6.1 Hz, 3H, H-6’), 0.86 (t, *^3^J*_H9”’-10”’_=6.9 Hz, 5H, H-10”’)

*-overlapped, ^#^- determined from ^1^H-^1^H ROESY NMR

^13^C NMR (126 MHz, dmso-d_6_) δ 164.3 (C-1), 157.8 (C-4”), 149.3 (C-5A), 144.3 (C-3), 136.2 (C-16), 135.4 (C-22), 133.4 (C-18), 131.9 (C-21), 131.3 (C-20), 131.1 (C-19), 130.75 (C-2”), 130.2 (C-5”), 129.8 (C-7”), 128.5 (C-23), 128.2 (C-17), 127.9 (C-4A, C-4AB), 126.8 (C-2A), 124.4 (C-2), 114.1 (C-3”), 112.8 (C-3A, C-3AB), 112.0 (C-6”), 96.9 (C-9), 96.3 (C-1’), 74.1 (C-15), 72.7 (C-5’), 69.8 (C-4’), 69.5 (C-25), 67.3 (C-1”’), 66.1 (C-7), 65.3 (C-2’), 65.1 (C-13), 64.6 (C-11), 61.1 (C-3’), 57.9 (C-5), 57.1 (C-12), 53.6 (C-4), 48.2 (C-1”), 46.3 (C-8), 44.6 (C-10), 41.3 (C-1A), 40.8 (C-6), 39.8* (C-7A), 39.7* (C-6A), 38.8*(C-24), 36.2 (C-14), 32.1 (C-7”’, C-8”’), 28.8 (C-6”’), 28.8 (C-5”’), 28.6 (C-4”’), 28.5 (C-2”’), 25.4 (C-3”’), 21.9 (C-9”’), 20.1 (C-27), 17.8 (C-6’), 13.8 (C-10”’)

*-overlapped

# Fig. S1 FT-IR spectrum of PIM (in KBr).

Fig. S2 1H NMR spectra of PIM in DMSO-d6.

# Fig. S3 ^13^C NMR spectra of PIM in DMSO-d_6_.

# Fig. S4 ^1^H-^13^C HSQC spectra of PIM in DMSO-d_6_.

# Fig. S5 ^1^H-^13^C HMBC spectra of PIM in DMSO-d_6_.

# Fig. S6 ^1^H-^1^H COSY spectra of PIM in DMSO-d_6_.

# Fig. S7 ^1^H-^1^H ROESY spectra of PIM in DMSO-d_6_.

# Fig. S8 ^1^H-^15^N gHSQC NMR spectrum of **PIM** revealing chemical shift of a broadened signal assigned to a highly exchangeable protons form group -NH_3_^+^.

# Fig. S9 FT-IR spectrum of compound **1** (in KBr).

Fig. S10 ESI-MS spectrum of compound 1.

# Fig. S11 ^1^H NMR spectra of compound **1** in DMSO-d_6_.

# Fig. S12 ^13^C NMR spectra of compound **1** in DMSO-d_6_.

# Fig. S13 ^1^H-^13^C HSQC spectra of compound **1** in DMSO-d_6_.

Fig. S14 1H-13C HMBC spectra of compound 1 in DMSO-d_6_.

# Fig. S15 ^1^H-^1^H COSY spectra of compound **1** in DMSO-d_6_.

# Fig. S16 ^1^H-^1^H ROESY spectra of compound **1** in DMSO-d_6_.

# Fig. S17 FT-IR spectrum of compound **2** (in KBr).

Fig. S18 ESI-MS spectrum of compound 2.

**[2+ H]** **^+^**

**[2-H]** **^-^**

# Fig. S19 ^1^H NMR spectra of compound **2** in DMSO-d_6_.

Fig. S20 13C NMR spectra of compound 2 in DMSO-d_6_.

# Fig. S21 ^1^H-^13^C HSQC spectra of compound **2** in DMSO-d_6_.

# Fig. S22 ^1^H-^13^C HMBC spectra of compound **2** in DMSO-d_6_.

# Fig. S23 ^1^H-^1^H COSY spectra of compound **2** in DMSO-d_6_.

Fig. S24A 1H-1H ROESY spectra of compound 2 in DMSO-d_6_.

# Fig. S24B Fragment of ^1^H-^15^N gHMBC NMR spectrum of **2** showing correlation between hydrogen atom (H-4’) of mycosamine and nitrogen atom from -NH_2_^+^-.

# Fig. S25 FT-IR spectrum of compound **3** (in KBr).

**[3+ Na]** **^+^**

**[3-H]** **^-^**

# Fig. S26 ESI-MS spectrum of compound **3**.

Fig. S27 1H NMR spectra of compound 3 in DMSO-d_6_.

# Fig. S28 ^13^C NMR spectra of compound **3** in DMSO-d_6_.

# Fig. S29 ^1^H-^13^C HSQC spectra of compound **3** in DMSO-d_6_.

# Fig. S30 ^1^H-^13^C HMBC spectra of compound **3** in DMSO-d_6_.

# Fig. S31 ^1^H-^1^H COSY spectra of compound **3** in DMSO-d_6_.

# Fig. S32 ^1^H-^1^H ROESY spectra of compound **3** in DMSO-d_6_.

Fig. S33 Structure of compound **3** being in agreement with ^1^H-^1^H ROESY contacts, noted in the spectra recorded in dmso-d_6_ solutions [*Scigress* FJ. 2.6 EU 3.1.9, Fujitsu 2008-2026; method B88 LYP (GGA)].

# Fig. S34 FT-IR spectrum of compound **4** (in KBr).

**[4-H]** **^-^**

**[4+ Na]** **^+^**

# Fig. S35 ESI-MS spectrum of compound **4**.

Fig. S36 1H NMR spectra of compound 4 in DMSO-d_6_.

# Fig. S37 ^13^C NMR spectra of compound **4** in DMSO-d_6_.

# Fig. S38 ^1^H-^13^C HSQC spectra of compound **4** in DMSO-d_6_.

# Fig. S39 ^1^H-^13^C HMBC spectra of compound **4** in DMSO-d_6_.

# Fig. S40 ^1^H-^1^H COSY spectra of compound **4** in DMSO-d_6_.

# Fig. S41 ^1^H-^1^H ROESY spectra of compound **4** in DMSO-d_6_.

Fig. S42 Structure of compound **4** being in agreement with ^1^H-^1^H ROESY contacts, noted in the spectra recorded in dmso-d_6_ solutions [*Scigress* FJ. 2.6 EU 3.1.9, Fujitsu 2008-2026; method B88 LYP (GGA)].

Fig.S42A

Fig. S42A Fragment of 1H-1H ROESY spectra of compound **4** showing correlations between hydroxyl group (OH-9) and protons from macrolactone ring.

# Fig. S43 FT-IR spectrum of compound **5** (in KBr).

Fig. S44 ESI-MS spectrum of compound **5**.

**[5+ Na]** **^+^**

**[5-H]** **^-^**

# Fig. S45 ^1^H NMR spectra of compound **5** in DMSO-d_6_.

# Fig. S46 ^13^C NMR spectra of compound **5** in DMSO-d_6_.

# Fig. S47 ^1^H-^13^C HSQC spectra of compound **5** in DMSO-d_6_.

# Fig. S48 ^1^H-^13^C HMBC spectra of compound **5** in DMSO-d_6_.

# Fig. S49 ^1^H-^1^H COSY spectra of compound **5** in DMSO-d_6_.

# Fig. S50 ^1^H-^1^H ROESY spectra of compound **5** in DMSO-d_6_.

# Fig. S51 FT-IR spectrum of compound **6** (in KBr).

**[6+ Na]** **^+^**

**[6-H]** **^-^**

# Fig. S52 ESI-MS spectrum of compound **6**.

# Fig. S53 ^1^H NMR spectra of compound **6** in DMSO-d_6_.

# Fig. S54 ^13^C NMR spectra of compound **6** in DMSO-d_6_.

# Fig. S55 ^1^H-^13^C HSQC spectra of compound **6** in DMSO-d_6_.

# Fig. S56 ^1^H-^13^C HMBC spectra of compound **6** in DMSO-d_6_.

# Fig. S57 ^1^H-^1^H COSY spectra of compound **6** in DMSO-d_6_.

# Fig. S58 ^1^H-^1^H ROESY spectra of compound **6** in DMSO-d_6_.

# Fig. S59 FT-IR spectrum of compound **7** (in KBr).

**[7-H]** **^-^**

**[7+ Na]** **^+^**

# Fig. S60 ESI-MS spectrum of compound **7**.

# Fig. S61 ^1^H NMR spectra of compound **7** in DMSO-d_6_.

# Fig. S62 ^13^C NMR spectra of compound **7** in DMSO-d_6_.

# Fig. S63 ^1^H-^13^C HSQC spectra of compound **7** in DMSO-d_6_.

# Fig. S64 ^1^H-^1^H COSY spectra of compound **7** in DMSO-d_6_.

# Fig. S65 ^1^H-^1^H ROESY spectra of compound **7** in DMSO-d_6_.

Fig. S66A Fragment of 1H NMR spectrum of compound 7, showing signals of exchangeable protons at the nitrogen of mycosamine (spectrum in the range 9.35-10.15 ppm).

# Fig. S66B Fragment of ^1^H-^15^N gHMBC NMR spectrum of **7** showing correlation between hydrogen atom (H-4’) of mycosamine and nitrogen atom from -NH_2_^+^-.

# Fig. S67 Fragment of ^13^C NMR spectrum of compound **7**, showing lack of signals from TEA.

# Fig. S68 Fragment of ^1^H-^13^C HSQC NMR spectrum of compound **7**, showing lack of correlations from TEA.

# Fig. S69 FT-IR spectrum of compound **8** (in KBr).

**[8+ H]** **^+^**

**[8-H]** **^-^**

# Fig. S70 ESI-MS spectrum of compound **8**.

# Fig. S71 ^1^H NMR spectra of compound **8** in DMSO-d_6_.

# Fig. S72 ^13^C NMR spectra of compound **8** in DMSO-d_6_.

# Fig. S73 ^1^H-^13^C HSQC spectra of compound **8** in DMSO-d_6_.

# Fig. S74 ^1^H-^13^C HMBC spectra of compound **8** in DMSO-d_6_.

# Fig. S75 ^1^H-^1^H COSY spectra of compound **8** in DMSO-d_6_.

# Fig. S76 FT-IR spectrum of compound **9** (in KBr).

**[9-H]** **^-^**

**[9+ Na]** **^+^**

# Fig. S77 ESI-MS spectrum of compound **9**.

# Fig. S78 ^1^H NMR spectra of compound **9** in DMSO-d_6_.

# Fig. S79 ^13^C NMR spectra of compound **9** in DMSO-d_6_.

# Fig. S80 ^1^H-^13^C HSQC spectra of compound **9** in DMSO-d_6_.

# Fig. S81 ^1^H-^13^C HMBC spectra of compound **9** in DMSO-d_6_.

# Fig. S82 ^1^H-^1^H COSY spectra of compound **9** in DMSO-d_6_.

# Fig. S83 ^1^H-^1^H ROESY spectra of compound **9** in DMSO-d_6_.

# Fig. S84 Fragment of ^1^H-^13^C HMBC spectra of **9** showing correlation between atom C-6” and protons H-4”a and H-4”b.

# Fig. S85 Fragment of ^1^H-^13^C HMBC spectra of **9** showing correlation between atom C-26 and protons H-11 and H-12.

# Fig. S86 FT-IR spectrum of compound **10** (in KBr).

**[10+ Na]** **^+^**

**[10-H]** **^-^**

# Fig. S87 ESI-MS spectrum of compound **10**.

# Fig. S88 ^1^H NMR spectra of compound **10** in DMSO-d_6_.

# Fig. S89 ^13^C NMR spectra of compound **10** in DMSO-d_6_.

# Fig. S90 ^1^H-^13^C HSQC spectra of compound **10** in DMSO-d_6_.

# Fig. S91 ^1^H-^13^C HMBC spectra of compound **10** in DMSO-d_6_.

# Fig. S92 ^1^H-^1^H COSY spectra of compound **10** in DMSO-d_6_.

# Fig. S93 ^1^H-^1^H ROESY spectra of compound **10** in DMSO-d_6_.

# Fig. S94 Fragment of ^13^C NMR spectrum of **10** showing singals from internal/external salt with TEA.

# Fig. S95 Fragment of ^1^H-^13^C HSQC NMR spectrum of compound **10** showing presence of internal/external salt with TEA.

# Fig. S96 FT-IR spectrum of compound **11** (in KBr).

**[11+ Na]** **^+^**

**[11-H]** **^-^**

# Fig. S97 ESI-MS spectrum of compound **11**.

# Fig. S98 ^1^H NMR spectra of compound **11** in DMSO-d_6_.

# Fig. S99 ^13^C NMR spectra of compound **11** in DMSO-d_6_.

# Fig. S100 ^1^H-^13^C HSQC spectra of compound **11** in DMSO-d_6_.

# Fig. S101 ^1^H-^13^C HMBC spectra of compound **11** in DMSO-d_6_.

# Fig. S102 ^1^H-^1^H COSY spectra of compound **11** in DMSO-d_6_.

# Fig. S103 ^1^H-^1^H ROESY spectra of compound **11** in DMSO-d_6_.

# Fig. S104 FT-IR spectrum of compound **12** (in KBr).

**[12+ Na]** **^+^**

**[12+ H]** **^+^**

**[12-H]** **^-^**

# Fig. S105 ESI-MS spectrum of compound **12**.

# Fig. S106 ^1^H NMR spectra of compound **12** in DMSO-d_6_.

# Fig. S107 ^13^C NMR spectra of compound **12** in DMSO-d_6_.

# Fig. S108 ^1^H-^13^C HSQC spectra of compound **12** in DMSO-d_6_.

# Fig. S109 ^1^H-^13^C HMBC spectra of compound **12** in DMSO-d_6_.

# Fig. S110 ^1^H-^1^H COSY spectra of compound **12** in DMSO-d_6_.

# Fig. S111 ^1^H-^1^H ROESY spectra of compound **12** in DMSO-d_6_.

Fig. S112 Structure of **12** containing functionalized benzyl substituent part being in agreement with ^1^H-^1^H ROESY contacts, noted in the spectra recorded in dmso-d_6_ solutions [*Scigress* FJ. 2.6 EU 3.1.9, Fujitsu 2008-2026; method B88 LYP (GGA)].

# Fig. S113 FT-IR spectrum of compound **13** (in KBr).

**[13+ Na]** **^+^**

**[13+ H]** **^+^**

**[13-H]** **^-^**

# Fig. S114 ESI-MS spectrum of compound **13**.

# Fig. S115 ^1^H NMR spectra of compound **13** in DMSO-d_6_.

# Fig. S116 ^13^C NMR spectra of compound **13** in DMSO-d_6_.

# Fig. S117 ^1^H-^13^C HSQC spectra of compound **13** in DMSO-d_6_.

# Fig. S118 ^1^H-^13^C HMBC spectra of compound **13** in DMSO-d_6_.

# Fig. S119 ^1^H-^1^H COSY spectra of compound **13** in DMSO-d_6_.

# Fig. S120 ^1^H-^1^H ROESY spectra of compound **13** in DMSO-d_6_.

# Fig. S121 FT-IR spectrum of compound **14** (in KBr).

**[14+ Na]** **^+^**

**[14+ H]** **^+^**

**[14-H]** **^-^**

# Fig. S122 ESI-MS spectrum of compound **14**.

# Fig. S123 ^1^H NMR spectra of compound **14** in DMSO-d_6_.

# Fig. S124 ^13^C NMR spectra of compound **14** in DMSO-d_6_.

# Fig. S125 ^1^H-^13^C HSQC spectra of compound **14** in DMSO-d_6_.

# Fig. S126 ^1^H-^13^C HMBC spectra of compound **14** in DMSO-d_6_.

# Fig. S127 ^1^H-^1^H COSY spectra of compound **14** in DMSO-d_6_.

# Fig. S128 ^1^H-^1^H ROESY spectra of compound **14** in DMSO-d_6_.

# Fig. S129 FT-IR spectrum of compound **15** (in KBr).

**[15+ H]** **^+^**

**[15-H]** **^-^**

# Fig. S130 ESI-MS spectrum of compound **15**.

# Fig. S131 ^1^H NMR spectra of compound **15** in DMSO-d_6_.

# Fig. S132 ^13^C NMR spectra of compound **15** in DMSO-d_6_.

# Fig. S133 ^1^H-^13^C HSQC spectra of compound **15** in DMSO-d_6_.

# Fig. S134 ^1^H-^13^C HMBC spectra of compound **15** in DMSO-d_6_.

# Fig. S135 ^1^H-^1^H COSY spectra of compound **15** in DMSO-d_6_.

# Fig. S136 ^1^H-^1^H ROESY spectra of compound **15** in DMSO-d_6_.

# Fig. S137 FT-IR spectrum of compound **16** (in KBr).

**[16+ Na]** **^+^**

**[16+ H]** **^+^**

**[16-H]** **^-^**

# Fig. S138 ESI-MS spectrum of compound **16**.

# Fig. S139 ^1^H NMR spectra of compound **16** in DMSO-d_6_.

# Fig. S140 ^13^C NMR spectra of compound **16** in DMSO-d_6_.

# Fig. S141 ^1^H-^13^C HSQC spectra of compound **16** in DMSO-d_6_.

# Fig. S142 ^1^H-^13^C HMBC spectra of compound **16** in DMSO-d_6_.

# Fig. S143 ^1^H-^1^H COSY spectra of compound **16** in DMSO-d_6_.

# Fig. S144 ^1^H-^1^H ROESY spectra of compound **16** in DMSO-d_6_.

# Fig. S145 FT-IR spectrum of compound **17** (in KBr).

**[17-H]** **^-^**

**[17+ H]** **^+^**

# Fig. S146 ESI-MS spectrum of compound **17**.

# Fig. S147 ^1^H NMR spectra of compound **17** in DMSO-d_6_.

# Fig. S148 ^13^C NMR spectra of compound **17** in DMSO-d_6_.

# Fig. S149 ^1^H-^13^C HSQC spectra of compound **17** in DMSO-d_6_.

# Fig. S150 ^1^H-^13^C HMBC spectra of compound **17** in DMSO-d_6_.

# Fig. S151 ^1^H-^1^H COSY spectra of compound **17** in DMSO-d_6_.

# Fig. S152 ^1^H-^1^H ROESY spectra of compound **17** in DMSO-d_6_.

# Fig. S153 FT-IR spectrum of compound **18** (in KBr).

**[18+ H]** **^+^**

**[18+ Na]** **^+^**

**[18-H]** **^-^**

# Fig. S154 ESI-MS spectrum of compound **18**.

# Fig. S155 ^1^H NMR spectra of compound **18** in DMSO-d_6_.

# Fig. S156 ^13^C NMR spectra of compound **18** in DMSO-d_6_.

# Fig. S157 ^1^H-^13^C HSQC spectra of compound **18** in DMSO-d_6_.

# Fig. S158 ^1^H-^13^C HMBC spectra of compound **18** in DMSO-d_6_.

# Fig. S159 ^1^H-^1^H COSY spectra of compound **18** in DMSO-d_6_.

# Fig. S160 ^1^H-^1^H ROESY spectra of compound **18** in DMSO-d_6_.

Fig. S161 Structure of 18 derivative containing functionalized antracene substituent part being in agreement with ^1^H-^1^H ROESY contacts, noted in the spectra recorded in dmso-d_6_ solutions [*Scigress* FJ. 2.6 EU 3.1.9, Fujitsu 2008-2026; method B88 LYP (GGA)].

# Fig. S162 FT-IR spectrum of compound **19** (in KBr).

**[19+ H]** **^+^**

**[19+ Na]** **^+^**

**[19-H]** **^-^**

# Fig. S163 ESI-MS spectrum of compound **19**.

# Fig. S164 ^1^H NMR spectra of compound **19** in DMSO-d_6_.

# Fig. S165 ^13^C NMR spectra of compound **19** in DMSO-d_6_.

# Fig. S166 ^1^H-^13^C HSQC spectra of compound **19** in DMSO-d_6_.

# Fig. S167 ^1^H-^13^C HMBC spectra of compound **19** in DMSO-d_6_.

# Fig. S168 ^1^H-^1^H COSY spectra of compound **19** in DMSO-d_6_.

# Fig. S169 ^1^H-^1^H ROESY spectra of compound **19** in DMSO-d_6_.

# Fig. S170 FT-IR spectrum of compound **20** (in KBr).

**[20-H]** **^-^**

**[20+ Na]** **^+^**

**[20+ H]** **^+^**

# Fig. S171 ESI-MS spectrum of compound **20**.

# Fig. S172 ^1^H NMR spectra of compound **20** in DMSO-d_6_.

# Fig. S173 ^13^C NMR spectra of compound **20** in DMSO-d_6_.

# Fig. S174 ^1^H-^13^C HSQC spectra of compound **20** in DMSO-d_6_.

# Fig. S175 ^1^H-^13^C HMBC spectra of compound **20** in DMSO-d_6_.

# Fig. S176 ^1^H-^1^H COSY spectra of compound **20** in DMSO-d_6_.

# Fig. S177 ^1^H-^1^H ROESY spectra of compound **20** in DMSO-d_6_.

# Fig. S178 FT-IR spectrum of compound **21** (in KBr).

**[21-H]** **^-^**

**[21+ Na]** **^+^**

**[21+ H]** **^+^**

# Fig. S179 ESI-MS spectrum of compound **21**.

# Fig. S180 ^1^H NMR spectra of compound **21** in DMSO-d_6_.

# Fig. S181 ^13^C NMR spectra of compound **21** in DMSO-d_6_.

# Fig. S182 ^1^H-^13^C HSQC spectra of compound **21** in DMSO-d_6_.

# Fig. S183 ^1^H-^13^C HMBC spectra of compound **21** in DMSO-d_6_.

# Fig. S184 ^1^H-^1^H COSY spectra of compound **21** in DMSO-d_6_.

# Fig. S185 ^1^H-^1^H ROESY spectra of compound **21** in DMSO-d_6_.

# Fig. S186 FT-IR spectrum of compound **22** (in KBr).

**[22-H]** **^-^**

**[22+ H]** **^+^**

**[22+ Na]** **^+^**

# Fig. S187 ESI-MS spectrum of compound **22**.

# Fig. S188 ^1^H NMR spectra of compound **22** in DMSO-d_6_.

# Fig. S189 ^13^C NMR spectra of compound **22** in DMSO-d_6_.

# Fig. S190 ^1^H-^13^C HSQC spectra of compound **22** in DMSO-d_6_.

# Fig. S191 ^1^H-^13^C HMBC spectra of compound **22** in DMSO-d_6_.

# Fig. S192 ^1^H-^1^H COSY spectra of compound **22** in DMSO-d_6_.

# Fig. S193 ^1^H-^1^H ROESY spectra of compound **22** in DMSO-d_6_.

# Fig. S194 FT-IR spectrum of compound **23** (in KBr).

**[23-H]** **^-^**

**[23+ Na]** **^+^**

**[23+ H]** **^+^**

# Fig. S195 ESI-MS spectrum of compound **23**.

# Fig. S196 ^1^H NMR spectra of compound **23** in DMSO-d_6_.

# Fig. S197 ^13^C NMR spectra of compound **23** in DMSO-d_6_.

# Fig. S198 ^1^H-^13^C HSQC spectra of compound **23** in DMSO-d_6_.

# Fig. S199 ^1^H-^13^C HMBC spectra of compound **23** in DMSO-d_6_.

# Fig. S200 ^1^H-^1^H COSY spectra of compound **23** in DMSO-d_6_.

# Fig. S201 ^1^H-^1^H ROESY spectra of compound **23** in DMSO-d_6_.

# Fig. S202 FT-IR spectrum of compound **24** (in KBr).

**[24-H]** **^-^**

**[24+ H]** **^+^**

# Fig. S203 ESI-MS spectrum of compound **24**.

# Fig. S204 ^1^H NMR spectra of compound **24** in DMSO-d_6_.

# Fig. S205 ^13^C NMR spectra of compound **24** in DMSO-d_6_.

# Fig. S206 ^1^H-^13^C HSQC spectra of compound **24** in DMSO-d_6_.

# Fig. S207 ^1^H-^13^C HMBC spectra of compound **24** in DMSO-d_6_.

# Fig. S208 ^1^H-^1^H COSY spectra of compound **24** in DMSO-d_6_.

# Fig. S209 ^1^H-^1^H ROESY spectra of compound **24** in DMSO-d_6_.

# Fig. S210 FT-IR spectrum of compound **25** (in KBr).

# Fig. S211 ESI-MS spectrum of compound **25**.

# Fig. S212 ^1^H NMR spectra of compound **25** in DMSO-d_6_.

# Fig. S213 ^13^C NMR spectra of compound **25** in DMSO-d_6_.

# Fig. S214 ^1^H-^13^C HSQC spectra of compound **25** in DMSO-d_6_.

# Fig. S215 ^1^H-^13^C HMBC spectra of compound **25** in DMSO-d_6_.

# Fig. S216 ^1^H-^1^H COSY spectra of compound **25** in DMSO-d_6_.

# Fig. S217 ^1^H-^1^H ROESY spectra of compound **25** in DMSO-d_6_.

Fig. S218 Shielding of H3’ proton signal in 1H NMR spectra of aryl-type derivatives **7**, **12** and **15** *vs*. deshielding of this signal in the spectra of **PIM** and its alkyl-type derivatives (monoalkyl as compound **21** and dialkyl compound **23**, suggesting the arrangement of the introduced substituent at the nitrogen relative to mycosamine moiety (all recorded in dmso-*d_6_*+ traces of water).

# ITC titration of ergosterol solutions with PIM and its derivatives solutions in DMSO

**Results**

The thermal effects of titration of ergosterol (20 μM) with 9 polyene-type ligands (1000 μM) were carried out in DMSO solution at 25℃ (Fig. S219), using the isothermal titration calorimetry. Heat effects of direct interactions of 9 ligands (**PIM, 7, 12, 15, 17, 19, 21, 23** and **25**) with **ERG** in DMSO were described (Origin MicroCal) with multi-parameter non-linear regression (Fig. S220) using One Set of Sites model. The obtained stoichiometries, binding and dissociation constants and standard thermodynamic functions were summarized in Table 4.

| **A) PIM** | |
| --- | --- |
|  |  |
|  |  |
| **B) 7** | |
|  |  |
|  |  |
| **C) 12** | |
|  |  |
|  |  |
| **D) 15** | |
|  |  |
|  |  |
| **E) 17** | |
|  |  |
|  |  |
| **F) 19** | |
|  |  |
|  |  |
| **G) 21** | |
|  |  |
|  |  |
| **H) 23** | |
|  |  |
|  |  |
| **I) 25** | |
|  |  |
|  |  |

Fig. S219 Thermal effects of 20 μM **ERG** solution titrated with 1000 μM ligand solution (■) as a function of molar ratio of ligand to Erg and corresponded to them thermal effects of ligand dilution (●) in DMSO at 25℃ for: A) **PIM**, B) **7**, C) **12**, D) **15**, E) **17**, F) **19**, G) **21**, H) **23**, I) **25**.

Fig. S220 Thermal effects of the direct interaction between **ERG** and ligands in DMSO at 25℃ described with One Set of Sites model (solid lines) for selected ligands.

The obtained results (Table 4) indicate that **ERG** binds with studied ligands in the thermodynamically spontaneous way ($\Delta G<0$). The driving force for **PIM**, **15**, **19** and **23** complexation is both exothermic enthalpy ($\Delta H<0$) and positive entropy of binding ($\Delta S$>0), whereas in the case of **7** and **17** unfavorable endothermic enthalpy of interactions ($\Delta H>0$) is overcome with positive entropy of binding (∆S>0). The binding constants of ergosterol complexed with studied ligands are high (logK>3) and show the formation of stable supramolecular complexes.

Fig. S221 Fragment of 1H NMR spectrum of mixture of compound **7** and **ERG (6:1**), showing signals of exchangeable protons at the nitrogen of mycosamine (spectrum in the range 10.03 to 10.7 ppm) and signal of nitrophenol group.

Fig. S222 Fragment of 1H NMR and 13C NMR spectrum of mixture (**7: ERG**) showing conserved positions of proton H(3) (δ=6.26 ppm) and carbon atom C(1) (δ=164.3 ppm).

Fig. S223 Model of Internal/ external salt of compound **10** being in agreement with ^1^H-^1^H ROESY contacts, noted in the spectra recorded in dmso-d_6_ solutions [*Scigress* FJ. 2.6 EU 3.1.9, Fujitsu 2008-2026; method B88 LYP (GGA)].

# Fig. S224 FT-IR spectrum of **PIM**, **7**, **2** showing absorption continuum measured in DMSO (with traces of water).

# Table S1 Summary of chemical C atoms shifts [ppm] for **PIM** and derivatives **1**-**14** from ^13^C NMR spectra.

| **Compound**    **ATOM NUMBER** | **PIM** | **1** | **2** | **3** | **4** | **5** | **6** | **7** | **8** | **9** | **10** | **11** | **12** | **13** | **14** |
| --- | --- | --- | --- | --- | --- | --- | --- | --- | --- | --- | --- | --- | --- | --- | --- |
| **1** | 164.3 | 164.3 | 164.3 | 164.3 | 164.3 | 164.3 | 164.3 | 164.3 | 164.3 | 164.3 | 164.3 | 164.3 | 164.3 | 164.3 | 164.3 |
| **2** | 124.5 | 124.5 | 124.4 | 124.5 | 124.4 | 124.4 | 124.4 | 124.4 | 124.4 | 124.3 | 124.4 | 124.5 | 124.4 | 124.5 | 124.5 |
| **3** | 144.4 | 144.4 | 144.4 | 144.4 | 144.4 | 144.4 | 144.4 | 144.4 | 144.4 | 144.4 | 144.5 | 144.4 | 144.4 | 144.4 | 144.4 |
| **4** | 53.6 | 53.7 | 53.7 | 53.7 | 53.7 | 53.7 | 53.7 | 53.5 | 53.6 | 53.7 | 54.8 | 53.7 | 53.7 | 54.7 | 53.7 |
| **5** | 57.9 | 57.9 | 57.8 | 57.8 | 57.8 | 57.9 | 57.9 | 57.8 | 57.8 | 57.9 | 57.8 | 57.9 | 57.8 | 57.9 | 57.6 |
| **6** | 40.8 | 40.7 | 40.8 | 40.8 | 40.7 | 40.8 | 40.8 | 40.7 | 40.8 | 40.8 | 40.7 | 40.8 | 40.8 | 40.8 | 40.8 |
| **7** | 66.1 | 66.0 | 65.9 | 65.9 | 65.9 | 66.0 | 65.9 | 66.1 | 66.0 | 66.0 | 65.8 | 66.0 | 66.0 | 65.9 | 66.0 |
| **8** | 46.4 | 46.3 | 46.4 | 46.3 | 46.3 | 46.4 | 46.4 | 46.2 | 46.2 | 46.2 | 46.8 | 46.3 | 46.3 | 46.3 | 46.3 |
| **9** | 96.9 | 97.1 | 97.0 | 97.1 | 97.1 | 97.0 | 97.2 | 97.0 | 97.0 | 97.0 | 96.8 | 97.0 | 97.2 | 97.2 | 97.2 |
| **10** | 44.2 | 44.1 | 44.1 | 44.1 | 44.1 | 44.1 | 44.1 | 44.2 | 44.1 | 44.1 | 43.6 | 44.1 | 44.1 | 44.1 | 44.1 |
| **11** | 65.2 | 65.2 | 65.2 | 65.1 | 65.0 | 65.1 | 65.2 | 65.5 | 66.0 | 65.0 | 64.7 | 65.1 | 65.2 | 65.1 | 65.2 |
| **12** | 58.1 | 57.1 | 57.7 | 57.0 | 57.0 | 56.9 | 56.9 | 58.00 | 57.3 | 57.2 | 57.7 | 57.1 | 57.1 | 57.1 | 57.1 |
| **13** | 65.0 | 65.1 | 65.2 | 64.7 | 64.7 | 65.2 | 64.8 | 64.9 | 64.9 | 65.2 | 65.5 | 64.7 | 65.1 | 65.2 | 64.7 |
| **14** | 36.3 | 36.9 | 37.2 | 37.0 | 36.9 | 37.1 | 37.3 | 36.4 | 36.9 | 36.8 | 37.9 | 36.9 | 37.1 | 36.9 | 37.0 |
| **15** | 74.2 | 74.3 | 74.5 | 74.3 | 74.3 | 74.5 | 74.6 | 74.4 | 74.4 | 74.2 | 75.6 | 74.3 | 74.3 | 74.2 | 74.4 |
| **16** | 136.3 | 136.3 | 136.5 | 136.3 | 136.3 | 136.4 | 136.4 | 135.6 | 136.2 | 136.1 | 137.3 | 136.3 | 136.4 | 136.3 | 136.3 |
| **17** | 128.3 | 128.5 | 128.3 | 128.2 | 128.4 | 128.4 | 128.4 | 128.6 | 128.5 | 128.0 | 127.8 | 127.9 | 128.4 | 128.4 | 128.4 |
| **18** | 133.4 | 133.4 | 133.5 | 133.4 | 133.4 | 133.5 | 133.5 | 133.2 | 133.4 | 133.3 | 133.7 | 133.4 | 133.4 | 133.4 | 133.4 |
| **19** | 131.1 | 131.1 | 131.0 | 131.0 | 131.1 | 131.1 | 131.1 | 131.4 | 130.9 | 131.2 | 132.0 | 130.9 | 131.3 | 131.3 | 131.1 |
| **20** | 131.9 | 131.8 | 131.9 | 131.9 | 131.9 | 131.9 | 131.9 | 131.8 | 131.9 | 131.8 | 131.0 | 131.3 | 131.0 | 131.9 | 131.9 |
| **21** | 131.3 | 131.3 | 131.2 | 131.3 | 131.3 | 131.3 | 131.3 | 133.4 | 131.3 | 131.3 | 130.6 | 131.9 | 131.9 | 131.1 | 131.3 |
| **22** | 135.4 | 135.4 | 135.4 | 135.4 | 135.4 | 135.4 | 135.4 | 135.6 | 135.4 | 135.3 | 135.4 | 135.4 | 135.4 | 135.4 | 135.4 |
| **23** | 128.5 | 128.4 | 128.4 | 128.3 | 128.5 | 128.5 | 128.5 | 128.4 | 128.4 | 128.0 | 128.2 | 128.3 | 128.4 | 128.5 | 128.5 |
| **24** | 38.8* | 38.9* | 38.8* | 39.0* | 39.0* | 38.9* | 39.0* | 38.9* | 38.7* | 38.8* | 38.6* | 38.6* | 38.7* | 38.7* | 38.7* |
| **25** | 69.50 | 69.5 | 69.5 | 69.6 | 69.5 | 69.5 | 69.5 | 69.5 | 69.5 | 69.5 | 69.4 | 69.5 | 69.5 | 69.5 | 69.5 |
| **26** | 175.1 | 174.0 | 174.3 | 174.0 | 174.0 | 174.0 | 174.6 | 172.6 | 174.3 | 173.8 | 177.6 | 174.0 | 174.1 | 174.0 | 174.1 |
| **27** | 20.1 | 20.1 | 20.0 | 20.0 | 20.0 | 20.0 | 20.1 | 20.1 | 20.0 | 20.0 | 19.9 | 20.0 | 20.1 | 20.1 | 20.1 |
| **1’** | 95.7 | 97.1 | 97.3 | 97.0 | 97.0 | 97.2 | 97.00 | 96.3 | 96.9 | 97.1 | 98.3 | 97.2 | 97.0 | 97.1 | 97.1 |
| **2’** | 68.1 | 66.1 | 66.1 | 66.2 | 66.1 | 66.2 | 66.3 | 65.2 | 65.2 | 66.1 | 66.3 | 66.1 | 65.9 | 66.0 | 65.7 |
| **3’** | 56.2 | 62.4 | 62.5 | 62.5 | 62.4 | 62.4 | 62.4 | 60.7 | 61.9 | 62.4 | 62.4 | 62.4 | 62.4 | 62.5 | 62.4 |
| **4’** | 70.4 | 70.9 | 70.9 | 70.9 | 70.9 | 70.9 | 71.0 | 69.5 | 70.5 | 70.7 | 70.8 | 70.9 | 70.9 | 70.8 | 70.7 |
| **5’** | 72.7 | 73.0 | 73.0 | 73.0 | 73.0 | 73.0 | 73.0 | 72.8 | 73.1 | 73.0 | 72.7 | 73.0 | 73.0 | 73.0 | 72.9 |
| **6’** | 17.8 | 18.0 | 18.0 | 18.0 | 18.0 | 18.0 | 18.0 | 17.8 | 17.9 | 17.9 | 18.0 | 18.0 | 18.0 | 18.0 | 18.0 |
| **1”** | - | 49.4 | 49.1 | 43.0 | 49.0 | 49.1 | 49.1 | 44.1 | 46.3 | 49.4 | 49.5 | 49.4 | 49.3 | 49.5 | 45.0 |
| **2”** | - | 142.1 | 141.6 | 124.4 | 144.1 | 146.0 | 149.6 | 135.3 | 127.7 | 147.5 | 139.9 | 140.9 | 139.3 | 137.9 | 128.4 |
| **3”** | - | - | 127.0 | 160.3 | 114.3 | - | - | 155.0 | 156.6 | - | 130.1 | - | - | - | 156.6 |
| **3”a** | - | 128.4 | - | - | - | 127.8 | 123.0 | - | - | 128.0 | - | 131.0 | 128.4 | 127.8 | - |
| **3”b** |  | 128.4 | - | - | - | 127.8 | 123.0 | - | - | 128.0 | - | 131.0 | 128.4 | 127.8 | - |
| **4”** | - | - | 121.4 | 130.2 | 162.2 | - | - | 117.7 | 117.7 | - | 129.8 | - | - | - | 111.9 |
| **4”a** | - | 131.9 | - | - | - | 129.4 | 128.9 | - | - | 129.1 | - | 128.4 | 125.8 | 127.9 | - |
| **4”b** | - | 131.9 | - | - | - | 129.4 | 128.9 | - | - | 129.1 | - | 128.4 | 125.8 | 127.9 | - |
| **5”** | - | 119.8 | 129.6 | 124.1 | 113.1 | 123.8 | 146.2 | 125.7 | 130.1 | 129.0 | 127.6 | 121.3 | 132.4 | 140.2 | 127.9 |
| **5”a** | - | - | - | - | - | - | - | - | - | - | - | - | - | - | - |
| **5”b** | - | - | - | - | - | - | - | - | - | - | - | - | - | - | - |
| **6”** | - | 83.5 | 128.5 | 127.7 | 129.7 | 124.7 | - | 138.6 | 109.2 | 167.2 | 127.3 | 81.0 | 138.1 | 36.8 | 120.2 |
| **6”a** | - | - | - | - | - | - | - | - | - | - | - | - | - | - | - |
| **6”b** | - | - | - | - | - | - | - | - | - | - | - | - | - | - | - |
| **7”** | - | 80.1 | 129.7 | 114.7 | 123.7 | - | - | 126.7 | 131.1 | - | 129.9 | 87.8 | - | 23.9 | 129.4 |
| **7”a** | - | - | - | - | - | - | - | - | - | - | - | - | 127.5 | - | - |
| **7”b** | - | - | - | - | - | - | - | - | - | - | - | - | 127.5 | - | - |
| **8”** | - | - | 83.6 | - | - | - | - | - | - | - | 177.6 | 23.2 | - | 13.5 | - |
| **8”a** | - | - | - | - | - | - | - | - | - | - | - | - | 114.3 | - | - |
| **8”b** | - | - | - | - | - | - | - | - | - | - | - | - | 114.3 | - | - |
| **9”** | - | - | 80.1 | - | - | - | - | - | - | - | - | 59.7 | 158.7 | - | - |
| **10”** | - | - | - | - | - | - | - | - | - | - | - | - | 55.1 | - | - |
| **11”** | - | - | - | - | - | - | - | - | - | - | - | - | - | - | - |
| **12”** | - | - | - | - | - | - | - | - | - | - | - | - | - | - | - |
| **13”** | - | - | - | - | - | - | - | - | - | - | - | - | - | - | - |
| **14”** | - | - | - | - | - | - | - | - | - | - | - | - | - | - | - |
| **15”** | - | - | - | - | - | - | - | - | - | - | - | - | - | - | - |
| **16”** | - | - | - | - | - | - | - | - | - | - | - | - | - | - | - |
| **17”** | - | - | - | - | - | - | - | - | - | - | - | - | - | - | - |
| **N-mycosamine** |  |  | -342.2^ |  |  |  |  | -344.3^ |  |  |  |  |  |  |  |

*-overlapped, ^- determined from ^1^H-^15^N gHMBC NMR

# Table S2 Summary of chemical C atoms shifts [ppm] for derivatives **15-25** from ^13^C NMR spectra.

| **Compound**  **ATOM NUMBER** | **15** | **16** | **17** | **18** | **19** | **20** | **21** | **22** | **23** | **24** | **25** |
| --- | --- | --- | --- | --- | --- | --- | --- | --- | --- | --- | --- |
| **1** | 164.3 | 164.3 | 164.3 | 164.3 | 164.3 | 164.3 | 164.3 | 164.3 | 164.3 | 164.3 | 164.3 |
| **2** | 124.4 | 124.4 | 124.4 | 124.4 | 124.5 | 124.5 | 124.4 | 124.4 | 124.5 | 124.4 | 124.4 |
| **3** | 144.4 | 144.4 | 144.4 | 144.4 | 144.4 | 144.4 | 144.4 | 144.4 | 144.4 | 144.4 | 144.4 |
| **4** | 53.7 | 53.6 | 54.7 | 53.7 | 53.6 | 53.6 | 53.6 | 53.6 | 53.5 | 53.7 | 53.6 |
| **5** | 57.8 | 57.9 | 57.9 | 57.3 | 57.9 | 57.8 | 57.9 | 57.9 | 57.5 | 57.9 | 57.9 |
| **6** | 40.8 | 40.8 | 40.8 | 40.8 | 40.8 | 40.8 | 40.8 | 40.8 | 40.7 | 40.8 | 40.8 |
| **7** | 65.9 | 66.0 | 65.9 | 66.8 | 66.0 | 65.8 | 66.0 | 66.0 | 66.1 | 66.0 | 66.1 |
| **8** | 46.3 | 46.2 | 46.3 | 46.2 | 46.3 | 46.2 | 46.4 | 46.4 | 46.3 | 46.3 | 46.3 |
| **9** | 97.1 | 97.1 | 97.2 | 97.1 | 97.0 | 97.0 | 97.0 | 97.00 | 97.0 | 97.1 | 96.9 |
| **10** | 44.1 | 44.1 | 44.1 | 44.2 | 44.2 | 44.1 | 44.1 | 44.1 | 44.2 | 44.2 | 44.6 |
| **11** | 65.2 | 65.1 | 65.2 | 65.2 | 64.7 | 65.2 | 65.2 | 65.3 | 65.3 | 65.1 | 64.6 |
| **12** | 57.1 | 57.2 | 57.1 | 57.3 | 57.3 | 57.2 | 57.4 | 57.4 | 57.9 | 57.0 | 57.1 |
| **13** | 65.1 | 64.9 | 65.1 | 65.0 | 65.0 | 65.0 | 65.1 | 65.1 | 65.1 | 65.1 | 65.1 |
| **14** | 37.0 | 36.5 | 37.0 | 36.7 | 36.7 | 36.8 | 36.7 | 36.7 | 36.6 | 36.9 | 36.3 |
| **15** | 74.3 | 74.2 | 74.3 | 74.1 | 74.2 | 74.1 | 74.3 | 74.3 | 74.1 | 74.1 | 74.1 |
| **16** | 136.2 | 135.7 | 136.3 | 136.2 | 136.3 | 136.3 | 136.2 | 136.2 | 136.2 | 136.3 | 136.3 |
| **17** | 128.5 | 128.1 | 128.2 | 128.5 | 128.4 | 128.3 | 128.4 | 128.4 | 128.4 | 128.4 | 128.2 |
| **18** | 133.3 | 133.3 | 133.4 | 133.4 | 133.4 | 133.4 | 133.4 | 133.4 | 133.4 | 133.4 | 133.4 |
| **19** | 131.3 | 131.2 | 131.9 | 131.3 | 131.3 | 131.3 | 131.3 | 131.3 | 131.1 | 131.1 | 131.1 |
| **20** | 131.1 | 131.3 | 132.9 | 131.0 | 131.9 | 131.9 | 131.1 | 131.1 | 131.9 | 131.9 | 131.9 |
| **21** | 131.9 | 131.8 | 132.0 | 131.2 | 131.1 | 131.1 | 131.9 | 131.9 | 131.3 | 131.3 | 131.3 |
| **22** | 135.4 | 135.7 | 135.4 | 135.4 | 135.4 | 135.4 | 135.4 | 135.4 | 135.4 | 135.4 | 135.4 |
| **23** | 128.5 | 128.5 | 128.5 | 128.7 | 128.5 | 128.5 | 128.5 | 128.5 | 128.5 | 128.5 | 128.5 |
| **24** | 38.7* | 38.8* | 38.7* | 38.7* | 38.9* | 38.7* | 38.7* | 38.8* | 38.7* | 38.8* | 38.8* |
| **25** | 69.6 | 69.5 | 69.5 | 69.5 | 69.5 | 69.5 | 69.6 | 69.5 | 69.6 | 69.5 | 69.5 |
| **26** | 174.0 | 173.9 | 174.1 | 174.0 | 174.4 | 174.1 | 174.9 | 174.8 | 174.7 | 174.1 | 171.4 |
| **27** | 20.1 | 20.1 | 20.1 | 20.1 | 20.1 | 20.0 | 20.0 | 20.0 | 20.1 | 20.1 | 20.1 |
| **1’** | 96.8 | 96.8 | 97.1 | 97.0 | 96.6 | 96.8 | 96.3 | 96.3 | 96.3 | 97.0 | 96.3 |
| **2’** | 65.7 | 65.7 | 66.1 | 66.1 | 65.7 | 66.0 | 65.5 | 65.5 | 65.6 | 68.6 | 65.3 |
| **3’** | 62.2 | 62.4 | 62.5 | 63.7 | 62.1 | 62.9 | 62.3 | 62.3 | 62.2 | 62.2 | 61.1 |
| **4’** | 70.6 | 69.7 | 70.8 | 70.8 | 70.2 | 70.5 | 69.8 | 69.8 | 69.9 | 67.4 | 69.8 |
| **5’** | 72.9 | 72.9 | 73.0 | 73.2 | 72.8 | 72.9 | 72.6 | 72.7 | 72.7 | 73.7 | 72.7 |
| **6’** | 17.9 | 17.8 | 18.0 | 18.0 | 17.9 | 17.9 | 17.9 | 17.9 | 17.9 | 17.9 | 17.9 |
| **1”** | 48.9 | 49.7 | 49.8 | 42.6 | 50.3 | 51.9 | 45.4 | 45.3 | 45.2 | 44.1 | 48.2 |
| **2”** | 131.7 | 70.6 | 138.4 | 129.8 | 10.2 | 34.4 | 28.8 | 26.5 | 28.0 | 14.5 | 130.8 |
| **3”** | 114.1 | 71.8 | 127.4 | - | 3.44 | 26.7 | 28.9 | 29.0 | 26.5 | - | 114.1 |
| **3”a** | - | - | - | 132.1 | - | - | - | - | - | - | - |
| **3”b** | - | - | - | 132.1 | - | - | - | - | - | - | - |
| **4”** | 157.5 | 138.3 | 132.9 | - | 3.2 | 11.0 | 26.5 | 28.9 | 28.5 | - | 157.8 |
| **4”a** | - | - | - | 125.6 | - | - | - | - | - | - | - |
| **4”b** | - | - | - | 125.6 | - | - | - | - | - | - | - |
| **5”** | 114.1 | - | 125.9 | - | - | 17.4 | 26.5 | 28.8 | 28.6 | - | 114.1 |
| **5”a** | - | 127.5 | - | 124.6 | - | - | - | - | - | - | - |
| **5”b** | - | 127.5 | - | 124.6 | - | - | - | - | - | - | - |
| **6”** | 130.2 | - | 125.3 | - | - | - | 28.7 | 28.7* | 31.1 | - | 130.2 |
| **6”a** | - | 127.4 | - | 124.9 | - | - | - | - | - | - | - |
| **6”b** | - | 127.4 | - | 124.9 | - | - | - | - | - | - | - |
| **7”** | 129.3 | 126.8 | 125.3 | - | - | - | 28.5 | 28.7* | 21.9 | - | 129.8 |
| **7”a** | - | - | - | 128.5 | - | - | - | - | - | - | - |
| **7”b** | - | - | - | 128.5 | - | - | - | - | - | - | - |
| **8”** | 67.3 | - | 125.9 | - | - | - | 31.2 | 28.7 | 13.7 | - | - |
| **8”a** | - | - | - | 131.2 | - | - | - | - | - | - | - |
| **8”b** | - | - | - | 131.2 | - | - | - | - | - | - | - |
| **9”** | 28.5 | - | 132.9 | 125.9 | - | - | 21.9 | 28.5 | - | - | - |
| **10”** | 25.4 | - | 127.4 | - | - | - | 13.8 | 31.1 | - | - | - |
| **11”** | 28.6 | - | 126.8 | - | - | - | - | 21.9 | - | - | - |
| **12”** | 28.8 | - | - | - | - | - | - | 13.7 | - | - | - |
| **1”’** | 28.7 | - | - | - | - | - | - | - | 45.2 | 44.1 | 67.3 |
| **2”’** | 28.6 | - | - | - | - | - | - | - | 28.0 | 14.5 | 28.5 |
| **3”’** | 31.1 | - | - | - | - | - | - | - | 26.5 | - | 25.4 |
| **4”’** | 21.9 | - | - | - | - | - | - | - | 28.5 | - | 28.6 |
| **5”’** | 13.7 | - | - | - | - | - | - | - | 28.6 | - | 28.7 |
| **6”’** |  |  | - | - | - | - | - | - | 31.1 | - | 28.8 |
| **7”’** |  |  | - | - | - | - | - | - | 21.9 | - | 32.1 |
| **8”’** |  |  | - | - | - | - | - | - | 13.7 | - | 31.1 |
| **9”’** |  |  | - | - | - | - | - | - | - | - | 21.9 |
| **10”’** |  |  | - | - | - | - | - | - | - | - | 13.8 |

# Table S3 Summary of chemical H atoms shifts [ppm] for PIM and derivatives **1-5** from ^1^H NMR spectra.

|  | **PIM** | **1** | **2** | **3** | **4** | **5** | **6** |
| --- | --- | --- | --- | --- | --- | --- | --- |
| **2** | 6.13-6.11  m, 1H | 6.15-6.10  m, 1H | 6.14-6.10  m, 1H | 6.1-6.08  m, 1H | 6.09-6.05  m, 3H | 6.15-6.06  m, 3H | 6.15-6.06  m, 3H |
| **3** | 6.27  dd, *^3^J*_H2-H3_=15.8 Hz, *^3^J*_H3-H4_=7.7 Hz, 1H | 6.26  dd, *^3^J*_H3-H2_= 15.8 Hz, *^3^J*_H3-H4_=7.7 Hz, 1H | 6.27  dd,*^3^J*_H2-H3_=15.8Hz, *^3^J*_H3-H4_=7.7Hz, 1H | 6.28  dd, *^3^J*_H3-H2_= 15.8 Hz, *^3^J*_H3-H4_=7.7 Hz, 1H | 6.27  dd, *^3^J*_H3-H2_= 15.8 Hz, *^3^J*_H3-H4_=7.7 Hz, 1H | 6.27  dd, *^3^J*_H2-H3_ = 15.7 Hz, *^3^J*_H3-H4_ = 7.8 Hz, 1H, | 6.27  dd, *^3^J*_H2-H3_ = 15.7 Hz, *^3^J*_H3-H4_ = 7.8 Hz, 2H |
| **4** | 3.23-3.19  m, 2H | 3.22  dd, *^3^J*_H3-H4_= 7.7 Hz, *^4^J*_H2-H4_ = 2.0 Hz 1H | 3.22  dd, *^3^J*_H3-H4_= 7.7 Hz, *^4^J*_H2-H4_ = 2.0 Hz 1H | 3.22  dd, *^3^J*_H3-H4_ = 7.6 Hz, *^4^J*_H2-H4_ = 2.0 Hz, 1H | 3.22  dd, *^3^J*_H3-H4_= 7.6 Hz, *^4^J*_H2-H4_ = 2.0 Hz, 1H | 3.22  dd, *^3^J*_H3-H4_= 7.7 Hz, *^3^J*_H2-H4_ = 2.0 Hz, 1H | 3.22  dd, *^3^J*_H3-H4_= 7.7 Hz, *^4^J*_H2-H4_ = 2.0 Hz 1H |
| **5** | 2.79-2.74  m, 2H | 2.76-2.73  m, 1H | 2.77-2.74  m, 1H | 2.77-2.74  m, 1H | 2.77-2.73  m, 1H | 2.77-2.73  m, 1H | 2.76-2.73  m, 1H |
| **6a** | 1.16-1.12  m, 1H | 1.15-1.07  m, 3H | 1.13-1.06  m, 2H, | 1.15-1.06  m, 3H | 1.19-1.12  m,5H | 1.14-1.06  m, 2H | 1.14-1.06  m, 2H |
| **6b** | 1.98-1.93  m, 1H | 1.98-1.90  m, 3H | 1.96-1.92  m, 1H | 1.96-1.92  m,H | 1.92-1.89  m, 1H | 1.91-1.88  m, 1H | 1.96-1.85 m, 2H, |
| **7** | 4.16-4.12  m. 1H | 4.19-4.10  m, 4H | 4.17-4.13  m, 1H | 4.14-4.10  m, 1H | 4.17-4.13  m, 1H | 4.16-4.10  m, 1H | 4.16-4.10  m, 1H |
| **8a** | 1.57  dd, *^2^J*_H8a-H8b_=14.4 Hz, *^3^J*_H7-H8a_=10.4 Hz, 1H, | 1.50-1.48  m, 1H | 1.63-1.54 m, 6H | 1.59-1.53 m, 2H | 1.60-1.55  m, 2H | 1.63-1.53  m, 2H | 1.62-1.52  m, 3H |
| **8b** | 1.49-1.45  m, 2H | 1.51-1.48  m, 1H | 1.51 -1.47  m, 1H | 1.51  dd, *^2^J*_H8a-H8b_= 14.2 Hz, *^3^J*_H8b-H7_= 2.0 Hz 1H | 1.52  dd, *^2^J*_H8a-H8b_= 14.2 Hz, *^3^J*_H8b-H7_= 2.0 Hz 1H | 1.50-1.47  m, 1H | 1.62-1.52  m, 3H |
| **10a** | 1.85-1.78  m, 3H | 1.85  dd, *^2^J*_H10a-H10b_= 12.4 Hz, *^3^J*_H10a-H11_= 4.7 Hz, 1H | 1.85  dd, *^2^J*_H10a-H10b_= 12.4 Hz, *^3^J*_H10a-H11_= 4.9 Hz, 1H | 1.85 (dd, *^2^J*_H10a-H10b_= 10.8 Hz, *^3^J*_H10a-H11_= 4.9 Hz 1H | 1.85  dd, *^2^J*_H10a-H10b_=12.3 Hz, *^3^J*_H10a-H11_= 4.9 Hz 1H | 1.85  dd, *^2^J*_H10a-H10b_=12.4 Hz, *^3^J*_H10a-H11_ = 4.7 Hz, 1H | 1.85  dd, *^2^J*_H10a-H10b_=12.4 Hz, *^3^J*_H10a-H11_ = 4.7 Hz, 1H |
| **10b** | 1.10-1.07  m, 1H | 1.15-1.07  m, 3H | 1.13-1.06  m, 2H | 1.15-1.06 m, 3H | 1.19-1.12  m,5H | 1.14-1.06 m,2H | 1.14-1.06 m, 2H |
| **11** | 4.01  td, *^3^J*_H11-H12_ =10.7 Hz, *^3^J*_H11-H10a_ =4.7 Hz, 1H | 4.06-4.01  m, 1H | 3.99-3.95  m, 1H | 4.01-3.97  m, 1H | 4.02  d, *^3^J*_H10a-H11_=4.7 Hz, 1H | 4.02  td, *^3^J*_H12-H11_=10.7Hz, *^3^J*_H10a-H11_ = 4.6 Hz, 1H | 4.02  td, *^3^J*_H12-H11_=10.7Hz, *^3^J*_H11-H10a_ = 4.6 Hz, 1H |
| **12** | 1.85-1.78  m, 3H | 1.98-1.90  m, 3H | 1.88-1.86  m, 1H | 1.96-1.92  m, | 1.95-1.92  m, 1H | 1.94-1.92  m, 1H | 1.96-1.85  m, 2H |
| **13** | 4.21-4.18  m, 1H | 4.22-4.20  m, 1H | 4.25-4.18  m, 3H | 4.22-4.19  m, 1H | 4.25-4.19  m, 1H | 4.25-4.20  m, 2H | 4.25-4.20  m, 2H |
| **14a** | 2.24-2.15  m, 3H | 2.05-2.00  m, 1H | 2.11-2.07  m, 1H | 2.09-2.02  m, 1H | 2.05-2.00  m, 1H | 2.08-2.00  m, 1H | 2.15-2.08  m, 1H |
| **14b** | 1.49-1.45  m, 2H | 1.63-1.53  m, 2H | 1.63-1.54  m, 6H, | 1.59-1.53 m, 2H, | 1.60-1.55  m, 2H | 1.63-1.53  m, 2H | 1.62-1.52  m, 3H |
| **15** | 4.41-4.36  m, 1H | 4.40-4.36  m, 2H | 4.38-4.30  m, 3H | 4.38-4.35  d, *^3^J*_H15-H16_ =8.6 Hz, 2H, | 4.38  ddd, *^3^J*_H16-H15_ =8.3 Hz, *^3^J*_H14a-H15_ = 4.6 Hz, *^4^J*_H15-H17_ =2.7 Hz , 1H | 4.37-4.34  m, 4H | 4.37-4.34  m, 4H |
| **16** | 5.91  dd, *^3^J*_H16-H17_=15.3 Hz, *^3^J*_H15-H16_=8.8 Hz, 1H | 5.92-5.86  m, 1H | 5.90  dd, *^3^J*_H16-H17_=15.3 Hz, *^3^J*_H16-H15_=8.8 Hz,1H | 5.89  dd, *^3^J*_H16-H17_ = 15.3 Hz, *^3^J*_H15-H16_ = 8.7 Hz, 1H | 5.87-5.82  m, 1H | 5.89  dd, *^3^J*_H16-H17_ = 15.3 Hz, *^3^J*_H16-H15_ = 8.8 Hz, 1H | 5.89  dd, *^3^J*_H16-H17_ = 15.3 Hz, *^3^J*_H16-H15_ = 8.8 Hz, 1H |
| **17** | 6.10-6.01  m, 2H | 6.09-6.05  m, 2H | 6.09-6.05  m, 2H | 6.08-6.05  m, 2H | 6.09-6.05  m, 3H | 6.15-6.06  m, 3H | 6.15-6.06  m, 3H |
| **18** | 6.49  dd, *^3^J*_H18-H19_=14.6 Hz, *^3^J*_H18-H17_=10.8 Hz, 1H | 6.49  dd,^3^*J*_H18-H19_=14.6 Hz, ^3^*J*_H17-H18_=10.8 Hz 1H | 6.49  dd,^3^*J*_H18-H19_=14.7 Hz, ^3^*J*_H18-H17_=10.7 Hz 1H | 6.49  dd, *^3^J*_H18-H19_ = 14.7 Hz, *^3^J*_H17-H18_ = 10.7 Hz, 1H | 6.49  dd, *^3^J*_H18-H19_ = 14.6 Hz, *^3^J*_HH17-H18_ = 10.8 Hz, 1H | 6.49  dd, *^3^J*_H18-H19_ = 14.5Hz, *^3^J*_H18-H17_ = 10.8 Hz, 1H | 6.49  dd, *^3^J*_H18-H19_ = 14.5Hz, *^3^J*_H18-H17_ = 10.8 Hz, 1H |
| **19** | 6.24-6.16.  m, 3H | 6.21-6.16  m, 3H | 6.24-6.16.  m, 3H | 6.19-6.14  m, 3H | 6.21-6.14  m, 3H | 6.21-6.16  m, 3H | 6.21-6.16  m, 3H |
| **20** | 6.24-6.16.  m, 3H | 6.21-6.16  m, 3H | 6.24-6.16.  m, 3H | 6.19-6.14  m, 3H | 6.21-6.14  m, 3H | 6.21-6.16  m, 3H | 6.21-6.16  m, 3H |
| **21** | 6.24-6.16.  m, 3H | 6.21-6.16  m, 3H | 6.24-6.16.  m, 3H | 6.19-6.14  m, 3H | 6.21-6.14  m, 3H | 6.21-6.16  m, 3H | 6.21-6.16  m, 3H |
| **22** | 6.10-6.01  m, 2H | 6.09-6.05  m, 2H | 6.09-6.05  m, 2H | 6.08-6.05  m, 2H | 6.09-6.05  m, 3H | 6.15-6.06  m, 3H | 6.15-6.06  m, 3H |
| **23** | 5.60  ddd, *^3^J*_H22-H23_=15.4Hz, *^3^J*_H23-H24a_=9.7 Hz, *^3^J*_H23-H24b_=5.9 Hz, 1H | 5.60  ddd, *^3^J*_H23-H22_= 15.3Hz; *^3^J*_H23-H24a_= 9.6 Hz, *^3^J*_H23-H24b_= 5.9Hz, 1H | 5.60  ddd, *^3^J*_H23-H22_=15.4, *^3^J*_H23-H24a_= 9.6 Hz, *^3^J*_H23-H24b_= 5.8Hz, 1H | 5.60  ddd, *^3^J*_H22-H23_= 15.3 Hz, *^3^J*_H23-H24a_ =9.6 Hz, *^3^J*_H23-H24b_ = 5.9 Hz, 1H | 5.62-5.58  m, 1H | 5.60  ddd, *^3^J*_H22-H23_ = 15.3 Hz, *^3^J*_H23-H24a_ = 9.6 Hz, *^3^J*_H23-H24b_ = 5.9 Hz, 2H | 5.60  ddd, *^3^J*_H22-H23_ = 15.3 Hz, *^3^J*_H23-H24a_ = 9.6 Hz, *^3^J*_H23-H24b_ = 5.9 Hz, 2H |
| **24a** | 2.24-2.15  m, 2H | 2.19-2.14  m, 1H | 2.19-2.15  m, 1H | 2.19-2.14  m, 1H | 2.21-2.19  m, 1H | 2.22-2.15  m, 1H, | 2.19-2.15  m, 1H |
| **24b** | 2.38-2.32  m, 1H | 2.42-2.35  m, 2H | 2.43-2.35  m, 2H | 2.41-2.35  m,1H | 2.41-2.38 m, 2H, | 2.40-2.35  m, 1H | 2.42-2.35  m, 2H |
| **25** | 4.68-4.62  m, 1H | 4.70-4.64  m, 1H | 4.70-4.65  m, 1H | 4.74-4.66  m, 1H | 4.74-4.64  m, 1H, | 4.69-4.64  m, 1H | 4.69-4.64  m, 1H |
| **27** | 1.26  d, *^3^J*_H25-H27_ =6.2 Hz, 3H | 1.26  d, *^3^J*_H25-H27_= 6.1 Hz, 3H | 1.26  d, *^3^J*_H25-H27_= 6.2 Hz, 3H | 1.27  d, *^3^J*_H25-H27_ = 6.0 Hz, 3H | 1.26-1.24  m, 3H | 1.25  d, *^3^J*_H25-H27_ = 6.1 Hz, 3H | 1.24  d, *^3^J*_H25-H27_ = 6.1 Hz, 3H |
| **1’** | 4.48  bs, 1H | 4.33  bs, 1H | 4.38  bs, 1H | 4.34  bs,1H | 4.33  bs, 1H | 4.35  bs, 1H | 4.42-4.31  m, 1H |
| **2’** | 3.75  d, *^3^J*_H2’-H3’_ =3.1 Hz, 1H | 3.76  d, *^3^J*_H2’-H3’_= 3.1 Hz, 1H | 3.75  d, *^3^J*_H2’-H3’_= 3.1 Hz, 1H | 3.78  d, *^3^J*_H2’-H3’_ = 3 Hz, 1H, | 3.76  d, *^3^J*_H2’-H3’_ = 3.1 Hz, 1H | 3.81-3.79  m, 2H | 3.79  d, *^3^J*_H2’-H3’_ = 2.9 Hz, 1H |
| **3’** | 2.79-2.74  m, 2H | 2.42-2.35  m, 2H | 2.43 – 2.36  m, 2H | 2.43  dd, *^3^J*_H3’-H4’_ = 9.6 Hz, *^3^J*_H2’-H3’_ = 3.0 Hz, 1H, | 2.41-2.38  m, 2H | 2.41  dd, *^3^J*_H4’-H3’_ = 9.4 Hz, *^3^J*_H2’-H3’_ = 2.6 Hz, 1H | 2.42-2.35  m, 2H |
| **4’** | 3.13-3.09  m, 1H | 3.02-2.98  m, 1H | 3.01-2.97  m, 1H | 3.02-2.97  m, 1H | 3.02-2.99  m, 1H | 3.04-2.98  m, 1H | 3.08-3.02  m, 1H |
| **5’** | 3.23-3.19  m, 2H | 3.12-3.09  m, 1H | 3.15-3.08  m, 1H | 3.12-3.07  m, 1H | 3.13-3.10  m, 1H | 3.14-3.09  m, 1H | 3.14-3.09  m, 1H |
| **6’** | 1.17  d, *^3^J*_H5’-H6’_ =6 Hz, 3H | 1.15  d, *^3^J*_H5’-H6’_= 6.1 Hz 3H | 1.15  d, *^3^J*_H5’-H6’_= 6.1 Hz 3H | 1.15-1.06  m, 3H | 1.19-1.12  m,5H | 1.15  d, *^3^J*_H5’-H6’_ = 6.1 Hz, 3H | 1.16  d, *^3^J*_H5’-H6’_ = 6.1 Hz, 3H |
| **1”a** | - | 3.87  d, *^2^J*_H1”a-H1”b_= 13.9Hz, 1H | 3.85  d, *^2^J*_H1”a-H1”b_= 13.6 Hz, 1H | 3.89  d, *^2^J*_H1’’a-H1’’b_ = 13.9 Hz, 1H | 3.87  d, *^2^J*_H1’’a-H1’’b_= 13.9 Hz, 1H | 3.94 d, *^2^J*_H1’’a-H1’’b_= 14.2 Hz, 1H | 4.02-3.95  m, 1H |
| **1”b** | - | 3.69  d, *^2^J*_H1”a-H1”b_= 13.8 Hz, 1H | 3.65  d, *^2^J*_H1”a-H1”b_= 13.6 Hz, 1H | 3.74  d, *^2^J*_H1’’a-H1’’b_ = 13.9 Hz, 1H | 3.70  d, *^2^J*_H1’’a-H1’’b_= 14 Hz, 1H | 3.81-3.79  m, 2H | 3.85  d, *^2^J*_H1”a-H1”b_ =14.9 Hz, 1H |
| **2”** | - | - | - | - | - |  |  |
| **2”a** | - | - | - | - | - |  |  |
| **2”b** | - | - | - | - | - |  |  |
| **3”** | - | - | 7.51  s, 1H | - | 7.30-7.25  m, 1H | - | - |
| **3”a** | - | 7.40  s, 1H | - | - | - | 7.64-7.61  m, 2H | 8.18-8.14  m, 2H |
| **3”b** | - | 7.40  s, 1H | - | - | - | 7.64-7.61  m, 2H | 8.18-8.14  m, 2H |
| **4”** | - | - | - | 7.56-7.50  m, 1H | - | - | - |
| **4”a** | - | 7.41  s, 1H | - | - | - | 8.06  s | 8.06 (s, H-4”a, H-4”b |
| **4”b** | - | 7.41  s, 1H | - | - | - | 8.06  s | 8.06 (s, H-4”a, H-4”b |
| **5”** | - | - | 7.43  d, *^3^J*_H5”-H6”_=2.4 Hz, 1H | 7.19-7.14  m, 2H | 7.02  td, *^3^J*_H5’’-H6’’_= 8.6 Hz, 1H | - | - |
| **5”a** | - | - | - | - | - | - | - |
| **5”b** | - | - | - | - | - | - | - |
| **6”** | - | - | 7.35-7.29  m, 6H | 7.28  tdd, *^3^J*_H6”-H7”_ = 7.5 Hz, *^3^J*_H6”=H5”_ = 5.3 Hz, 1H, | 7.33-7.30  m, 1H | - | - |
| **6”a** | - | - | - | - | - | - | - |
| **7”** | - | 4.06  s, 1H | 7.35-7.29  m, 6H | 7.19-7.14  m, 2H | 7.21  d, *^3^J*_H6’’-H7’’_= 7.6 Hz, 1H | - | - |
| **9-OH*** | 5.22 | 5.24 | 5.20 | 5.21 | 5.24 | 5.24 | 5.22 |
| **H-NH_3_^+^** | 7.24^×^ | - | - | - | - | - | - |
| **HN^+^-H_a_** | - | 10.5 | 10.01 | 10.24 | 10.02 | 10.2 | 10.50 |
| **HN^+^-H_b_** | - | 9.55 | 9.54 | 9.50 | 9.53 | 8.80 | 9.50 |

×- determined from ^1^H-^15^N HSQC NMR; *- determined from ^1^H-^1^H ROESY NMR

# Table S4 Summary of chemical H atoms shifts [ppm] for PIM derivatives **7-13** from ^1^H NMR spectra.

|  | **7** | **8** | **9** | **10** | **11** | **12** | **13** |
| --- | --- | --- | --- | --- | --- | --- | --- |
| **2** | 6.11-6.08  m, 1H | 6.13-6.10  m, 1H | 6.12-6.06  m, 3H | 6.17-6.12  m, 4H | 6.13-6.10  m, 1H | 6.13-6.10  m, 2H | 6.13-6.10  m, 2H |
| **3** | 6.27  dd, *^3^J*_H2-H3_ = 15.9Hz; *^3^J*_H3-H4_ = 7.7 Hz, 1H | 6.26  dd, *^3^J*_H3-H2_= 15.8 Hz, *^3^J*_H3-H4_=7.7 Hz, 1H | 6.27  dd, *^3^J*_H2-H3_=15.8 Hz, *^3^J*_H3-H4_=7.8 Hz, 1H | 6.27  dd, *^3^J*_H2-H3_= 15.8 Hz, *^3^J*_H3-H4_= 7.7 Hz, 1H | 6.27  dd, *^3^J*_H2-H3_ = 15.8 Hz, *^3^J*_H3-H4_ = 7.7 Hz, 1H | 6.27  dd, *^3^J*_H2-H3_ =15.8 Hz, *^3^J*_H3-H4_ = 7.7 Hz 1H | 6.26  dd, *^3^J*_H3-H2_= 15.8 Hz, *^3^J*_H3-H4_=7.7 Hz, 1H |
| **4** | 3.22  dd, *^3^J*_H3-H4_ = 7.7Hz, *^4^J*_H2-H4_ = 2.0 Hz, 1H | 3.22  dd, *^3^J*_H3-H4_ =7.6 Hz, *^4^J*_H2-H4_ = 2.0 Hz ,1H | 3.22  dd, *^3^J*_H3-H4_ = 7.7 Hz, *^4^J*_H2-H4_ = 2.0 Hz, 2H | 3.22  dd, *^3^J*_H3-H4_= 7.7 Hz, *^4^J*_H2-H4_ = 2.0 Hz 2H | 3.22  dd, *^3^J*_H3-H4_ = 7.7, *^4^J*_H2-H4_ = 2.0 Hz, 2H | 3.22  dd, *^3^J*_H3-H4_ = 7.7 Hz, *^4^J*_H2-H4_ = 2.0 Hz, 1H | 3.22  dd, *^3^J*_H3-H4_ = 7.7 Hz, *^4^J*_H2-H4_ = 2.0 Hz 1H |
| **5** | 2.75-2.72  m, 1H | 2.76-2.73  m, 1H | 2.76-2.73  m, 1H | 2.78-2.74  m, 1H | 2.76-2.73  m, 1H | 2.77-2.73  m, 1H | 2.76-2.73  m, 1H |
| **6a** | 1.17-1.14  m, 5H | 1.15-1.06  m, 2H | 1.15-1.11  m, 2H | 1.19-1.07  m, 5H | 1.20-1.07  m, 5H | 1.22-1.16  m, 2H | 1.15-1.13  m, 2H |
| **6b** | 1.96-1.90  m, 1H | 1.98-1.89  m, 3H | 1.95-1.92  m, 2H | 1.92-1.90  m, 1H) | 1.98-1.88  m,2H | 1.97-1.89  m, 3H | 1.98-1.88  m, 2H |
| **7** | 4.19-4.11  m, 2H | 4.14-4.10  m, 1H | 4.14-4.10  m, 1H | 4.11-4.07  m, 1H | 4.17-4.10  m, 1H | 4.14-4.10  m, 1H | 4.14-4.10  m, 1H |
| **8a** | 1.58  dd, *^2^J*_H8a-8b_= 14.3 Hz, 1H | 1.59-1.50  m, 3H | 1.64-1.56  m, 4H | 1.66-1.55  m, 2H | 1.60-1.58  m, 1H | 1.65-1.53 m, 2H | 1.65-1.58  m, 2H |
| **8b** | 1.53-1.44  m, 2H | 1.59-1.50  m, 3H, | 1.52  dd, *^2^J*_H8a-H8b_= 14.3 Hz, *^3^J*_H8b-H7_ =2.0 Hz,1H | 1.51-1.48  m, 1H | 1.58-1.48  m,2H | 1.51  dd, *^2^J*_H8a-H-8b_= 14.2 Hz, *^3^J*_H8b-H7_ =2.0 Hz, 1H | 1.57-1.48  m, 2H |
| **10a** | 1.88-1.79  m, 3H | 1.84  dd, *^2^J*_H10a-H10b_= 12.3 Hz, *^3^J*_H10a-H11_=4.6 Hz 1H | 1.86  dd, *^2^J*_H10a-H10b_= 12.4 Hz, *^3^J*_H10a-H11_=4.6 Hz, 1H | 1.84-1.80  m, 1H | 1.85  dd, *^2^J*_H10a-H10b_= 12.4 Hz, *^3^J*_H10a-H11_=4.6 Hz 1H | 1.85  dd, *^2^J*_H10a-H10b_= 12.3 Hz, *^3^J*_H10a-H11_=4.7 Hz 1H | 1.85  dd, *^2^J*_H10a-H10b_= 12.3 Hz, *^3^J*_H10a-H11_=4.7 Hz 1H, |
| **10b** | 1.17-1.14  m, 5H | 1.15-1.06  m, 2H | 1.15-1.11  m, 2H | 1.19-1.07  m, 5H | 1.20-1.07  m, 5H | 1.14- 1.10  m, 1H | 1.15-1.13  m, 2H |
| **11** | 4.09-4.07  m, 2H | 4.04-3.99  m,1H | 4.05  td, *^3^J*_H11-H12_ = 10.8 Hz, *^3^J*_H10a-H11_=4.6 Hz ,1H | 4.04-3.99  m, 1H | 4.02  td, *^3^J*_H11-H12_ = 10.8 Hz, *^3^J*_H11-H10a_ = 4.6 Hz, 1H | 4.03  td, *^3^J*_H11-H12_ = 10.8 Hz, *^3^J*_H11-10a_ = 4.7 Hz, 1H | 4.03  td, *^3^J*_H11-H12_ = 10.8 Hz, *^3^J*_H11-H10a_ = 4.7 Hz, 1H |
| **12** | 1.88-1.79  m, 3H | 1.98-1.89  m, 3H | 1.95-1.92  m, 2H | 1.90-1.85  m,1H | 1.98-1.88  m,2H | 1.97-1.89  m, 3H | 1.98-1.88  m, 2H |
| **13** | 4.25-4.20  m, 1H | 4.22-4.20  m, 1H | 4.26-4.21  m, 1H | 4.24-4.20  m, 1H | 4.26-4.19-  m, 1H | 4.23-4.20  m, 1H | 4.23 -4.20  m, 1H |
| **14a** | 2.20-2.28  m, 2H | 2.18-2.08  m, 2H | 2.04-1.99  m, 1H | 2.23-2.14  m, 2H | 2.05-2.00  m, 1H, | 2.07-2.00  m, 1H | 2.05-2.00  m, 1H |
| **14b** | 1.53-1.44  m, 2H | 1.59-1.50  m, 3H | 1.64-1.56  m, 4H | 1.66-1.55  m, 5H | 1.58-1.48  m,2H | 1.65-1.53  m, 2H | 1.57-1.48  m, 2H |
| **15** | 4.40-4.33  m, H | 4.38  dd, *^3^J*_H15-H16_=8.8 Hz, *^3^J*_H15-H14a_= 4.2 Hz, 1H, | 4.38  dd, *^3^J*_H15-H16_= 8.7 Hz, *^3^J*_H15-H14a, H14b_ = 4.7 Hz, 1H | 4.30 –4.27  m,1H | 4.40-4.34  m, 2H | 4.41-4.37  m, 2H | 4.38-4.35  m, 1H |
| **16** | 5.88  dd, *^3^J*_H16-H17_ = 15.3Hz, *^3^J*_H16-H15_ = 9.0 Hz, 1H | 5.89-5.84  m, 1H | 5.88  dd, *^3^J*_H16-H17_ = 15.3 Hz, *^3^J*_H15-H16_= 8.7 Hz, 1H | 5.91-5.88  m, 1H | 5.89  dd, *^3^J*_H16-H17_ = 15.3 Hz, *^3^J*_H16-H15_ = 8.7 Hz, 1H | 5.89  dd, *^3^J*_H16-H17_ = 15.3 Hz, *^3^J*_H16-H15_ = 8.8 Hz, 1H | 5.89  dd, *^3^J*_H16-H17_ = 15.3 Hz, *^3^J*_H16-H15_ = 8.7 Hz, 1H |
| **17** | 6.08- 6.06  m, 2H | 6.09-6.05  m 2H | 6.12-6.06  m, 3H | 6.05- 6.00  m, 2H | 6.09-6.03  m, 2H | 6.13-6.10  m, 2H | 6.13-6.10  m, 2H |
| **18** | 6.54-6.50  m, 2H | 6.50-6.45  m, 1H | 6.49  dd, *^3^J*_H18-H19_=14.6 Hz, *^3^J*_H18-H17_=10.8 Hz, 1H | 6.45  dd, *^3^J*_H18-H19_=14.6; *^3^J*_H18-H17_=10.9 Hz, 1H | 6.49  dd, *^3^J*_H18-H19_ =14.7 Hz, *^3^J*_H18-H17_ = 10.7 Hz, 1H | 6.49  dd, *^3^J*_H18-H19_ =14.5 Hz, *^3^J*_H18-H17_ = 10.8 Hz, 1H | 6.49  dd, *^3^J*_H19-H19_ =14.6 Hz, *^3^J*_H18-H17_ = 10.0 Hz, 1H |
| **19** | 6.21-6.13  m, 3H | 6.19-6.14  m, 3H | 6.22-6.16  m, 3H | 6.17-6.12  m, 4H | 6.21-6.16  m, 3H | 6.24-6.16  m, 3H | 6.24- 6.16  m, 3H |
| **20** | 6.21-6.13  m, 3H | 6.19-6.14  m, 3H | 6.22-6.16  m, 3H | 6.17-6.12  m, 4H | 6.21-6.16  m, 3H | 6.24-6.16  m, 3H | 6.24- 6.16  m, 3H |
| **21** | 6.21-6.13  m, 3H | 6.19-6.14  m, 3H | 6.22-6.16  m, 3H | 6.17-6.12  m, 4H | 6.21-6.16  m, 3H | 6.24-6.16  m, 3H | 6.24- 6.16  m, 3H |
| **22** | 6.08- 6.06  m, 2H | 6.09-6.05  m 2H | 6.12-6.06  m, 3H | 6.05- 6.00  m, 2H | 6.09-6.03  m, 2H | 6.07-6.03  m, 2H | 6.08-6.02  m,2H |
| **23** | 5.60  ddd, *^3^J*_H23-H22_ = 15.4; *^3^J*_H23-H24a_ = 9.9; *^3^J*_H23-H24b_ = 5.8Hz, 1H | 5.63  ddd, *^3^J*_H23-H22_=15.2 Hz, *^3^J*_H23-H24a_=9.6 Hz, *^3^J*_H23-H24b_= 5.9 Hz, 1H | 5.61  ddd, *^3^J*_H23-H22_ = 15.5 Hz, *^3^J*_H23-H24a_ = 10.7 Hz, , *^3^J*_H23-H24b_= 5.9 Hz, 1H | 5.59  ddd, *^3^J*_H22-H23_=15.3 Hz, *^3^J*_H23-H24a_= 9.2 Hz, *^3^J*_H23-H24b_= 6.2 Hz, 1H | 5.60  ddd, *^3^J*_H23-H22_ = 15.2 Hz, *^3^J*_H23-H24a_ =9.7 Hz, *^3^J*_H23-H24b_ =5.9 Hz, 1H | 5.61  ddd, *^3^J*_H23-H22_ = 15.3 Hz, *^3^J*_H23-H24a_=9.6 Hz, *^3^J*_H23-H24b_ =5.9 Hz, 1H | 5.61  ddd, *^3^J*_H23-H22_ = 15.2 Hz, *^3^J*_H23-H24a_ =9.6 Hz, *^3^J*_H23-H24b_ =5.8 Hz, 1H |
| **24a** | 2.20-2.28  m, 2H | 2.18-2.08  m, 2H | 2.19-2.11  m, 1H | 2.23-2.14  m, 2H | 2.25-2.14  m, 1H | 2.20-2.15  m, 1H | 2.20-2.15  m, 1H |
| **24b** | 2.42-2.35  m, 1H | 2.43-2.34  m, 1H | 2.41-2.35  m, 1H | 2.41-2.33  m, 2H | 2.42- 2.38  m, 2H | 2.41-2.35  m, 1H | 2.42-2.34  m, 2H |
| **25** | 4.68-4.63  m, 1H | 4.69-4.63  m, 1H | 4.69-4.62  m, 1H | 4.71-4.66  m, 1H | 4.70-4.65  m, 1H | 4.70-4.65  m, 1H | 4.73-4.65  m, 1H |
| **27** | 1.25-1.20  m, 3H | 1.26  d, *^3^J*_H25-H27_ =6.2 Hz, 3H | 1.26  d, *^3^J*_H25-H27_ = 6.1 Hz, 3H | 1.25-1.19  m, 3H | 1.26  d, *^3^J*_H25-H27_ = 6.2 Hz, 3H | 1.26  d, *^3^J*_H25-H27_ = 6.3 Hz, 3H, | 1.26 d, *^3^J*_H25-H27_ = 6.2 Hz, 3H |
| **1’** | 4.30  bs, 1H | 4.34  bs, 1H | 4.31  bs, 1H | 4.37  bs, 1H | 4.33  bs, 1H | 4.36  bs, 1H | 4.34  bs, 1H |
| **2’** | 4.09-4.07  m, 1H | 3.84  d, *^3^J*_H2’-H3’_ =3.0 Hz 1H | 3.79-3.78  m, 2H | 3.88-3.72  m, 3H | 3.77  d, *^3^J*_H2’-H3’_ = 3.1 Hz, 1H | 3.81  d, *^3^J*_H2’-H3’_ = 3.2 Hz, 1H | 3.78  d, *^3^J*_H2’-H3’_ = 3.2 Hz, 1H |
| **3’** | 2.54* | 2.46  dd, *^3^J*_H3’-H4’_ =9.7 Hz, *^3^J*_H2’-H3’_ = 2.8 Hz 1H | 2.46-2.42  m, 1H | 2.41-2.33  m, 2H | 2.42- 2.38  m, 2H | 2.44  dd, *^3^J*_H3’-H4’_ = 9.7, *^3^J*_H2’-H3’_ = 3.0 Hz, 1H | 2.42-2.34  m, 2H |
| **4’** | 3.14-3.01  m,2H | 3.04-2.99  m, 1H | 3.07-3.00  m, 1H | 2.98-2.95  m, 1H | 3.02-2.97  m, 1H | 3.05-3.00  m,1H | 3.05-3.00  m,1H |
| **5’** | 3.14-3.01  m, 2H | 3.12-3.08  m, 1H | 3.12-3.08  m, 1H | 3.06-3.04  m, 1H | 3.14-3.09  m, 1H | 3.13  dq, *^3^J*_H4’-H5’_ = 9.2 Hz,*^3^J*_H5’-H6’_ = 6 Hz, 1H | 3.15-3.09  m, 1H |
| **6’** | 1.17-1.14  m, 5H | 1.16  d, *^3^J*_H5’-H6’_ =5.9 Hz, 3H | 1.16  d, *^3^J*_H5’-H6’_ = 5.8 Hz, 3H | 1.19-1.07  m, 5H | 1.20-1.07  m, 5H | 1.16  d, *^3^J*_H5’-H6’_=6.0Hz, 3H | 1.15  d, *^3^J*_H5’-H6’_*=* 6.0 Hz, 3H |
| **1’’a** | 4.02  d, *^2^J*_H1’’a-H1’’b_=13.5 Hz, 1H | 3.89  d, *^2^J*_H1’’a-H1’’b_=14.1 Hz, 1H | 3.95  d, *^2^J*_H1’’a-H1’’b_= 14.0 Hz, 1H | 3.88-3.72  m, 3H | 3.85  d, *^2^J*_H1’’a-H1’’b_ = 13.7 Hz, 1H | 3.88  d, *^2^J*_H1’’a-H1’’b_ = 13.4 Hz, 1H | 3.81  d, *^2^J*_H1’’a-H1’’b_ = 13.3 Hz, 1H |
| **1’’b** | 3.81  d, *^2^J*_H1’’a-H1’’b_=13.5 Hz, 1H | 3.76  d, *^2^J*_H1’’a-H1’’b_=14.1 Hz, 1H | 3.79-3.78  m, 2H | 3.88-3.72  m, 3H | 3.66  d, *^2^J*_H1’’a-H1’’b_ = 13.7 Hz, 1H | 3.69  d, *^2^J*_H1’’a-H1’’b_ = 13.4 Hz | 3.69  d, *^2^J*_H1’’a-H1’’b_ = 13.3 Hz, 1H |
| **2’’** | - | - | - | - | - | - | - |
| **2’’a** | - | - | - | - | - | - | - |
| **2’’b** | - | - | - | - | - | - | - |
| **3’’** | - | - | - | 8.06-8.02  m, 1H | - | - | - |
| **3”a** | - | - | 7.53  d, *^3^J* _H4”b-H3”b, H3”a-H4”a_ = 8.0 Hz, 1H | - | 7.34-7.31  m, 3H | 7.44  d, *^3^J*_H3”a.3”b-4”a,b_ = 8.2 Hz, 3H | 7.28  d, *^3^J*_H3”a-4”a, H4”b-H-3”b_ = 7.7 Hz, 2H |
| **3”b** | - | - | 7.53  d, *^3^J* _H4”b-H3”b, H3”a-H4”a_ = 8.0 Hz, 1H | - | 7.34-7.31  m, 3H | 7.44  d, *^3^J*_H3”a.3”b-4”a,b_ = 8.2 Hz 3H | 7.28  d, *^3^J*_H3”a-4”a, H4”b-H-3”b_ = 7.7 Hz, 2H |
| **4”** | 6.54-6.50  m, 2H | 6.72  d, *^3^J*_H4’’-H5’’)_= 8.6 Hz, 1H | - | - | - | - | - |
| **4”a** | - | - | 7.89  dd, *^3^J*_H4”b-H3”b, H3”a-H4”a_ = 8.0 Hz, 5H | - | 7.39-7.33  m, 2H | 7.57-7.53  m, 3H | 7.12  d, *^3^J*_H3”a-4”a, H4”b-H-3”b_ = 7.7 Hz, 2H |
| **4”b** | - | - | 7.89  dd, *^3^J*_H4”b-H3”b, H3”a-H4”a_ = 8.0 Hz, 5H | - | 7.39-7.33  m, 2H | 7.57-7.53  m, 3H | 7.12  d, *^3^J*_H3”a-4”a, H4”b-H-3”b_ = 7.7 Hz, 2H |
| **5”** | 7.88  dd, *^3^J*_H4’’-H5’’_ = 9.2 Hz, 1H | 7.19  dd, *^3^J*_H5’’-H4’’_= 8.56 Hz, *^4^J*_H5’’-H7’’_ = 2.6 Hz 1H, | - | 7.88-7.78  m, 2H | - | - | - |
| **5”a** | - | - | - | - | - | - | - |
| **5”b** | - | - | - | - | - | - | - |
| **6”** |  | - | - | 7.29-7.23  m, 1H | - | - | 2.54  d, *^3^J*_H-6”-H7”_= 7.6 Hz, 2H |
| **6”a** | - | - | - | - | - | - | - |
| **6”b** | - | - | - | - | - | - | - |
| **7”** | 8.10-8.06  m, 1H | 7.31  d, *^4^J*_H7’’-H5’’_= 2.6 Hz, 1H | - | 7.42-7.37  m, 1H | - | - | 1.65-1.58  m, 2H |
| **7”a** | - | - | - | - | - | 7.61-7.57  m, 3H | - |
| **7”b** | - | - | - | - | - | 7.61-7.57  m, 3H | - |
| **8”** | - | - | - | - | 2.54  t, *^3^J*_H8”-H9”_ = 6.9 Hz 2H | - | 0.89  t, *^3^J*_H7”-H8”_= 7.3 Hz, 3H |
| **8”a** | - | - | - | - | ^-^ | 7.01-6.09  m, 3H | - |
| **8”b** | - | - | - | - | ^-^ | 7.01-6.09  m, 3H | - |
| **9”** | - | - | - | - | 3.58  t, *^3^J*_H8”-H9”_ = 6.9 Hz 2H | - | - |
| **10”** | - | - | - | - | - | 3.80  s, 3H, | - |
| **9-OH*** | 5.32 | 5.24 | 5.26 | 5.07 | 5.23 | 5.23 | 5.24 |
| **HN^+^-H_a_** | 10.7 | 10.0 | 10.09 | 10.03 | 10.00 | 10.03 | 9.96 |
| **HN^+^-H_b_** | 10.03 | 8.50 | 9.20 | 9.52 | 9.42 | 9.55 | 9.54 |
| **1_TEA_** | - | - | - | 2.93  q, *^3^J*_H1TEA-H2TEA_ =7.2Hz, 1H, | - | - | - |
| **2_TEA_** | - | - | - | 1.19-1.07  m, 5H | - | - | - |

*- determined from ^1^H-^1^H ROESY NMR

# Table S5 Summary of chemical H atoms shifts [ppm] for PIM derivatives **14-20** from ^1^H NMR spectra.

|  | **14** | **15** | **16** | **17** | **18** | **19** | **20** |
| --- | --- | --- | --- | --- | --- | --- | --- |
| **2** | 6.14-6.10  m, 2H | 6.12-6.07  m, 2H | 6.12-6.11  m, 1H | 6.12-6.10  m, 1H | 6.18-6.12  m, 3H | 6.12-6.10  m, 1H | 6.12-6.10  m, 1H |
| **3** | 6.27  dd, *^3^J*_H3-H2_= 15.8 Hz, *^3^J*_H3-H4_=7.7 Hz, 1H | 6.27  dd, *^3^J*_H2-H3_ = 15.8Hz, *^3^J*_H3-H4_ = 7.8 Hz, 1H | 6.27  dd, *^3^J*_H2-H3_= 15.8 Hz, *^3^J*_H3-H4_ = 7.7 Hz, 1H | 6.27  dd, *^3^J*_H2-H3_ = 15.8 Hz, *^3^J*_H3-H4_ = 7.7 Hz, 1H | 6.28  dd, *^3^J*_H2-H3_ = 15.8Hz, *^3^J*_H3-H4_ = 7.8 Hz, 1H | 6.26  dd, *^3^J*= 15.8 Hz, *^3^J*_H3-H4_ = 7.7 Hz, 1H | 6.27  dd, *^3^J*_H3-H2_= 15.8 Hz, *^3^J*_H3-H4_=7.7 Hz, 1H, |
| **4** | 3.22  dd, *^3^J*_H3-H4_ = 7.7 Hz, *^4^J*_H2-H4_ = 2.0 Hz, 2H | 3.22  dd, *^3^J*_H3-H4_ = 7.7 Hz, *^4^J*_H2-H4_ = 2.0 Hz 1H | 3.23  dd, *^3^J*_H3-H4_ = 7.6 Hz, *^4^J*_H2-H4_ = 2.0 Hz 1H | 3.22  dd, *^3^J*_H3-H4_ = 7.7 Hz, *^4^J*_H2-H4_ = 2.0 Hz 1H | 3.23-3.20  m, 2H | 3.22  dd, *^3^J*_H3-H4_ = 7.7 Hz, *^4^J*_H2-H4_ = 2.0 Hz 1H | 3.22  dd, *^3^J*_H3-H4_ = 7.7 Hz, *^4^J*_H2-H4_ = 2.0 Hz 1H |
| **5** | 2.76-2.73  m, 1H | 2.77-2.73  m, 1H | 2.78-2.73  m, 1H) | 2.77-2.73  m, 1H | 2.78-2.73  m, 1H | 2.76-2.73  m, 1H | 2.76-2.73  m, 1H |
| **6a** | 1.14-1.10  m, 3H | 1.22- 1.16  m, 1H | 1.21-1.10  m, 5H | 1.21-1.18  m, 1H | 1.16 – 1.05  m, 2H | 1.13-1.07  m, 2H | 1.22-1.17  m, 1H |
| **6b** | 1.97-1.91  m, 3H | 1.98- 1.89  m, 2H | 2.00-1.92  m, 2H | 1.98-1.89  m, 2H | 2.01-1.94  m, 2H | 1.98-1.92  m, 2H | 2.00-1.91  m, 2H |
| **7** | 4.17-4.10  m, 1H | 4.14-4.09  m, 1H | 4.17-4.10  m, 1H | 4.14-4.09  m, 1H | 4.12- 4.06  m, 3H | 4.17-4.10  m, 1H | 4.14-4.09  m, 1H |
| **8a** | 1.60-1.55  m, 2H | 1.72-1.65  m, 3H | 1.64-1.53  m, 3H | 1.64-1.54  m, 2H | 1.67-1.59  m, 3H | 1.59  dd, *^2^J*_H8a-H8b_= 14.1 Hz, *^3^J*_H8a-H7_=10.6 Hz, 1H | 1.64-1.50  m, 3H |
| **8b** | 1.55-1.50  m, 1H | 1.72-1.65  m, 3H | 1.64-1.53  m, 3H | 1.51  dd, *^2^J*_H8a-H8b_ = 14.2 Hz, *^3^J*_H_ =2.0 Hz , 1H | 1.67-1.59  m, 3H | 1.56-1.47  m, 2H | 1.64-1.50  m, 3H |
| **10a** | 1.85  dd, *^2^J*_H10a-H10b_= 12.3 Hz, *^3^J*_H10a-H11_=4.7 Hz, 1H | 1.85  dd, *^2^J*_H10a-H10b_= 12.4 Hz, *^3^J*_H10a-H11_=4.7 Hz 1H | 1.89-1.83  m, 1H | 1.86  dd, *^2^J*_H10a-H10b_ = 12.6 Hz, *^3^J*_H10a-H11_=4.7 Hz , 1H | 1.88  dd, *^2^J*_H10a-H10b_= 12.3 Hz, *^3^J*_H10a-H11_=4.6 Hz , 1H | 1.83  dd, *^2^J*_H10a-H10b_= 12.3 Hz, *^3^J*_H10a-H11_=4.7 Hz ,1H | 1.85  dd, *^2^J*_H10a-H10b_= 12.3 Hz, *^3^J*_H10a-H11_=4.6 Hz 1H |
| **10b** | 1.14-1.10  m, 3H | 1.14-1.09  m, 2H | 1.21-1.10  m, 5H | 1.15-1.09  m, 1H | 1.16 – 1.05  m, 2H | 1.13-1.07  m, 2H | 1.15-1.04  m, 5H |
| **11** | 4.05-4.03  m, 3H | 4.04  td, *^3^J*_H11-H12_ = 10.8Hz , *^3^J*_H11-H10a_ = 4.7 Hz 1H | 4.10 – 3.99  m, 2H | 4.07-4.03  m, 2H | 4.12- 4.06  m, 3H | 4.00  td, *^3^J*_H11-H12_= 10.8 Hz, *^3^J*_H11-H10a_= 4.7 Hz, 1H | 4.02  td, *^3^J*_H11-H12_ = 10.8 Hz, *^3^J*_H11-H10a_= 4.7 Hz, 1H |
| **12** | 1.97-1.91  m, 3H | 1.98- 1.89  m, 2H | 2.00-1.92  m, 2H | 1.98-1.89  m, 2H | 2.01-1.94  m, 2H | 1.98-1.92  m, 2H | 2.00-1.91  m, 2H |
| **13** | 4.22-4.19  m, 1H | 4.23-4.20  m 1H | 4.24-4.20  m, 1H | 4.24-4.20  m, 1H | 4.32-4.28  m 1H, | 4.20-4.18  m, 1H | 4.21-4.18  m, 1H |
| **14a** | 2.03-1.99  m, 1H | 2.04-2.00  m, 1H | 2.05-1.99  m, 1H | 2.05-2.00  m, 1H | 2.12-2.08  m, 1H | 2.09-2.04  m, 1H | 2.04-2.00  m, 1H |
| **14b** | 1.60-1.55  m, 2H | 1.72-1.65  m, 3H | 1.64-1.53  m, 3H | 1.64-1.54  m, 2H | 1.67-1.59  m, 3H | 1.56-1.47  m, 2H | 1.64-1.50  m, 3H |
| **15** | 4.38  ddd, *^3^J*_H15-H16_ =8.3 Hz, *^3^J*_H15-H14a_ = 4.6 Hz, *^4^J*_H15-H17_ =2.7 Hz, 1H | 4.40-4.38  m, 2H | 4.38-4.33  m, 1H | 4.41 – 4.36  m, 2H | 4.46-4.45  m, 2H | 4.41-4.35  m, 2H | 4.38-4.34  m, 1H |
| **16** | 5.89  dd, *^3^J*_H16-H17_ = 15.3 Hz, *^3^J*_H16-H15_ = 8.8 Hz, 1H | 5.93  dd, *^3^J*_H16-H17_ = 15.3 Hz, *^3^J*_H16-H15_ = 8.7 Hz, 1H | 5.88-5.80  m, 1H | 5.89  dd, *^3^J*_H16-H17_ = 15.3 Hz, *^3^J*_H_ = Hz, 1H | 5.93  dd, *^3^J*_H16-H17_ = 15.3 Hz, *^3^J*_H16-H15_ = 8.7 Hz, 1H | 5.88  dd, *^3^J*_H16-H17_ = 15.3 Hz, *^3^J*_H16-H15_ = 8.8 Hz, 1H | 5.88  dd, *^3^J*_H16-H17_ = 15.3 Hz, *^3^J*_H16-H15_ = 8.7 Hz, 1H |
| **17** | 6.14-6.10  m, 2H | 6.12-6.07  m, 2H | 6.11-6.02  m, 2H | 6.11 – 6.04  m, 2H | 6.18-6.12  m, 2H | 6.09-6.04  m, 2H | 6.10-6.02  m, 2H |
| **18** | 6.49  dd, *^3^J*_H18-H19_ =14.6 Hz, *^3^J*_H18-H17_ = 10.8 Hz, 1H | 6.50  dd, *^3^J*_H18-H19_ =14.5 Hz, *^3^J*_H18-H17_ = 10.8 Hz, 1H | 6.49  dd, *^3^J*_H18-H19_ = 14.7 Hz, *^3^J*_H17-H18_ = 10.7 Hz, 1H | 6.50  dd, *^3^J*_H18-H19_ = 14.7 Hz, *^3^J*_H17-H18_ = 10.8 Hz, 1H | 6.53  dd, *^3^J*_H18-H19_ =14.6 Hz, *^3^J*_H18-H17_ = 10.8 Hz, 1H | 6.49  dd, *^3^J*_H18-H19_ =14.6 Hz, *^3^J*_H18-H17_ = 10.8 Hz, 1H | 6.49  dd, *^3^J*_H18-H19_ =14.6 Hz, *^3^J*_H18-H17_ = 10.7 Hz, 1H |
| **19** | 6.22-6.14  m, 3H | 6.24-6.14  m, 3H | 6.23-6.12  m, 3H | 6.21-6.16  m, 3H | 6.26-6.20  m, 2H, | 6.21-6.16  m, 3H | 6.21-6.16  m, 3H |
| **20** | 6.22-6.14  m, 3H | 6.24-6.14  m, 3H | 6.23-6.12  m, 3H | 6.21-6.16  m, 3H | 6.18-6.12  m, 2H | 6.21-6.16  m, 3H | 6.21-6.16  m, 3H |
| **21** | 6.22-6.14  m, 3H | 6.24-6.14  m, 3H | 6.23-6.12  m, 3H | 6.21-6.16  m, 3H | 6.26-6.20  m, 2H | 6.21-6.16  m, 3H | 6.21-6.16  m, 3H |
| **22** | 6.08 – 6.03  m, 1H | 6.06  d, *^3^J*_H_ = 5.1 Hz, 1H | 6.11-6.02  m, 2H | 6.11 – 6.04  m, 2H | 6.08 d, *^3^J*_H22-H23_ = 15.0 Hz, 2H | 6.09-6.04  m, 2H | 6.10-6.02  m, 2H |
| **23** | 5.61  ddd, *^3^J*_H23-H22_ = 15.4 Hz, *^3^J*_H23-H24a_ =9.7 Hz, *^3^J*_H23-H24b, H23-H21_ =5.9 Hz, 1H | 5.60  ddd, *^3^J*_H23-H22_ = 15.4 Hz, *^3^J*_H23-H24a_ =9.7 Hz, *^3^J*_H23-H24b_ =5.9 Hz, 1H | 5.64 – 5.57  m, 1H | 5.60  ddd, *^3^J*_H23-H22_ = 15.3 Hz, *^3^J*_H23-H24a_ = 9.7 Hz, 1H | 5.61  ddd, *^3^J*_H23-H22_ = 15.4 Hz, *^3^J*_H23-H24a_ =9.7 Hz, *^3^J*_H23-H24b_ =5.9 Hz, 1H | 5.60  ddd, *^3^J*_H23-H22_ = 15.2 Hz, *^3^J*_H23-H24a_ =9.6 Hz, *^3^J*_H23-H24b_ =5.8 Hz, 1H | 5.60  ddd, *^3^J*_H23-H22_ = 15.3 Hz, *^3^J*_H23-H24a_ =9.6 Hz, *^3^J*_H23-H24b_ =5.9 Hz, 1H |
| **24a** | 2.24-2.18  m, 1H | 2.19-2.14  m, 1H | 2.24-2.15  m, 1H | 2.19-2.13  m, 1H | 2.20-2.15  m, 1H | 2.19-2.13  m, 1H | 2.19-2.13  m, 1H |
| **24b** | 2.42-3.35  m, 1H | 2.41-2.35  m, 1H | 2.42-2.36  m, 1H | 2.41-2.35  m, 1H | 2.41-2.36  m, 1H | 2.41-2.35  m, 1H | 2.42-2.34  m, 2H |
| **25** | 4.70-4.64  m, 1H | 4.69-4.62  m, 1H | 4.70-4.65  m, 1H | 4.69-4.62  m, 1H | 4.70-4.64  m, 1H | 4.69-4.64  m, 1H | 4.73-4.65  m, 1H |
| **27** | 1.26  d, *^3^J*_H25-H27_ = 6.1 Hz, 3H | 1.30-1.23*  m, 24H | 1.26  d, *^3^J*_H25-H27_ = 6.2 Hz, 3H | 1.25  d, *^3^J*_H25-H27_ = 6.9 Hz, 3H | 1.26  d, *^3^J*_H25-H27_ =6.2 Hz, 3H | 1.26  d, *^3^J*_H25-H27_ = 6.1 Hz, 3H | 1.26  d, *^3^J*_H25-H27_ = 6.1 Hz,  3H |
| **1’** | 4.34  bs, 1H | 4.32  bs, 1H | 4.32-4.30  m, 1H | 4.41 – 4.36  m, 2H | 4.46-4.45  m, 2H | 4.41-4.35  m, 2H | 4.33  bs, 1H |
| **2’** | 3.80  d, *^3^J*_H2’-H3’_ = 3.1 Hz, 1H | 3.83-3.77  m, 2H | 3.77-3.69  m, 1H | 3.88-3.81  m, 2H | 4.20-4.14  m,1H | 3.72  d, *^3^J*_H2’-H3’_ = 3.2 Hz, 1H | 3.68  d, *^3^J*_H2’-H3’_ = 3.1 Hz, 1H |
| **3’** | 2.46  dd, *^3^J*_H3’-H4’_ = 9.6, *^3^J*_H3’-H2’_ = 3.0 Hz, 1H | 2.46  dd, *^3^J*_H3’-H4’_ = 9.8 Hz, *^3^J*_H2’-H3’_ = 3.0 Hz, 1H | 2.68-2.64  m, 1H | 2.50^  1H | 2.82  dd, *^3^J*_H3’-H4’_ = 9.8 Hz, *^3^J*_H2—H3’_ = 3.0 Hz, 1H | 2.56- 2.52  m, 1H | 2.42-2.34  m, 2H |
| **4’** | 3.02-2.98  m, 1H | 3.04-2.99  m, 1H | 3.12-3.05  m, 1H | 3.07-3.01  m, 1H | 3.07-3.01  m, 1H | 3.05-2.99  m, 1H | 2.98-2.92  m, 1H |
| **5’** | 3.13-3.04  m, 1H | 3.12  dq, *^3^J*_H4’-H5’_ = 8.9 Hz, *^3^J*_H5’-H6’_ = 6.1 Hz, 1H | 3.18-3.11  m, 1H | 3.13  dq, *^3^J*_H4’-H5’_ = 9.0 Hz, *^3^J*_H5’-H6’_ = 6.0 Hz, 1H | 3.23-3.20  m, 2H | 3.16  dq, *^3^J*_H4’-H5’_ =9.1 Hz, *^3^J*_H5’-H6’_ = 6.1 Hz, 1H | 3.17-3.10  m, 1H |
| **6’** | 1.15  d, *^3^J*_H5’-H6’_ =6.1 Hz, 3H | 1.15  d, *^3^J*_H5’-H6’_ = 6.0 Hz, 3H | 1.21-1.10  m, 5H | 1.16  d, *^3^J*_H5’-H6’_ = 6.0 Hz, 3H | 1.18  d, *^3^J*_H5’-H6’_ = 6.1 Hz, 3H | 1.16  d, *^3^J*_H5’-H6’_ = 6.0 Hz, 3H | 1.15-1.04  m, 5H |
| **1”a** | 3.85  d, *^2^J*_H1’’a-H1’’b_ = 13.3 Hz, 1H | 3.83-3.77  m, 2H | 4.10 – 3.99  m, 2H | 4.07-4.03  m, 2H | 4.81  d, *^2^J*_H1”a-H1”b_ = 11.9 Hz, 1H | 2.50^ | 2.31  dd, *^2^J*_H1”a=1”b_ = 11.5 Hz, *^3^J*_H1”a-2”_ =6.4 Hz, 1H |
| **1”b** | 3.69  d, *^2^J*_H1’’a-H1’’b_ = 13.3 Hz, 1H | 3.63  d, *^2^J*_H1”a-H1”b_ = 12.9 Hz, 1H | 3.93 – 3.85  m, 1H | 3.88-3.81  m, 2H | 4.58  d, *^2^J*_H1”a-H1”b_ = 11.9 Hz, 1H | 2.50^ | 2.59  dd, *^2^J*_H1”a=1”b_ = 11.5 Hz, *^3^J*_H1”b-2”_ =6.4 Hz, 1H |
| **2”** | - | - | - |  | - | 1.03-0.92  m, 1H | 1.49  d, *^3^J*_H1”a-2”_= 6.0 Hz, 1H |
| **2”a** | - | - | 4.43 – 4.34  m, 1H |  | - | - | - |
| **2”b** | - | - | 4.43 – 4.34  m, 1H |  | - | - | - |
| **3”** | - | 6.87-6.83  m, 3H | - | 7.92-7.83  m, 6H | - | 0.48-0.40  m, 2H | 1.15-1.04  m, 5H |
| **3”a** | - | - | 4.52 – 4.48  m, 1H | - | - | - | - |
| **3”b** | - | - | 4.52 – 4.48  m, 1H | - | - | - | - |
| **4”** | 6.97  dd, *^3^J*_H4’’-H5’’_=7.8Hz, *^4^J*_H4’’-H6’’_=1.1 Hz, 1H | - | - | - | - | 0.20-0.13  m, 2H | 0.88-0.86  m, 6H |
| **4”a** | - | - | - | - | 8.47  d, *^3^J*_H4”a,H4”b-H5”a,H5”b_ = 8.9 Hz, 3H | - | - |
| **4”b** | - | - | - | - | 8.47  d, *^3^J*_H4”a,H4”b-H5”a,H5”b_ = 8.9 Hz, 3H | - | - |
| **5”** | 7.21  td, *^3^J*_H5’’-H4’’_=7.8Hz, *^4^J*_H5’’-H7’’_= 1.1 Hz, 1H | 7.32-7.27  m, 3H | - | 7.92-7.83  m, 6H | - | - | 0.88-0.86  m, 6H |
| **5”a** | - | - | 7.38-7.24  m, 2H | - | 7.54-7.49  m, 3H | - | - |
| **5”b** | - | - | 7.38-7.24  m, 2H | - | 7.54-7.49  m, 3H | - | - |
| **6”** | 6.90  td, *^3^J*_H6’’-H7’’_=7.5Hz, *^4^J*_H6’’-H4’’_= 1.1 Hz, 1H | 6.87-6.83  m, 3H | - | 7.50-7.44  m, 3H | - | - | - |
| **6”a** | - | - | 7.93-7.85  m, 2H | - | 7.54-7.49  m, 3H | - | - |
| **6”b** | - | - | 7.93-7.85  m, 2H | - | 7.54-7.49  m, 3H | - | - |
| **7”** | 7.36  dd, *^3^J*_H7’’-H6’’_=7.5 Hz, *^4^J*_H5’’-H7’’_= 1.8 Hz, 1H | 7.32-7.27  m, 3H | 7.64-7.51  m, 1H | 7.50-7.44  m, 3H | - | - | - |
| **7”a** | - | - | - | - | 8.08  dt, *^3^J*_H7”a,H7”b-H6”a,H6”b_ =8.9 Hz, 4H | - | - |
| **7”b** | - | - | - | - | 8.08  dt, *^3^J*_H7”a,H7”b-H6”a,H6”b_ =8.9 Hz, 4H | - | - |
| **8”** | 4.05-4.03  m, 3H | 3.93  t, *^3^J* = 6.5 Hz, 3H | - | 7.92-7.83  m, 6H | - | - | - |
| **8”a** | - | - | - | - | - | - | - |
| **8”b** | - | - | - | - | - | - | - |
| **9”** | 3.75-3.70  m, 1H | 1.72-1.65  m, 3H | - | - | 8.54  s, 1H | - | - |
| **9”a** | - | - | - | - | - | - | - |
| **9”b** | - | - | - | - | - | - | - |
| **10”** | - | 1.45-1.34  m, 4H | - | 7.92-7.83  m, 6H | - | - | - |
| **11”** |  | 1.30-1.23*  m, 24H | - | 7.57  dd, *^3^J*_H_ = 8.4 Hz, *^3^J*_H_ = 1.8 Hz, 2H | - | - | - |
| **12”** |  | 1.30-1.23*  m, 24H | - | - | - | - | - |
| **13”** |  | 1.30-1.23*  m, 24H | - | - | - | - | - |
| **14”** |  | 1.30-1.23*  m, 24H | - | - | - | - | - |
| **15”** |  | 1.30-1.23*  m, 24H | - | - | - | - | - |
| **15”** |  | 1.30-1.23*  m, 24H | - | - | - | - | - |
| **16”** |  | 1.30-1.23*  m, 24H | - | - | - | - | - |
| **17”** |  | 0.86  t, *^3^J*_H17”-H16”_= 6.9 Hz, 4H | - | - | - | - | - |
| **9-OH^^** | 5.24 | 5.26 | 5.27 | 5.26 | 5.31 | 5.24 | 5.26 |
| **HN^+^-H_a_** | 10.46 | 9.86 | 10.02 | 10.16 | 10.13 | 9.60 | 9.60 |
| **HN^+^-H_b_** | 9.54 | 9.20 | 9.50 | 9.51 | 9.50 | 8.80 | 8.30 |

*-overlapped, ^-overlapped in DMSO-*d_6_*, ^^- determined from ^1^H-^1^H ROESY NMR

# Table S6 Summary of chemical H atoms shifts [ppm] for PIM derivatives **21-25** from ^1^H NMR spectra.

|  | **21** | **22** | **23** | **24** | **25** |
| --- | --- | --- | --- | --- | --- |
| **2** | 6.13-6.10  m, 1H | 6.13-6.10  m, 1H | 6.13-6.10  m, 1H | 6.10-6.08  m, 2H | 6.12-6.10  m, 1H |
| **3** | 6.27  dd, *^3^J*_H2-H3_ = 15.8Hz, *^3^J*_H3-H4_ = 7.7 Hz, 1H | 6.27  dd, *^3^J*_H2-H3_ = 15.8Hz, *^3^J*_H3-H4_ = 7.7 Hz, 1H | 6.27  dd, *^3^J*_H2-H3_ = 15.8Hz, *^3^J*_H3-H4_ = 7.7 Hz, 1H | 6.27  dd, *^3^J*_H3-H2_= 15.8 Hz, *^3^J*_H3-H4_=7.7 Hz | 6.28  dd, *^3^J*_H2-H3_=15.8 Hz, *^3^J*_H3-H4_= 7.6 Hz, 1H |
| **4** | 3.22  dd, *^3^J*_H3-H4_ = 7.7 Hz, *^4^J*_H2-H4_ = 2.0 Hz,2H | 3.22  dd, *^3^J*_H3-H4_ = 7.6 Hz, *^4^J*_H2-H4_ = 1.9 Hz, 1H | 3.22  dd, *^3^J*_H3-H4_ = 7.7 Hz, *^4^J*_H2-H4_ = 2.0 Hz, 1H | 3.22-3.21  m, 1H | 3.24-3.21  m, 1H |
| **5** | 2.80-2.74  m, 2H | 2.80-2.73  m, 2H | 2.78-2.73  m, 3H | 2.76-2.72  m, 1H | 2.76-2.72  m, 1H |
| **6a** | 1.15-1.08  m, 2H | 1.20 – 1.09  m, 5H | 1.16-1.07  m, 9H | 1.19-1.16  m, 1H | 1.17-1.13  m, 10H |
| **6b** | 1.94-1.90  m,1H | 1.94-1.90  m,1H | 1.98-1.91  m, 2H | 1.95-1.90  m, 2H | 2.00-1.93  m, 3H |
| **7** | 4.14-4.09  m, 1H | 4.14-4.09  m, 1H | 4.14-4.09  m, 1H | 4.12-4.09  m, 1H | 4.24-4.03  m, 4H |
| **8a** | 1.62-1.54  m, 1H | 1.62 – 1.57  m, 1H | 1.63-1.48  m, 5H | 1.63-1.52  m, 2H | 1.69-1.58  m, 2H |
| **8b** | 1.56-1.49  m, 2H | 1.58 – 1.51  m, 2H | 1.63-1.48  m, 5H | 1.50  dd, *^2^J*_H8a-H8b_= 14.2 Hz, 1H, *^3^J*_H8b-H7_= 2.0 Hz, 1H | 1.51-1.47  m, 1H |
| **10a** | 1.84-1.80  m, 1H | 1.84  dd, *^2^J*_H10a-H10b_= 12.3 Hz, *^3^J*_H11-10a_= 4.6 Hz, 1H | 1.84  dd, *^2^J*_H10a-H10b_= 12.3 Hz, *^3^J*_H10a-H11_=4.6 Hz 1H, | 1.84  dd, *^2^J*_H10a-H10b_= 12.3 Hz, *^3^J*_H10a-H11_= 4.6 Hz, 1H | 1.87  dd, *^2^J*_H10a-H10b_= 12.4 Hz, *^3^J*_H10a-H11_= 4.7 Hz, 1H |
| **10b** | 1.15-1.08  m, 2H | 1.20–1.09  m, 5H | 1.16-1.07 m, 9H | 1.14-1.07  m, 3H | 1.17-1.13  m, 10H, |
| **11** | 4.00  td, *^3^J*_H11-H12_ = 10.7 Hz, *^3^J*_H11-10a_= 4.7 Hz, 1H | 4.00  td, *^3^J*_H11-H12_ = 10.7 Hz, *^3^J*_H11-10a_= 4.7 Hz, 1H | 4.01  td, *^3^J*_H11-H12_ = 10.8 Hz, *^3^J*_H_ _11-H10a_= 4.7 Hz, 1H | 4.00-3.97  m, 1H | 4.24-4.03  m, 4H |
| **12** | 1.89-1.85  m, 1H | 1.88-1.85  m, 1H | 1.98-1.91  m,2H | 1.95-1.90  m, 2H | 2.00-1.93  m, 3H |
| **13** | 4.20-4.18  m, 1H | 4.20-4.17  m, 1H | 4.20-4.17  m, 1H | 4.20-4.17  m, 1H | 4.32-4.28  m, 1H |
| **14a** | 2.11-2.05  m, 1H | 2.11-2.04  m, 1H | 2.08-2.00  m, 1H | 2.06-2.02  m, 1H | 2.00-1.93  m, 3H |
| **14b** | 1.56-1.49  m, 2H | 1.58 – 1.51  m, 2H | 1.63-1.48  m, 5H | 1.63-1.52  m, 2H | 1.44-1.41  mM, 1H |
| **15** | 4.40-4.36  m, 1H | 4.44-4.35  m, 2H | 4.42-4.39  m,1H | 4.40-4.34  m, 2H | 4.36-4.31  m, 1H |
| **16** | 5.89  dd, *^3^J*_H16-H17_ = 15.3 Hz, *^3^J*_H16-H15_ = 8.7 Hz, 1H | 5.89  dd, *^3^J*_H16-H17_ = 15.3 Hz, *^3^J*_H16-H15_ = 8.8 Hz, 1H | 5.88  dd, *^3^J*_H16-H17_ = 15.3 Hz, *^3^J*_H16-H15_ = 8.8 Hz, 1H | 5.92-5.85  m, 1H | 5.89  dd, *^3^J*_H16-H17_= 15.3 Hz, *^3^J*_H16-H15_= 8.9 Hz, 1H |
| **17** | 6.10-6.07  m, 2H | 6.10-6.07  m, 2H | 6.10 – 6.03  m, 2H | 6.10-6.08  m, 2H | 6.09 – 6.03  m, 2H |
| **18** | 6.50  dd, *^3^J*_H18-H19_ =14.5 Hz, *^3^J*_H18-H17_ = 10.8 Hz, 1H | 6.50  dd, *^3^J*_H18-H19_ =14.5 Hz, *^3^J*_H18-H17_ = 10.8 Hz, 1H | 6.49  dd, *^3^J*_H18-H19_ =14.6 Hz, *^3^J*_H18-H17_ = 10.8 Hz, 1H | 6.49  dd, ^3^*J* _H19-H18_= 14.8, ^3^*J*_H18-H17_ =10.0 Hz, 1H | 6.50  dd, *^3^J*_H18-H19_= 14.7 Hz, *^3^J*_H18-H17_= 10.9 Hz, 1H |
| **19** | 6.21-6.16  m, 3H | 6.24-6.16  m, 3H | 6.21-6.16  m, 3H | 6.25-6.16  m, 3H | 6.24 – 6.17  m, 3H |
| **20** | 6.21-6.16  m, 3H | 6.24-6.16  m, 3H | 6.21-6.16 | 6.25-6.16  m, 3H | 6.24 – 6.17  m, 3H |
| **21** | 6.21-6.16  m, 3H | 6.24-6.16  m, 3H | m, 3H | 6.25-6.16  m, 3H | 6.24 – 6.17  m, 3H |
| **22** | 6.10-6.07  m, 2H | 6.10-6.07  m, 2H | 6.21-6.16  m, 3H | 6.08-6.02  m, 1H | 6.09 – 6.03  m, 2H |
| **23** | 5.60  ddd, *^3^J*_H23-H22_ = 15.3 Hz, *^3^J*_H23-H24a_ =9.7 Hz, *^3^J*_H23-H24b_ =5.9 Hz, 1H | 5.60  ddd, *^3^J*_H23-H22_ = 15.3 Hz, *^3^J*_H23-H24a_ =9.7 Hz, *^3^J*_H23-H24b_ =6.0 Hz, 1H | 5.61  ddd, *^3^J*_H22-H23_ = 15.3 Hz, *^3^J*_H23-H24a_ =9.7 Hz, *^3^J*_H23-H24b_ =5.9 Hz, 1H | 5.60  ddd, *^3^J*_H23-H22_= 15.3Hz; *^3^J*_H23-H24a_= 9.7Hz, *^3^J*_H23-H24b_= 5.9Hz, 1H | 5.60  ddd, *^3^J*_H22-H23_= 15.4 Hz, *^3^J*_H23-H24a_= 9.8 Hz, *^3^J*_H23-H24b_= 5.9 Hz, 1H |
| **24a** | 2.19-2.12  m, 1H | 2.19-2.13  m, 1H | 2.19-2.13  m, 1H | 2.19-2.13  m, 1H | 2.19-2.14  m, 1H |
| **24b** | 2.38-2.33  m, 1H | 2.38-2.34  m, 1H | 2.41-2.35  m, 1H | 2.41-2.34  m, 1H | 2.42 – 2.35  m, 2H |
| **25** | 4.69-4.62  m, 1H | 4.69-4.62  m, 1H | 4.68-4.61  m, 1H | 4.72-4.67  m, 1H | 4.69-4.62  m, 1H |
| **27** | 1.26- 1.20*  m, 19H | 1.30.-1.20*  m, 23H | 1.29-1.24*  m, 26H | 1.28-1.22  m, 3H | 1.31 – 1.23*  m, 28H |
| **1’** | 4.41  bs, 1H | 4.44-4.35  m, 2H | 4.38  bs, 1H | 4.32-4.29  m, 1H | 4.24-4.03  m, 4H |
| **2’** | 3.78  d, *^3^J*_H2’-H3’_ = 3.1 Hz, 1H | 3.78  d, *^3^J*_H3’-H2’_ = 3.1 Hz, 1H | 3.75  d, *^3^J*_H2’-H3’_ = 3.1 Hz, 1H | 3.81  d, *^3^J*_H2’-H3’_= 2.5 Hz, 1H | 3.95 – 3.91  m, 2H |
| **3’** | 2.67-2.59  m, 2H | 2.66-2.60  m, 2H | 2.65-2.56  m, 3H | 2.51-2.45  m, 1H | 2.42 – 2.35  m, 2H |
| **4’** | 3.12-3.07  m, 1H | 3.12-3.07  m, 1H | 3.10-3.05  m, 1H | 3.40-3.38  m, 1H | 3.16 – 2.97  m, 1H |
| **5’** | 3.21-3.17  m, 1H | 3.22-3.16  m, 1H | 3.21-3.16  m,1H | 3.16-3.09  m, 1H | 2.96 – 2.91  m, 1H |
| **6’** | 1.16  d, *^3^J­*_H5’-H6’_*=*6.1 Hz, 3H | 1.20 – 1.09  m, 5H | 1.16-1.10  m, 3H | 1.15-1.12  m, 3H | 1.12  d, *^3^J*_H5’-H6’_= 6.1 Hz, 3H |
| **1”a** | 2.80-2.74  m, 2H | 2.80-2.73  m, 2H | 2.78-2.73  m, 3H | 2.85-2.80  m, 2H | 3.95 – 3.91  m, 2H |
| **1”b** | 2.67-2.59  m, 2H | 2.66-2.60  m, 2H | 2.65-2.56  m, 3H | 2.71-2.65  m, 1H | 3.69  d, *^2^J*_H1”a-H1”b_=11.3 Hz, 1H |
| **2”** | 1.26- 1.20*  m, 19H | 1.30.-1.20*  m, 23H | 1.63-1.48  m, 5H | 0.99  q, *^3^J* _H1”-H2”, H1”’-H2”_= 6.7 Hz, 7H | - |
| **2”a** | - | - | - | - |  |
| **2”b** | - | - | - | - |  |
| **3”** | 1.26- 1.20*  m, 19H | 1.30.-1.20 *  m, 23H | 1.29-1.24*  m, 26H | - | 6.82  d, *J* = 8.5 Hz, 2H |
| **3”a** | - | - | - | - | - |
| **3”b** | - | - | - | - | - |
| **4”** | 1.26- 1.20*  m, 19H | 1.30.-1.20 *  m, 23H | 1.29-1.24* m, 26H | - | - |
| **4”a** | - | - | - | - | - |
| **4”b** | - | - | - | - | - |
| **5”** | 1.26- 1.20*  m, 19H | 1.30.-1.20 *  m, 23H | 1.29-1.24* m, 26H | - | 7.38-7.30  m, 3H |
| **5”a** | - | - | - | - | - |
| **5”b** | - | - | - | - | - |
| **6”** | 1.26- 1.20*  m, 19H | 1.30.-1.20 *  m, 23H | 1.29-1.24* m, 26H | - | 6.82  d, *J* = 8.5 Hz, 2H |
| **6”a** | - | - | - | - | - |
| **6”b** | - | - | - | - | - |
| **7”** | 1.26- 1.20*  m, 19H | 1.30.-1.20 *  m, 23H | 1.29-1.24* m, 26H | - | 7.38-7.30  m, 3H |
| **7”a** | - | - | - | - | - |
| **7”b** | - | - | - | - | - |
| **8”** | 1.26- 1.20*  m, 19H | 1.30.-1.20*  m, 23H | 0.89-0.87  m, 8H | - | - |
| **9”** | 1.26- 1.20*  m, 19H | 1.30.-1.20 *  m, 23H | - | - | - |
| **10”** | 0.88-0.84  m, 4H | 1.30.-1.20 *  m, 23H | - | - | - |
| **11”** | - | 1.30.-1.20 *  m, 23H | - | - | - |
| **12”** | - | 0.86  t, *^3^J*_H11”-H12”_ =6.9 Hz, 3H | - | - | - |
| **1”’** | - | - | - | - | 3.85 – 3.80  m, 2H |
| **1”’a** | - | - | 2.78-2.73  m, 3H | 2.85-2.80  m, 2H | - |
| **1”’b** | - | - | 2.65-2.56  m, 3H | 2.71-2.65  m, 1H | - |
| **2”’** | - | - | 1.63-1.48  m, 5H | 0.99  q, *^3^J* _H1”-H2”, H1”’-H2”_= 6.7 Hz, 7H | 1.69-1.58  m, 2H |
| **3”’** | - | - | 1.29-1.24*  m, 26H | - | 1.40-1.32  m, 2H |
| **4”’** | - | - | 1.29-1.24*  m, 26H | - | 1.31 – 1.23*  m, 28H |
| **5”’** | - | - | 1.29-1.24*  m, 26H | - | 1.31 – 1.23*  m, 28H |
| **6”’** | - | - | 1.29-1.24*  m, 26H | - | 1.31 – 1.23*  m, 28H |
| **7”’** | - | - | 1.29-1.24*  m, 26H | - | 1.31 – 1.23*  m, 28H |
| **8”’** | - | - | 0.89-0.87  m, 8H | - | 1.31 – 1.23*  m, 28H |
| **9”’** | - | - | - | - | 1.31 – 1.23*  m, 28H |
| **10”’** | - | - | - | - | 0.86  t, *^3^J*_H9”’-10”’_=6.9  Hz, 5H |
| **1A** | - | - | - | - | 4.24-4.03  m, 4H |
| **3A** | - | - | - | - | 6.64-6.61  m,2H |
| **3AB** | - | - | - | - | 6.64-6.61  m,2H |
| **4A** | - | - | - | - | 7.06-7.02  m, 2H |
| **4AB** | - | - | - | - | 7.06-7.02  m, 2H |
| **6A** | - | - | - | - | 2.90  s, 4H |
| **6B** | - | - | - | - | 2.84  s, 10H |
| **9-OH^** | 5.26 | 5.27 | 5.25 | 5.21 | 5.30 |
| **HN^+^-H_a_** | 8.50 | 10.30 | - | - | 10.13 |
| **HN^+^-H_b_** | 8.20 | 9.50 | - | - | 9.67 |
| **H-N^+^** | - | - | 8.28 | 9.50 | - |

*-overlapped

^- determined from ^1^H-^1^H ROESY NMR

# Fig. S225 The **PIM** molecule in the **PIM2** crystal structure with the labelling scheme for non-hydrogen atoms. Displacement ellipsoids are shown at the 50% probability level.

# Fig. S226 The **PIM** molecule in the **PIM2** crystal structure with the labels shown for O and N atoms. atoms. Displacement ellipsoids are shown at the 20% probability level.

Fig. S227 O-H···O and N-H···O hydrogen bonding interactions connecting PIM molecules into a 1D network extending along the a axis in the crystal form **PIM2**. Only O and N atoms involved in hydrogen bonding interactions are labeled. The solvent molecules are omitted for clarity.

Fig. S228 O-H···O and N-H···O hydrogen bonding interactions connecting PIM molecules into a 1D network extending along the a axis in the crystal form **PIM1**: left – for the major conformer, right – for the minor conformer. Only O and N atoms involved in hydrogen bonding interactions are labeled.

# Fig. S229 Solvate accessible area in the crystal structure of **PIM1.**

# Table S7. Crystal data and details of structure refinement

| Compound reference | **PIM1** | **PIM2** |
| --- | --- | --- |
| CCDC no. | 2561067 | 2561066 |
| Chemical formula | C_33_H_47_NO_7_·unknown solvent | C_33_H_47_NO_7_·(CH_3_OH)_2.81_·  (H_2_O)_0.58_ |
| Formula Mass | 665.71 | 765.91 |
| Crystal system | Orthorhombic | Orthorhombic |
| *a*/Å | 8.1971(3) | 8.5666(5) |
| *b/* Å | 14.8717(5) | 18.6303(12) |
| *c/* Å | 33.0965(11) | 25.0629(15) |
| α/° | 90 | 90 |
| β/° | 90 | 90 |
| γ/° | 90 | 90 |
| Unit cell volume/Å^3^ | 4034.6(2) | 4000.0(4) |
| Temperature/K | 130 | 100 |
| Space group | *P*2_1_2_1_2_1_ | *P*2_1_2_1_2_1_ |
| No. of formula units per unit cell, Z | 4 | 4 |
| Radiation type | Cu *K*α | Cu *K*α |
| Absorption coefficient, μ/mm^-1^ | 0.706 | 0.841 |
| No. of reflections measured, 2Θ_max_/° | 9076, 102.8 | 57771, 145.0 |
| No. of independent reflections | 4105 | 7787 |
| *R*_int_ | 0.0663 | 0.0325 |
| Final *R_1_* values (*I*> 2σ(I)) | 0.0750 | 0.0372 |
| Final wR(F^2^) values (I>2σ(I)) | 0.2025 | 0.0906 |
| Final *R_1_* values (all data) | 0.0869 | 0.0446 |
| Final wR(F^2^) values (all data) | 0.2192 | 0.0944 |
| Goodness of fit on F^2^ | 1.012 | 1.020 |

# Fig. S230 Chromatograms of chemical stability of compound **3** in solutions of different pH with 2-thiophenecarboxylic hydrazide as internal standard.
